# Bis(pyridino‑*o*‑carboranyl)phosphenium Cation: A Platform for Dual-Site Reactivity

**DOI:** 10.1021/acs.inorgchem.6c01373

**Published:** 2026-06-29

**Authors:** Shiv Kumar, Deependra Bawari, Roman Dobrovetsky

**Affiliations:** a School of Chemistry, Raymond and Beverly Sackler Faculty of Exact Sciences, 208982 Tel Aviv University, Tel Aviv 69978, Israel; b Department of Chemistry, School of Basic Sciences, Central University of Haryana, Mahendergarh, 123031 Haryana, India

## Abstract

Phosphenium salt, [**L**_**2**_ **P** ^**+**^]­[B­(C_6_F_5_)_4_], substituted by two pyridino-*ortho*-carboranyl groups (**L**), was prepared, fully characterized, and its preliminary reactivity with small molecules was studied. Surprisingly, cation [**L**_**2**_ **P** ^**+**^]­[B­(C_6_F_5_)_4_] reacts differently with O–H and N–H bonds. With RO–H (R = H, Me, Ph), the reaction takes place at P center in [**L**_**2**_ **P** ^**+**^]­[B­(C_6_F_5_)_4_], via hydrolysis or alcoholysis of the P–C bonds, leading to the carboranopyridinium salt ([**LH**_**2**_^**+**^]­[B­(C_6_F_5_)_4_]) and the complementary (RO)_2_P­(O)­H. In contrast, the reaction of [**L**_**2**_ **P** ^**+**^]­[B­(C_6_F_5_)_4_] with R_2_N–H (Et_2_NH, Ph­(H)­NH, ^*i*^Pr­(H)­NH) does not proceed at the P center, but takes place at the *ortho*-carboranyl (*o*Cb) moiety by deboronation, producing zwitterionic phosphenium (**L­(L** ^**nido**^ **)­P** ^**+**^), with significant reduction in the reactivity of the P center compared to **L**_**2**_ **P** ^**+**^. Remarkably, [**L**_**2**_ **P** ^**+**^]­[B­(C_6_F_5_)_4_] reacts with SO_2_ in MeCN at both sites, the P and *o*Cb, stripping both oxygens from sulfur, leading to zwitterionic phosphonium sulfide (**L­(L** ^**nido**^ **)­P** ^**+**^ **=S**).

## Graphical abstract

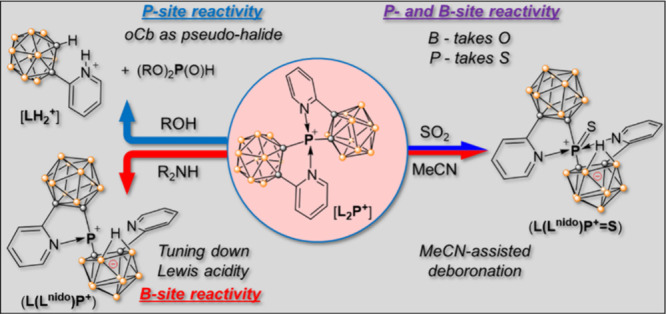

## Introduction

Transition metals (TMs) are well-known for their ability to activate small molecules and for their central role in catalysis.^1^ For a long time, this reactivity was considered the exclusive domain of TMs, but over the past two decades, this dogma has been challenged by significant advances in main-group (MG) chemistry.^2^ A variety of intriguing low-valent MG compounds capable of engaging in small-molecule activation have been prepared,^3–6^ and the introduction of frustrated Lewis pairs (FLPs) propelled the field further, establishing MG species as potential alternatives for small-molecule activation and catalysis.^7^

In recent years, the concept of applying structural constraints (SC) to MG centers has attracted considerable attention as an effective strategy for generating highly reactive MG compounds capable of small-molecule activation.^8^ Among structurally constrained MG centers, phosphorus stood out for its ability to access two stable oxidation states, typically cycling between P^III^ and P^V^.^9^ Several structurally constrained neutral P^III^ species have been reported and shown to activate various small molecules; however, the catalysis using these species was rather limited.^10^ In contrast, structurally constrained phosphenium cations (**I–III**) introduced by our group (Figure 1) were found to be capable not only of small-molecule activation but also of metallomimetic catalysis, involving analogous key steps such as “oxidative addition” (OA) → “ligand metathesis” (LM) → “reductive elimination” (RE).^11^ For example, SC P^III^ cation **I** has been employed in the metallomimetic hydrosilylation of aldehydes (Figure 1);^12a^ **II** was used in the hydrodefluorination and amination of electron-poor fluoroarenes (Figure 1).^12b^ Since then, other groups have also become interested in this approach and reported the synthesis of SC phosphenium cations that showed reactivity toward strong bonds.^13^

**Figure 1:**
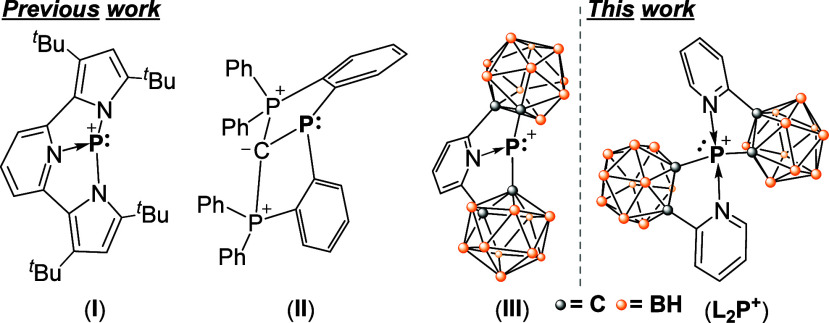
Examples of previously reported SC P^III^ cations (**I–III**), and P^III^ cation (**L**_2_ **P** ^+^) reported in this work.

In recent years, *o*Cb-based ligands have emerged as powerful tools for enhancing the reactivity of MG centers, particularly by amplifying their Lewis acidity. Such as exemplified by Wang and co-workers, who strategically designed an *o*Cb-substituted borenium cation that enabled activation of inert bonds, including H_3_C–H.^14^ Martin reported the synthesis of *o*Cb_3_B and *o*Cb_2_BH benchmark Lewis superacids, noted for their exceptional reactivity.^15^ Ye reported 3D analogues of 9,10-diboraanthracene and 9-borafluorene that operate as neutral, boron-centered Lewis superacids, expanding the structural and functional diversity of this class.^16^ Peryshkov demonstrated the redox-active potential of *o*Cb scaffolds, enabling N–H bond activation in ammonia using an *ortho*-carboranyl diphosphine.^17^ Very recently, Dutton reported the first *o*Cb-supported hypervalent iodine­(III) type species, which was found to be more reactive than any known aryl hypervalent iodine­(III) compound.^18^

Recently, our group used the high electron-withdrawing ability of *o*Cb to synthesize SC phosphenium cation **III** (Figure 1), which was used for the hydrogenation of C=C double bonds and fused aromatic systems.^19^ Motivated by this result, we aimed at synthesizing a modified version of **III**, **L**_**2**_ **P** ^**+**^, in which the strain is slightly released, and an additional pyridinyl donor is attached to increase the stability of this phosphenium cation (Figure 1). We envisioned that one of the pyridyl donors in **L**_**2**_ **P** ^**+**^ could be detached from the P center by heating, exposing the ambiphilicity of this center.

Herein, we report the synthesis, isolation, and preliminary reactivity studies of [**L**_**2**_ **P** ^**+**^]­[B­(C_6_F_5_)_4_]. Interestingly, unlike previous P^III^ cationic and neutral analogs, [**L**_**2**_ **P** ^**+**^]­[B­(C_6_F_5_)_4_] reacts differently with O–H and N–H bonds; while the former reacts with the cationic P^III^ center, the latter reacts at the *o*Cb moiety. Remarkably, in the reaction with SO_2_ in MeCN, both sites (P and *o*Cb) react, stripping both oxygen atoms of sulfur.

## Results and Discussion

First, we decided to prepare **L**_**2**_ **PCl**, which, after Cl^–^ abstraction, should lead to the desired **L**_**2**_ **P** ^**+**^ (Scheme 1). To synthesize **L**_**2**_ **PCl**, we first deprotonated pyridinocarborane^20^ (**LH**) with ^*n*^BuLi, and then reacted it with 0.5 equiv of PCl_3_ in a one-pot reaction. This, however, did not result in the formation of **L**_**2**_ **PCl** but led to a mixture of products. We, thus, decided to attempt the stepwise approach. First, we reacted deprotonated **LH** (**LLi**) with a slight excess of PCl_3_ (1.1 equiv), which cleanly led to **LPCl**_**2**_ (Scheme 1). **LPCl**_**2**_ was isolated in 89% yield by crystallization from CHCl_3_. The molecular structure of **LPCl**_**2**_ was determined by single-crystal X-ray diffraction (SC-XRD) (Figure 2a).^21^ Importantly, the P–N distance in **LPCl**_**2**_ of 2.335 Å indicates the absence of a bonding interaction between these two centers. Isolated **LPCl**_**2**_ was reacted with an additional equivalent of **LLi**; this, as well, did not yield the target compound **L**_**2**_ **PCl**. An in situ ^31^P NMR revealed a single new phosphorus-containing species, displaying a sharp singlet at 76.8 ppm. While in the ^1^H and ^13^C NMR spectra, in addition to the expected signals of the pyridine moieties, several unexpected upfield signals were also observed. ^1^H–^13^C HSQC and HMBC NMR experiments showed a doublet of doublets at 4.63 ppm in the ^1^H NMR spectrum (*J* = 12 Hz, 6 Hz) correlated to a doublet at 56.42 ppm in the ^13^C NMR spectrum (*J*_C–P_ = 19 Hz), indicative of an aliphatic CH fragment. Additionally, signals at 6.21, 5.60, and 5.39 ppm in the ^1^H NMR spectrum correlated to resonances at 125.1, 104.8, and 117.4 ppm in the ^13^C NMR spectrum. These data suggested the presence of a conjugated olefinic moiety (see Figures S8–S15). Overall, the spectral data indicated the formation of an unsymmetrical species featuring a dearomatized pyridine ring.

**Scheme 1:**
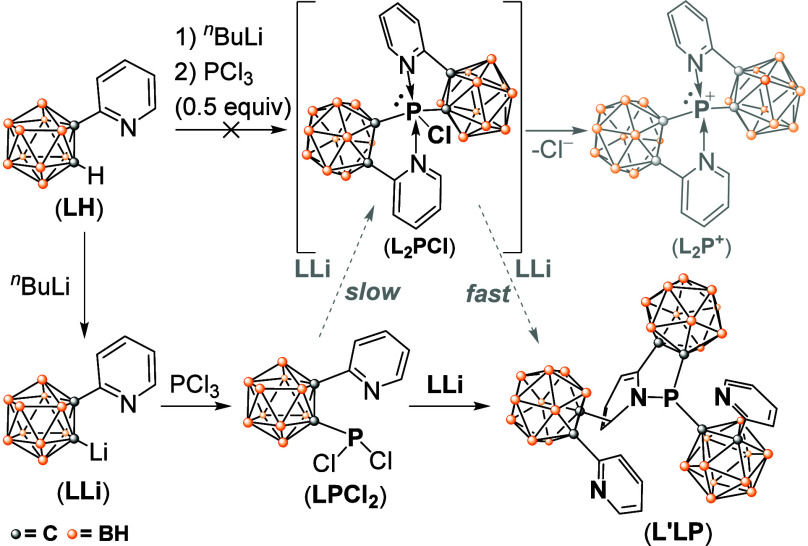
Attempted Synthesis of **L**_**2**_ **PCl**, Formation of **L’LP** via a Reaction of **LPCl**_**2**_ and **LLi**

**Figure 2:**
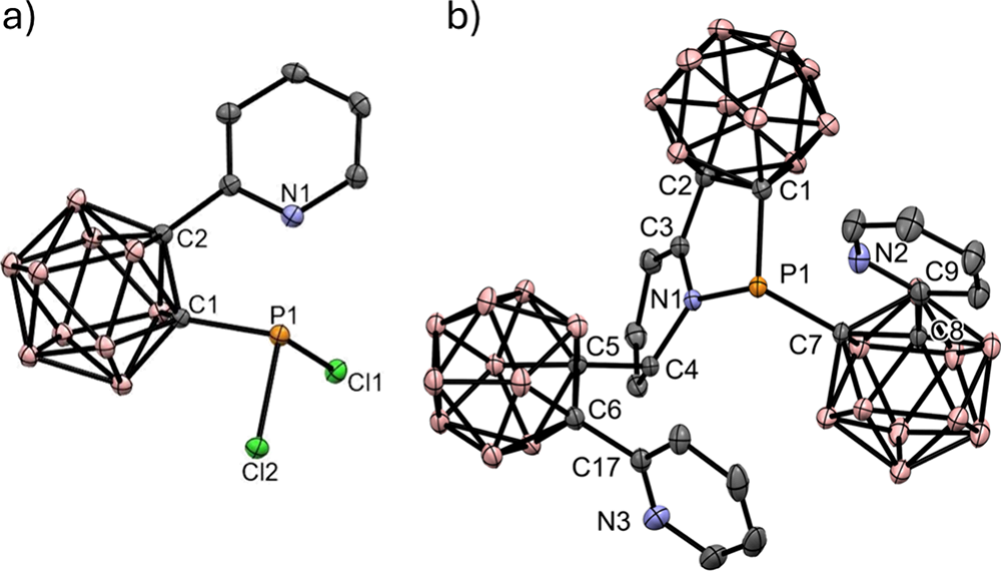
Molecular structure of (a) **LPCl**_2_, and (b) **L’LP**. Thermal ellipsoids have been adjusted to the 50% probability level; hydrogen atoms were omitted for clarity.

The clean product was isolated by crystallization from toluene. The SC-XRD of these crystals confirmed the identity of the product as **L’LP** (Figure 2b). The phosphorus center is bonded to two carbons (P1–C1 = 1.891 Å, P1–C7 = 1.940 Å) from two ligands and to a nitrogen (P1–N1 = 1.754 Å) of the dearomatized pyridine ring. The dearomatization is evident from the puckering of the ring and elongation of the C4–N1 (1.470 Å) and adjacent C–C (1.508 Å) bond distances (Figure 2b).

The formation of compound **L’LP** likely originates from the slow generation of **L**_**2**_ **PCl**, which, upon formation, reacts rapidly with a second equivalent of **LLi** (Scheme 1). The observed regioselectivity can be attributed to the LUMO being localized at the ortho position (see ESI, Figure S74).

The inability to obtain **L**_**2**_ **PCl** and the formation of **L’LP** prompted us to try to get the desired cation **L**_**2**_ **P** ^**+**^ from it. We hypothesized that, upon heating, the **L’LP** system could dissociate into the desired **L**_**2**_ **P** ^**+**^ with the **L** ^**–**^ counteranion, driven by rearomatization of the pyridine ring. If the **L** ^**–**^ could be trapped, then the desired **L**_**2**_ **P** ^**+**^ could be obtained. We thus decided to react **L’LP** with MeOTf. As a result, after 12 h, we were pleased to find that a reaction of **L’LP** with MeOTf resulted in the formation of the desired [**L**_**2**_ **P** ^**+**^]­[OTf] and the complementary **LMe** at r.t. (Scheme 2). This transformation was evidenced by the appearance of a new signal at 30 ppm (CH_3_CN) in the ^31^P NMR spectrum and the emergence of a more symmetric pattern in both ^1^H and ^13^C NMR spectra, accompanied by the complete disappearance of features associated with the dearomatized pyridine moiety. The observed downfield shift in the ^31^P NMR signal is significantly lower than that reported for previously characterized cationic P^III^ species (δ > 100 ppm),^12,13,19^ suggesting a more electron-rich phosphorus center in **L**_**2**_ **P** ^**+**^. This may be attributed to additional coordination at the phosphorus center by the nitrogen atom of a second pyridine ring.

**Scheme 2:**
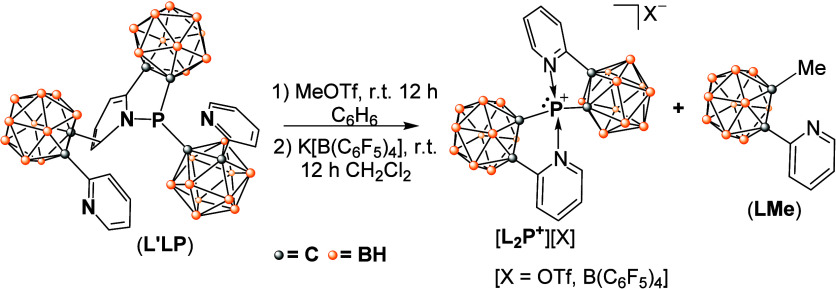
Synthesis of [**L**_**2**_ **P** ^**+**^]­[B­(C_6_F_5_)_4_]

Due to the poor solubility of [**L**_**2**_ **P** ^**+**^]­[OTf], the TfO^–^ was replaced by the (C_6_F_5_)_4_B^–^ anion by an ion exchange reaction of [**L**_**2**_ **P** ^**+**^]­[OTf] with K­[B­(C_6_F_5_)_4_], affording [**L**_**2**_ **P** ^**+**^]­[B­(C_6_F_5_)_4_] (Scheme 2). [**L**_**2**_ **P** ^**+**^]­[B­(C_6_F_5_)_4_] was purified by crystallization from a CH_2_Cl_2_:CHCl_3_ mixture (1:1.5), and its molecular structure was determined by SC-XRD (Figure 3).

**Figure 3:**
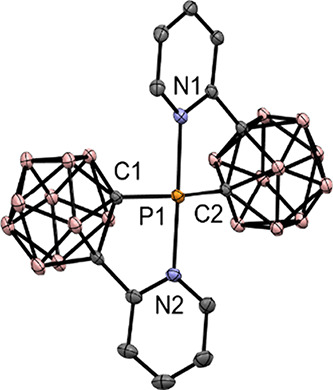
Molecular structure of [**L**_2_ **P** ^+^]­[B­(C_6_F_5_)_4_]. Thermal ellipsoids have been adjusted to the 50% probability level; hydrogen atoms and (C_6_F_5_)_4_B^–^ were omitted for clarity.

The phosphorus center in the **L**_**2**_ **P** ^**+**^ cation adopts a tetracoordinate, slightly distorted seesaw geometry, with bond angles of ∠C1–P1–C2 = 108.81° and ∠N1–P1–N2 = 173.84°. The P1–C1 and P1–C2 (1.910 and 1.913 Å, respectively) bond lengths are slightly longer than a typical covalent P–C bond length,^19^ while the P1–N1 and P1–N2 (2.054 and 2.031 Å, respectively) are significantly longer than typical covalent P–N bonds.^11a,18^ As well as elongated considerably in comparison to the coordinative P→N bond lengths in previously reported SC phosphenium cations.^12,13,19^ This elongation is likely a consequence of the 3c-4e interaction involving the axial pyridine ligands, which weakens the N→P interactions via donation along the coordination axis. Notably, a similar bonding motif has previously been reported for similar species.^22^

To gain deeper insight into the electronic structure of cation **L**_**2**_ **P** ^**+**^, density functional theory (DFT) calculations were performed at the B3LYP-D3­(BJ)/6–311++G­(d,p) level of theory.^23,24^ The optimized geometry of **L**_**2**_ **P** ^**+**^ is in good agreement with its experimental X-ray molecular structure (see Table T1 in ESI). Frontier molecular orbital (FMO) analysis reveals that the HOMO is primarily localized on the phosphorus center, whereas the LUMO is distributed over the π*-orbitals of the pyridine rings (Figure 4a). Natural Bond Orbital (NBO) analysis indicates a σ-type lone pair on the phosphorus atom with an occupancy of 1.93 electrons and sp^0.52^ hybrid character. Natural Population Analysis (NPA) charges show a significant positive charge on phosphorus (+1.315) and negative charges on nitrogen (−0.544) and carbon (−0.687) atoms.

**Figure 4:**
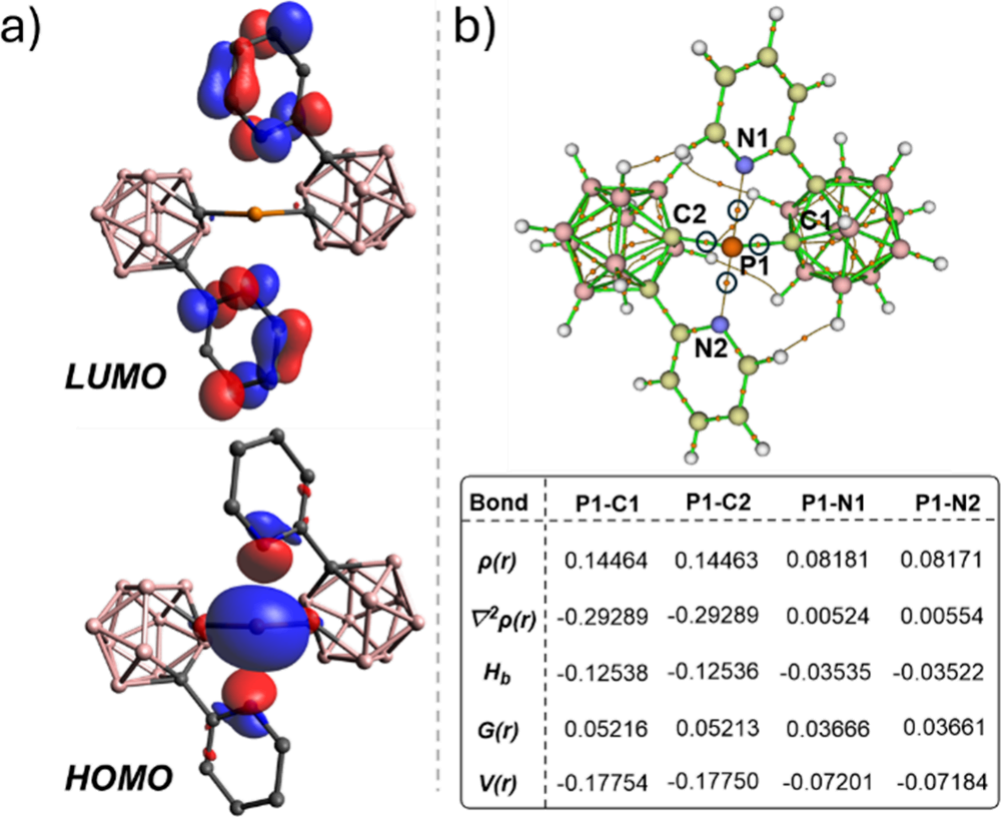
a) HOMO and LUMO orbitals (isovalue 0.05) of **L**_2_ **P** ^+^; b) AIM analysis of **L**_2_ **P** ^+^.

The Wiberg Bond Index (WBI)^25^ values indicate partial single-bond character for the P–N bonds (0.383), while the P–C bonds (0.782) exhibit predominantly single-bond character. Bader’s Atoms in Molecules (AIM) analysis further clarifies bonding interactions: the bond critical point (BCP) of the P1–N1 bond exhibits a positive Laplacian of electron density (∇^2^ρ = 0.00524), low electron density (ρ = 0.0818), the relationships *G­(r)* ≪ |*V*(*r*)| and a negative total energy density normalized by electron density (*H*_b_ */ρ­(r)* = – 0.4320) at the BCP, consistent with a donor–acceptor type interaction (Figure 4b).^26^ In contrast, the AIM parameters for the P–C bonds are indicative of covalent bonding. Collectively, these computational and experimental findings corroborate the description of **L**_**2**_ **P** ^**+**^ as a bipyridyl-supported phosphenium cation.

Having [**L**_**2**_ **P** ^**+**^]­[B­(C_6_F_5_)_4_] in hand, we started studying its reactivity with small molecules. First, [**L**_**2**_ **P** ^**+**^]­[B­(C_6_F_5_)_4_] was reacted with one equiv of H_2_O in CDCl_3_, and the reaction progress was monitored by ^31^P NMR spectroscopy at r.t., after 5 min approximately 40% of [**L**_**2**_ **P** ^**+**^]­[B­(C_6_F_5_)_4_] was consumed. The ^31^P NMR spectrum was recorded, which showed a doublet at 13.53 ppm (*J­(PH)* = 646 Hz), indicating the formation of a P–H moiety, which was corroborated by a corresponding doublet at 7.36 ppm (*J­(PH)* = 646 Hz) in the ^1^H NMR spectrum. After 30 min, the NMR picture remained the same, with [**L**_**2**_ **P** ^**+**^]­[B­(C_6_F_5_)_4_] still present in the reaction mixture. The addition of excess (5 equiv) H_2_O resulted in complete consumption of [**L**_**2**_ **P** ^**+**^]­[B­(C_6_F_5_)_4_]. Interestingly, the ^1^H NMR spectrum also displayed a broad singlet at 15.94 ppm, attributed to a pyridinium N–H fragment. These resonances are consistent with the formation of the protonated ligand **LH**_**2**_^**+**^. Over time, crystals precipitated from the reaction mixture, and the SC-XRD analysis confirmed the formation of [**LH**_**2**_^**+**^]­[B­(C_6_F_5_)_4_] (see Figure S30). Overall, combined ^1^H and ^31^P NMR data and the isolation of [**LH**_**2**_^**+**^]­[B­(C_6_F_5_)_4_] clearly indicate that the P-containing product of this reaction is (HO)_2_P­(O)H (Scheme 3).^27,28^ Notably, analogous reactivity was observed with MeOH and PhOH, yielding the corresponding alcoholysis products, (MeO)_2_P­(O)H and (PhO)_2_P­(O)­H,^27,28^ respectively; however, unlike in the case with H_2_O, heating to 85 °C was required for these reactions to proceed (see ESI for more details).

**Scheme 3:**
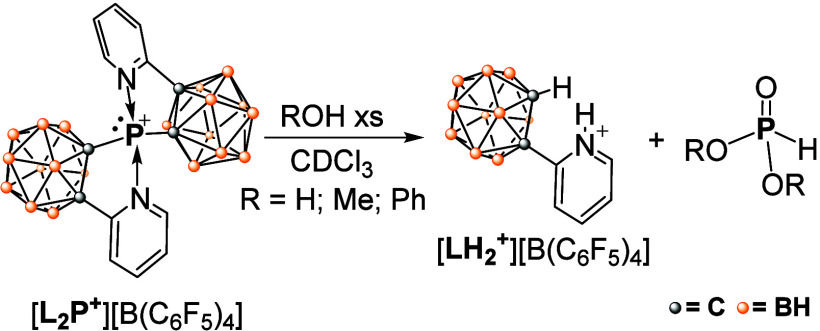
Reactivity of [**L**_**2**_ **P** ^**+**^]­[B­(C_6_F_5_)_4_] with ROH (R = H, Me, Ph)

This type of reactivity is very unusual for P–C bonds in P^III^ compounds, which are typically robust and resistant to hydrolysis and, more so, to alcoholysis.^28^ The observed reactivity is usually associated with P–X (X = Cl, Br, I), meaning that in the case of [**L**_**2**_ **P** ^**+**^]­[B­(C_6_F_5_)_4_], the *o*Cb moiety acts as a pseudohalide.^29,30^ To examine whether this behavior is intrinsic to the P–C bonds in phosphinocarboranes, we treated Ph_2_P­(*o*Cb) with H_2_O and MeOH. No hydrolysis or alcoholysis of Ph_2_P­(*o*Cb) was observed, even after prolonged heating (see ESI Scheme S12 for more details). This indicates that the observed hydrolysis (alcoholysis) of **L**_**2**_ **P** ^**+**^ represents an atypical case rather than a general property of phosphinocarboranes. The hydrolysis (alcoholysis) of the P–C bonds in **L**_**2**_ **P** ^**+**^ likely arises due to their high polarity (P^δ+^–C^δ−^) along with the stabilization of the cationic intermediates by the pyridinyl moiety (see ESI for more details, Figure S44).

Next, we tested the reactivity of [**L**_**2**_ **P** ^**+**^]­[B­(C_6_F_5_)_4_] with amines. First, a reaction between [**L**_**2**_ **P** ^**+**^]­[B­(C_6_F_5_)_4_] and an equiv of Et_2_NH was performed in CDCl_3_, and its progress was monitored using ^31^P NMR spectroscopy at r.t.. In ^31^P NMR, after 1 h, the signal at 36 ppm, corresponding to **L**_**2**_ **P** ^**+**^, decreased, and a new signal appeared at 78 ppm. After 3 h, an additional signal at 69 ppm emerged, with approximately 50% of [**L**_**2**_ **P** ^**+**^]­[B­(C_6_F_5_)_4_] consumed. Two additional equiv of Et_2_NH were added, which finally led to 69 ppm as the major signal, while the signals at 35 and 78 ppm completely disappeared (see ESI).

Upon completion of the reaction, CDCl_3_ was evaporated, and the remaining solid was washed with C_6_H_6_. The ^31^P NMR of both fractions was measured, showing that only C_6_H_6_ had P-containing products dissolved in it. Slow evaporation of the C_6_H_6_ resulted in the formation of crystals. SC-XRD analysis of these crystals revealed the molecular structure of the product as zwitterionic phosphenium **L­(L** ^**nido**^ **)­P** ^**+**^ (Figure 5). Interestingly, a number of zwitterionic P­(I) compounds have been previously reported,^13c‑d^ while the zwitterionic phosphenium cations are rare.^31^

**Figure 5:**
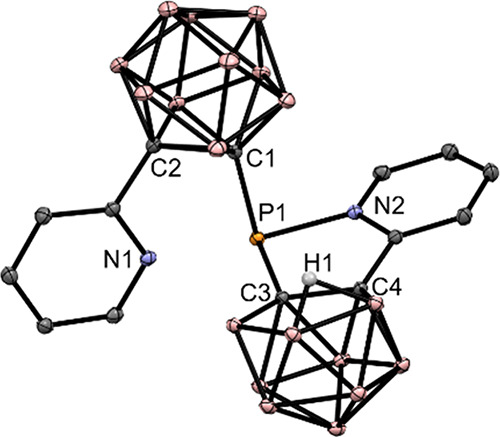
Molecular structure of **L­(L** ^nido^ **)­P** ^+^. Thermal ellipsoids have been adjusted to the 50% probability level; nonrelevant hydrogen atoms were omitted for clarity.

The formation of **L­(L** ^**nido**^ **)­P** ^**+**^ as the only P-containing product indicates that Et_2_NH reacted with the *o*Cb moiety via a deboronation reaction,^32,33^ and not at the P^III^ cationic center (Scheme 4). Importantly, the reaction of [**L**_**2**_ **P** ^**+**^]­[B­(C_6_F_5_)_4_] with other amines, such as PhNH_2_, ^*i*^PrNH_2,_ and 2,4,6-trimethylaniline, as well as with MeCN, also afforded **L­(L** ^**nido**^ **)­P** ^**+**^, but required heating to 85 °C (Scheme 4). It is important to note that, unlike in the reaction of [**L**_**2**_ **P** ^**+**^]­[B­(C_6_F_5_)_4_] with amines, where changes in the ^11^B NMR spectra were observed and attributed to formation of a B3-amine adduct,^32,33^ no significant changes in the ^11^B NMR spectra were detected at any stage during the reactions of [**L**_**2**_ **P** ^**+**^]­[B­(C_6_F_5_)_4_] with ROH (R = H, Me, Ph) (see Figures S32 and S33). These observations indicate that, unlike amines, which react directly with the *o*Cb cage in [**L**_**2**_ **P** ^**+**^], water and alcohols preferentially react at the P center.

**Scheme 4:**
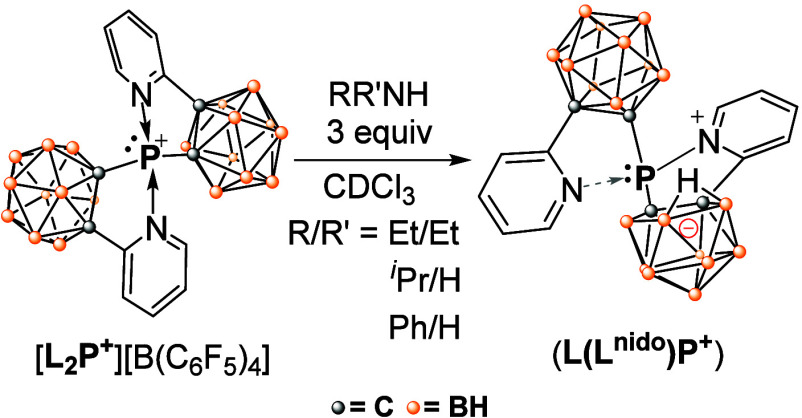
Reactivity of [**L**_**2**_ **P** ^**+**^]­[B­(C_6_F_5_)_4_] with RR’NH (RR’NH = Et_2_NH, PhNH_2_, and iPrNH_2_) Formation of **L­(L** ^**nido**^ **)­P** ^**+**^

The structure of **L­(L** ^**nido**^ **)­P** ^**+**^ features a tetracoordinate phosphorus center that adopts a seesaw geometry, with key bond angles of ∠C1–P1–C3 = 105.02° and ∠N1–P1–N2 = 168.93°. The P1–N2 and P1–C3 (1.947 and 1.868 Å, respectively) bond lengths are shorter than the P1–N1 and P1–C1 (2.180 and 1.922 Å, respectively) bond lengths, indicating a stronger bonding of the *nido o*Cb fragment to the P center. This can be explained by the fact that the *nido* cluster is anionic, and thus more electron-rich compared to *closo o*Cb, which results in a stronger donor ability of the adjacent pyridine nitrogen,^34^ while the pyridine nitrogen bound to the electron-withdrawing *closo o*Cb remains a weak donor.

To compare the electronic structures of **L**_**2**_ **P** ^**+**^ and **L­(L** ^**nido**^ **)­P** ^**+**^, the structure of **L­(L** ^**nido**^ **)­P** ^**+**^ was DFT computed (B3LYP-D3­(BJ)/6–311++G­(d,p)).^23,24^ The optimized geometry is in good agreement with the experimentally determined solid-state structure (see ESI Table T2). MO analysis reveals that, in contrast to **L**_**2**_ **P** ^**+**^, where the phosphorus lone pair constitutes the HOMO, in **L­(L** ^**nido**^ **)­P** ^**+**^, the lone pair at phosphorus corresponds to the HOMO–1­(Figure 6), while the HOMO is primarily localized on the *nido*-carborane fragment (see ESI, Figure S73). The LUMO is mainly distributed over the π* system of the pyridine ring attached to the *nido*-carborane moiety (Figure 6). NBO analysis indicates the presence of a σ-type lone pair at phosphorus with an occupancy of 1.94 electrons and an sp^0.58^ hybridization. NPA charges show that P1 bears a positive charge of +1.261, which is slightly lower than in **L**_**2**_ **P** ^**+**^ (+1.315). Both N1 and N2 are negatively charged (−0.501 and – 0.587, respectively). The WBI values highlight the asymmetric bonding environment at phosphorus. The P1–N2 bond exhibits a WBI of 0.4913, whereas the P1–N1 bond shows a significantly lower value of 0.2532, indicating a stronger P1–N2 interaction and a weaker P1–N1 bond. This asymmetry reflects the electronic consequences of the deboronation process - conversion of the *closo* cluster into the *nido* form strengthens the P1–N2 interaction.

**Figure 6:**
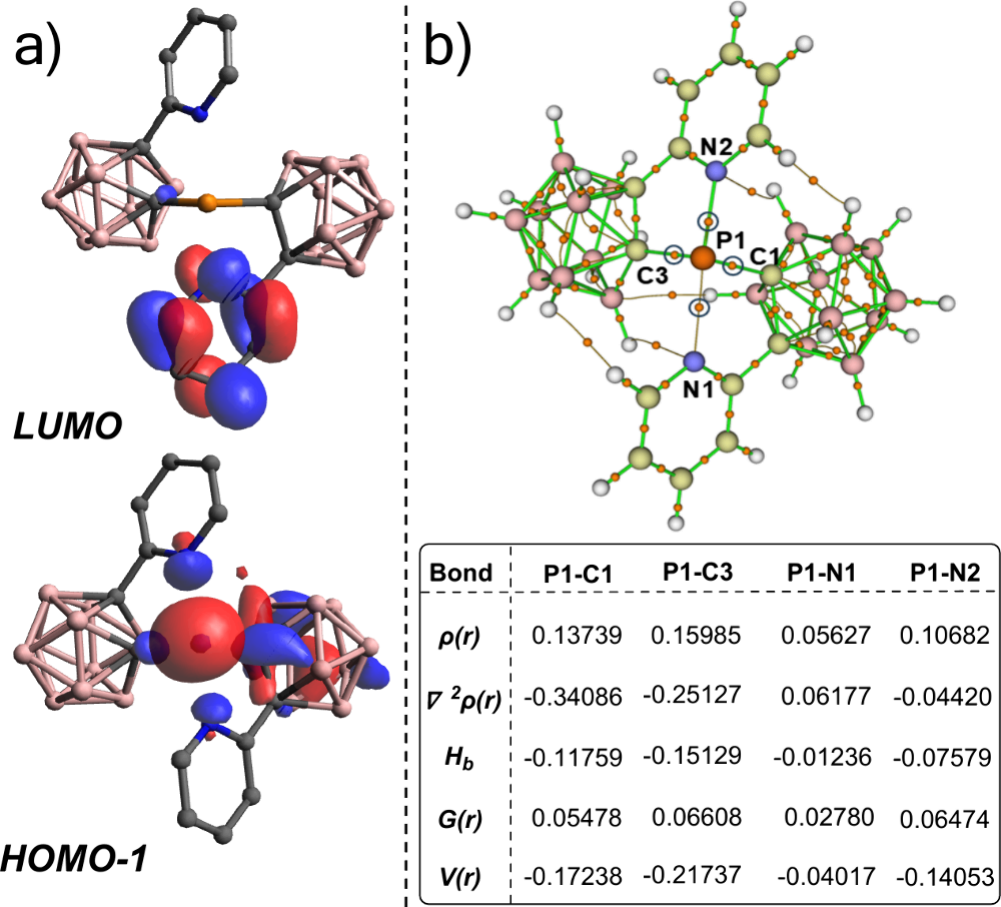
(a) HOMO–1 and LUMO orbitals (isovalue 0.05) of **L­(L** ^nido^ **)­P** ^+^; (b) AIM analysis of **L­(L** ^nido^ **)­P** ^+^.

AIM analysis further supports this picture. The bond critical point (BCP) for P1–N1 shows a positive Laplacian of the electron density (∇^2^ρ = +0.06177) and relatively low electron density (ρ = 0.05627), consistent with a weak coordination-type interaction. In contrast, the P1–N2 BCP exhibits a near-zero, slightly negative Laplacian (∇^2^ρ = – 0.004420) and a significantly higher electron density (ρ = 0.10682) (Figure 6), indicative of a strong coordinative bond with substantial covalent character. Collectively, these computational data indicate that the P1–N1 interaction is best described as a weak donor–acceptor contact, whereas P1–N2 represents a strong coordinative bond with pronounced covalent character.

These structural and electronic differences suggest that the reactivity of **L­(L** ^**nido**^ **)­P** ^**+**^ should differ significantly from that of the parent phosphenium cation **L**_**2**_ **P** ^**+**^, potentially accounting for their distinct reactivity profiles. This expectation was confirmed experimentally. A catalytic amount of **L**_**2**_ **P** ^**+**^ (5 mol %) rapidly polymerizes THF at room temperature within approximately 10 min (Scheme 5),^35^ dimerizes 1,1-diphenylethylene at r.t. after 24 h (Scheme 5),^36^ and immediately catalyzes (5 mol %) the cyanosilylation of benzaldehyde using TMSCN (Scheme 5). In contrast, **L­(L** ^**nido**^ **)­P** ^**+**^ is completely inactive toward THF and 1,1-diphenylethylene under identical conditions and does not promote cyanosilylation (Scheme 5).^37^ These findings indicate that B-vertex decapitation and formation of **L­(L** ^**nido**^ **)­P** ^**+**^ substantially reduce the Lewis acidity relative to **L**_**2**_ **P** ^**+**^ (see ESI for a comparison of the FIA values of **L**_**2**_ **P** ^**+**^, **L­(L** ^**nido**^ **)­P** ^**+**^, and other reported phosphenium cations), thereby suppressing its catalytic activity.

**Scheme 5:**
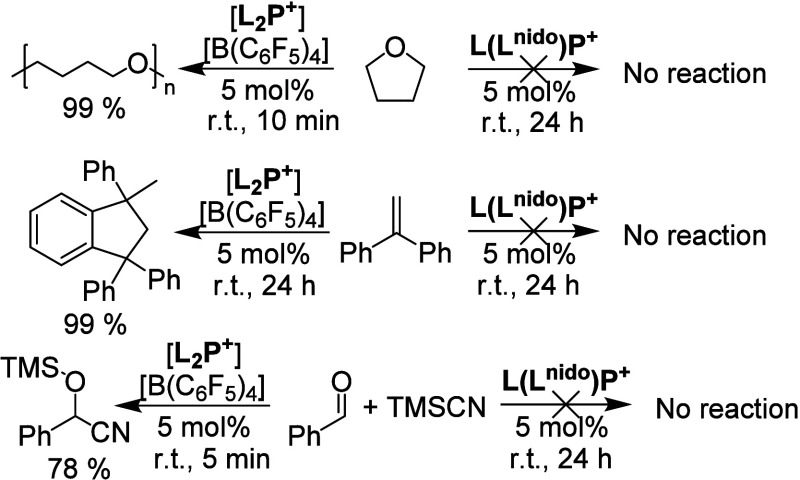
Experimental Differences in the Reactivities of **L**_**2**_ **P** ^**+**^ vs **L­(L^nido^)­P** ^**+**^

Importantly, the finding that deboronation of the *o*Cb substituent directly impacts the reactivity of the MG element bound to it highlights an attractive strategy for reactivity control. For instance, selective deboronation, as in the conversion of **L**_**2**_ **P** ^**+**^ to **L­(L** ^**nido**^ **)­P** ^**+**^, can be employed as a deliberate tool to “tune down” or completely “shut down” the Lewis acidity without altering much the core structure of the ligand framework. This approach introduces an additional level of flexibility in ligand design, enabling precise modulation of reactivity profiles in catalysis and small-molecule activation.

In an effort to probe the reactivity of **L**_**2**_ **P** ^**+**^ at both sites (the P and *o*Cb), we reacted [**L**_**2**_ **P** ^**+**^]­[B­(C_6_F_5_)_4_] with SO_2_, an ambiphilic substrate, in CD_3_CN and monitored the reaction progress by ^31^P NMR spectroscopy (Scheme 6). No reaction was observed at r.t., however, after heating at 100 °C for 3 h, in addition to the signal at δ = 31 ppm (in CD_3_CN) corresponding to **L**_**2**_ **P** ^**+**^, a new signal appeared at δ = – 1.9 ppm (see ESI, Figure S53). After 6 h, another signal appeared at 26.4 ppm, which, after 48 h, was the only signal in ^31^P NMR.

**Scheme 6:**
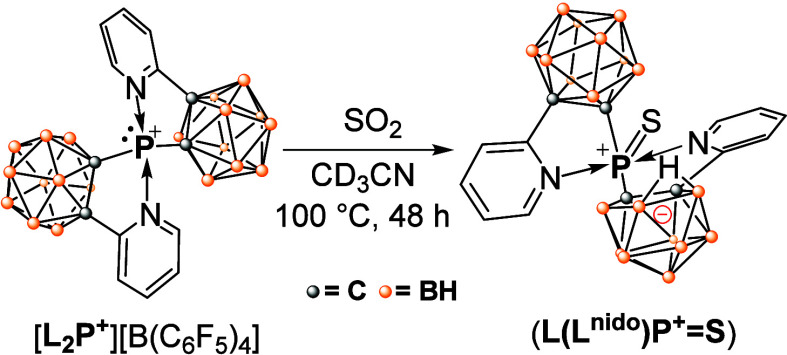
Reactivity of [**L**_**2**_ **P** ^**+**^]­[B­(C_6_F_5_)_4_] with SO_2_ at Both Reactive Sites, the P Center and the *o*Cb Moiety, Leading to **L­(L** ^**nido**^ **)­P** ^**+**^ **=S**

Over time, crystals precipitated from the reaction mixture (CD_3_CN). The SC-XRD analysis of these crystals revealed their identity as a *nido*-zwitterionic phosphonium sulfide species (**L­(L** ^**nido**^ **)­P** ^**+**^ **=S**) (Figure 7). In contrast to **L­(L** ^**nido**^ **)­P** ^**+**^, compound **L­(L** ^**nido**^ **)­P** ^**+**^ **=S** exhibits only slight differences between the P–N and P–C bond lengths: P1–N1 = 2.030 Å, P1–N2 = 1.980 Å, and P1–C1 = 1.890 Å, P1–C8 = 1.860 Å. This can be explained by the fact that the P^V^ cationic center in **L­(L** ^**nido**^ **)­P** ^**+**^ **=S** is significantly more electron-poor compared to the P^III^ cationic center in **L­(L** ^**nido**^ **)­P** ^**+**^, which results in a contraction of the substituents toward the phosphorus atom. The P=S bond length is 1.938 Å, consistent with a typical P=S double bond.^38^

**Figure 7:**
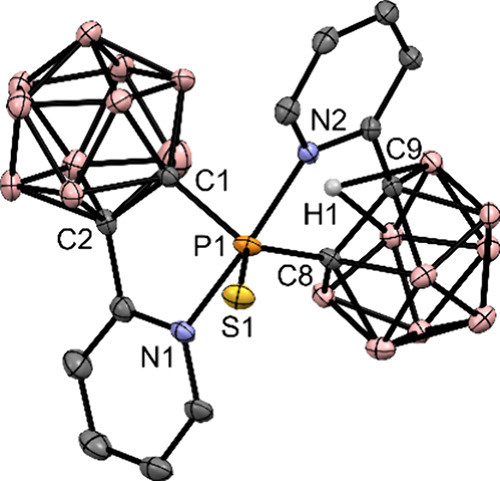
Molecular structure of **L­(L^nido^)­P** ^+^ **=S**. Thermal ellipsoids have been adjusted to the 50% probability level; nonrelevant hydrogen atoms were omitted for clarity.

The formation of **L­(L** ^**nido**^ **)­P** ^**+**^ **=S** could potentially be explained by cooperative engagement of the two reactive sites present in **L**_**2**_ **P** ^**+**^: the P^III^ center and the *o*Cb moiety. We speculate that, rather than acting independently, these sites operate synergistically, and that this dual-site reactivity, assisted by MeCN-induced deboronation (see above) of **L**_**2**_ **P** ^**+**^, ultimately enables the cleavage of both S=O bonds. It is important to note that the fate of the boron species generated upon *o*Cb decapitation is difficult to follow; however, related species have previously been shown to deoxygenate organic molecules.^32,33^ To support this hypothesis, we conducted two control experiments: (i) the reaction of isolated **L­(L** ^**nido**^ **)­P** ^**+**^ with SO_2_ in CH_2_Cl_2_ (see ESI), and (ii) the in situ formation of **L­(L** ^**nido**^ **)­P** ^**+**^ from **L**_**2**_ **P** ^**+**^ and MeCN, followed by the addition of SO_2_ (see ESI). In both cases, **L­(L** ^**nido**^ **)­P** ^**+**^ **=S** was not formed. Instead, prolonged heating of both reactions at 100 °C resulted in a complex mixture of products (see ESI, Figures S54 and S55). Although this proposal remains preliminary and more detailed mechanistic studies are required to fully elucidate the reaction pathway, these experiments support our hypothesis that, in the direct reaction of **L**_**2**_ **P** ^**+**^ with SO_2_ in MeCN, both the P and *o*Cb sites act cooperatively.

## Conclusions

In summary, we synthesized, isolated, and characterized the phosphenium salt [**L**_**2**_ **P** ^**+**^]­[B­(C_6_F_5_)_4_]. Preliminary reactivity studies of [**L**_**2**_ **P** ^**+**^]­[B­(C_6_F_5_)_4_] showed that it has two reactive sites: one is the cationic P^III^ center, and the second is the *o*Cb moiety. In reactions with ROH (R = H, Me, Ph), **L**_**2**_ **P** ^**+**^ reacts through the P center, leading to the hydrolysis (or alcoholysis) of the P–C bonds, giving (RO)_2_P­(O)H (R = H, Me, Ph). In contrast, **L**_**2**_ **P** ^**+**^ reacted with R_2_NH (R_2_NH = Et_2_NH, PhNH_2_, ^*i*^PrNH_2_) through deboronation of the *o*Cb moiety, producing a zwitterion **L­(L** ^**nido**^ **)­P** ^**+**^. We found that the reactivity of **L­(L** ^**nido**^ **)­P** ^**+**^ is significantly diminished compared to **L**_**2**_ **P** ^**+**^, which can provide an interesting avenue for modulating the chemical properties, such as acidity or basicity, of MG centers by modification of *o*Cb-based periphery. Finally, in the reaction with SO_2_ in MeCN, **L**_**2**_ **P** ^**+**^ reacted at both reactive sites, leading to **L­(L** ^**nido**^ **)­P** ^**+**^ **=S**, in which both of the oxygens are stripped of the S center. We continue our research on MG reactive centers and carborane-based substituents.

## Experimental Section

### Safety Statement

No unexpected, unusual, or unusually hazardous materials, conditions, or procedures were encountered in this study.

### General Considerations

All preparations were carried out under an anhydrous N_2_ atmosphere using standard Schlenk and glovebox techniques. All glassware was oven-dried and cooled under vacuum before use. Commercial reagents were purchased from Sigma-Aldrich, Yunali Tech., Stream, or Apollo Scientific and used without further purification unless indicated otherwise. **LH** and **SO**_**2**_ were prepared following the reported procedures.^20,39^ NMR spectra were recorded at room temperature using a Bruker AvanceIII-400 MHz spectrometer. Data for ^1^H NMR are reported as follows: chemical shift (δ ppm), integration, multiplicity (s = singlet, d = doublet, t = triplet, q = quartet, m = multiplet, br = broad, brs = broad singlet), coupling constant (Hz), assignment. High-resolution mass spectrometry was performed with a Waters SYNAPT G2-S.

### Synthesis of LPCl_2_

^*n*^BuLi (1.0 equiv., 1.87 mL, 3.0 mmol, 1.6 M in hexanes) was added to Et_2_O solution (20 mL) of **LH** (663 mg, 3.0 mmol) at 0 °C. The reaction mixture was left in a cold bath to warm slowly to room temperature. The appearance of a light-yellow color indicated the lithiation of **LH**, and the solution was stirred for an additional hour at room temperature. The mixture was cooled again at 0 °C, and PCl_3_ (1.1 equiv. 287 μL, 3.3 mmol) was added. The solution was left in a cold bath to warm to room temperature and then stirred for an additional 12 h. After completion of the reaction, all the volatiles were evaporated under vacuum, and the solid was extracted with CH_2_Cl_2_ (20 mL). A yellow-white solid was obtained upon evaporation. The solid was crystallized by slow evaporation from a chloroform solution, yielding colorless crystals after 2 days. Overall isolated yields of **LPCl**_**2**_ were: 863 mg (89%). ^**1**^ **H NMR** (400 MHz, CDCl_3_); δ = 8.35 (d, *J* = 4 Hz, 1H), 7.89 (t, *J* = 8 Hz, 1H), 7.57 (d, *J* = 8 Hz, 2H), 7.51–7.47 (m, 1H), 3.3–1.7 (br, 10H, carborane_B–H_). ^**13**^ **C­{** ^**1**^ **H} NMR** (100 MHz, CDCl_3_); δ = 150.29 (d, *J* = 6 Hz, Ar–C_Pyridine‑carborane_), 144.13 (Ar–C_H_), 139.53 (Ar–C_H_), 125.33 (Ar–C_H_), 121.71 (Ar–C_H_), 80.73 (d, *J* = 152 Hz, carborane_C–P_), 79.15 (d, *J* = 7 Hz, carborane_C–P_) ppm. ^**31**^ **P NMR** (162 MHz, CDCl_3_); δ = 111.2 ppm. ^**11**^ **B NMR** (128 MHz, CDCl_3_); δ = – 10.1 to – 7.9 (m), – 2.0 (d, *J* = 147 Hz) ppm. **HRMS** (APCI positive mode): Calc. for C_7_ H_14_^10^B_2_^11^B_8_ N P ^35^Cl_1_ = 286.1555; found = 286.1558.

### Synthesis of L’LP

A 100 mL Schlenk flask equipped with a magnetic stir bar was charged with 30 mL of dry benzene and 700 mg of **LPCl**_**2**_ (2.17 mmol). The resulting solution was cooled to 0 °C using an ice bath, and a solution of the lithiated ligand (**LLi**, 2.0 equiv, 4.34 mmol) in benzene was added dropwise over 10 min. After the addition was complete, the reaction mixture was allowed to gradually warm to room temperature and was subsequently stirred for an additional 12 h. After completion of the reaction, all volatiles were removed under vacuum. The resulting solid was extracted with 20 mL CH_2_Cl_2_, producing a brownish-red precipitate upon solvent evaporation. This solid was washed with hexane, yielding an off-white product. The isolated yield of the compound was 704 mg (47%). The off-white solid was dissolved in 10 mL of toluene, and after 4 days at room temperature, slow evaporation afforded colorless crystals. ^**1**^ **H NMR** (400 MHz, CDCl_3_); δ = 8.58 (d, *J* = 16 Hz, 2H), 7.72–7.66 (m, 3H), 7.43 (d, *J* = 8 Hz, 1H), 7.41–7.35 (m, 2H), 6.21 (brs, 1H­(*H*_*d*_)), 5.60 (brs, 1H­(*H*_*b*_)), 5.39 (t, *J* = 6.8, 1H­(*H*_*c*_)), 4.63 (dd, *J* = 12.0, 6.2 Hz, 1H­(*H*_*e*_)), 3.1–1.7 (br, 30H, carborane_B–H_) ppm. ^**13**^ **C­{** ^**1**^ **H} NMR** (100 MHz, CDCl_3_); δ = 151.25 (d, *J* = 4 Hz), 149.92, 149.07, 147.67, 138.36, 137.86, 126.31, 125.96­(*C*_*d*_), 125.13, 124.52, 122.43, 117.46­(*C*_*c*_), 104.84­(*C*_*b*_), 102.61­(*C*_*a*_), 87.45 (d, *J* = 11 Hz­(*C*_*f*_)), 85.47­(*C*_*g*_), 83.16 (d, *J* = 6 Hz­(*C*_*j*_)), 81.93 (d, *J* = 87 Hz­(*C*_*i*_)),78.79 (d, *J* = 4 Hz­(*C*_*h*_)), 71.89 (d, *J* = 46 Hz­(*C*_*k*_)) 56.41 (d, *J* = 19 Hz­(*C*_*e*_)) ppm. ^**31**^ **P NMR** (162 MHz, CDCl_3_); δ = 76.8 ppm. ^**11**^ **B NMR** (128 MHz, CDCl_3_); δ = – 9.1 to – 1.6 (br) ppm. **HRMS** (APPI positive mode): Calc. for C_21_ H_42_^10^B_6_^11^B_24_ N_3_ P= 691.6126; found = 691.6129.

### Synthesis of [**L**_**2**_ **P** ^**+**^]­[OTf]

MeOTf (10 equiv) was added dropwise to a 20 mL benzene solution of **L’LP** (700 mg, 1.01 mmol) under constant stirring. A precipitate gradually formed as the reaction progressed. The mixture was stirred for an additional 12 h at room temperature. The resulting solid was collected by filtration and washed with hexane (3 × 20 mL) to remove excess MeOTf. The obtained off-white solid ([**L**_**2**_ **P** ^**+**^]­[OTf]; 315 mg, 51% yield) exhibits a singlet at 30.78 ppm in acetonitrile (soluble in dilute solution) and was used in subsequent steps without further purification. [**L**_**2**_ **P** ^**+**^]­[OTf] exhibited poor solubility in most organic solvents, which precluded its characterization by ^1^H and ^13^C NMR. Repeated attempts to obtain a crystal suitable for single-crystal X-ray diffraction analysis were not successful. ^**31**^ **P NMR** (162 MHz, CDCl_3_); δ = 30.7 ppm.

### Synthesis of [**L**_**2**_ **P** ^**+**^]­[B­(C_6_F_5_)_4_

]: 15 mL of CH_2_Cl_2_ was added to a mixture of [**L**_**2**_ **P** ^**+**^]­[OTf] (500 mg, 0.80 mmol) and [K]­[ B­(C_6_F_5_)_4_] (637 mg, 0.88 mmol). The reaction mixture was stirred for 12 h, then filtered to remove KOTf. The solvent was evaporated under reduced pressure, and the resulting solid was washed thoroughly with hexane (3 × 10 mL) to afford an off-white solid. This material was subsequently dissolved in a CH_2_Cl_2_/CHCl_3_ mixture (1:1.5) and left to crystallize by slow evaporation at room temperature. After 5 days, colorless crystals of [**L**_**2**_ **P** ^**+**^]­[B­(C_6_F_5_)_4_] were obtained. The SC-XRD analysis of [**L**_**2**_ **P** ^**+**^]­[B­(C_6_F_5_)_4_], revelaved that K­[B­(C_6_F_5_)_4_] was cocrystallized with it. The isolated yield of the crystal was 670 mg (42%). Notably, for the synthesis of [**L**_**2**_ **P** ^**+**^]­[B­(C_6_F_5_)_4_], 1.1 equiv K­[B­(C_6_F_5_)_4_] were used, which after washing with hexane result to off white powder [**L**_**2**_ **P** ^**+**^]­[B­(C_6_F_5_)_4_. Subsequently, the solid [**L**_**2**_ **P** ^**+**^]­[B­(C_6_F_5_)_4_] was dissolved in CHCl_3_ to remove insoluble [K]­[ B­(C_6_F_5_)_4_]. The isolated yield of the crystal was 670 mg (72%). ^**1**^ **H NMR** (400 MHz, CDCl_3_); δ = 8.73 (d, *J* = 8 Hz, 2H (*H*_*d*_)), 8.37 (t, *J* = 8 Hz, 2H (*H*_*e*_)), 7.91 (t, *J* = 8 Hz, 2H (*H*_*c*_)), 7.83 (d, *J* = 8 Hz, 2H (*H*_*b*_)), 3.5–1.5 (br, 20H, carborane_B–H_) ppm. ^**13**^ **C­{** ^**1**^ **H} NMR** (100 MHz, CDCl_3_); δ = 149.43 (d, *J* = 5 Hz, (*C*_*a*_)), 148.09 (d, *J*_C–F_ = 236 Hz, B­(C_6_F_5_)_4_), 146.41 (d, *J* = 8 Hz, (*C*_*d*_)), 145.46­(*C*_*e*_), 138.13 (d, *J*_C–F_ = 248 Hz, B­(C_6_F_5_)_4_), 136.25 (d, *J*_C–F_ = 242 Hz, B­(C_6_F_5_)_4_), 126.64­(*C*_*c*_), 122.76­(*C*_*b*_), 76.43 (d, *J* = 11 Hz, (*C*_*f*_)), 70.77 (d, *J* = 107 Hz, (*C*_*g*_)) ppm. ^**31**^ **P NMR** (162 MHz, CDCl_3_); δ = 36.0 ppm. ^**11**^ **B NMR** (128 MHz, CDCl_3_); δ = – 16.7 (s, B­(C_6_F_5_)_4_), – 10.5 to 0.2 (br) ppm. ^**19**^ **F NMR** (376.5 MHz, CDCl_3_); δ = – 167.2, – 163.1, – 133.2 ppm. **HRMS** (APCI positive mode): Calc. for C_14_ H_28_^10^B_5_^11^B_15_ N_2_ P= 470.4039; found = 470.4040.

### General Procedure for Reaction of [L_2_P^+^]­[B­(C_6_F_5_)_4_] with O–H Bond Containing Molecules

In a J-Young NMR tube, a solution of [**L**_**2**_ **P** ^**+**^]­[B­(C_6_F_5_)_4_] (50 mg, 0.043 mmol) was prepared in 0.5 mL CDCl_3_ and followed by the addition of an excess amount of the respective alcohols or H_2_O. The progress of the reaction was monitored by ^31^P NMR spectroscopy. Notably the reaction with H_2_O proceeded at r.t. while MeOH and PhOH required heating at 85 °C for 24–48 h.

### General Procedure for Reaction of [L_2_P^+^]­[B­(C_6_F_5_)_4_] with N–H Bond Containing Molecules or Synthesis of L­(L^nido^)­P^+^

In a J-Young NMR tube, a solution of [**L**_**2**_ **P** ^**+**^]­[B­(C_6_F_5_)_4_] (50 mg, 0.043 mmol) was prepared in 0.5 mL CDCl_3_ and followed by the addition of an excess amount of the respective amines. The progress of the reaction was monitored by ^31^P NMR spectroscopy. Following the completion of the reaction, the solvent was evaporated, and the residue was washed with hexane (2 × 5 mL). The isolated solid exhibits very low solubility in benzene. The product was isolated by crystallization (21 mg, 84%) from a THF:benzene (1:5) mixture via slow evaporation at room temperature. The SC-XRD of these crystals was measured, determining the molecular structure of the obtained product as a zwitterion (**L­(L** ^**nido**^ **)­P** ^**+**^). Notably the reaction with Et_2_NH proceeded at r.t. while 2,4,6-trimethylaniline, isopropylamine, and aniline required heating at 85 °C for 24 h. **NMR of L­(L** ^**nido**^ **)­P** ^**+**^: ^**1**^ **H NMR** (400 MHz, CD_2_Cl_2_); δ = 8.60 (d, *J* = 4 Hz, 1H), 8.36 (d, *J* = 8 Hz, 1H), 8.06 (m, 2H), 7.68 (d, *J* = 8 Hz, 1H), 7.63 (t, *J* = 6 Hz, 1H), 7.49 (d, *J* = 8 Hz, 1H), 7.44 (t, *J* = 6 Hz, 1H), 2.35 (br, 19H, carborane_B–H_), −2.01 (brs, 1H, B–H_bridge_) ppm. ^**13**^ **C­{** ^**1**^ **H} NMR** (100 MHz, CD_2_Cl_2_); δ = 166.74 (d, *J* = 5 Hz), 148.95 (d, *J* = 6), 144.91 (Ar_C–H_), 143.90 (Ar_C–H_), 142.59 (d, *J* = 15 Hz), 141.49 (Ar_C–H_), 125.43 (Ar_C–H_), 121.89 (Ar_C–H_), 121.07 (Ar_C–H_), 120.37 (d, *J* = 3 Hz), 77.45 (d, *J* = 8 Hz, carborane_C‑Pyridine_), 75.40 (d, *J* = 106 Hz, carborane_C–P_) ppm. ^**31**^ **P NMR** (162 MHz, CD_2_Cl_2_); δ = 71.0 ppm. ^**11**^ **B NMR** (128 MHz, CD_2_Cl_2_); δ = – 32.2 (d, *J* = 147 Hz), – 29.7 (d, *J* = 129 Hz), – 21.8 (d, *J* = 142 Hz), – 19.4 (d, *J* = 145 Hz), – 15.7 (d, *J* = 151 Hz), – 9.6 (d, *J* = 124 Hz), – 7.7 (br), – 3.9 (br) ppm. **HRMS** (APCI positive mode): Calc. for C_14_ H_26_^10^B_4_^11^B_15_ N_2_ P = 458.3755; found = 458.3752.

### General Procedure for Reaction of [L_2_P^+^]­[B­(C_6_F_5_)_4_] with SO_2_ or Synthesis of L­(L^nido^)­P^+^=S

A solution of [**L**_**2**_ **P** ^**+**^]­[B­(C_6_F_5_)_4_] (50 mg, 0.043 mmol) was prepared in 0.7 mL of CH_3_CN and transferred into a J-Young NMR tube under an inert atmosphere. The tube was degassed two times using freeze–pump–thaw cycles. While the tube was immersed in liquid nitrogen, SO_2_ was added. The sealed tube was then placed in an oil bath and heated at 100 °C for 48 h.The reaction progress was monitored by ^31^P NMR spectroscopy. After 3 h, in addition to the signal corresponding to **L**_**2**_ **P** ^**+**^ (at 31 ppm in CD_3_CN), a new signal appeared at δ = – 1.9 ppm, possibly assigned to an adduct formed between SO_2_ and the boron center of the *o*-Cb cluster. After 6 h, another signal appeared at 26 ppm, which, after 48 h, was the only signal in ^31^P NMR. After 5 days, crystals were formed from the reaction mixture. The SC-XRD of these crystals showed the molecular structure as a *nido*-zwitterionic phosphonium sulfide (**L­(L** ^**nido**^ **)­P** ^**+**^ **=S**). **NMR of L­(L** ^**nido**^ **)­P** ^**+**^ **=S**: ^**1**^ **H NMR** (400 MHz, DMSO-*d*_6_); δ = 8.99 (d, *J* = 4 Hz, 1H), 8.81 (d, *J* = 4 Hz, 1H), 8.08–8.0 (m, 2H), 7.51 (t, *J* = 8 Hz, 2H), 7.31 (d, *J* = 8.0 Hz, 1H), 7.24 (d, *J* = 8 Hz, 1H), 1.23 (br, 19H, carborane_B–H_), – 2.51 (brs, 1H, B–H_bridge_) ppm. ^**13**^ **C­{** ^**1**^ **H} NMR** (100 MHz, DMSO-*d*_6_); δ = 157.18 (d, *J* = 4 Hz), 154.16 (d, *J* = 6), 143.97 (Ar_C–H_), 143.62 (Ar_C–H_), 142.96 (Ar_C–H_), 142.65 (d, *J* = 10) (Ar_C–H_), 120.96 (Ar_C–H_), 120.19 (Ar_C–H_), 119.46 (Ar_C–H_), 117.96 (Ar_C–H_), 117.45 (Ar_C–H_), 63.70 (d, *J* = 13 Hz carborane_C–P_), 60.68 (d, *J* = 10 Hz carborane_C–P_) ppm. ^**31**^ **P NMR** (162 MHz, DMSO-*d*_6_); δ = 24.1 ppm. ^**11**^ **B NMR** (128 MHz, DMSO-*d*_6_); δ = −32.3 to 20.5 (br) ppm. **HRMS** for [M – B^+^ ] (APCI negative mode): Calc. for C_14_ H_28_^10^B_3_^11^B_15_ N_2_ P S = 482.3495; found = 482.3493.

### X-ray Crystallography Details

The crystals for all the compounds herein were mounted on a cryoloop with Paratone oil, and all data were collected at 100(2) or 110(2) K (for L’LP). Single crystal X-ray diffraction data for **LPCl**_**2**_, **L**_**2**_ **P** ^**+**^, **L­(L** ^**nido**^ **)­P** ^**+**^, **LH**_**2**_^**+**^ were collected on a Rigaku Oxford Diffraction - XtaLAB Synergy-S diffractometer operated with monochromated MoKα (α = 0.71073 Å) or CuKα (for L_2_P^+^, LH_2_^+^; α = 1.54184 Å) X-ray source, respectively. The data collection, cell refinement, data reduction, and analytical method absorption correction were performed using CrysAlisPro. Single crystal X-ray diffraction data for **L’LP** were collected on a Bruker D8 KAPPA APEXDuo diffractometer equipped with an APEX II CCD detector using a TRIUMPH monochromator with a MoKα X-ray source (α = 0.71073 Å). Unit cell determination, refinement, and data collection were done using the Bruker APEX-III suite,^40^ data reduction and integration were performed using SAINT v8.34A (Bruker, 2013)^40^ and absorption corrections and scaling were done using SADABS-2014/5 (Bruker,2014/5).^40^ All the crystal structures were solved through OLEX2 ^41^ package using SHELXT^41^ and the structures were refined using SHELXL.^41^ All non-hydrogen atoms were refined anisotropically. All the figures were generated using Mercury 3.0.

### DFT Computational Details

DFT calculations were performed using Gaussian 09.2.^42^ Geometry optimization of all the molecules was carried out using the B3LYP-D3­(BJ)/6–311++G­(d,p)^23,24^ and BP86-D3­(BJ)/def2-TZVP^45^ implemented in the Gaussian 09 software. Conductor-like polarizable continuum model (CPCM)^46^ was used to account for the effect of chloroform. Thermal energy corrections were extracted from the results of frequency analysis performed at the same level of theory. Frequency analysis of all the molecules and intermediates contained no imaginary frequency, showing that these are energy minima. AIM analysis was performed using Multiwfn.^47^ The computational pictures were generated using Avogadro.^48^

## Supplementary Material



## Notes

NMR spectra (PDF)

## References

[1] a Crabtree, R. H. The Organometallic Chemistry of the Transition Metals, 6th ed.; Wiley-VCH: Hoboken, NJ, 2014.

[2] Power P. P. (2010). Main-Group Elements as Transition
Metals. Nature.

[3] Chu T., Nikonov G. I. (2018). Oxidative Addition
and Reductive
Elimination at Main-Group Element Centers. Chem.
Rev..

[4] Martin D., Soleilhavoup M., Bertrand G. (2011). Stable Singlet Carbenes
as Mimics for Transition Metal Centers. Chem.
Sci..

[5] Légaré M.-A., Bélanger-Chabot G., Dewhurst R. D., Welz E., Krummenacher I., Engels B., Braunschweig H. (2018). Nitrogen fixation and reduction at
boron. Science.

[6] Hicks J., Vasko P., Goicoechea J. M., Aldridge S. (2018). Synthesis, Structure and Reaction Chemistry of a Nucleophilic
Aluminyl Anion. Nature.

[7] Stephan D. W. (2015). Frustrated
Lewis Pairs: From Concept to Catalysis. Acc.
Chem. Res..

[8] Hannah T. J., Chitnis S. S. (2024). Ligand-Enforced Geometric Constraints
and Associated Reactivity in p-Block Compounds. Chem. Soc. Rev..

[9] Bonfante S., Lorber C., Lynam J. M., Simonneau A., Slattery J. M. (2024). Metallomimetic C–F Activation
Catalysis by Simple
Phosphines. J. Am. Chem. Soc..

[10] Lin Y.-C., Hatzakis E., McCarthy S. M., Reichl K. D., Lai T.-Y., Yennawar H. P., Radosevich A. T. (2017). P–N
Cooperative Borane Activation and Catalytic Hydroboration by a Distorted
Phosphorous Triamide Platform. J. Am. Chem.
Soc..

[11] Bawari D., Toami D., Dobrovetsky R. (2025). Advancing
Metallomimetic Catalysis through Structural Constraints of Cationic
P^III^ Species. Chem. Commun..

[12] Volodarsky S., Bawari D., Dobrovetsky R. (2022). Dual Reactivity
of a Geometrically Constrained Phosphenium Cation. Angew. Chem., Int. Ed..

[13] Roth D., Radosevich A. T., Greb L. (2023). Reversible Oxidative Addition of Nonactivated C–H Bonds to
Structurally Constrained Phosphenium Ions. J.
Am. Chem. Soc..

[14] Liu Y., Dong W., Li Z. H., Wang H. (2021). Methane Activation
by a Borenium Complex. Chem..

[15] Akram M. O., Tidwell J. R., Dutton J. L., Martin C. D. (2022). Tris­(ortho-Carboranyl)­Borane: An Isolable, Halogen-Free,
Lewis Superacid. Angew. Chem., Int. Ed..

[16] Zhang C., Wang J., Su W., Lin Z., Ye Q. (2021). Synthesis, Characterization, and Density Functional
Theory Studies of Three-Dimensional Inorganic Analogues of 9,10-Diboraanthracene—A
New Class of Lewis Superacids. J. Am. Chem.
Soc..

[17] Humphries A. L., Tellier G. A., Smith M. D., Chianese A. R., Peryshkov D. V. (2024). N–H Bond Activation of Ammonia
by a Redox-Active Carboranyl Diphosphine. J.
Am. Chem. Soc..

[18] Sceney M., White K. F., Dutton J. L. (2025). Ortho-Carborane-Supported
Hypervalent Iodine­(III) Reagents: A Scaffold for Enhanced Oxidative
Capability. J. Am. Chem. Soc..

[19] Bawari D., Toami D., Jaiswal K., Dobrovetsky R. (2024). Hydrogen Splitting
at a Single Phosphorus Center and Its Use for Hydrogenation. Nat. Chem..

[20] Anderson K. P., Mills H. A., Mao C., Kirlikovali K. O., Axtell J. C., Rheingold A. L., Spokoyny A. M. (2019). Improved Synthesis
of Icosahedral Carboranes Containing Exopolyhedral B C and C C Bonds. Tetrahedron.

[21] Deposition Numbers 2512627; 2512628; 2512629; 2512630; 2512631; 2512661 contain the supplementary crystallographic data for this paper. These data are provided free of charge by the joint Cambridge Crystallographic Data Center and Fachinformationszentrum Karlsruhe Access Structures service.

[22] Lamande′ L., Munoz A. (1991). Stabilisation de cations
oxophosphenium par liaisons datives P←N. Tetrahedron Lett..

[23] Becke A. D. (1993). Density-Functional Thermochemistry.
III. The Role of Exact Exchange. J. Chem. Phys..

[24] Krishnan R., Binkley J. S., Seeger R., Pople J. A. (1980). Self-Consistent Molecular Orbital Methods. XX. A Basis
Set for Correlated Wave Functions. J. Chem.
Phys..

[25] b Glendening, E. D. ; Badenhoop, J. K. ; Reed, A. E. ; Carpenter, J. E. ; Bohmann, J. A. ; Morales, C. M. ; Weinhold, F. NBO 6.0; Theoretical Chemistry Institute, University of Wisconsin: Madison, WI, 2013.

[26] Bader, R. F. W. Atoms in Molecules: A Quantum Theory; Clarendon Press: Oxford, 1994.

[27] Li C., Wang Q., Zhang J.-Q., Ye J., Xie J., Xu Q., Han L.-B. (2019). Water Determines
the Products: An Unexpected Bro̷nsted Acid-Catalyzed PO–R
Cleavage of P­(III) Esters Selectively Producing P­(O)–H and
P­(O)–R Compounds. Green Chem..

[28] Inoue A., Shinokubo H., Oshima K. (2003). Oxidative Heck-Type Reaction Involving Cleavage of
a Carbon–Phosphorus Bond of Arylphosphonic Acids. J. Am. Chem. Soc..

[29] Hudson R. F., Moss G. (1962). The mechanism of hydrolysis
of phosphorochloridates and related compounds. Part IV. Phosphoryl
chloride. J. Chem. Soc..

[30] Gopalakrishnan J. (2009). Aminophosphines:
Their Chemistry and Role as Ligands and Synthons. Appl. Organometal. Chem..

[31] Oberdorfer R., Nieger M., Niecke E. (1994). 1,3-Diaza-2λ2-phosphonia-4λ4-gallatacyclobutane. Chem. Ber..

[32] Willans C. E., Kilner C. A., Fox M. A. (2010). Deboronation
and Deprotonation of ortho-Carborane with N-Heterocyclic Carbenes. Chem.—Eur. J..

[33] Taoda Y., Sawabe T., Endo Y., Yamaguchi K., Fujii S., Kagechika H. (2008). Identification of an Intermediate
in the Deboronation of Ortho-Carborane: An Adduct of Ortho-Carborane
with Two Nucleophiles on One Boron Atom. Chem.
Commun..

[34] Grimes, R. N. Carboranes, 2nd ed.; Academic Press: London, 2011.

[35] Han Z.-Z., Zhang C.-P. (2021). Phenyl­(Trifluoroethyl)­Iodonium-Triflate-Initiated
Ring-Opening Polymerization of Tetrahydrofuran. Tetrahedron Lett..

[36] Pérez M., Hounjet L. J., Caputo C. B., Dobrovetsky R., Stephan D. W. (2013). Olefin Isomerization and Hydrosilylation
Catalysis
by Lewis Acidic Organofluorophosphonium Salts. J. Am. Chem. Soc..

[37] Rawat S., Bhandari M., Prashanth B., Singh S. (2020). Three Coordinated Organoaluminum
Cation for Rapid and Selective Cyanosilylation of Carbonyls under
Solvent-Free Conditions. ChemCatChem..

[38] Hu C., Pink M., Goicoechea J. M. (2025). Isolation
of a Phosphinidene Sulfide and Selenide. Chem.
Sci..

[39] Adenot A., von Wolff N., Lefevre ` G., Berthet J.-C., Thuéry P., Cantat T. (2019). Activation of SO2 by N/Si+ and N/B Frustrated Lewis
Pairs: Experimental and Theoretical Comparison with CO2 Activation. Chem.—Eur. J..

[40] a Bruker (2019). APEX ΙΙΙ. Bruker AXS Inc.: Madison, Wisconsin, USA.

[41] Dolomanov O. V., Bourhis L. J., Gildea R. J., Howard J. A. K., Puschmann H. (2009). OLEX2: A Complete Structure Solution,
Refinement and Analysis Program. J. Appl. Crystallogr..

[42] Gaussian 09, Revision D.01, M. J., Frisch , G. W., Trucks , H. B., Schlegel , G. E., Scuseria , M. A., Robb , J. R., Cheeseman , G., Scalmani , V., Barone , B., Mennucci , G. A., Petersson , H., Nakatsuji , M., Caricato , X., Li , H. P., Hratchian , A. F., Izmaylov , J., Bloino , G., Zheng , J. L., Sonnengerg , M., Hada , M., Ehara , K., Toyota , R., Fukuda , J., Hasegawa , M., Ishida , T., Nakajima , Y., Honda , O., Kitao , H., Nakai , T., Vreven , J. A., Montgomery, Jr. , J. E., Peralta , F., Ogliaro , M., Bearpark , J. J., Heyd , E., Brothers , K. N., Kudin , V. N., Staroverov , T., Keith , R., Kobayashi , J., Normand , K., Raghavachari , A., Rendell , J. C., Burant , S. S., Iyengar , J., Tomasi , M., Cossi , N., Rega , N. J., Millam , M., Klene , J. E., Knox , J. B., Cross , V., Bakken , C., Adamo , J., Jaramillo , R., Gomperts , R. E., Stratmann , O., Yazyev , A. J., Austin , R., Cammi , C., Pomelli , J. W., Ochterski , R. L., Martin , K., Morokuma , V. G., Zakrzewski , G. A., Voth , P., Salvador , J. J., Dannenberg , S., Dapprich , A. D., Daniels , Ö., Farkas , J. B., Foresman , J. V., Ortiz , J., Cioslowski ; D. J., Fox , Gaussian, Inc.: Wallingford CT, 2010.

[45] Becke A. D. (1988). Density-functional exchange-energy
approximation with correct asymptotic behavior. Phys. Rev. A.

[46] Barone V., Cossi M. (1998). Quantum Calculation
of Molecular Energies and Energy Gradients in Solution by a Conductor
Solvent Model. J. Phys. Chem. A.

[47] Lu T., Chen F. (2012). Multiwfn: A Multifunctional
Wavefunction
Analyzer. J. Comput. Chem..

[48] Hanwell M. D., Curtis D. E., Lonie D. C., Vandermeersch T., Zurek E., Hutchison G. R. (2012). Avogadro:
An advanced semantic chemical
editor, visualization, and analysis platform. J. Cheminform..

