# Supplementary material for: Bis(pyridino‑o‑carboranyl)phosphenium Cation: A Platform for Dual-Site Reactivity
Source: Inorg Chem. 2026 Jun 29;65(27):15640–50. doi: 10.1021/acs.inorgchem.6c01373 (PMC13370872; doi:10.1021/acs.inorgchem.6c01373)
Supplement: Supplementary file 1 [file ic6c01373_si_001.pdf]

Bis(pyridino-o-carboranyl)phosphenium Cation: a Platform for Dual-site Reactivity

Shiv Kumar,[a] Deependra Bawari,* [a,b] and Roman Dobrovetsky*[a]

[a]School of Chemistry, Raymond and Beverly Sackler Faculty of Exact Sciences, Tel Aviv University, Tel Aviv 69978, Israel

[b]Department of Chemistry, School of Basic Sciences, Central University of Haryana, Mahendergarh-123031, Haryana, India

Email: rdobrove@tau.ac.il

Table of Contents

1. NMR data and X-Ray crystallographic tables ……………………………….…S3

2. DFT Computations………………………………………………….....………S51

3. References………………………………………………………….….……..S125

1. Experimental procedures and NMR:

1.1 NMR of $LPCl_2$:

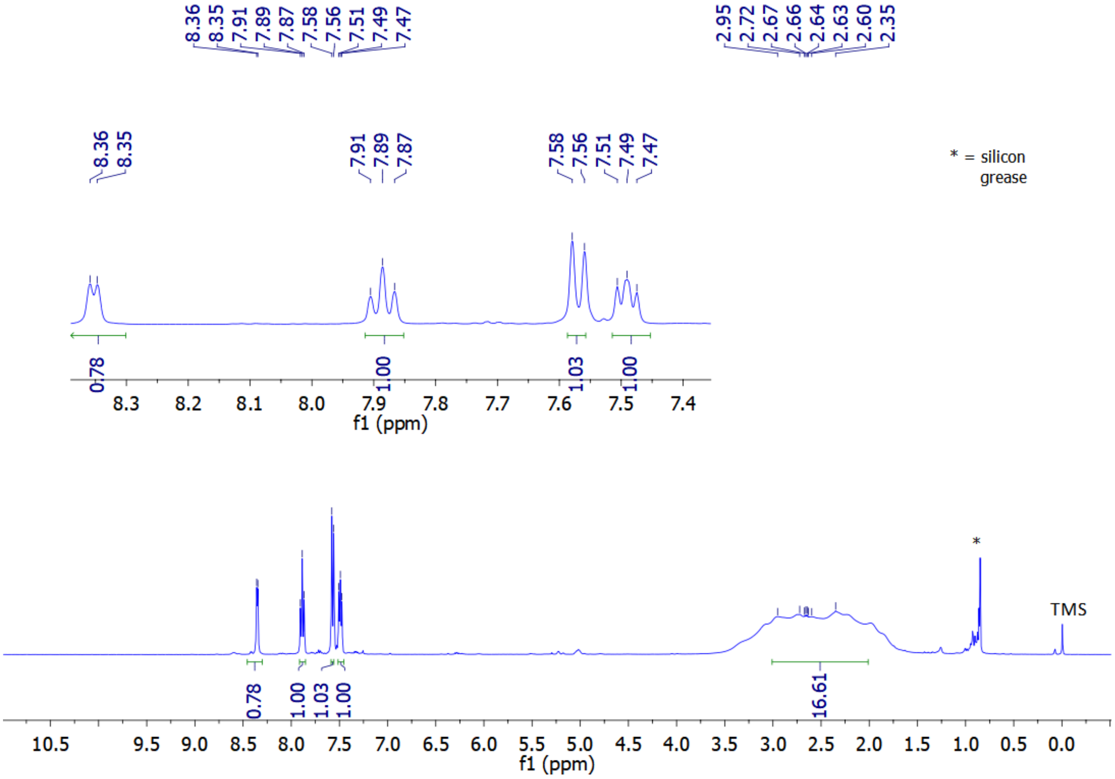

Figure S1. ^{1}H NMR spectrum (400 MHz, $CDCl_3$) of **$LPCl_2$**.

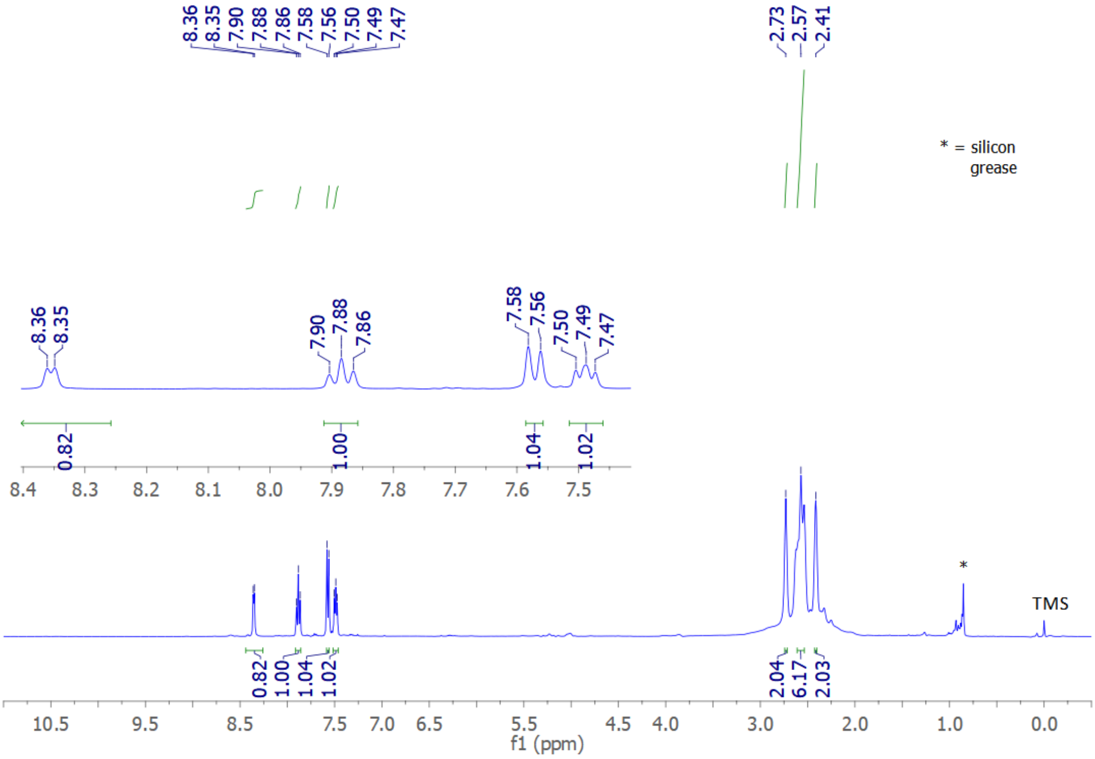

Figure S2. $^{1}H\{^{11}B\}$ NMR spectrum (400 MHz, $CDCl_3$) of **$LPCl_2$**.

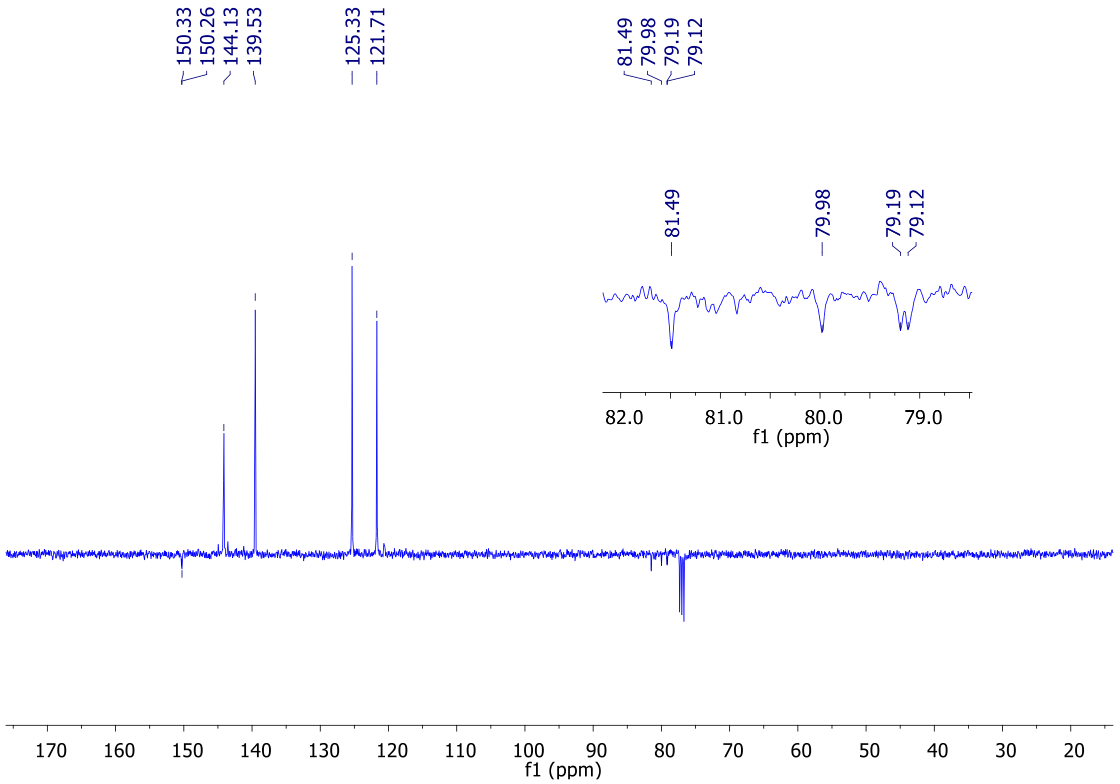

Figure S3. $^{13}C\{^{1}H\}$-JMOD NMR spectrum (100 MHz, $CDCl_3$) of $\mathbf{LPCl_2}$.

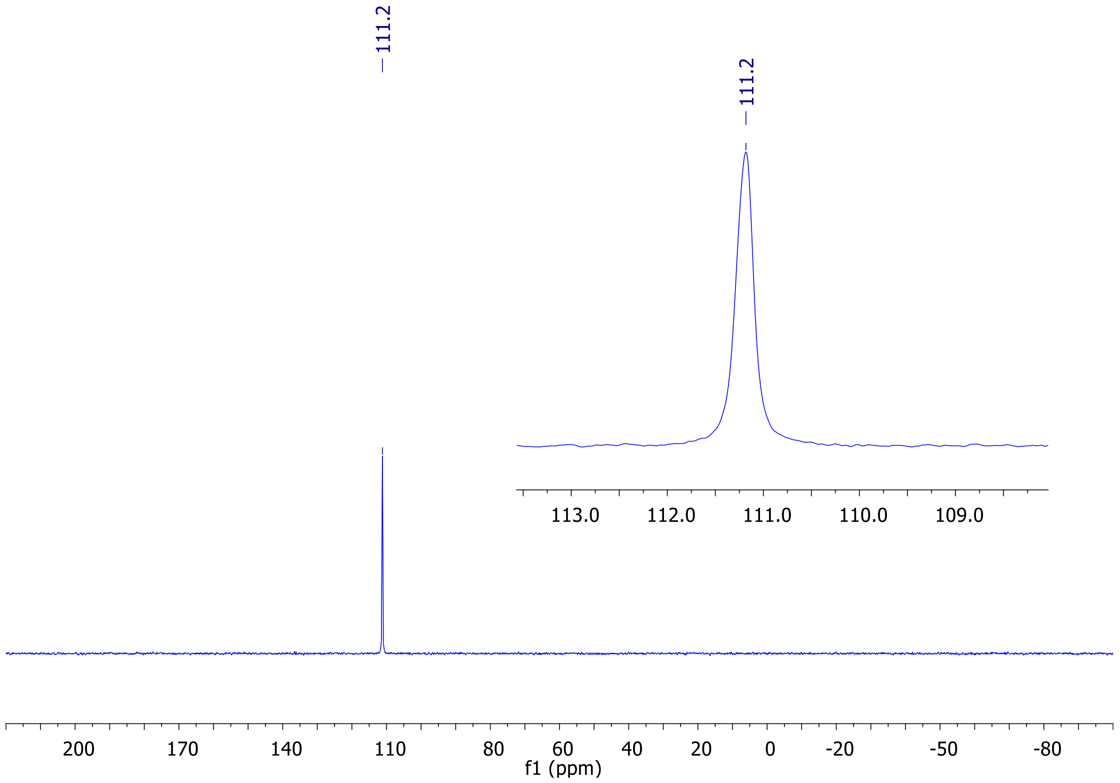

Figure S4. ^{31}P NMR spectrum (162 MHz, $CDCl_3$) of $\mathbf{LPCl_2}$.

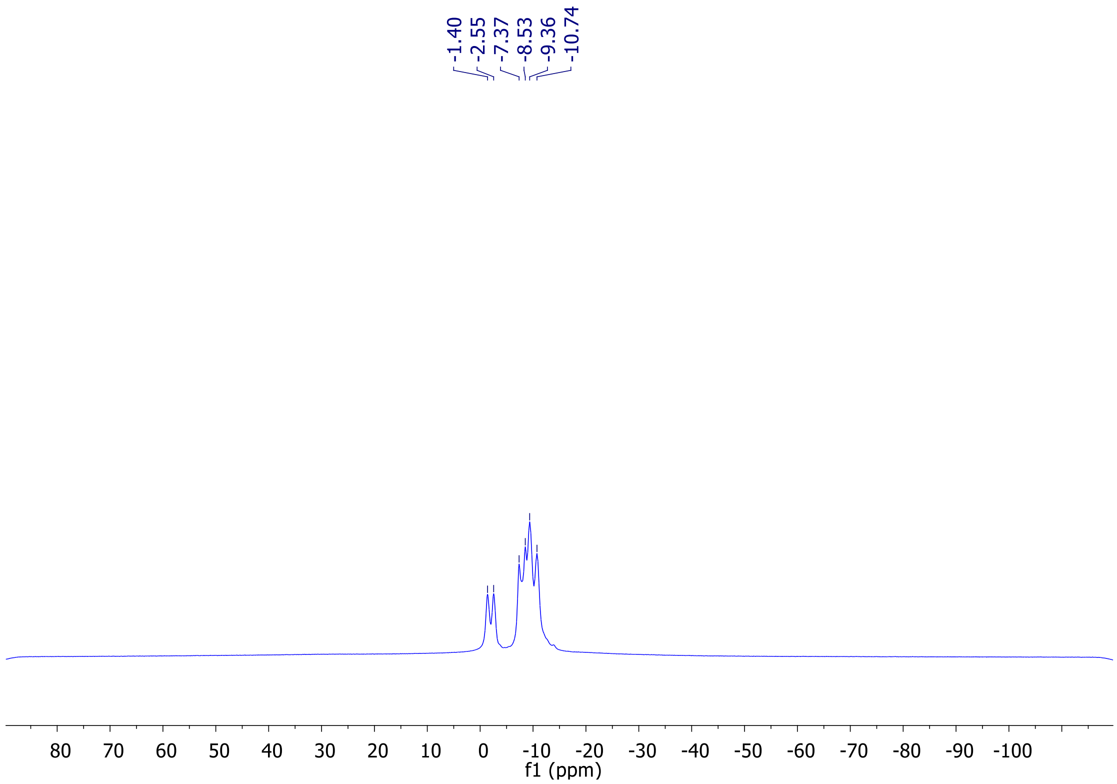

Figure S5. ^{11}B NMR spectrum (128 MHz, $CDCl_3$) of **$LPCl_2$**.

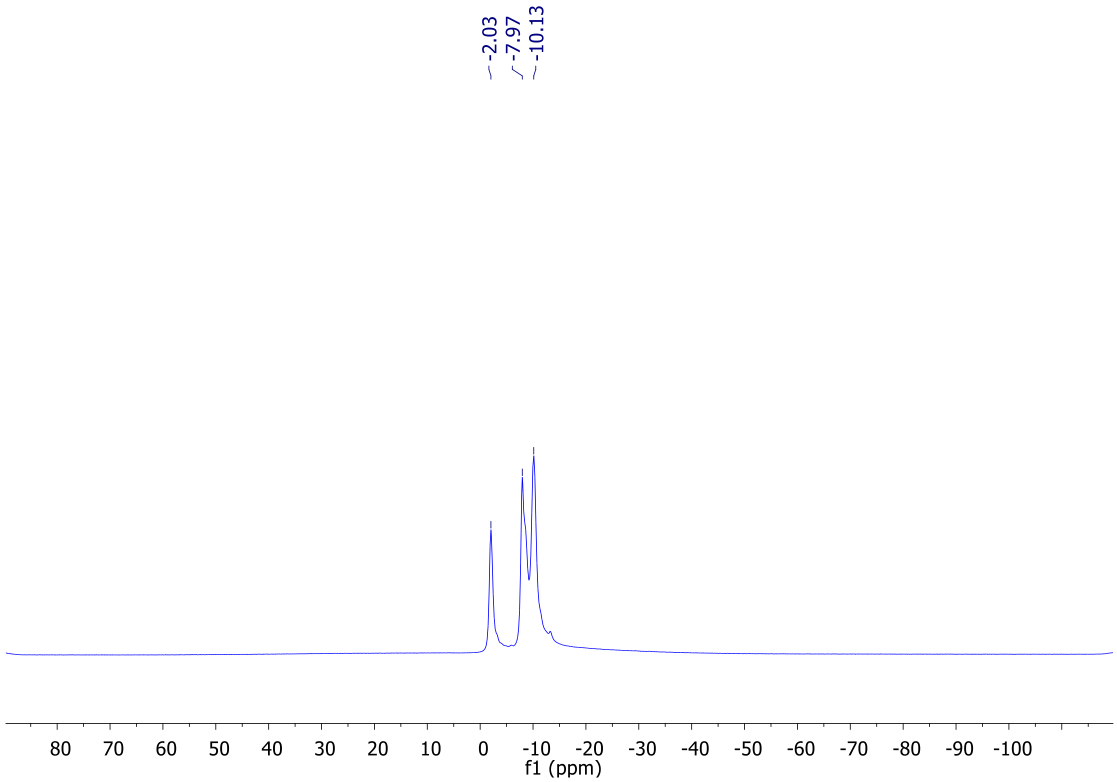

Figure S6. ^{11}B{^{1}H} NMR spectrum (128 MHz, $CDCl_3$) of **$LPCl_2$**.

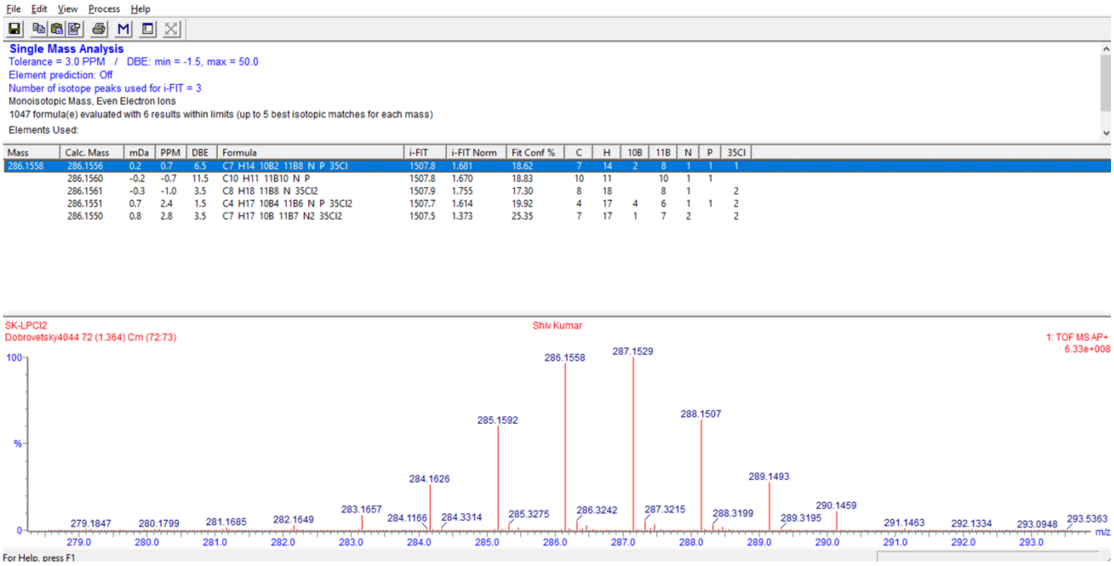

File Edit View Process Help

Single Mass Analysis
Tolerance = 3.0 PPM / DBE: min = -1.5, max = 50.0
Element prediction: Off
Number of isotope peaks used for i-FIT = 3
Monoisotopic Mass, Even Electron Ions
1047 formula(e) evaluated with 6 results within limits (up to 5 best isotopic matches for each mass)
Elements Used:

Mass	Calc. Mass	mDa	PPM	DBE	Formula	i-FIT	i-FIT Norm	Fit Conf %	C	H	10B	11B	N	P	35Cl
286.1558	286.1556	0.2	0.7	6.5	C7 H14 10B2 11B8 N P 35Cl	1507.8	1.681	18.62	7	14	2	8	1	1	1
	286.1560	-0.2	-0.7	11.5	C10 H11 11B10 N P	1507.8	1.670	18.83	10	11		10	1	1	
	286.1561	-0.3	-1.0	3.5	C8 H18 11B8 N 35Cl2	1507.9	1.755	17.30	8	18		8	1		2
	286.1551	0.7	2.4	1.5	C4 H17 10B4 11B6 N P 35Cl2	1507.7	1.614	19.92	4	17	4	6	1	1	2
	286.1550	0.8	2.8	3.5	C7 H17 10B 11B7 N2 35Cl2	1507.5	1.373	25.35	7	17	1	7	2		2

Figure S7. HRMS spectrum of **$LPCl_2$**.

1.2 NMR of L'LP:

NMR values are given below to specify the peaks corresponding to respective atoms in the structure.

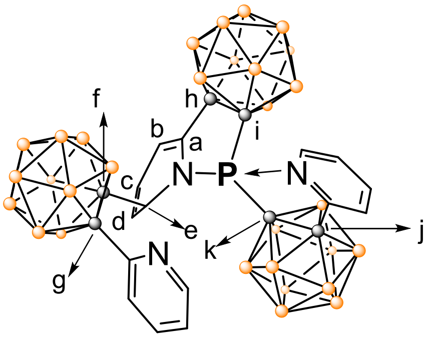

(L'LP)

^{1}H NMR (400 MHz, $CDCl_3$); δ = 8.58 (d, J = 16.8 Hz, 2H), 7.72-7.66 (m, 3H), 7.43 (d, J = 7.9 Hz, 1H), 7.41-7.35 (m, 2H), 6.21 (brs, 1H(H_d)), 5.60 (brs, 1H(H_b)), 5.39 (t, J = 6.8 Hz, 1H(H_c)), 4.63 (dd, J = 12.0, 6.2 Hz, 1H(H_e)), 3.1-1.7 (br, 30H, carborane$_{B\text{-}H}$) ppm.

^{13}C{^{1}H} NMR (100 MHz, $CDCl_3$); δ = 151.25 (d, J = 4.0f Hz), 149.92, 149.07, 147.67, 138.36, 137.86, 126.31, 125.96(C_d), 125.13, 124.52, 122.43, 117.46(C_c), 104.84(C_b), 102.61(C_a), 87.45 (d, J = 11.0 Hz, $^3J_{P\text{–}C}$ (C_f)), 85.47(C_g), 83.16 (d, J = 6.0 Hz, $^2J_{P\text{–}C}$

(C_j)), 81.93 (d, J = 174.0 Hz, $^{1}J_{P\text{–}C}$ (C_i)), 78.79 (d, J = 4.0 Hz, $^{2}J_{P\text{–}C}$ (C_h)), 71.89 (d, J = 46.0 Hz, $^{1}J_{P\text{–}C}$ (C_k)) 56.41 (d, J = 19 Hz, $^{2}J_{P\text{–}C}$ (C_e)) ppm**.**

^{31}P NMR (162 MHz, $CDCl_3$); δ = 76.87 ppm.

^{11}B NMR (128 MHz, $CDCl_3$); δ = –9.16 to –1.68 (br) ppm.

HRMS (APPI positive mode): Calc. for C_{21} H_{42} $^{10}B_6$ $^{11}B_{24}$ N_3 P= 691.6126; found = 691.6129.

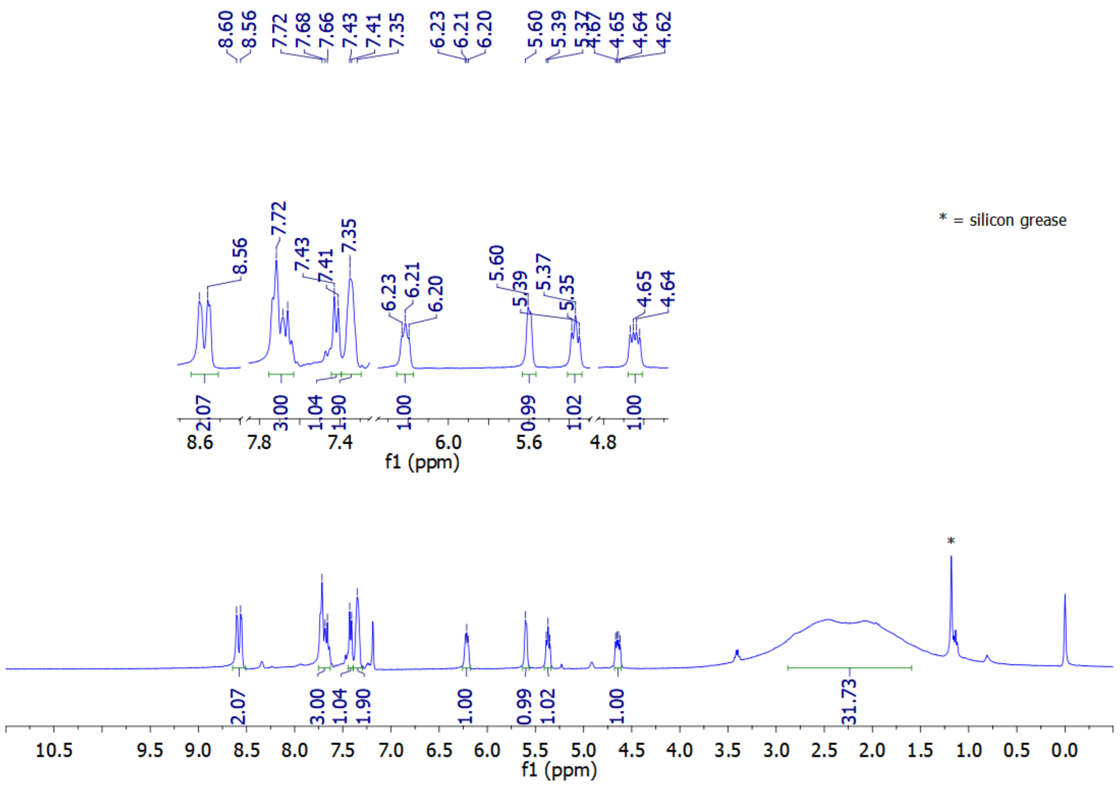

Figure S8. ^{1}H NMR spectrum (400 MHz, $CDCl_3$) of **L'LP**.

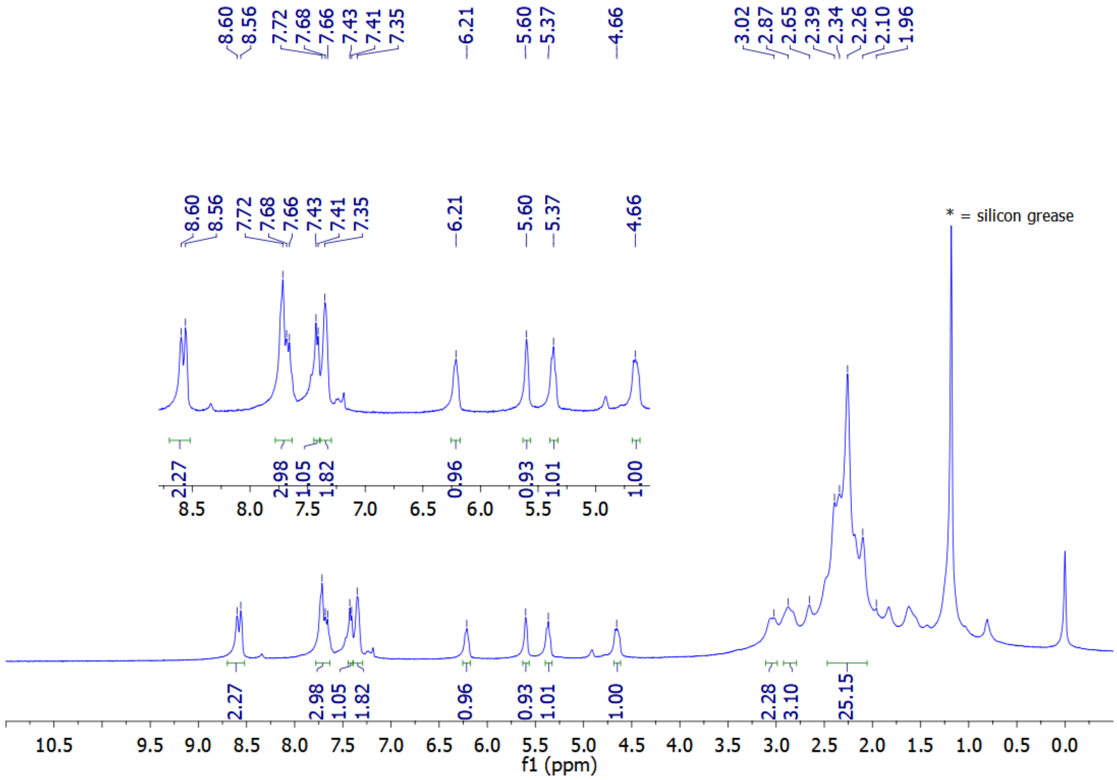

Figure S9. $^{1}H\{^{11}B\}$ NMR spectrum (400 MHz, $CDCl_3$) of **L'LP**.

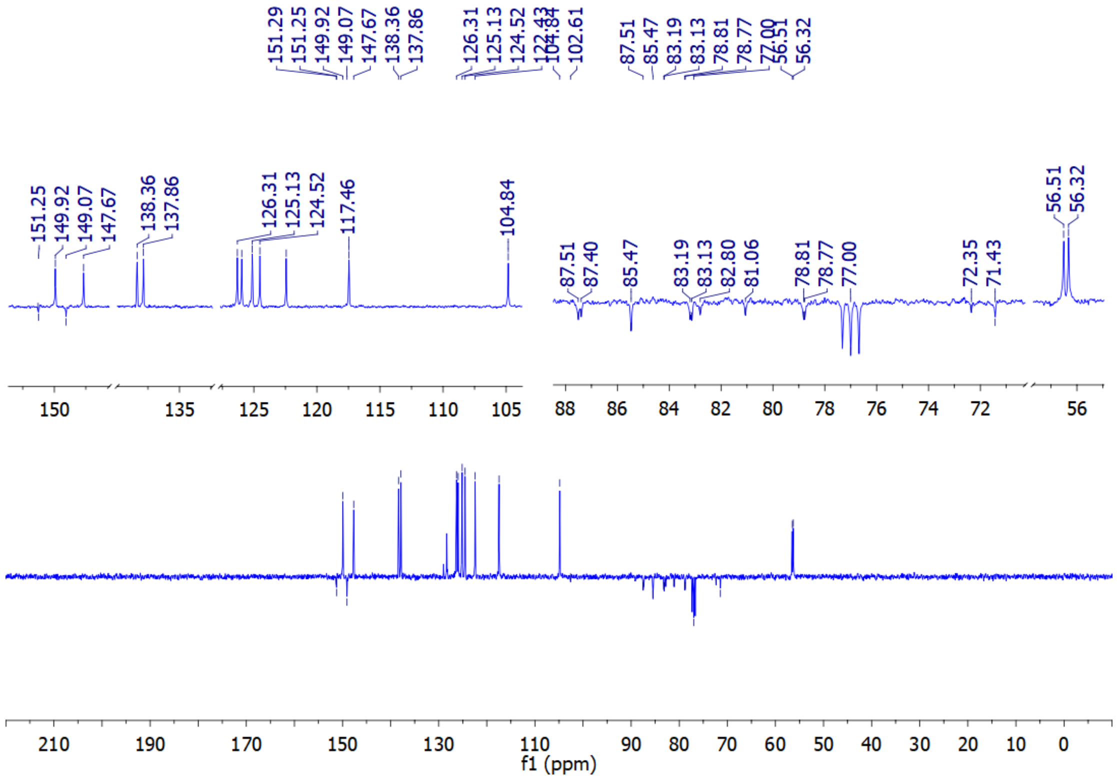

Figure S10. $^{13}C\{^{1}H\}$-JMOD NMR spectrum (100 MHz, $CDCl_3$) of **L'LP**.

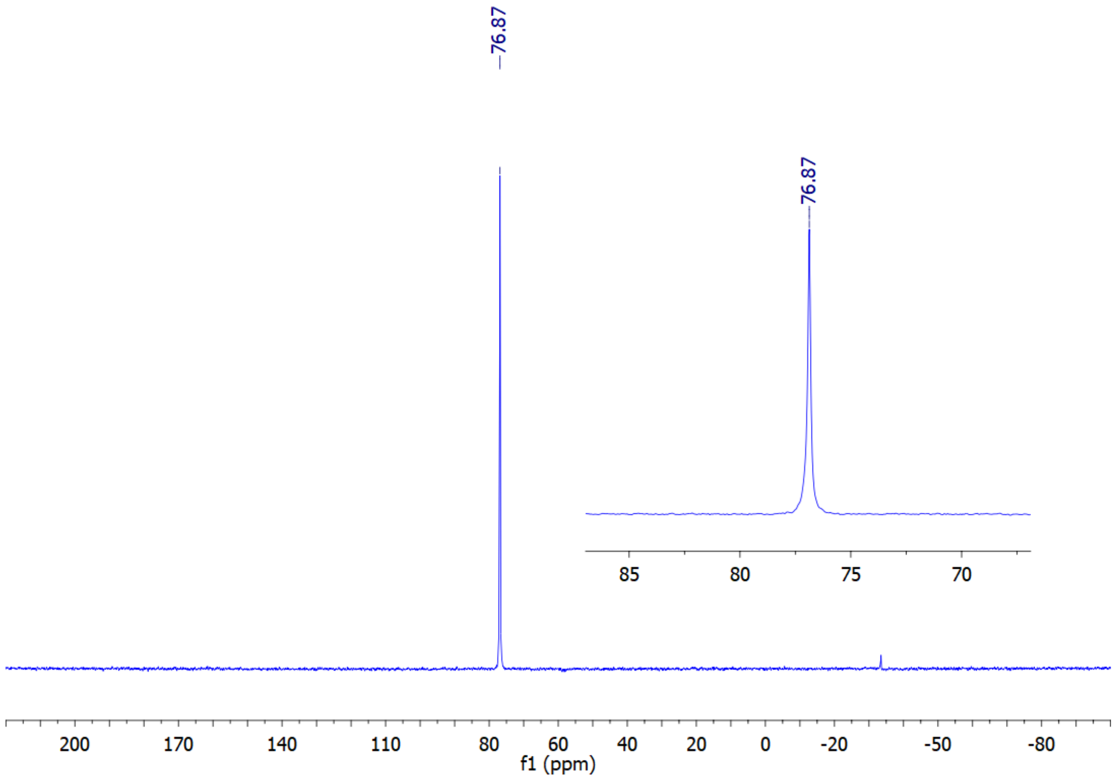

Figure S11. ^{31}P NMR spectrum (162 MHz, $CDCl_3$) of **L'LP**.

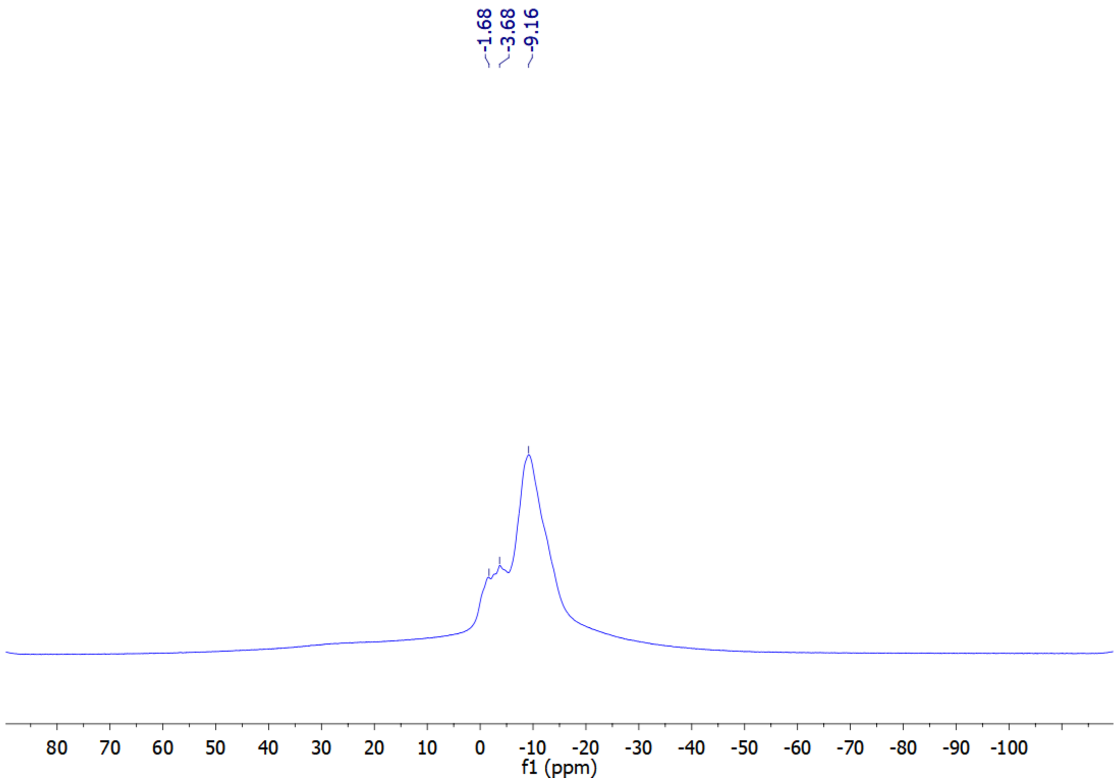

Figure S12. ^{11}B NMR spectrum (128 MHz, $CDCl_3$) of **L'LP**.

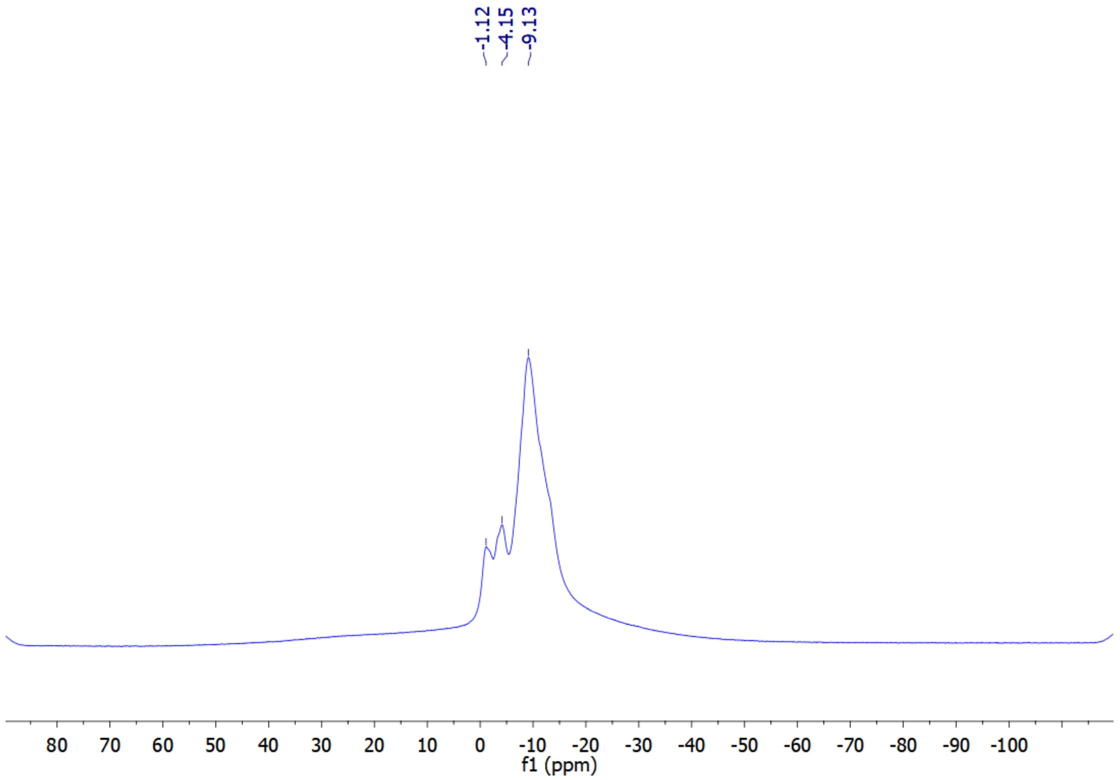

Figure S13. $^{11}B\{^1H\}$ NMR spectrum (128 MHz, $CDCl_3$) of **L'LP**.

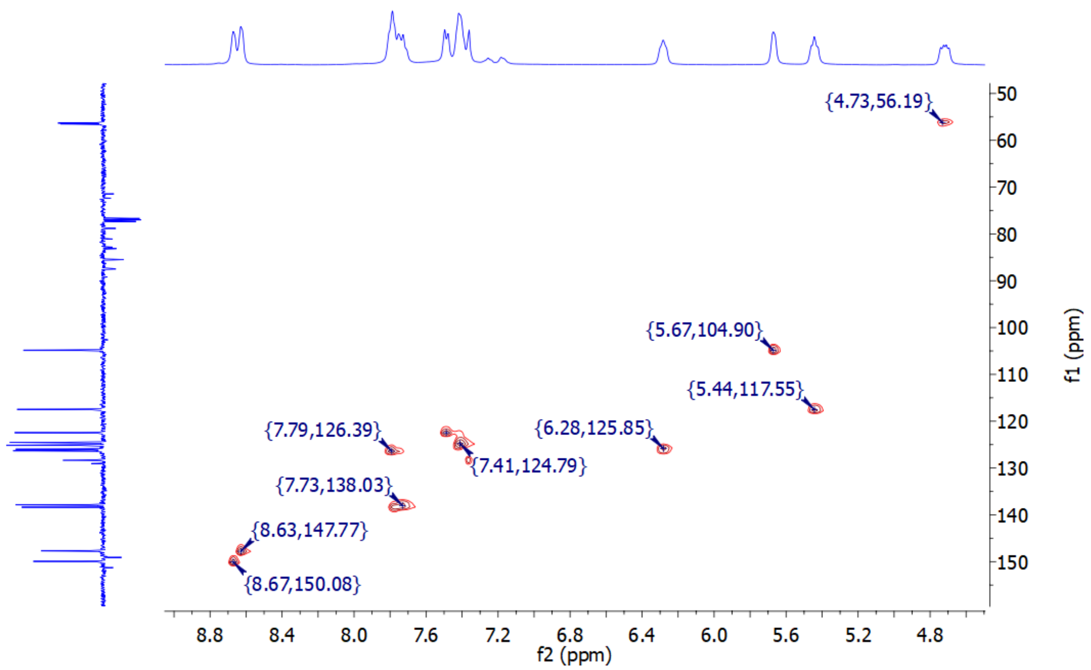

Figure S14. ^1H-^{13}C HSQC NMR spectrum ($CDCl_3$) of **L'LP**.

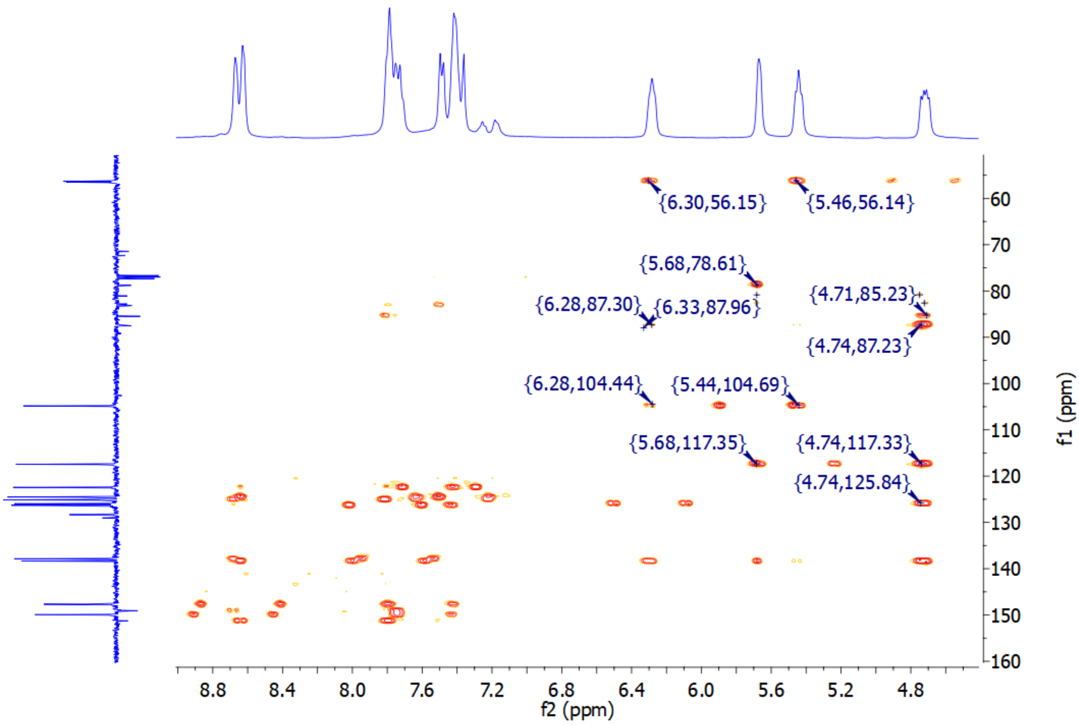

Figure S15. ^{1}H-^{13}C HMBC NMR spectrum ($CDCl_3$) of **L'LP**.

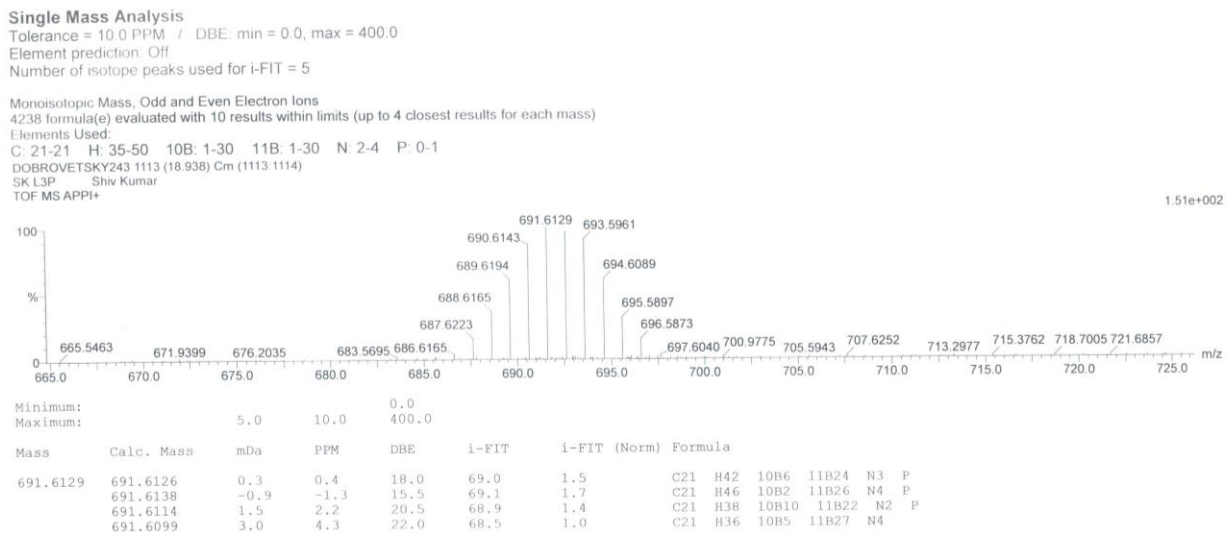

Figure S16. HRMS spectrum of **L'LP**.

1.3 Synthesis of [L_2P^+][OTf]:

[$\mathbf{L_2P^+}$][OTf] exhibited poor solubility in most organic solvents, which precluded its characterization by ^{1}H and ^{13}C NMR. Repeated attempts to obtain a crystal suitable for single-crystal X-ray diffraction analysis were not successful.

The residual hexane was removed under reduced pressure, and the resulting solid was washed with cold pentane to eliminate residual impurities. Subsequent drying afforded a white solid,

which was characterized without further purification. The ^{1}H and ^{13}C NMR spectra were consistent with the reported data for **LMe**.[1]

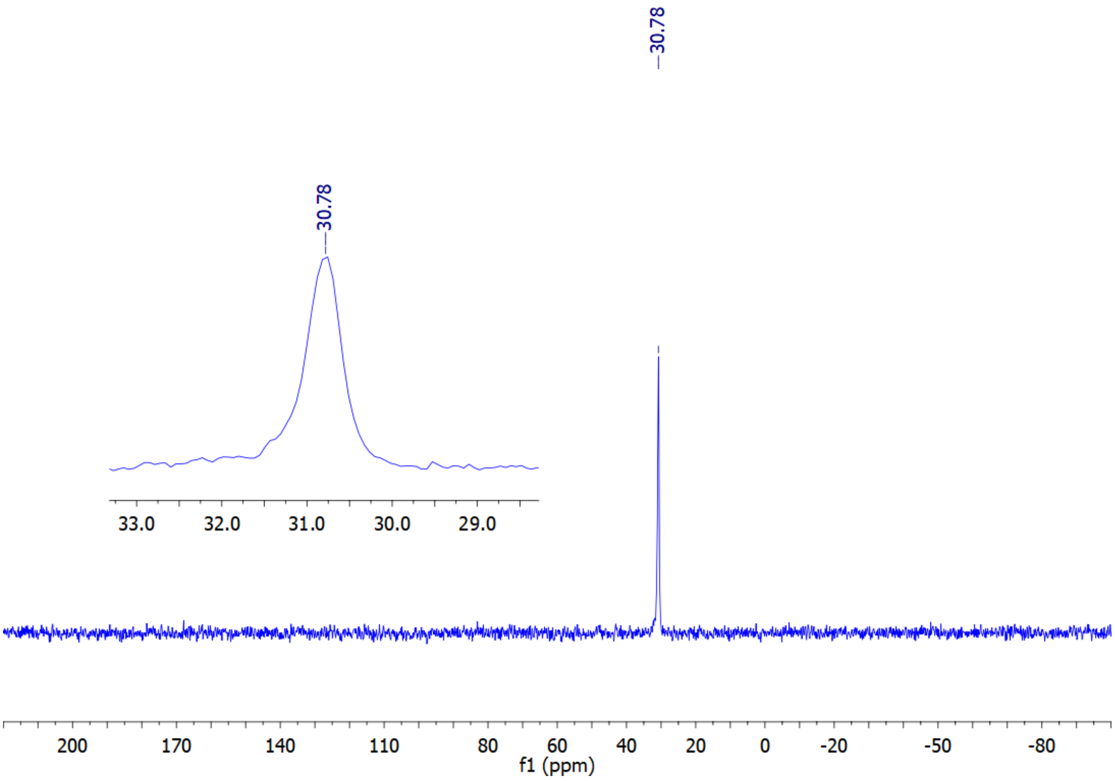

Figure S17. ^{31}P NMR spectrum (162 MHz, CH_3CN) of [$\mathbf{L_2P^+}$][OTf].

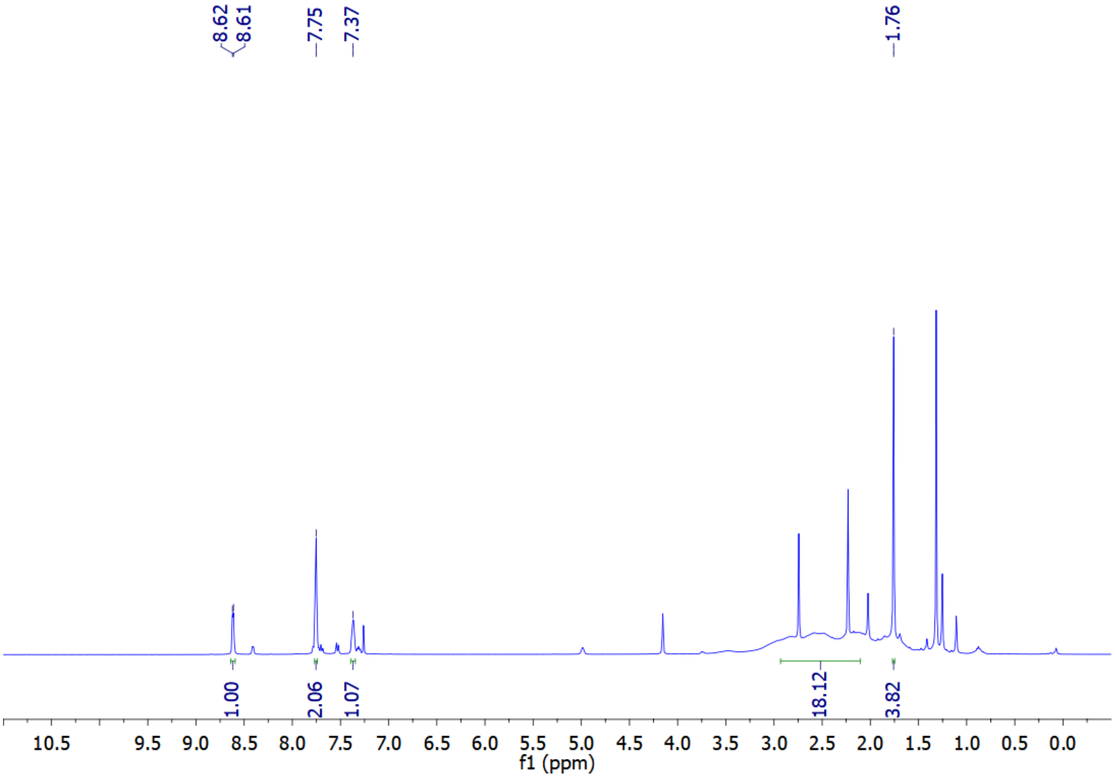

Figure S18. ^{1}H NMR spectrum (400 MHz, $CDCl_3$) of **LMe**.

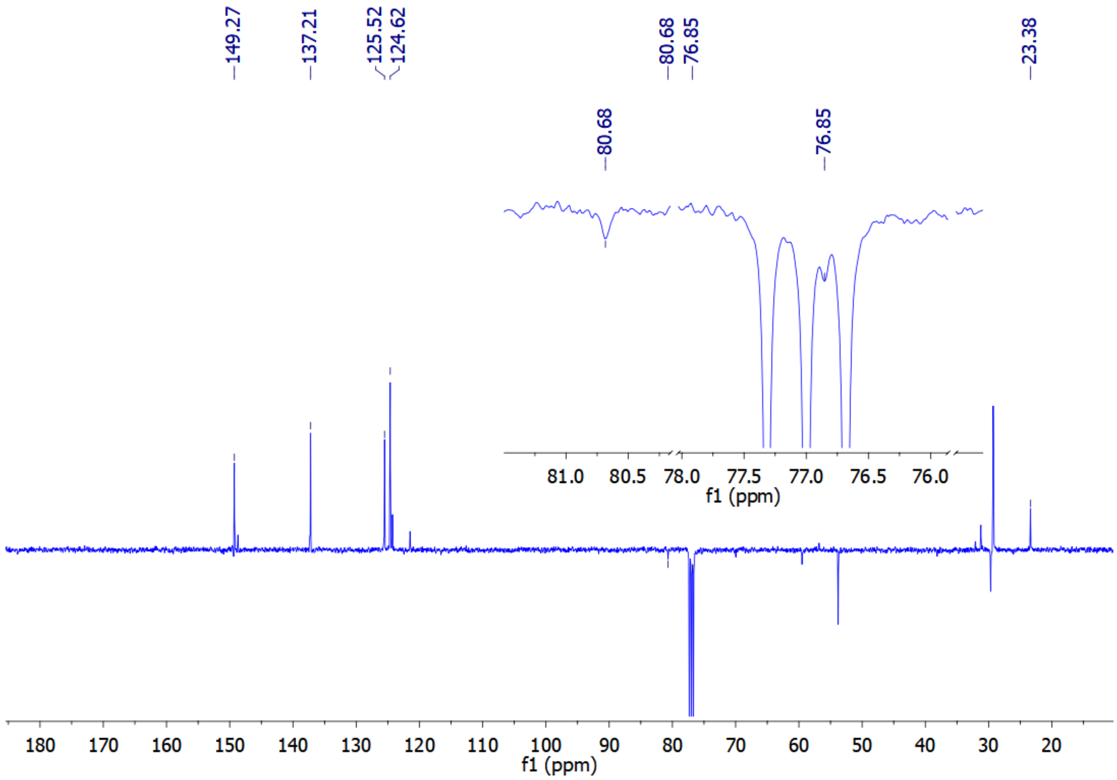

Figure S19. $^{13}C\{^1H\}$-JMOD NMR spectrum (100 MHz, $CDCl_3$) of **LMe**.

1.4 NMR of $[L_2P^+][B(C_6F_5)_4]$:

NMR values are given below to specify the peaks corresponding to respective atoms in the structure.

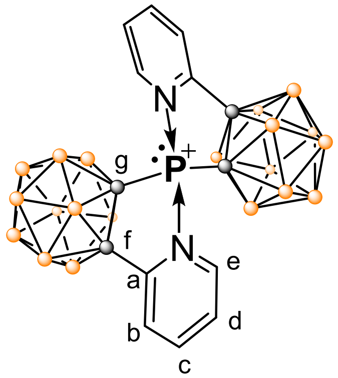

$[L_2P^+][B(C_6F_5)_4]$

^{1}**H NMR** (400 MHz, $CDCl_3$); δ = 8.73 (d, J = 5.4 Hz, 2H (H_d)), 8.37 (t, J = 7.7 Hz, 2H (H_e)), 7.91 (t, J = 6.4 Hz, 2H (H_c)), 7.83 (d, J = 7.8 Hz, 2H (H_b)), 3.5-1.5 (br, 20H, carborane$_{B\text{-}H}$) ppm.

^{13}C{^{1}H} NMR (100 MHz, $CDCl_3$); δ = 149.43 (d, J = 5.0 Hz, (C_a)), 148.09 (d, $J_{C\text{-}F}$ = 236.0 Hz, $B(C_6F_5)_4$), 146.41 (d, J = 8.6 Hz, (C_d)), 145.46(C_e), 138.13 (d, $J_{C\text{-}F}$ = 248.0 Hz, $B(C_6F_5)_4$), 136.25 (d, $J_{C\text{-}F}$ = 242.0 Hz, $B(C_6F_5)_4$), 126.64(C_c), 122.76(C_b), 76.43 (d, J = 10.4 Hz, $^{2}J_{P\text{–}C}$ (C_f)), 70.77 (d, J = 106.9 Hz, $^{1}J_{P\text{–}C}$ (C_g)) ppm.

^{31}P NMR (162 MHz, $CDCl_3$); δ = 36.03 ppm.

^{11}B NMR (128 MHz, $CDCl_3$); δ = –16.76 (s, $B(C_6F_5)_4$), –10.59 to 0.21 (br) ppm.

^{19}F NMR (376.5 MHz, $CDCl_3$); δ = –167.25, –163.18, –133.23 ppm.

HRMS (APCI positive mode): Calc. for C_{14} H_{28} $^{10}B_5$ $^{11}B_{15}$ N_2 P= 470.4039; found = 470.4040.

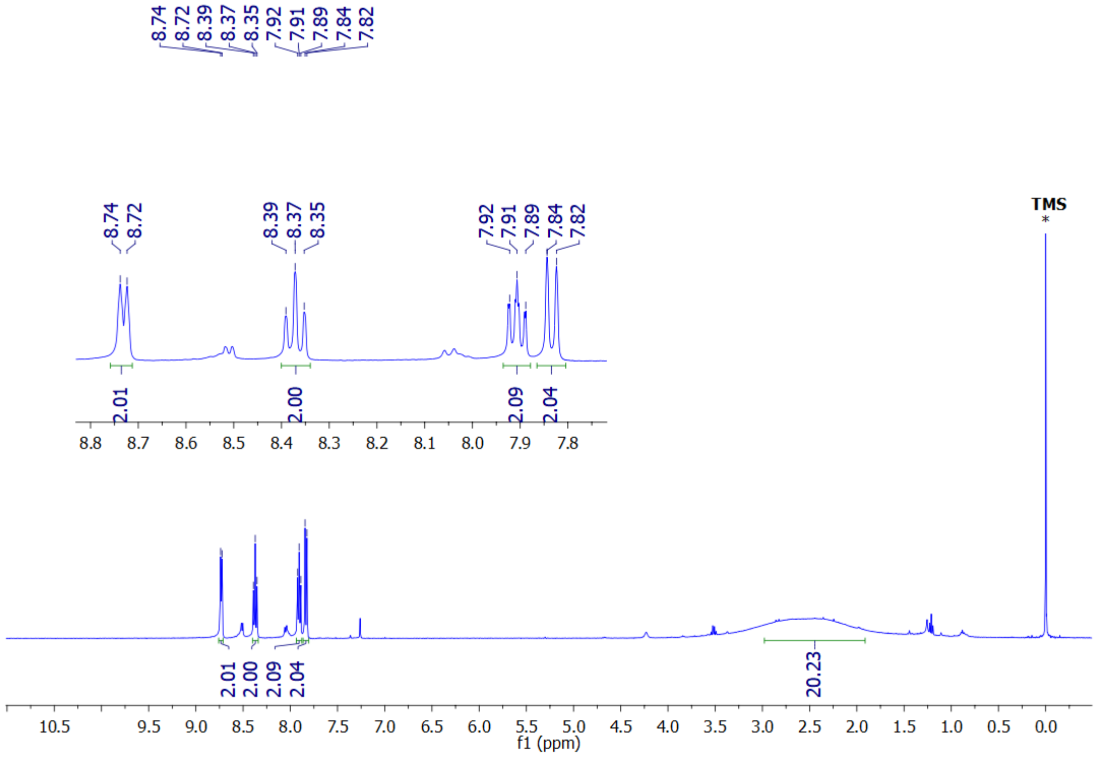

Figure S20. ^{1}H NMR spectrum (400 MHz, $CDCl_3$) of [$\mathbf{L_2P^+}$][$B(C_6F_5)_4$].

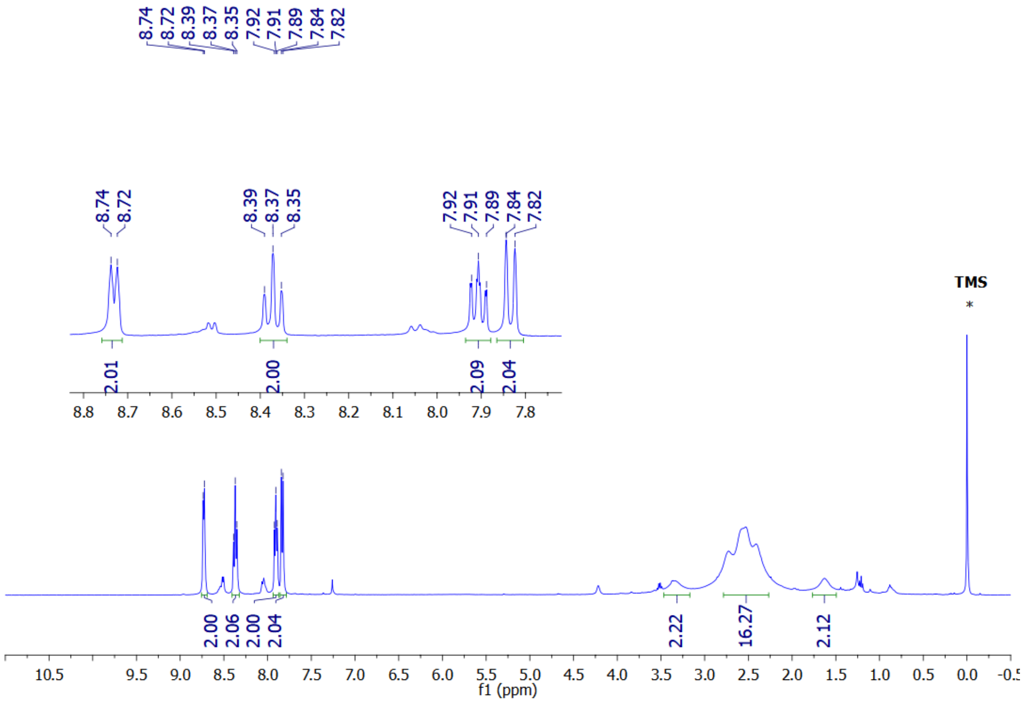

Figure S21. ${}^{1}H\{{}^{11}B\}$ NMR spectrum (400 MHz, $CDCl_3$) of [**L$_2$P$^+$**][$B(C_6F_5)_4$].

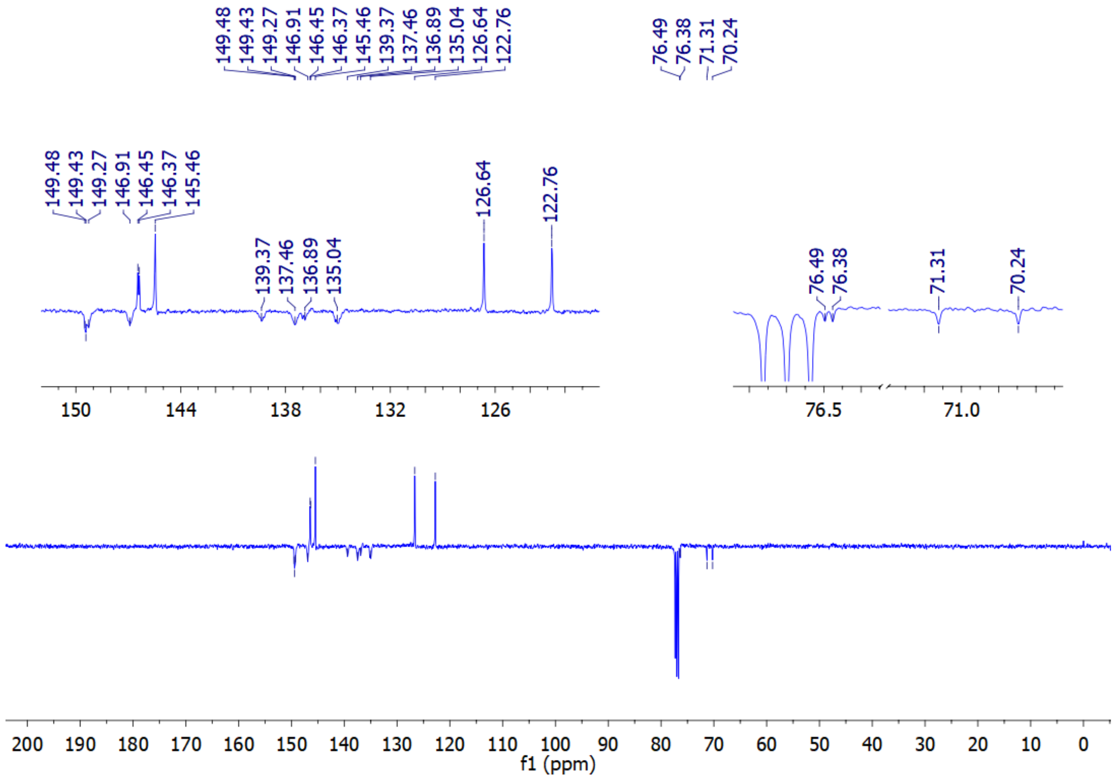

Figure S22. ${}^{13}C\{{}^{1}H\}$-JMOD NMR spectrum (100 MHz, $CDCl_3$) of [**L$_2$P$^+$**][$B(C_6F_5)_4$].

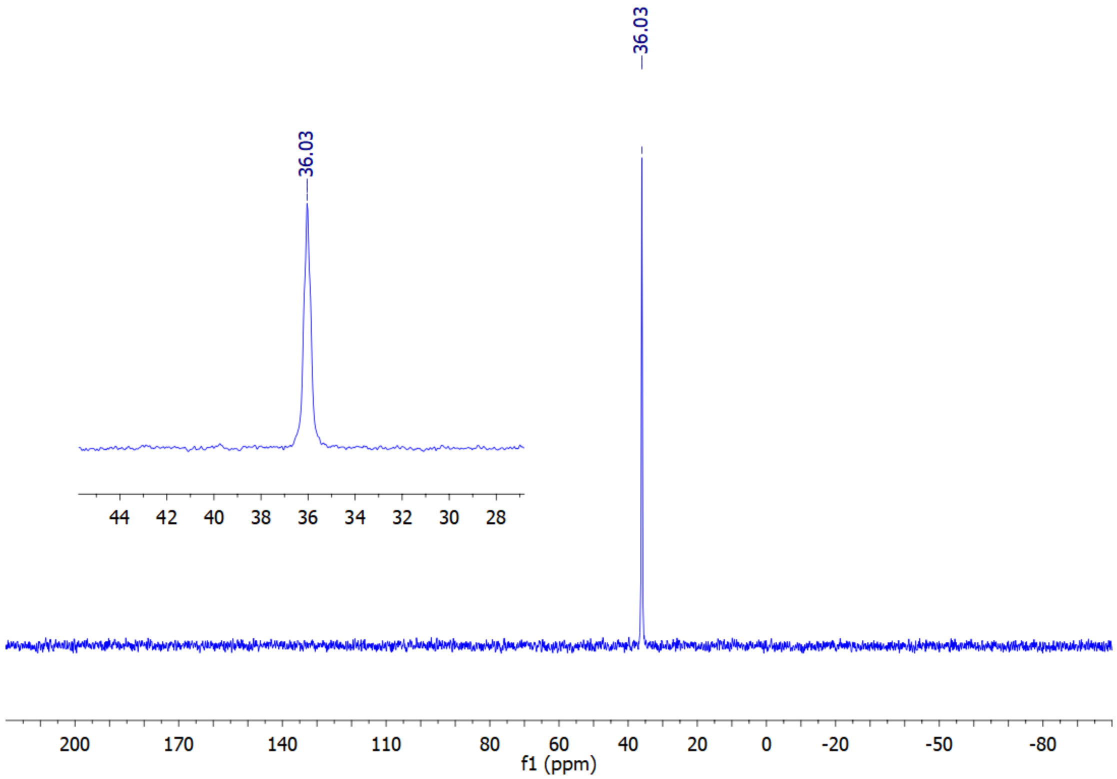

Figure S23. ^{31}P NMR spectrum (162 MHz, $CDCl_3$) of [$\mathbf{L_2P^+}$][$B(C_6F_5)_4$].

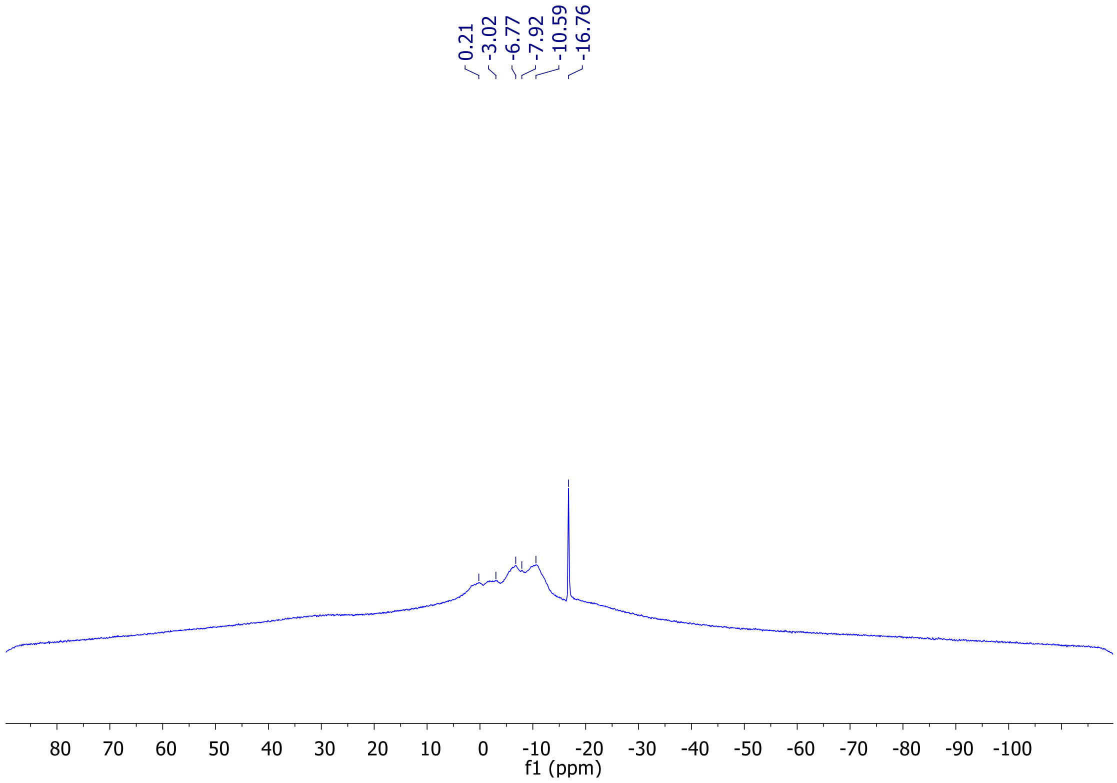

Figure S24. ^{11}B NMR spectrum (128 MHz, $CDCl_3$) of [$\mathbf{L_2P^+}$][$B(C_6F_5)_4$].

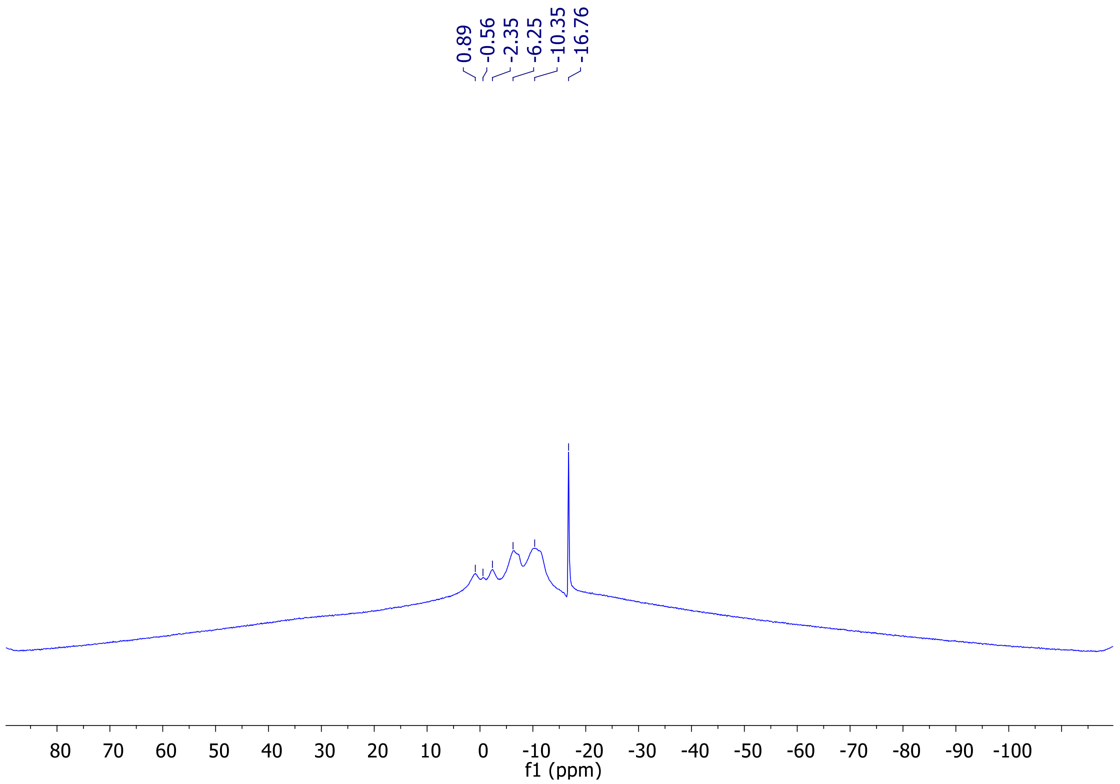

Figure S25. $^{11}B\{^{1}H\}$ NMR spectrum (128 MHz, $CDCl_3$) of [$\mathbf{L_2P^+}$][$B(C_6F_5)_4$].

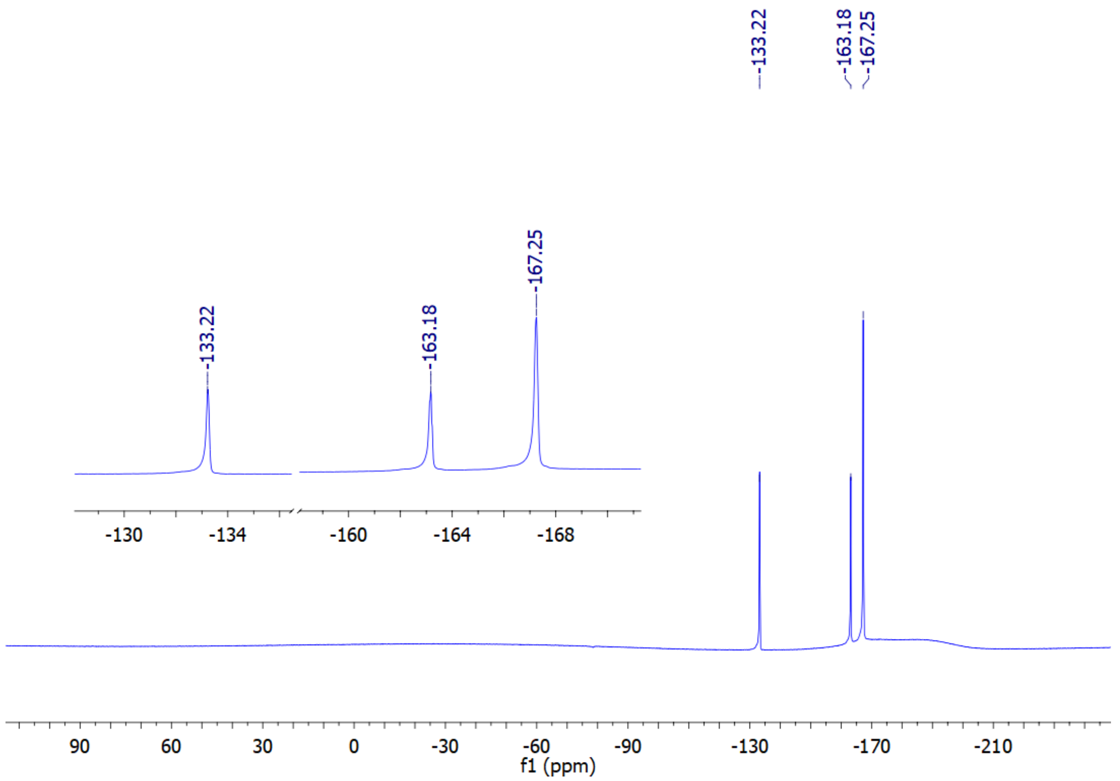

Figure S26. ^{19}F NMR spectrum (376.5 MHz, $CDCl_3$) of [$\mathbf{L_2P^+}$][$B(C_6F_5)_4$].

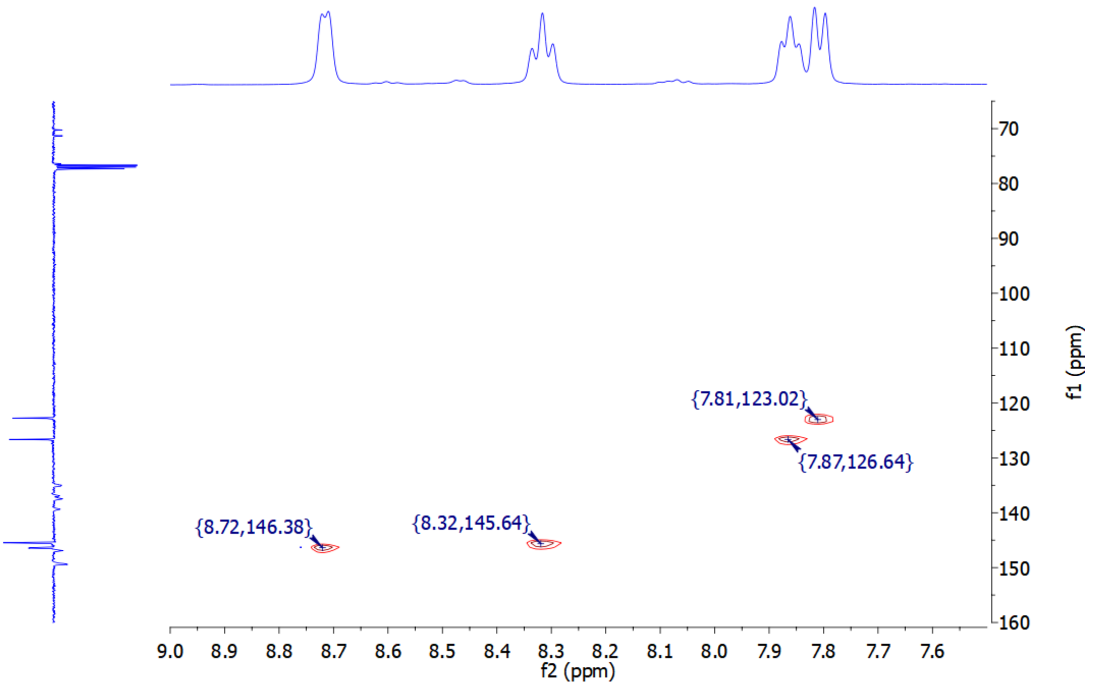

Figure S27. ^{1}H-^{13}C HSQC NMR spectrum ($CDCl_3$) of [$\mathbf{L_2P^+}$][$B(C_6F_5)_4$].

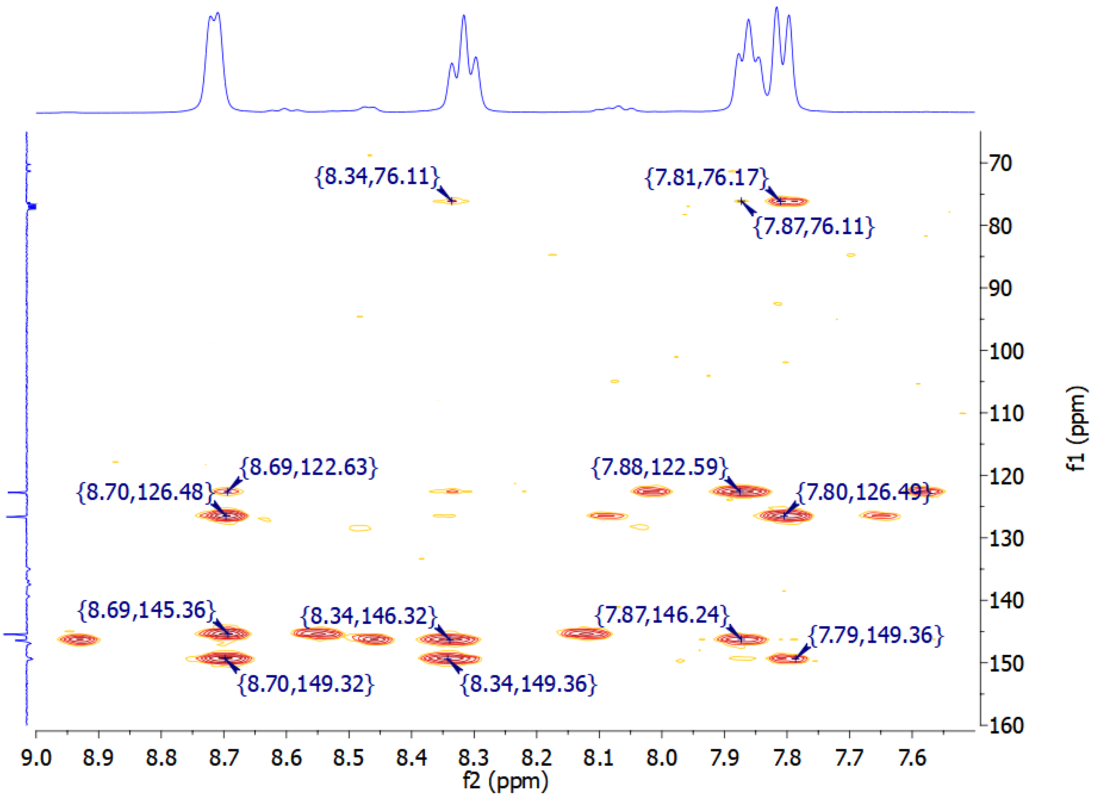

Figure S28. ^{1}H-^{13}C HMBC NMR spectrum ($CDCl_3$) of [$\mathbf{L_2P^+}$][$B(C_6F_5)_4$].

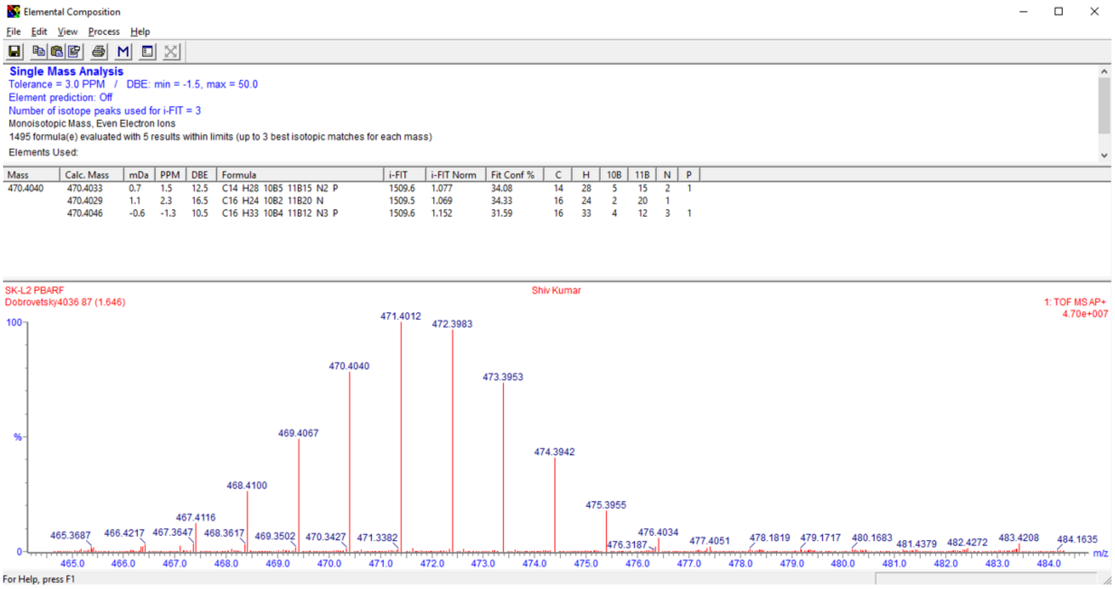

Figure S29. HRMS spectrum of [$\mathbf{L_2P^+}$][$B(C_6F_5)_4$].

Reaction of [L_2P^+][$B(C_6F_5)_4$] with O–H bond containing molecules:

In a J-Young NMR tube, a solution of [$\mathbf{L_2P^+}$][$B(C_6F_5)_4$] (50 mg, 0.043 mmol) was prepared in 0.5 mL $CDCl_3$ and followed by the addition of an excess amount of the respective alcohols or H_2O.

1.5 Reaction of [L_2P^+][$B(C_6F_5)_4$] with H_2O.

In a J-Young NMR tube, a solution of [$\mathbf{L_2P^+}$][$B(C_6F_5)_4$] (50 mg, 0.043 mmol) was prepared in 0.5 mL $CDCl_3$ and followed by the addition of 5 equiv of H_2O. The reaction proceeded at r.t.

Next, the reaction mixture was evaporated, and fresh $CDCl_3$ was added. In the ^{31}P NMR spectrum, a doublet at 13.53 ppm (J = 646 Hz) indicated the formation of a P–H moiety, which was corroborated by a corresponding doublet in the ^{1}H NMR spectrum (d: 8.17 and 6.55 ppm, J = 646 Hz; in ^{1}H{^{31}P} NMR at 7.36 ppm). Moreover, a peak at 4.67 ppm corresponding to C–H of [$\mathbf{LH_2^+}$][$B(C_6F_5)_4$] was also observed in the ^{1}H NMR spectrum. Interestingly, the ^{1}H NMR spectrum also displayed a broad singlet at 15.94 ppm, attributed to a pyridinium N–H proton and a peak at 4.57 ppm (in ^{13}C NMR at 58.63 ppm) corresponding to C–H. These resonances are consistent with the formation of the protonated ligand salt [$\mathbf{LH_2^+}$][$B(C_6F_5)_4$], the identity of which was further confirmed by SC-XRD analysis (see figure S30). These resonances are consistent with the formation of the protonated ligand salt [$\mathbf{LH_2^+}$][$B(C_6F_5)_4$], the identity of which was further confirmed by SC-XRD analysis (*vide supra*). Combined ^{1}H and ^{31}P NMR data unequivocally support the formation of [$\mathbf{LH_2^+}$][$B(C_6F_5)_4$] and the alcoholysis byproduct $(HO)_2P(O)H$.10-1[2-3]

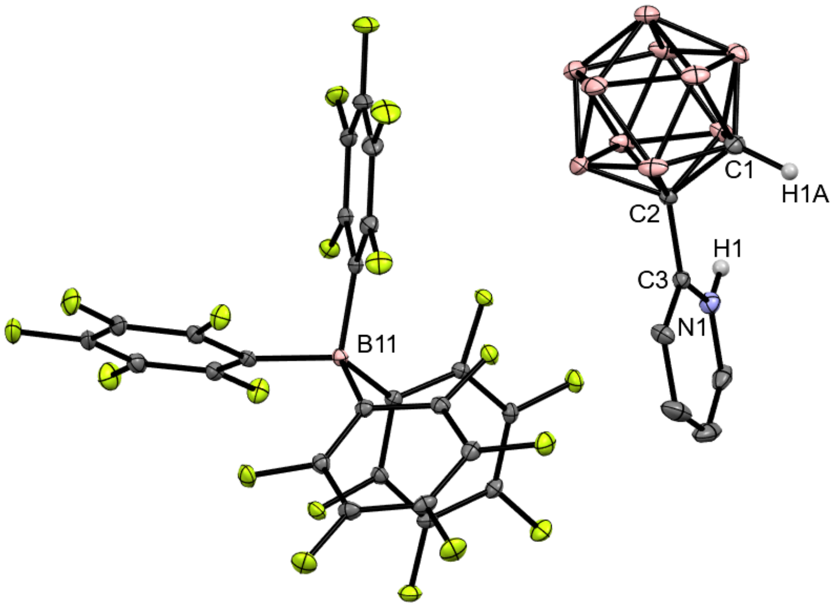

Figure S30. Molecular structures of [$\mathbf{LH_2^+}$][$B(C_6F_5)_4$]. Thermal ellipsoids at 50% probability; non-relevant hydrogen atoms were omitted for clarity (CCDC number - 2512661).

NMR

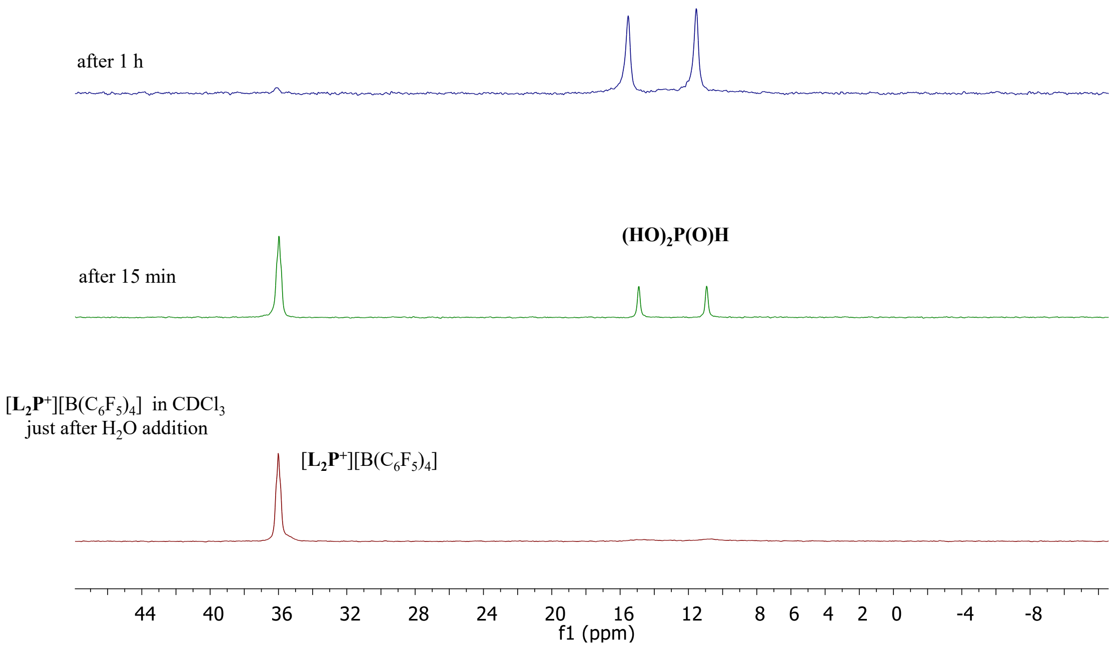

Figure S31. Stacked ^{31}P NMR spectra showing the progress of the reaction between [$\mathbf{L_2P^+}$][$B(C_6F_5)_4$] and H_2O in $CDCl_3$ over time.

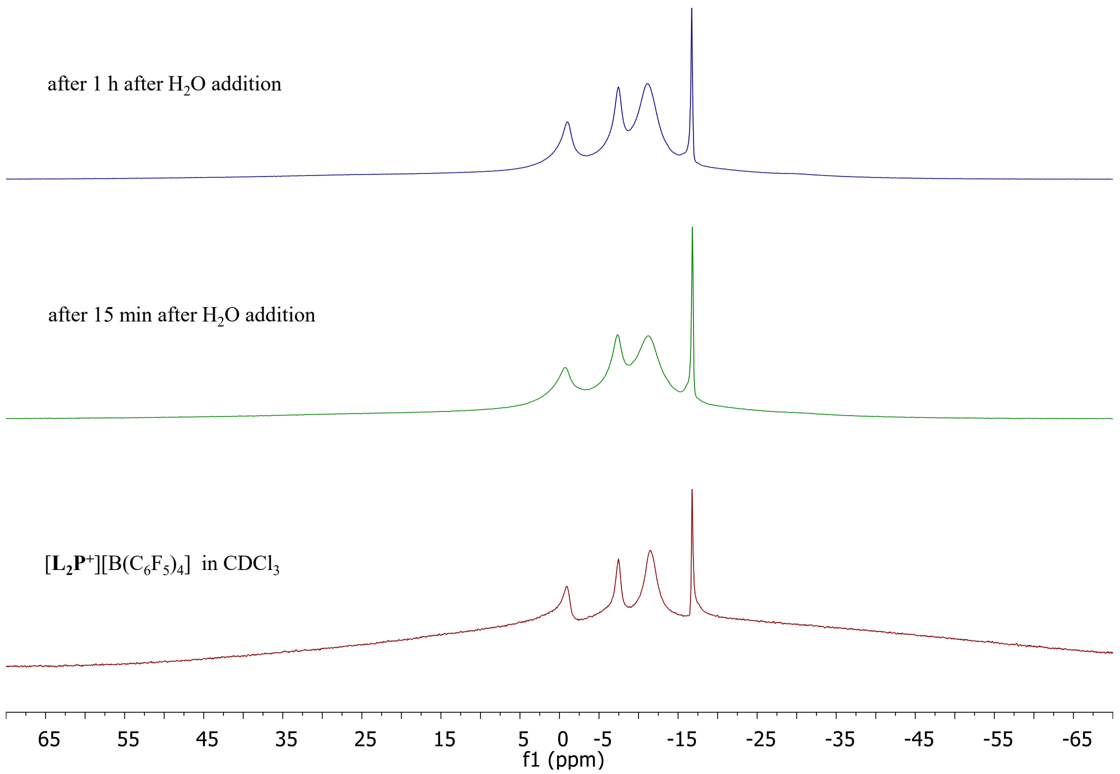

Figure S32. Stacked $^{11}B\{^1H\}$ NMR spectra showing the progress of the reaction between [$\mathbf{L_2P^+}$][$B(C_6F_5)_4$] and H_2O in $CDCl_3$ over time.

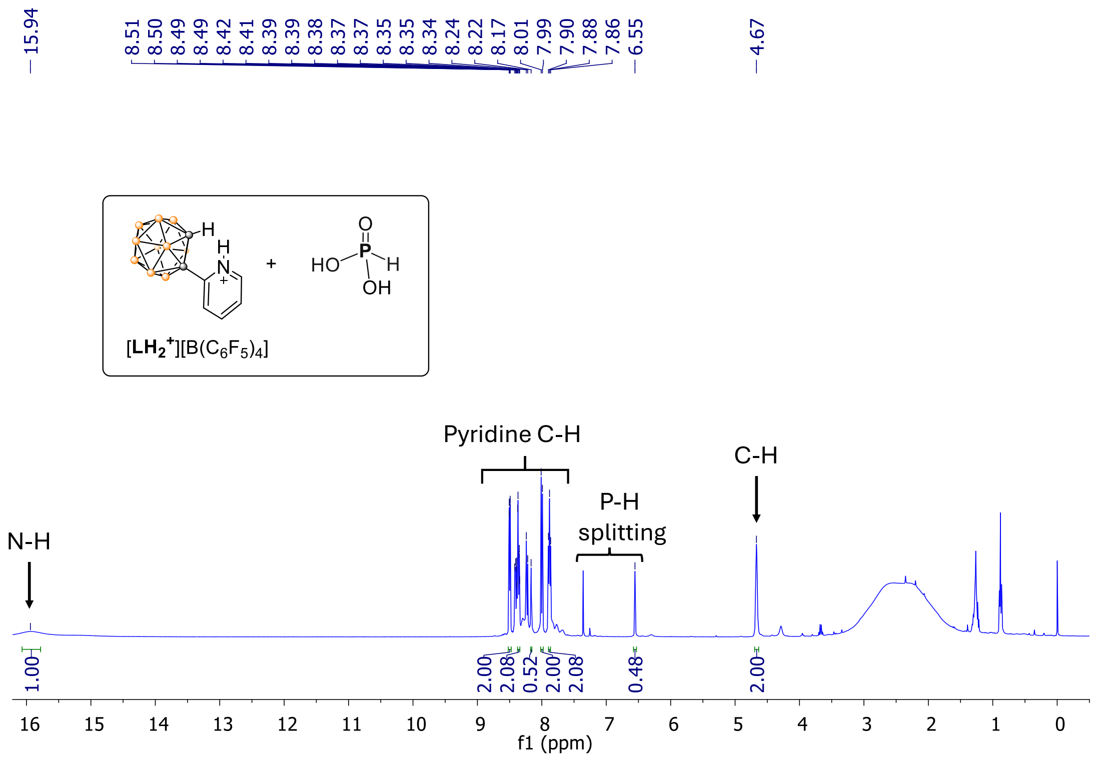

Figure S33. ^{1}H NMR spectrum (400 MHz, $CDCl_3$) of the reaction mixture of [**L_2P^+**][$B(C_6F_5)_4$] and H_2O.

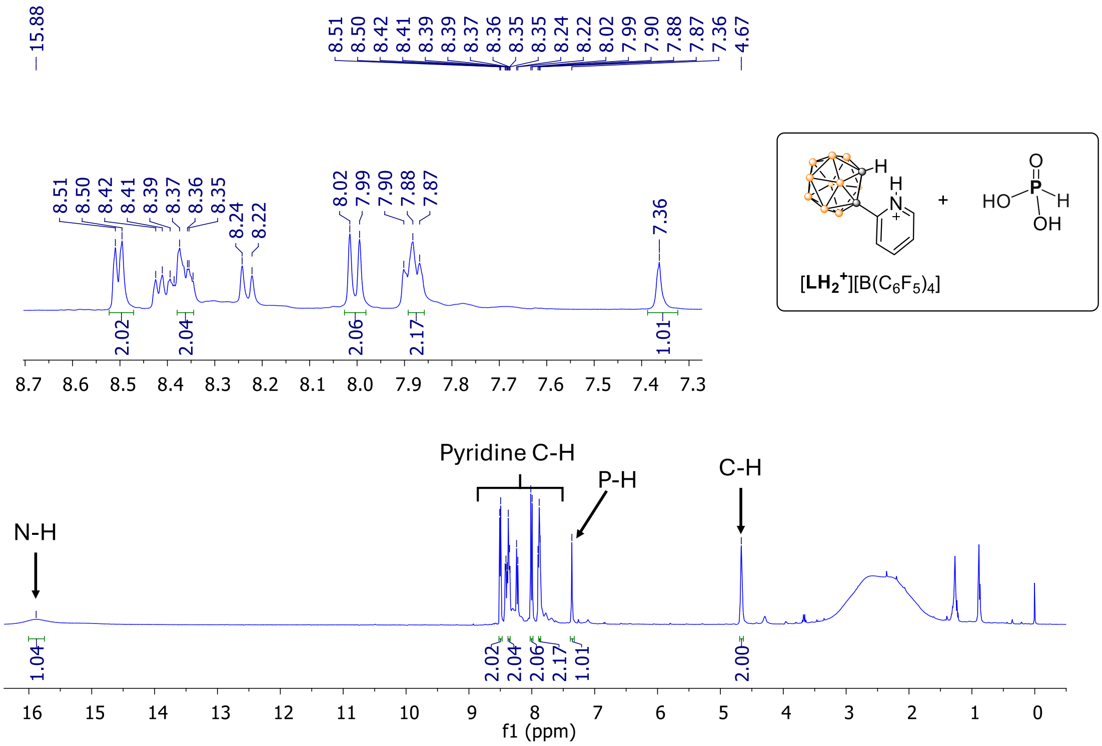

Figure S34. ^{1}H{^{31}P} NMR spectrum (400 MHz, $CDCl_3$) of the reaction mixture of [**L_2P^+**][$B(C_6F_5)_4$] and H_2O.

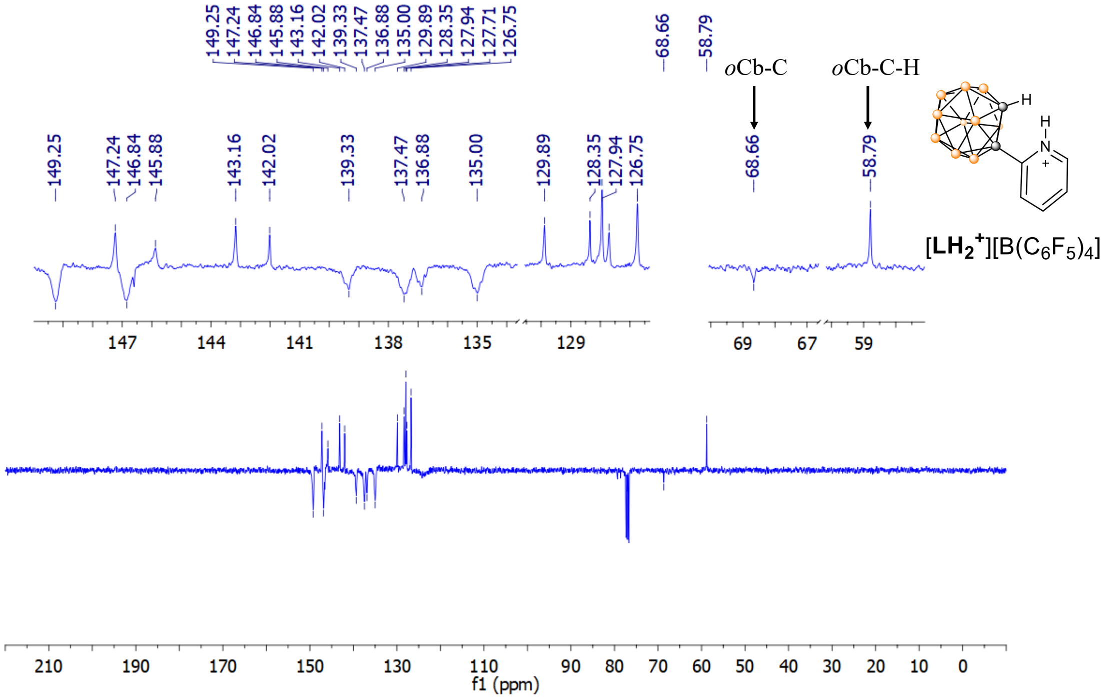

Figure S35. $^{13}C\{^{1}H\}$-JMOD NMR spectrum (100 MHz, $CDCl_3$) of the reaction mixture of [$\mathbf{L_2P}^+$][$B(C_6F_5)_4$] and H_2O.

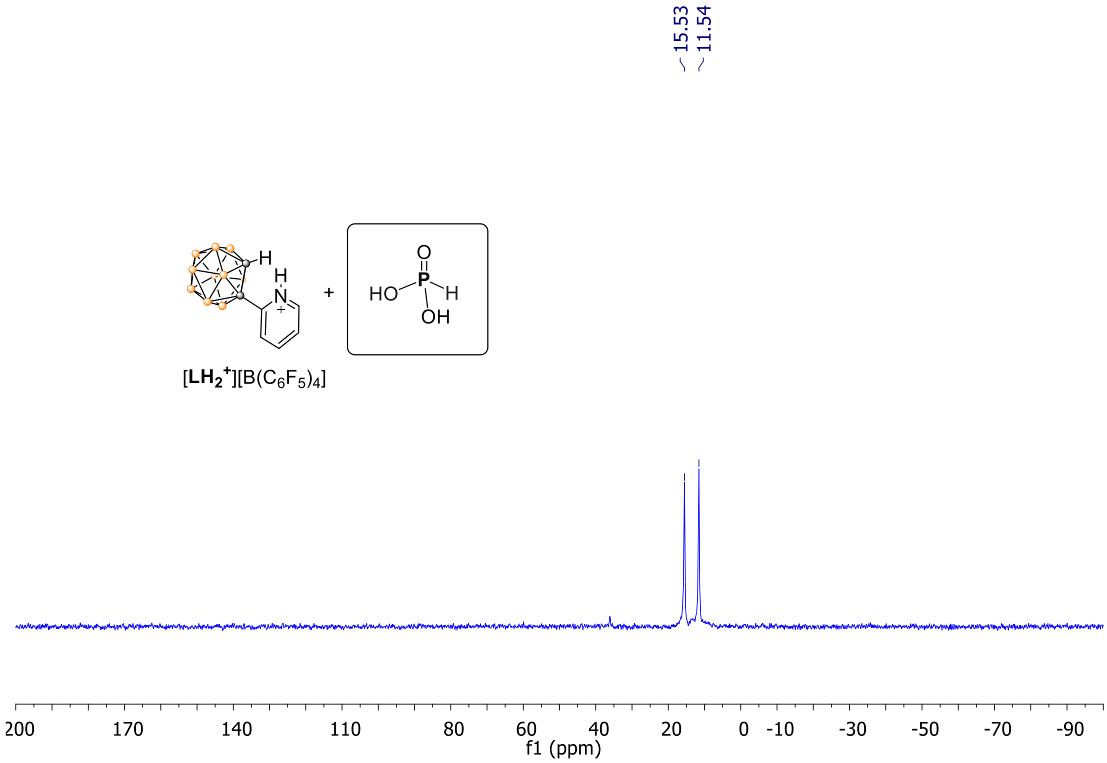

Figure S36. ^{31}P NMR spectrum (162 MHz, $CDCl_3$) of [$\mathbf{L_2P}^+$][$B(C_6F_5)_4$] and H_2O.

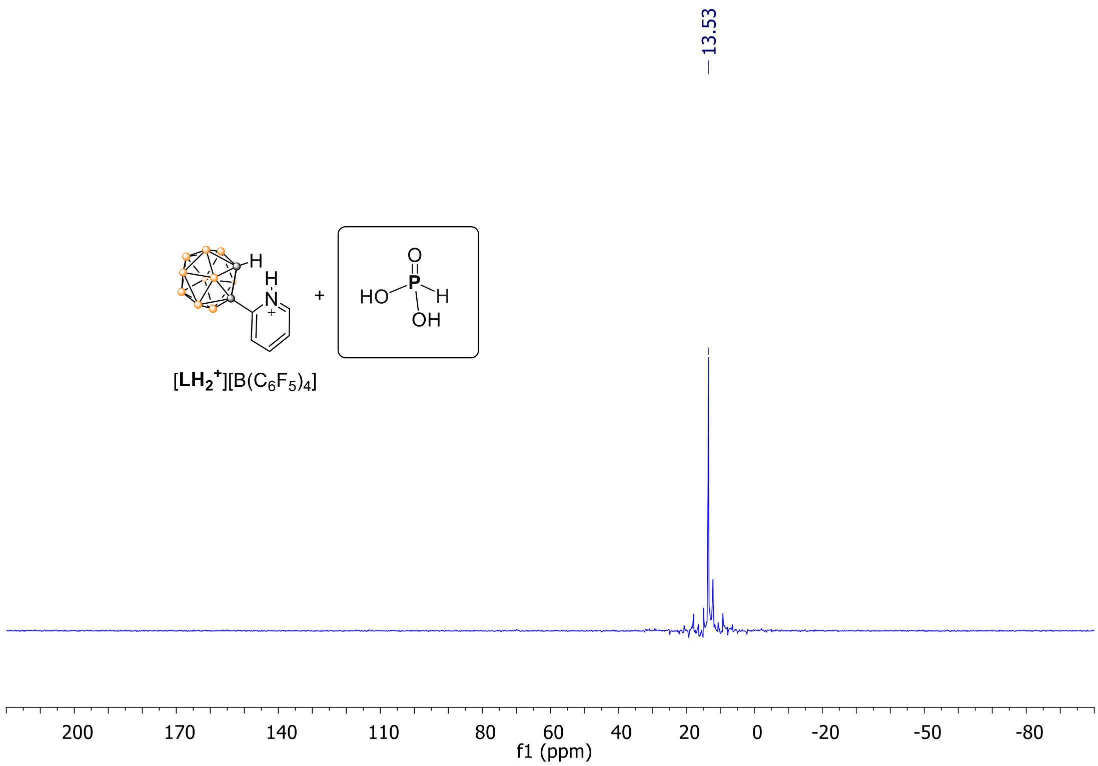

Figure S37. $^{31}P\{^1H\}$ NMR spectrum (162 MHz, $CDCl_3$) of [$\mathbf{L_2P^+}$][$B(C_6F_5)_4$] and H_2O.

1.6 Reaction of [L_2P^+][$B(C_6F_5)_4$] with MeOH.

In a J-Young NMR tube, a solution of [$\mathbf{L_2P^+}$][$B(C_6F_5)_4$] (50 mg, 0.043 mmol) was prepared in 0.5 mL of $CDCl_3$, followed by the addition of 5 equivalents of MeOH. The reaction showed no progress at room temperature, so the mixture was subsequently heated to 85 °C. After 36 h, complete consumption of [$\mathbf{L_2P^+}$] was observed, accompanied by the appearance of a new doublet at 5 ppm in the ^{31}P NMR spectrum. The reaction mixture was then dried under vacuum to remove excess MeOH, and fresh $CDCl_3$ was added for further analysis. In the ^{31}P NMR spectrum, a doublet at 11.0 ppm (J = 706 Hz) indicated the formation of a P−H moiety, which was corroborated by a corresponding doublet in the ^1H NMR spectrum (d: 7.67 and 5.88 ppm, J = 706 Hz). Moreover, a doublet at 3.85 ppm (J = 12.2 Hz) in the ^1H NMR spectrum and its corresponding doublet at 53.24 ppm (J = 7 Hz) in the ^{13}C NMR spectrum were assigned to a P−bound −OMe group.

Interestingly, the ^1H NMR spectrum also displayed a broad singlet at 13.7 ppm, attributed to a pyridinium N−H proton and a peak at 4.57 ppm (in ^{13}C NMR at 58.63 ppm) corresponding to C−H. These resonances are consistent with the formation of the protonated ligand salt [$\mathbf{LH_2^+}$][$B(C_6F_5)_4$], the identity of which was further confirmed by SC-XRD analysis (see figure S30). Combined ^1H and ^{31}P NMR data unequivocally support the formation of [$\mathbf{LH_2^+}$][$B(C_6F_5)_4$] and the alcoholysis byproduct $(MeO)_2P(O)H$.[2-3]

NMR

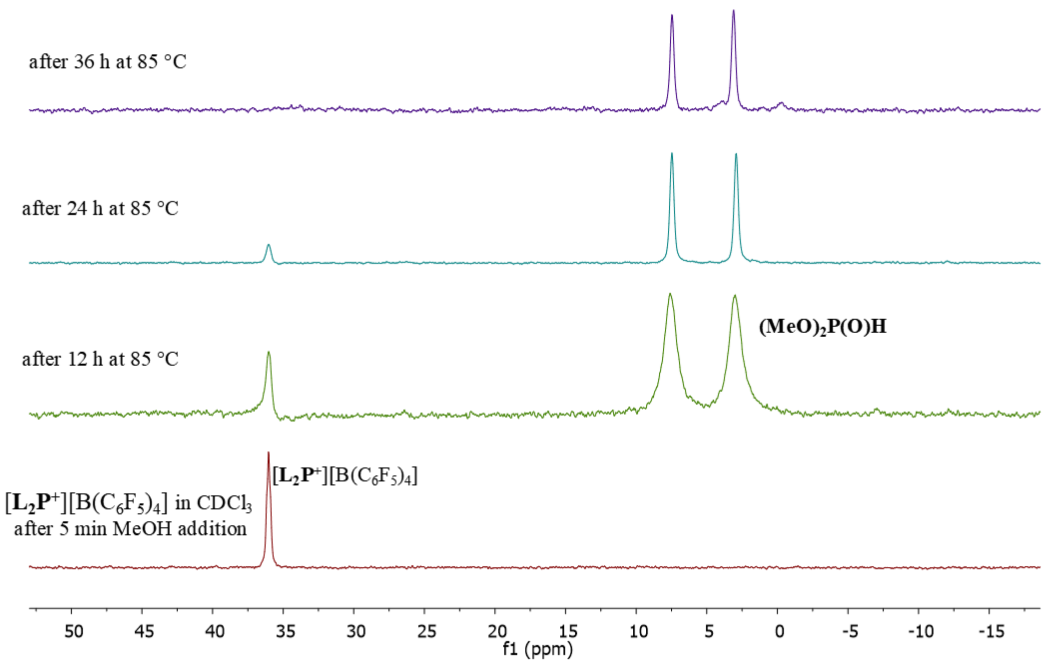

Figure S38. Stacked ^{31}P NMR spectra showing the progress of the reaction between [$\mathbf{L_2P^+}$][$B(C_6F_5)_4$] and MeOH in $CDCl_3$ at 85 °C over time.

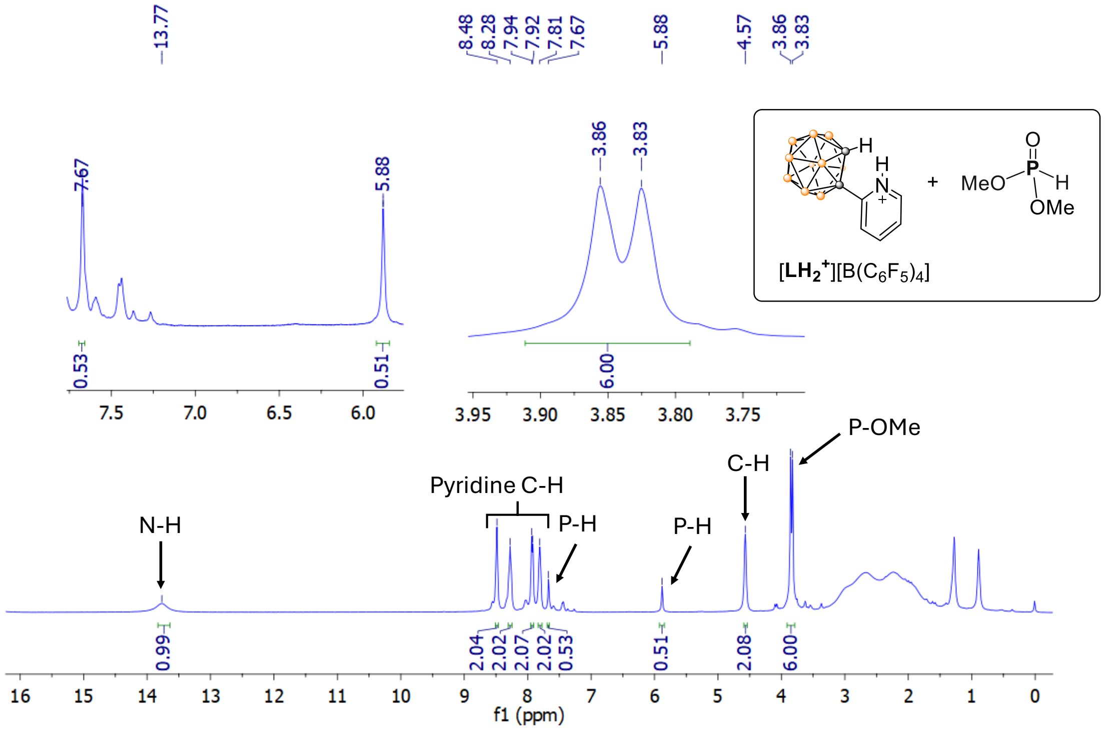

Figure S39. ^1H NMR spectrum (400 MHz, $CDCl_3$) of the reaction mixture of [$\mathbf{L_2P^+}$][$B(C_6F_5)_4$] and MeOH.

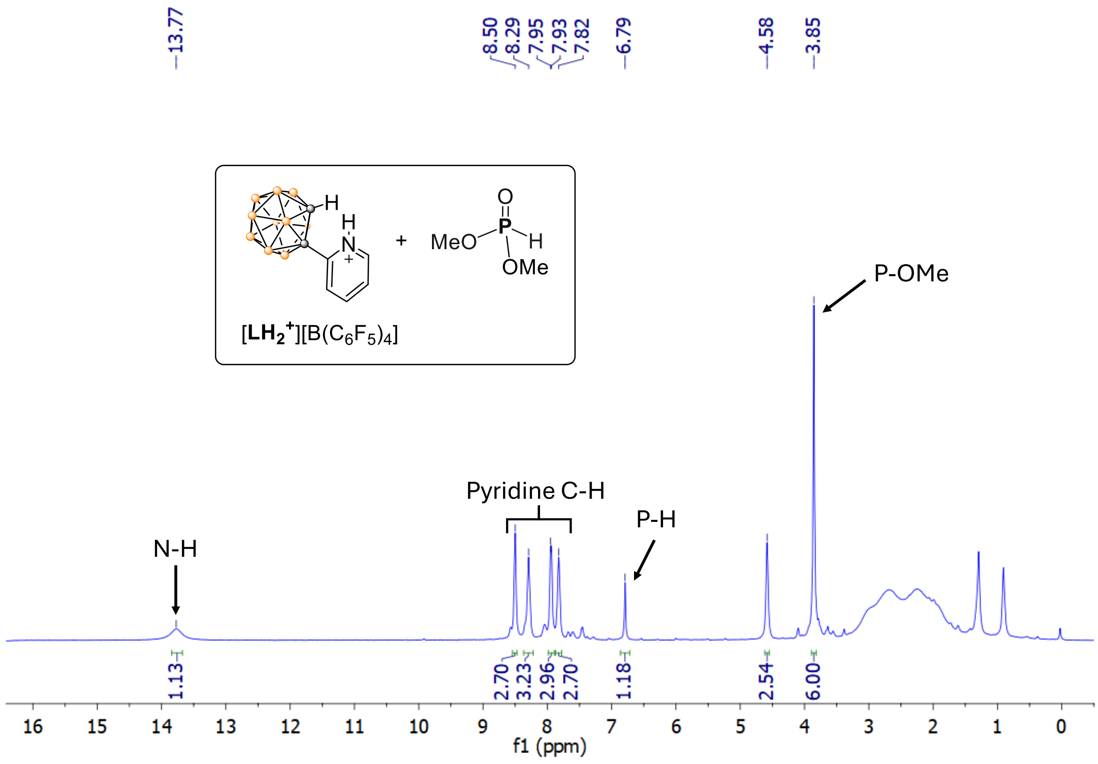

Figure S40. $^{1}H\{^{31}P\}$ NMR spectrum (400 MHz, $CDCl_3$) of the reaction mixture of [$\mathbf{L_2P^+}$][$B(C_6F_5)_4$] and MeOH.

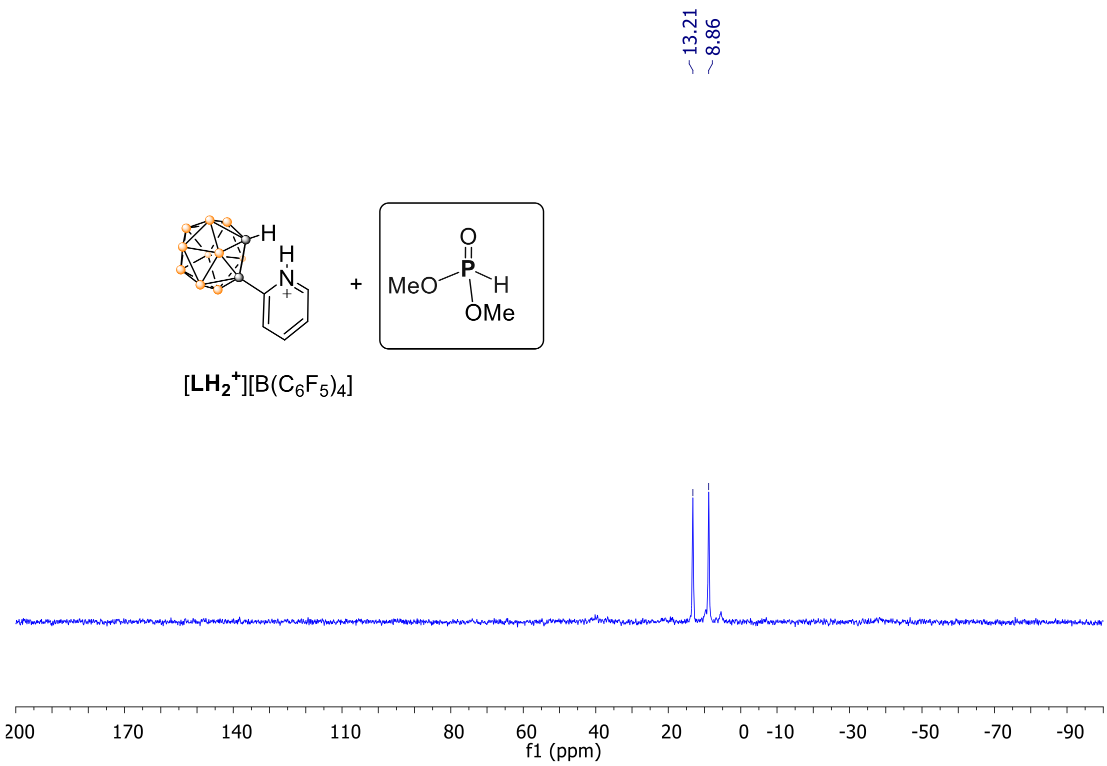

Figure S41. ^{31}P NMR spectrum (162 MHz, $CDCl_3$) of [$\mathbf{L_2P^+}$][$B(C_6F_5)_4$] and MeOH.

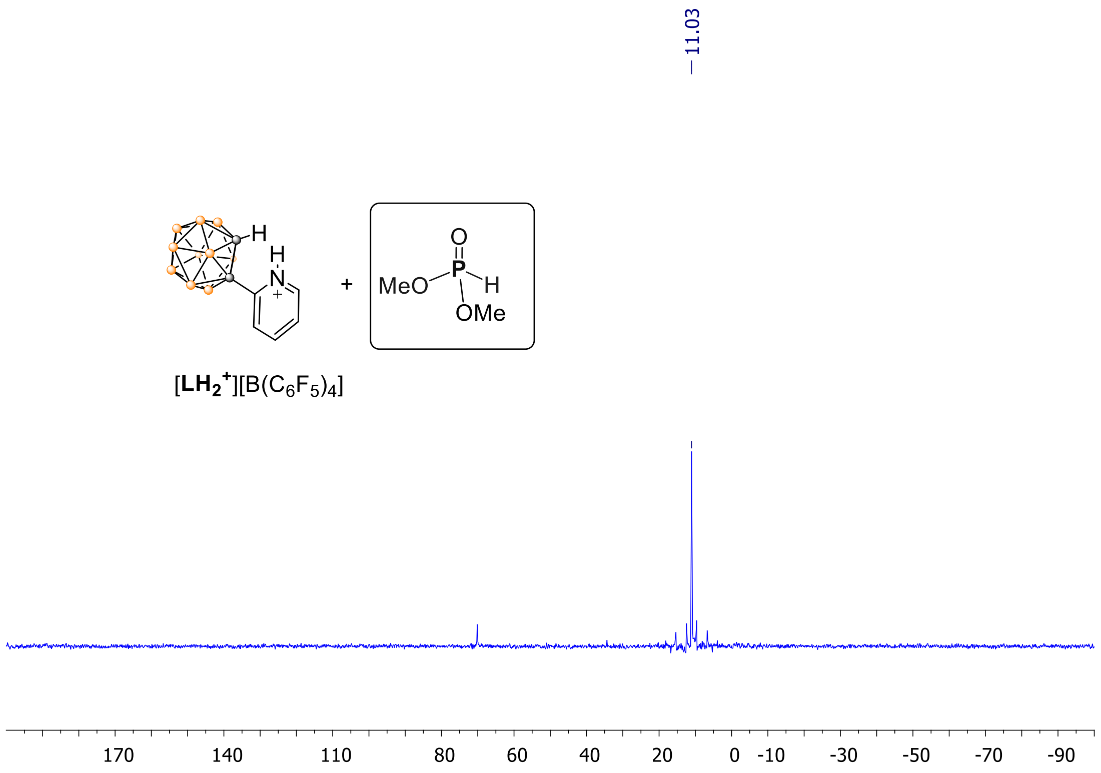

Figure S42. $^{31}P\{^1H\}$ NMR spectrum (162 MHz, $CDCl_3$) of [$\mathbf{L_2P^+}$][$B(C_6F_5)_4$] and MeOH.

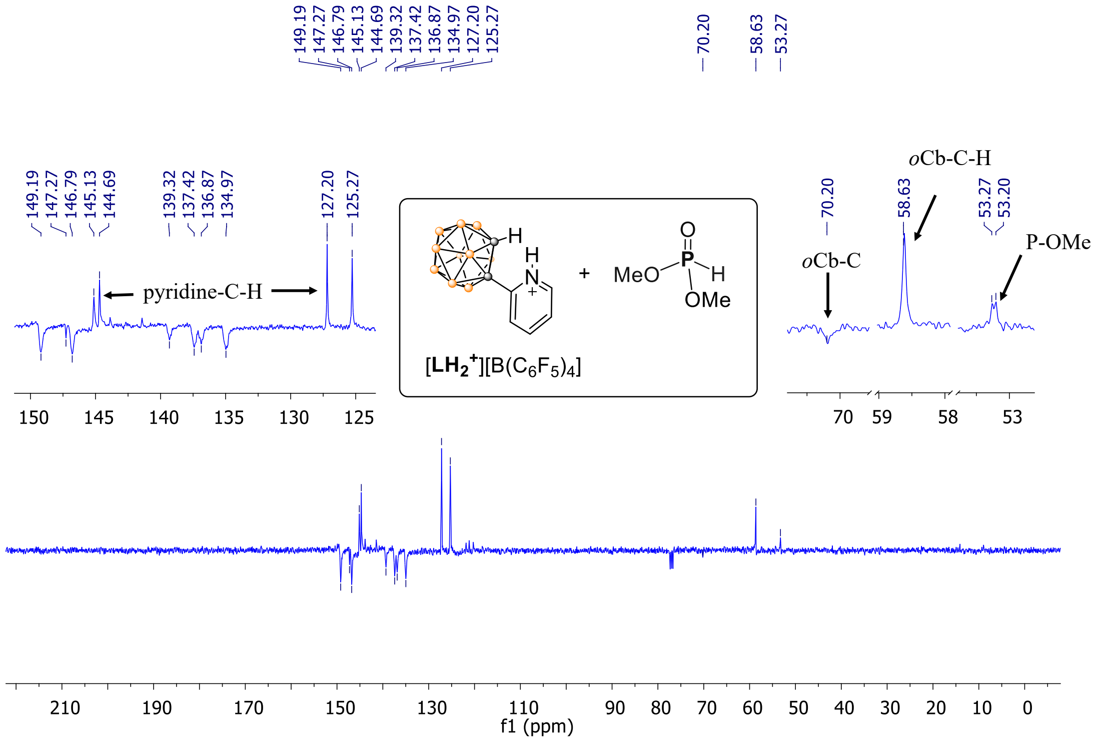

Figure S43. $^{13}C\{^1H\}$-JMOD NMR spectrum (100 MHz, $CDCl_3$) of the reaction mixture of [$\mathbf{L_2P^+}$][$B(C_6F_5)_4$] and MeOH.

Notably, similar C–P bond alcoholysis was observed with phenol, resulting in the dissociation of the ligand unit and the phosphorus moiety.

Furthermore, a possible mechanism for the alcoholysis of **L2P**$^+$ with MeOH was DFT calculated using B3LYP-D3(BJ)/6-311++G(d,p) level of theory.

..

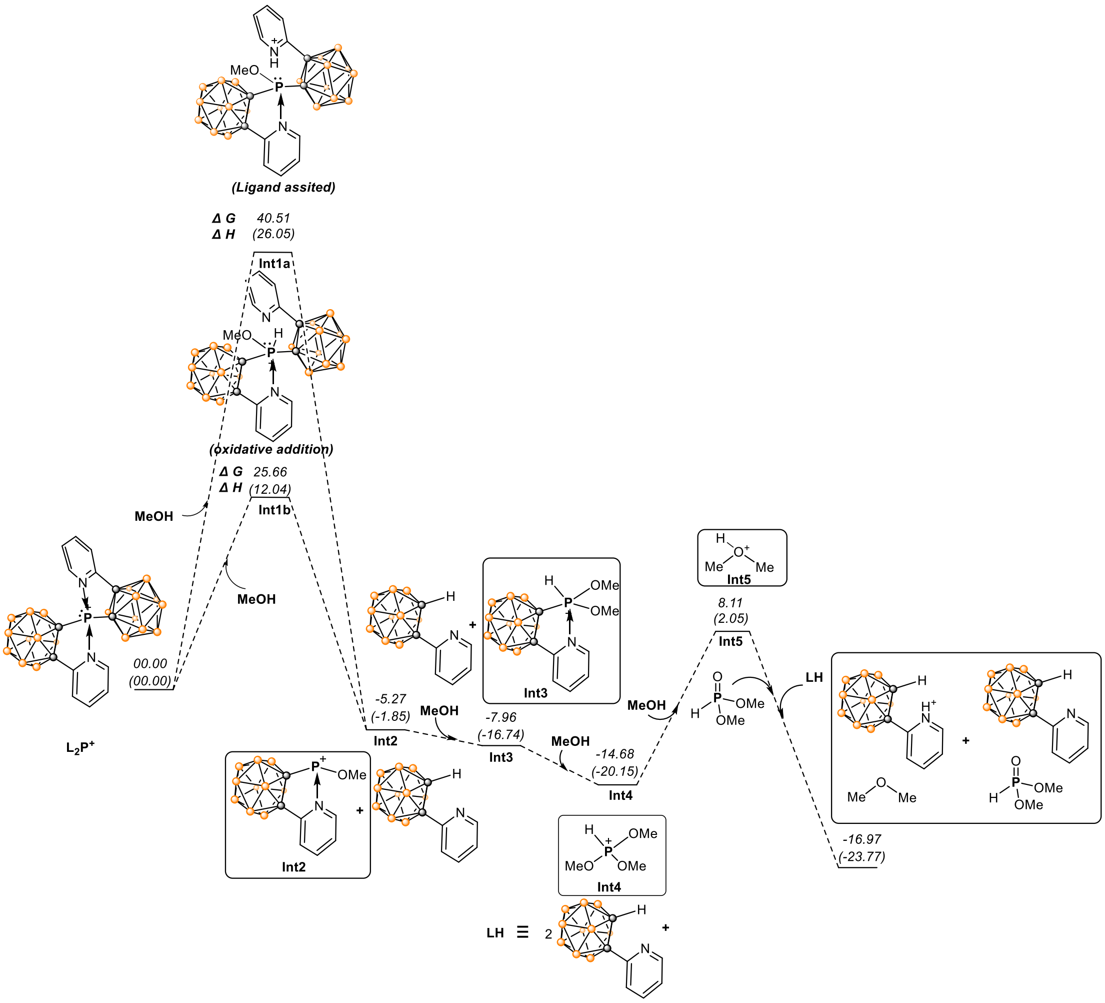

Figure S44. DFT-computed plausible mechanism for methanolysis of $\mathbf{L_2P^+}$, energies are in kcal·mol^{-1}.

Reaction of [L_2P^+][$B(C_6F_5)_4$] with N–H bond containing molecules:

In a J-Young NMR tube, a solution of [$\mathbf{L_2P^+}$][$B(C_6F_5)_4$] (50 mg, 0.043 mmol) was prepared in 0.5 mL $CDCl_3$ and followed by the addition of an equimolar amount of the respective amines.

1.7 Reaction of [L_2P^+][$B(C_6F_5)_4$] with Et_2NH.

In a J-Young NMR tube was charged with a 0.5 mL solution of [$\mathbf{L_2P^+}$][$B(C_6F_5)_4$] (63 mg, 0.054 mmol) in $CDCl_3$, followed by the addition of diethylamine (Et_2NH 5.6 µL, 0.054 mmol). The reaction progress was monitored using ^{31}P NMR spectroscopy at r.t.. In ^{31}P NMR, after 1 h, the signal at 36 ppm, corresponding to $\mathbf{L_2P^+}$, decreased, and a new signal appeared at 78 ppm. After 3 h, an additional signal at 69 ppm emerged, with approximately 50% of [$\mathbf{L_2P^+}$][$B(C_6F_5)_4$] consumed. Two additional equiv of Et_2NH were added, which finally led to 69 ppm as the major signal, while the signals at 35 and 78 ppm completely disappeared (^{31}P NMR stack plot). Thus with excess diethylamine and at r.t., the reaction finishes in 24 h. Following the completion of the reaction, the solvent was evaporated, and the residue was washed with hexane (2 × 5 mL). The isolated solid exhibits very low solubility in benzene. The product was isolated by crystallization (21 mg, 84%) from a THF:benzene (1:5) mixture via slow evaporation at room temperature. The SC-XRD of these crystals was measured, determining the molecular structure of the obtained product as a zwitterion ($\mathbf{L(L^{nido})P^+}$). Notably, one of the carborane units in $\mathbf{L(L^{nido})P^+}$ was found to undergo transformation into a *nido* cluster, likely via deborylation of the *closo* carborane cluster present in [$\mathbf{L_2P^+}$], while the other *closo* carborane unit remains intact.[4]

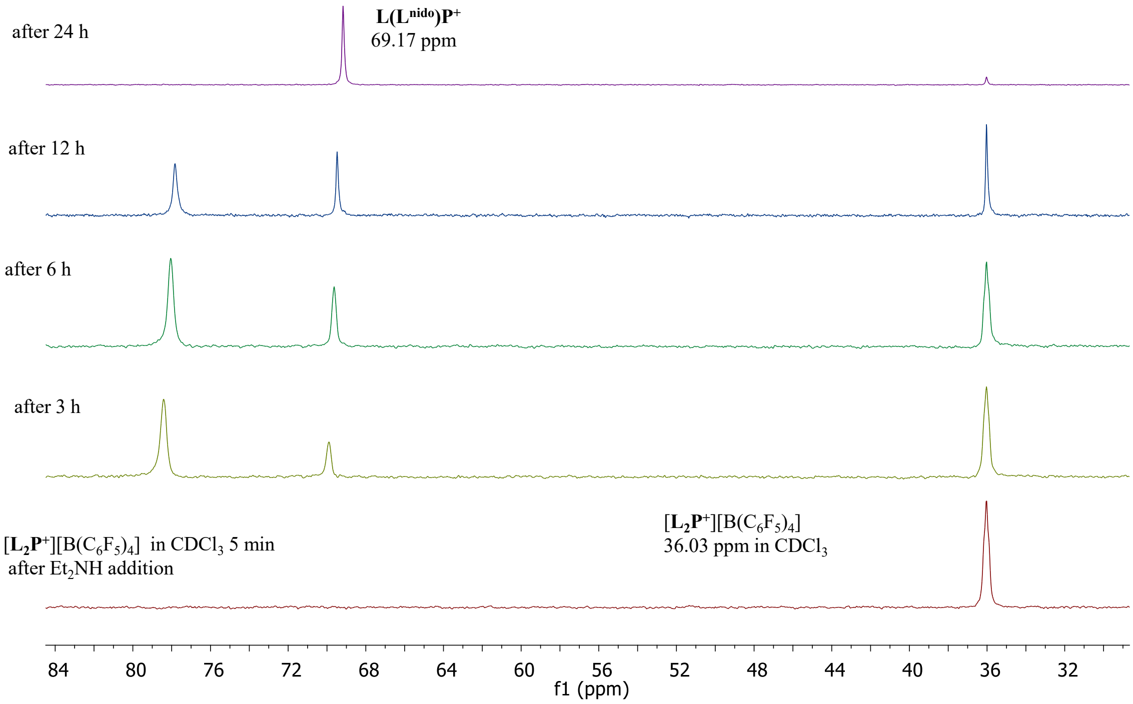

Figure S45. Stacked ^{31}P NMR spectra showing the progress of the reaction between [$\mathbf{L_2P^+}$][$B(C_6F_5)_4$] and Et_2NH in $CDCl_3$ at 85 °C.

Similar results were also obtained with other amines, such as 2,4,6-trimethylaniline, isopropylamine, and aniline required heating at 85 °C for 24 h. Notably, the reaction with diethylamine (Et_2NH) proceeded rapidly at room temperature, reaching completion in 24 h resulting in the slowly formation of $\mathbf{L(L^{nido})P^+}$.

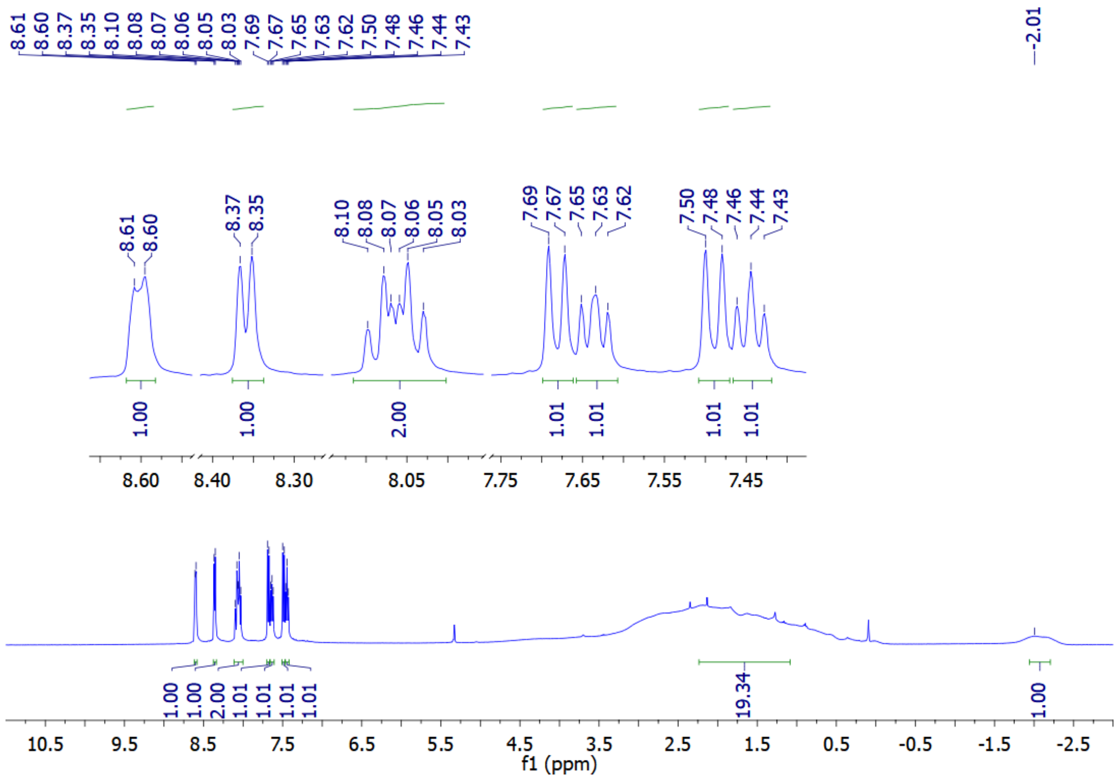

Figure S46. ^{1}H NMR spectrum (400 MHz, CD_2Cl_2) of **L(L^{nido})P^{+}**.

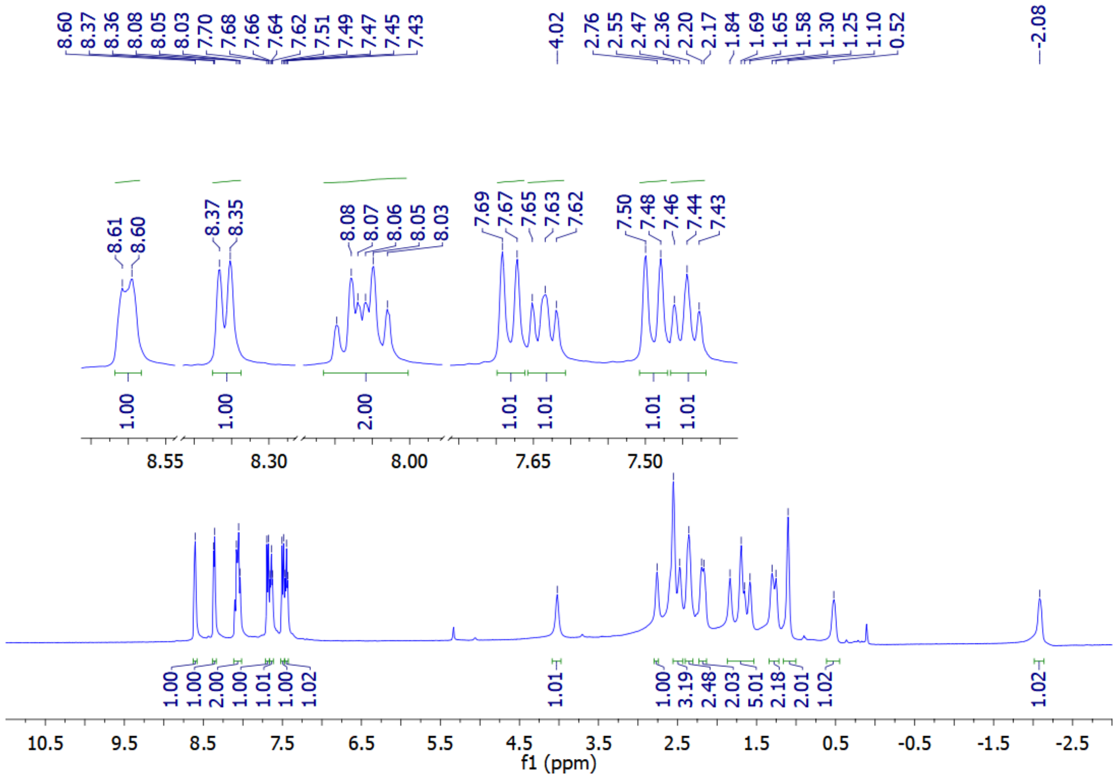

Figure S47. ^{1}H{^{11}B} NMR spectrum (400 MHz, CD_2Cl_2) of **L(L^{nido})P^{+}**.

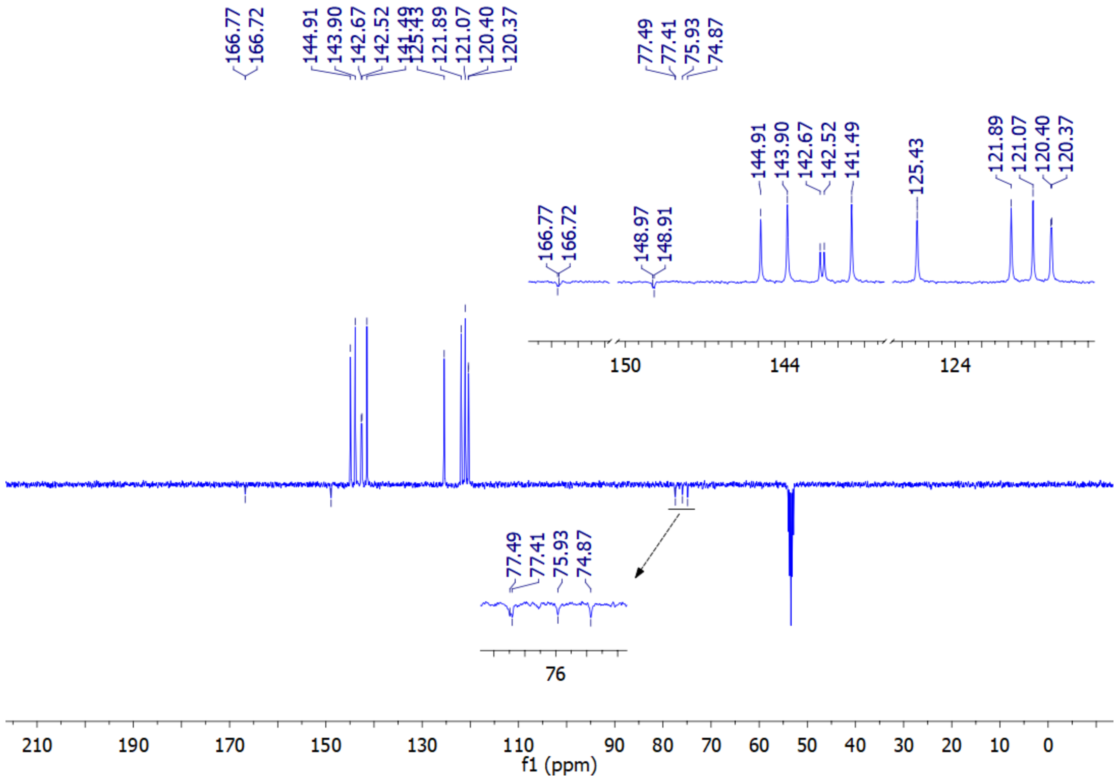

Figure S48. ${}^{13}C\{{}^{1}H\}$-JMOD NMR spectrum (100 MHz, CD_2Cl_2) of **L(L^{nido})P$^+$**.

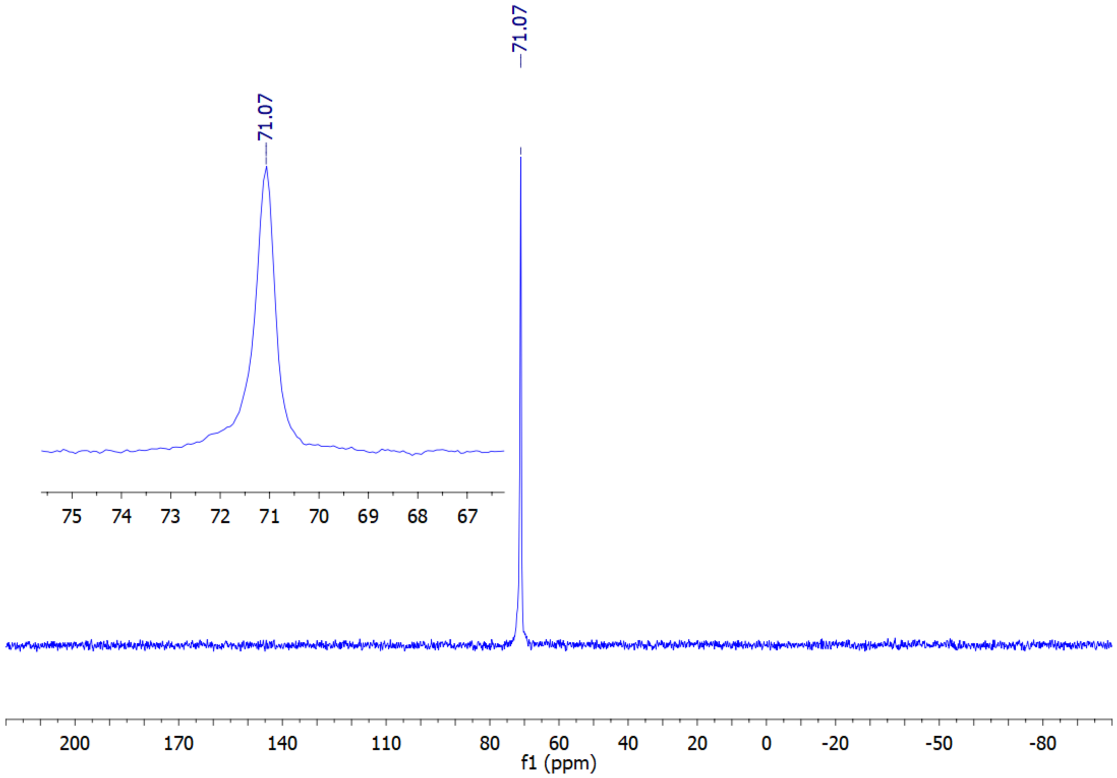

Figure S49. ${}^{31}P$ NMR spectrum (162 MHz, CD_2Cl_2) of **L(L^{nido})P$^+$**.

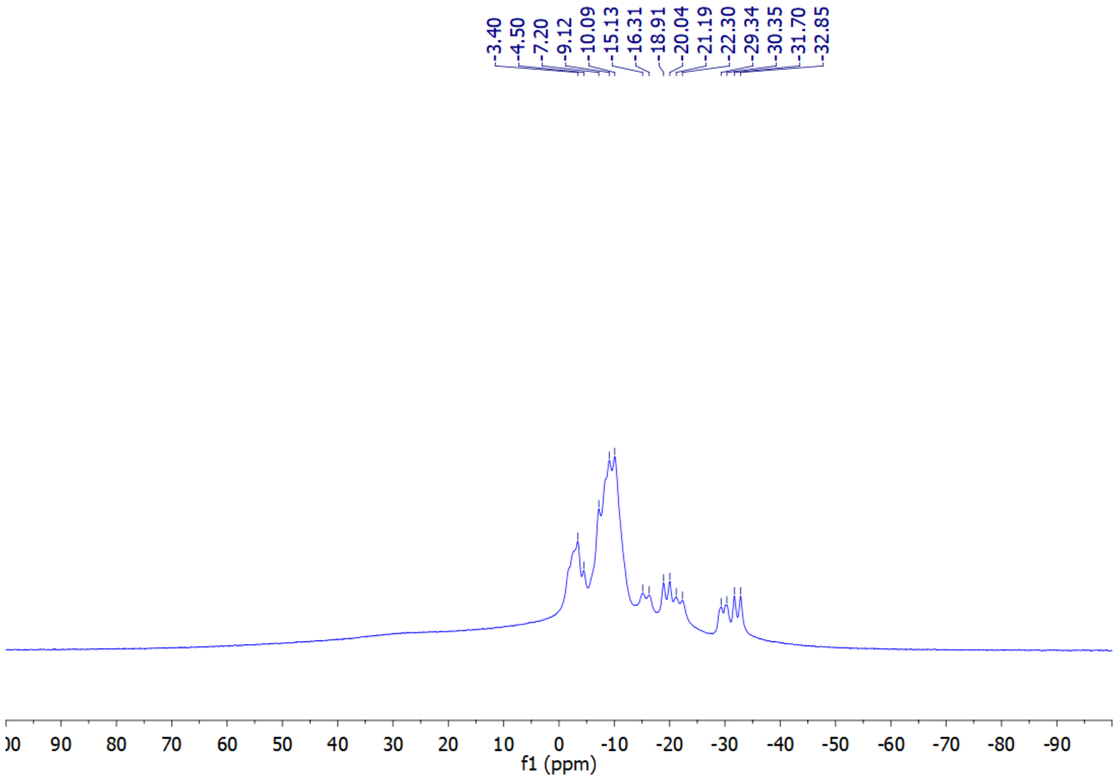

Figure S50. ^{11}B NMR spectrum (128 MHz, CD_2Cl_2) of **L(L^{nido})P$^+$**.

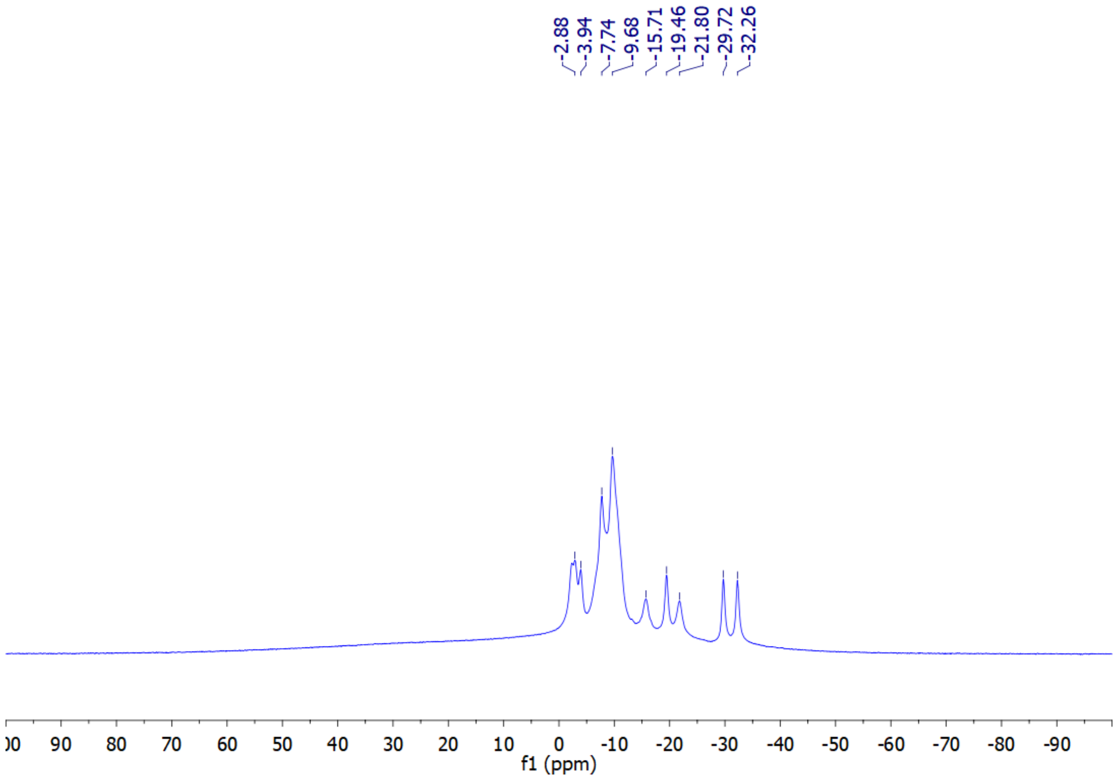

Figure S51. $^{11}B\{^1H\}$ NMR spectrum (128 MHz, CD_2Cl_2) of **L(L^{nido})P$^+$**.

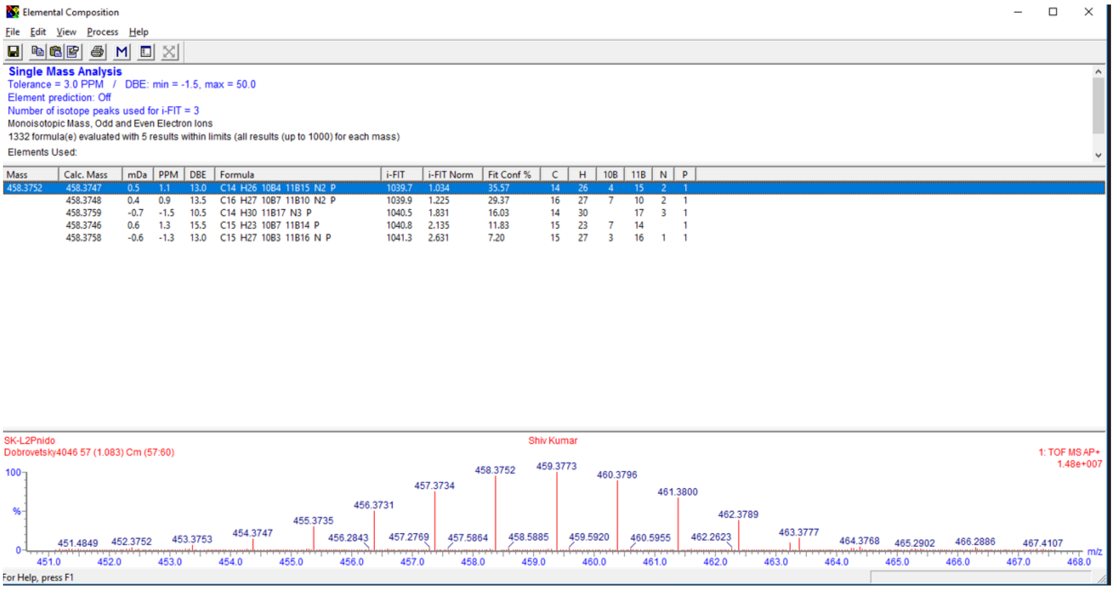

Figure S52. HRMS spectrum of $\mathbf{L(L^{nido})P^{+}}$.

1.8 Reaction of [L_2P^+][$B(C_6F_5)_4$] **with SO_2:**

A solution of [$\mathbf{L_2P^+}$][$B(C_6F_5)_4$] (50 mg, 0.043 mmol) was prepared in 0.7 mL of CH_3CN and transferred into a J-Young NMR tube under an inert atmosphere. The tube was degassed two times using freeze-pump-thaw cycles. While the tube was immersed in liquid nitrogen, SO_2 was added. The sealed tube was then placed in an oil bath and heated at 100 °C for 48 hours.

The reaction progress was monitored by ^{31}P NMR spectroscopy. After 3 hours, in addition to the signal corresponding to $\mathbf{L_2P^+}$ (at 31 ppm in CD_3CN), a new signal appeared at $\delta = -1.9$ ppm, possibly assigned to an adduct formed between SO_2 and the boron center of the *o*-Cb cluster. After 6 h, another signal appeared at 26 ppm, which, after 48 h, was the only signal in ^{31}P NMR. After 5 days, crystals were formed from the reaction mixture. The SC-XRD of these crystals showed the molecular structure as a nido-zwitterionic phosphonium sulphide ($\mathbf{L(L^{nido})P^+{=}S}$).

In the x-ray structure of $\mathbf{L(L^{nido})P^+{=}S}$, one boron center (of closo cluster) is primarily located on the closo cluster (0.715 occupancy) and partially on the nido cluster (0.285 occupancy). The disorder was modeled by splitting the boron atom over two positions, with positional and site-occupancy restraints applied to maintain chemically reasonable B–B distances. Anisotropic displacement parameters of the low-occupancy atoms were restrained. This model provides a chemically consistent description of the partial delocalization of the boron center between the nido and closo clusters.

Due to the poor solubility of compound $\mathbf{L(L^{nido})P^+{=}S}$ in acetonitrile, its NMR spectra were recorded in DMSO-d_6. However, decomposition occurred in DMSO as a result of the compound's instability in this solvent.

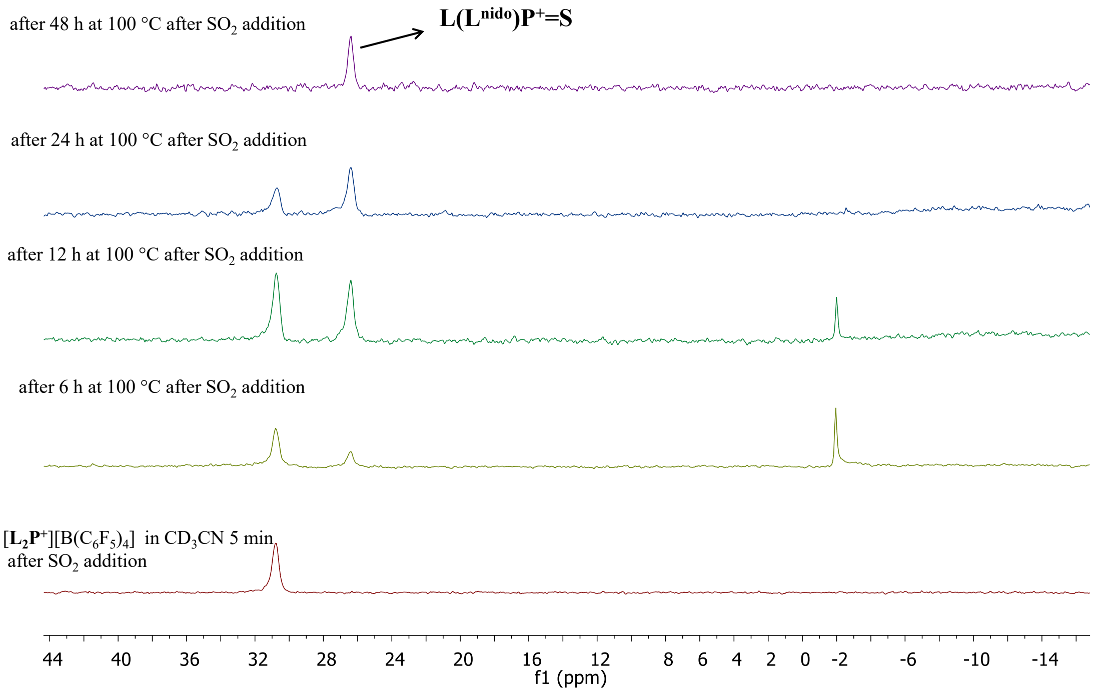

Figure S53. Stacked ^{31}P NMR spectra showing the progress of the reaction between [$\mathbf{L_2P^+}$][$B(C_6F_5)_4$] and SO_2 in CH_3CN at 100 °C over time.

To investigate the reactivity of $\mathbf{L_2P^+}$ with SO_2, we performed two control experiments: (i) the reaction of isolated **L(L^{nido})P$^+$** with SO_2 in CH_2Cl_2 (Figure S54), where isolated crystals of **L(L^{nido})P$^+$** were dissolved in CH_2Cl_2 and exposed to SO_2; and (ii) the in situ generation of **L(L^{nido})P$^+$** from $\mathbf{L_2P^+}$ and MeCN, followed by SO_2 addition (Figure S55), in which a solution of [$\mathbf{L_2P^+}$][$B(C_6F_5)_4$] in MeCN was prepared in a J-Young NMR tube, monitored until complete conversion to **L(L^{nido})P$^+$**, and then treated with SO_2 gas. In both cases, heating to 100 °C led to the formation of a complex mixture of products.

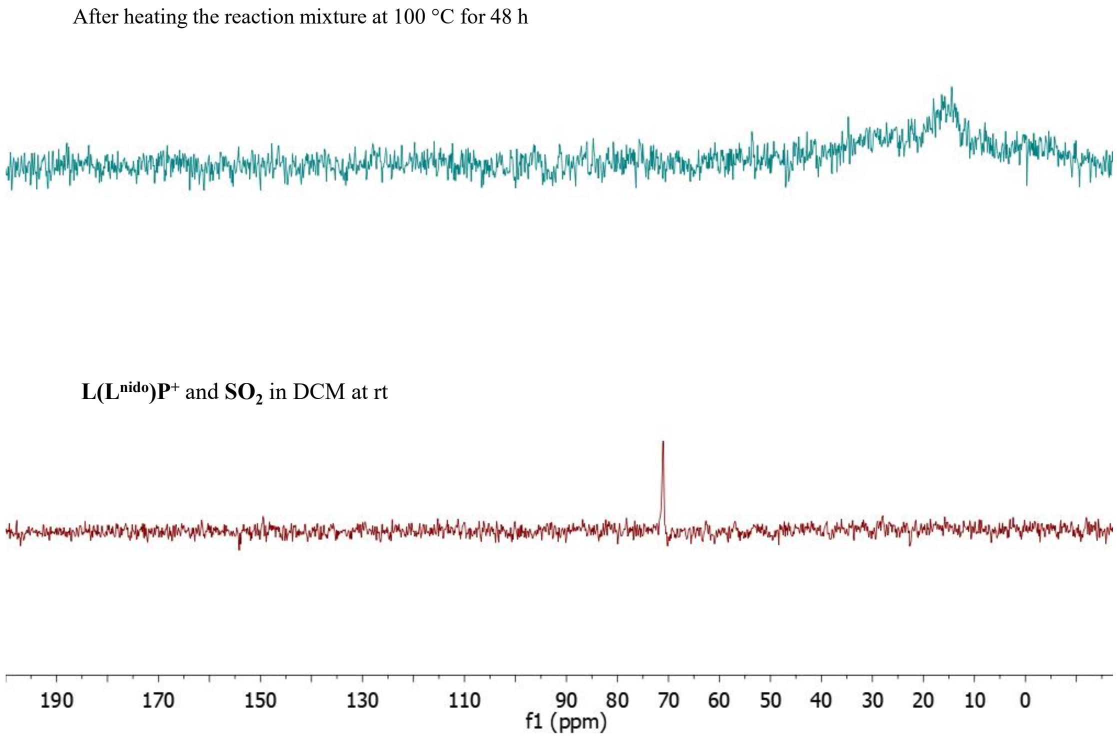

Figure S54. Stacked ^{31}P NMR spectra showing the progress of the reaction of isolated **L(L^{nido})P^{+}** with SO_2 in CH_2Cl_2 at 100 °C over time.

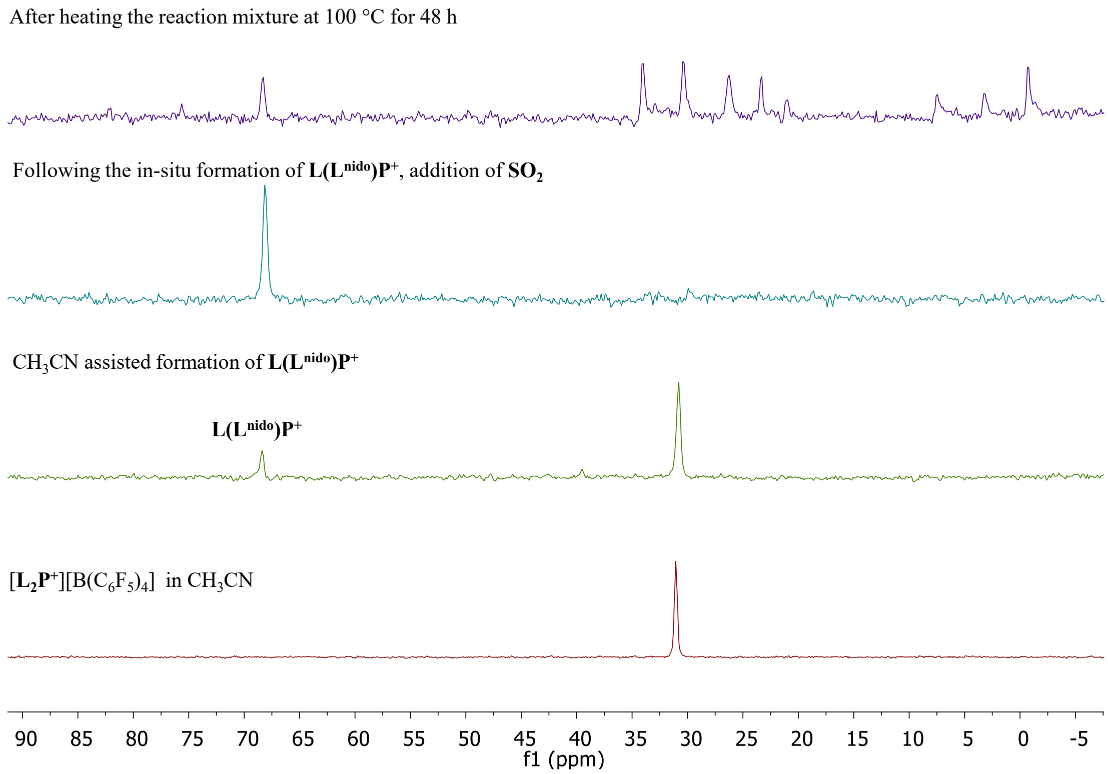

Figure S55. Stacked ^{31}P NMR spectra showing the progress of the in-situ formation of **L(L^{nido})P^{+}** from **L$_2$P^{+}** and MeCN, followed by the addition of SO_2 in CH_3CN at 100 °C over time.

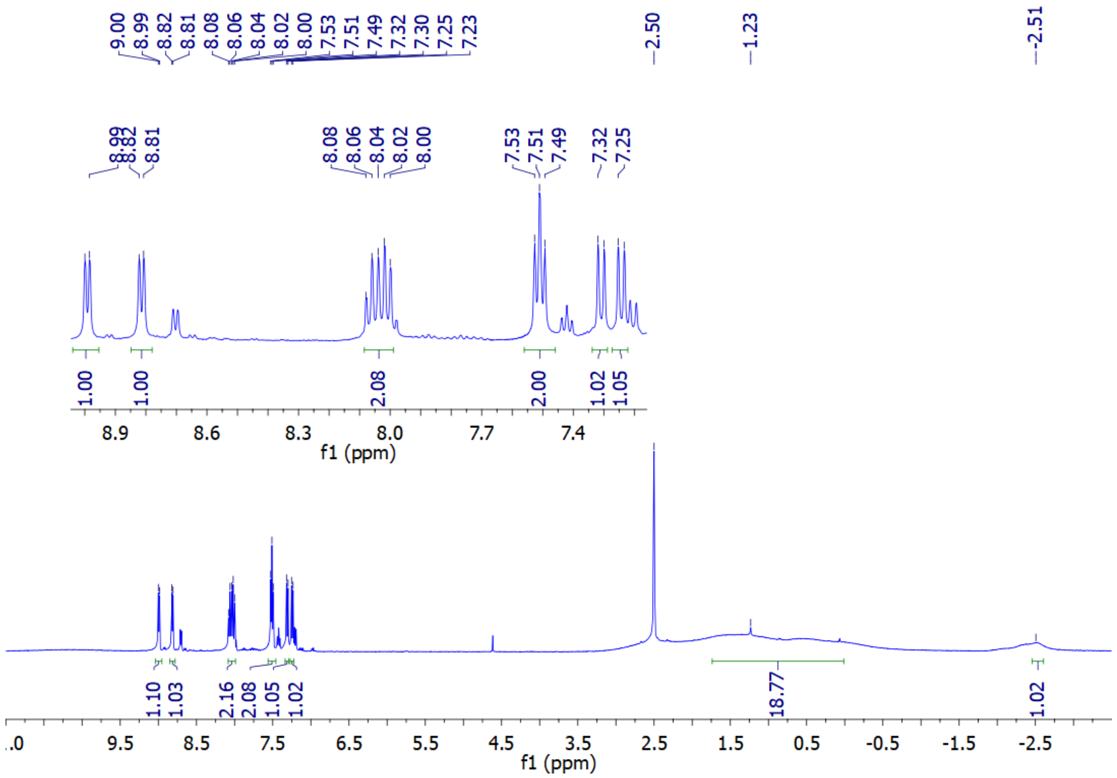

Figure S56. ^{1}H NMR spectrum (400 MHz, DMSO-d_6) of **L(L^{nido})P^{+}=S**.

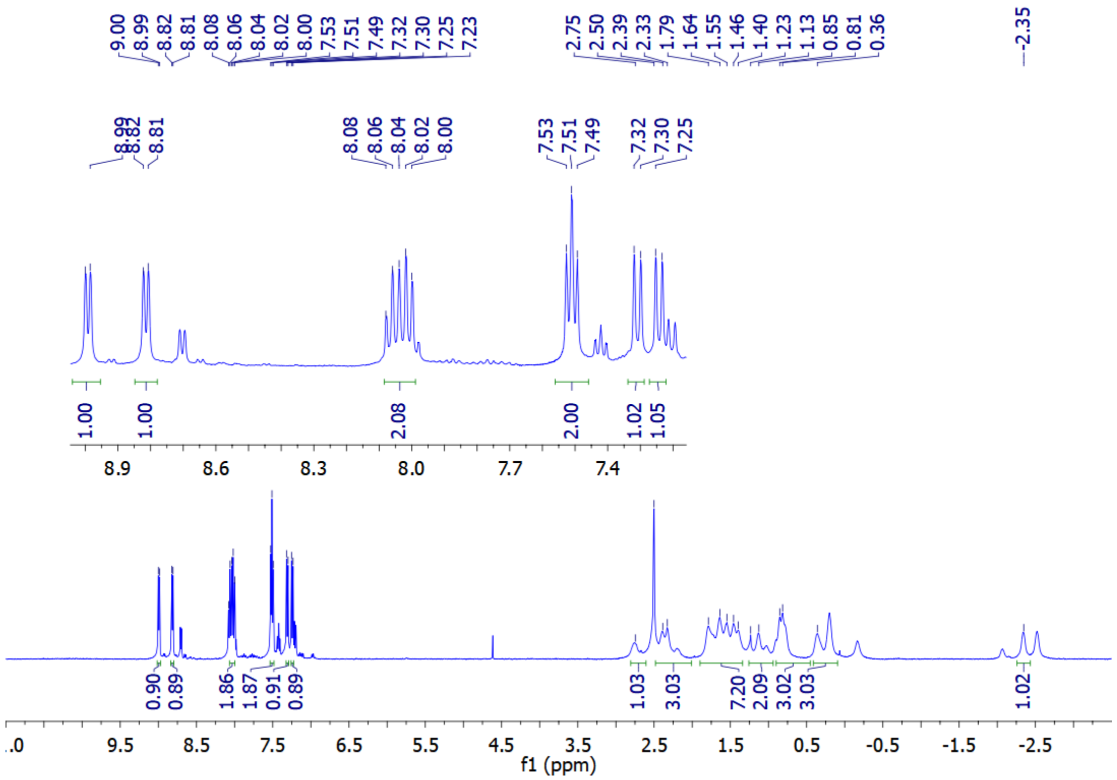

Figure S57. $^{1}H\{^{11}B\}$ NMR spectrum (400 MHz, DMSO-d_6) of **L(L^{nido})P^{+}=S**.

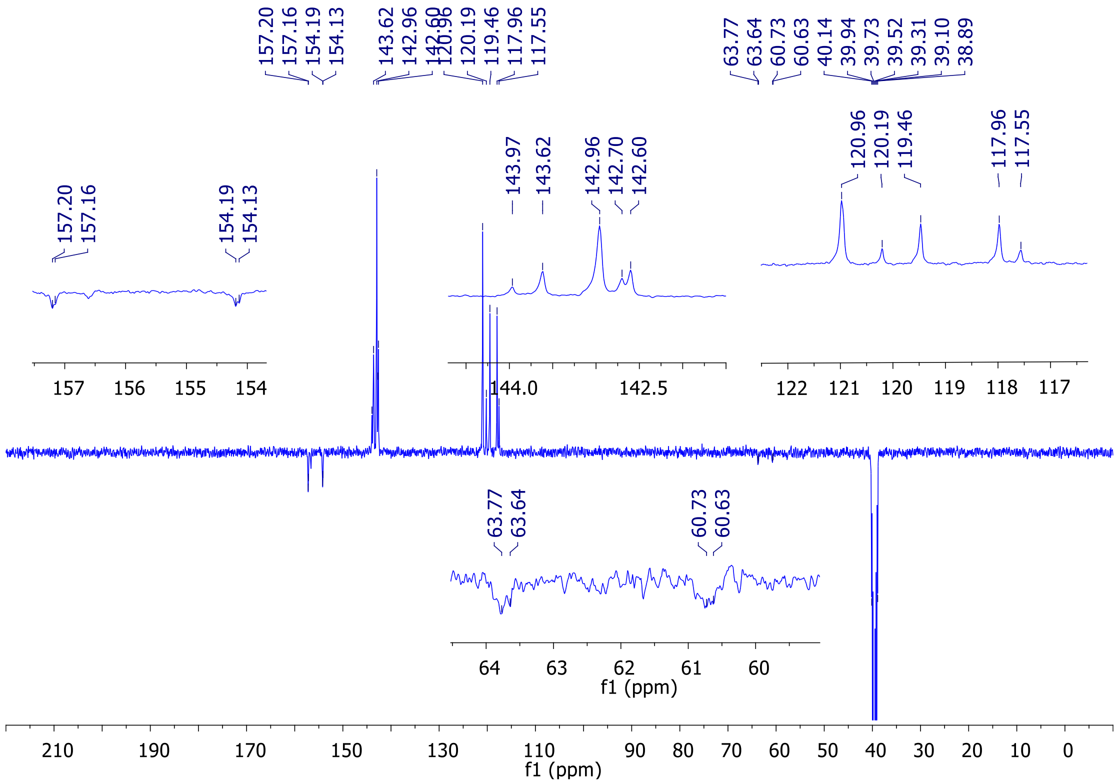

Figure S58. $^{13}C\{^{1}H\}$-JMOD NMR spectrum (100 MHz, DMSO-d_6) of **L(L^{nido})P^{+}=S**.

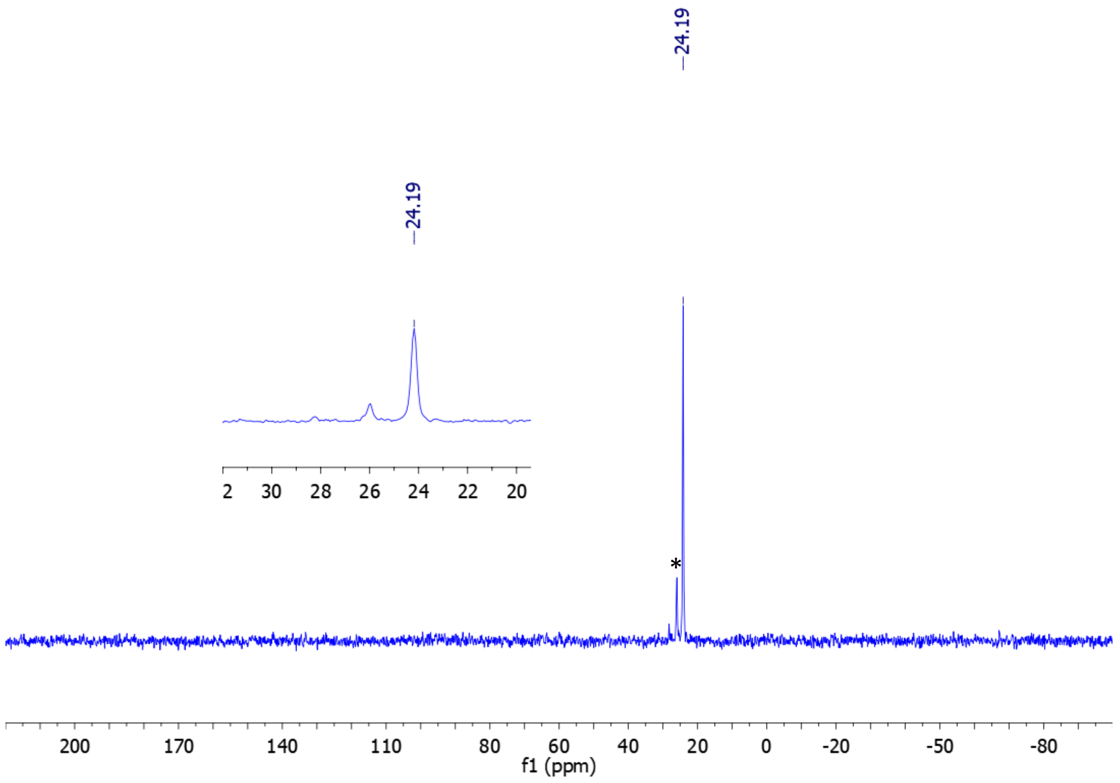

Figure S59. ^{31}P NMR spectrum (162 MHz, DMSO-d_6) of **L(L^{nido})P^{+}=S**. (* decomposition in DMSO-d_6)

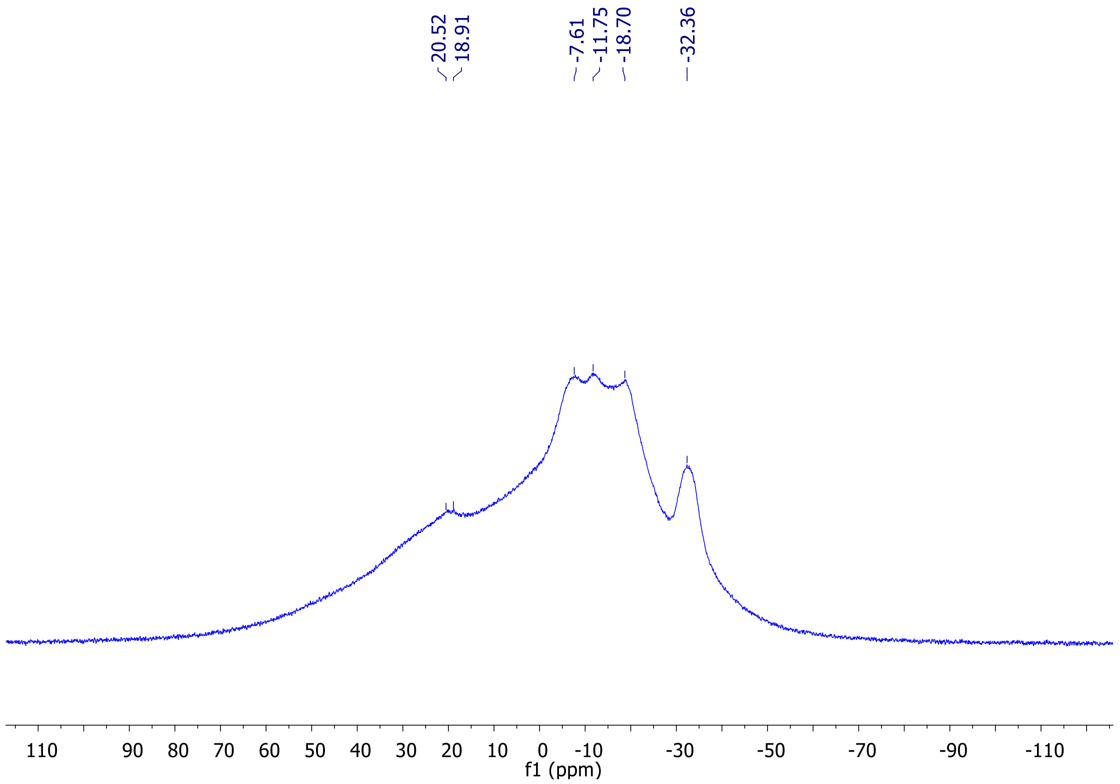

Figure S60 ^{11}B NMR spectrum (128 MHz, DMSO-d$_6$) of **L(L^{nido})P$^+$=S**.

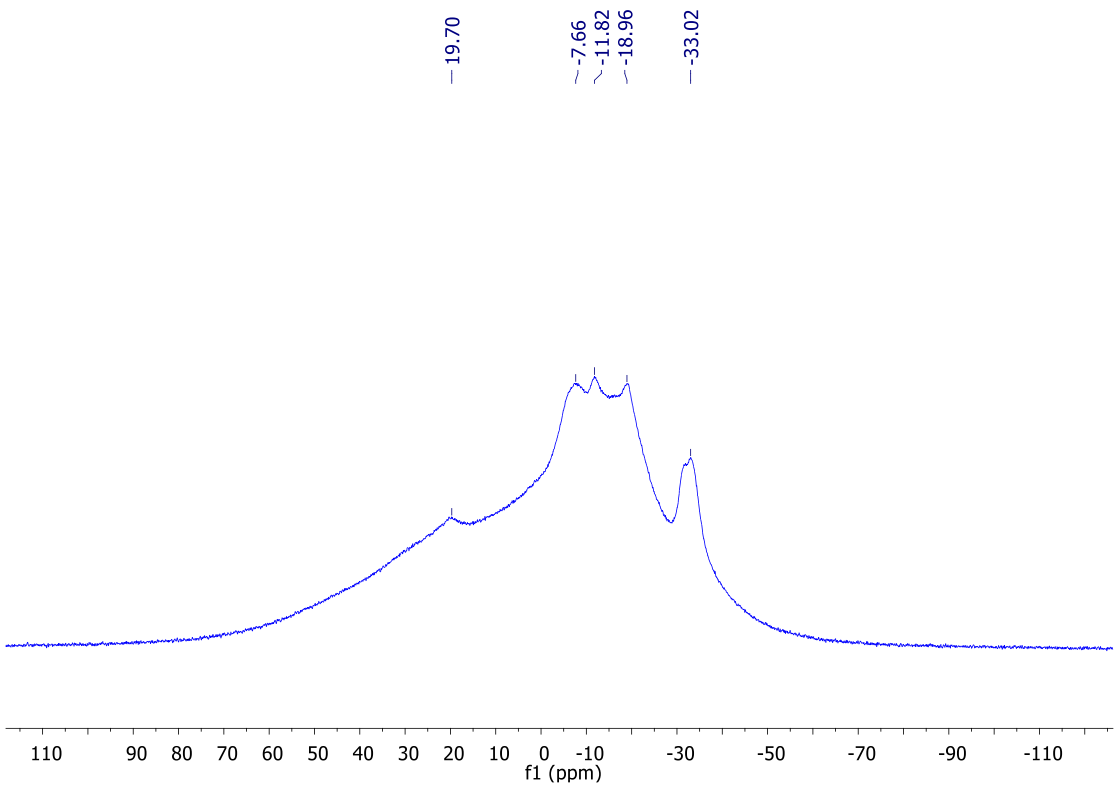

Figure S61. $^{11}B\{^1H\}$ NMR spectrum (128 MHz, DMSO-d$_6$) of **L(L^{nido})P$^+$=S**.

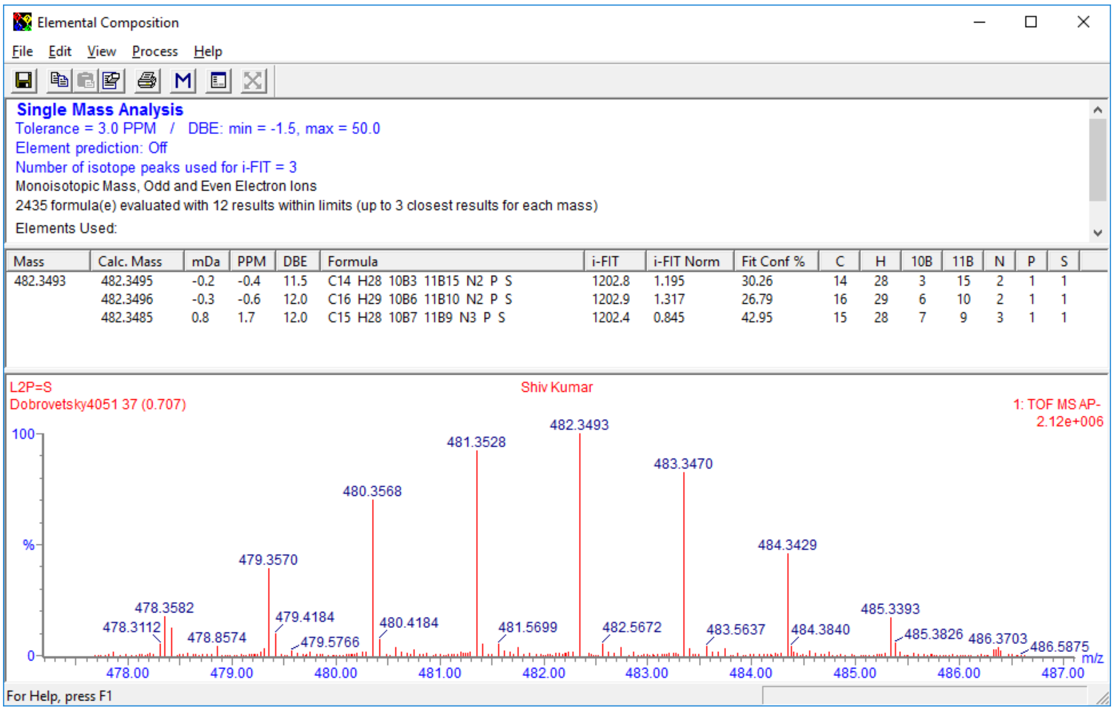

Figure S62. HRMS spectrum of **L(L^{nido})P^{+}=S**.

1.9 Reaction of [L$_2$P$^+$][B(C$_6$F$_5$)$_4$] with THF:

In a J-Young NMR tube, a solution of [**L$_2$P$^+$**][B(C$_6$F$_5$)$_4$] (15 mg, 0.013 mmol, 5 mol %) was prepared in 0.5 mL CDCl$_3$, followed by the addition of 22 μL (0.26 mmol) of THF. An immediate reaction was observed, and the mixture was stirred for 30 minutes to ensure complete consumption of THF. All volatiles were then removed under reduced pressure; the desired compound was extracted with n-hexane. Drying the resulting solution under vacuum afforded an off white solid. The NMR conversion was 99%.

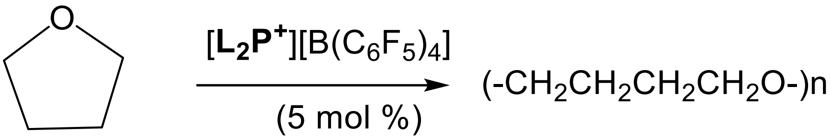

Scheme S9. Reactivity of [**L$_2$P$^+$**][B(C$_6$F$_5$)$_4$] with THF.

NMR:

^{1}H NMR (400 MHz, CDCl$_3$); δ = 3.39 (m), 1.60 (m).[5]

^{13}C{^{1}H} NMR (100 MHz, CDCl$_3$); δ = 70.6, 26.5.

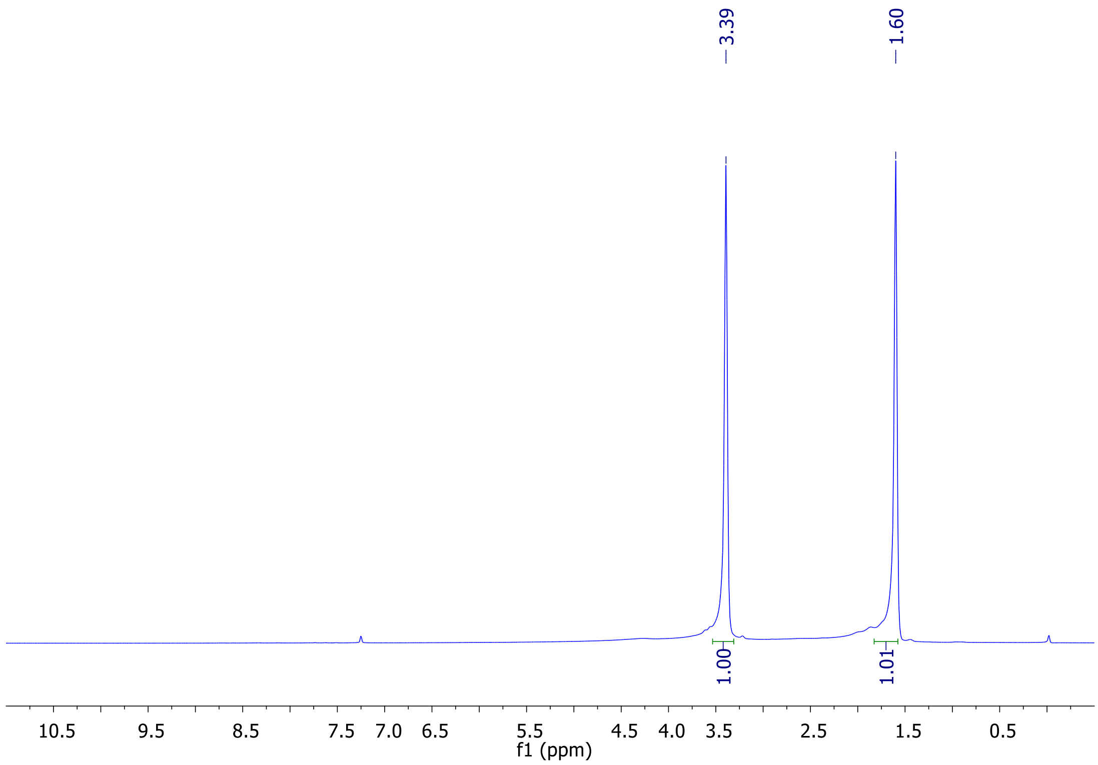

Figure S63. ^{1}H NMR spectrum (400 MHz, $CDCl_3$) of Poly(tetrahydrofuran).

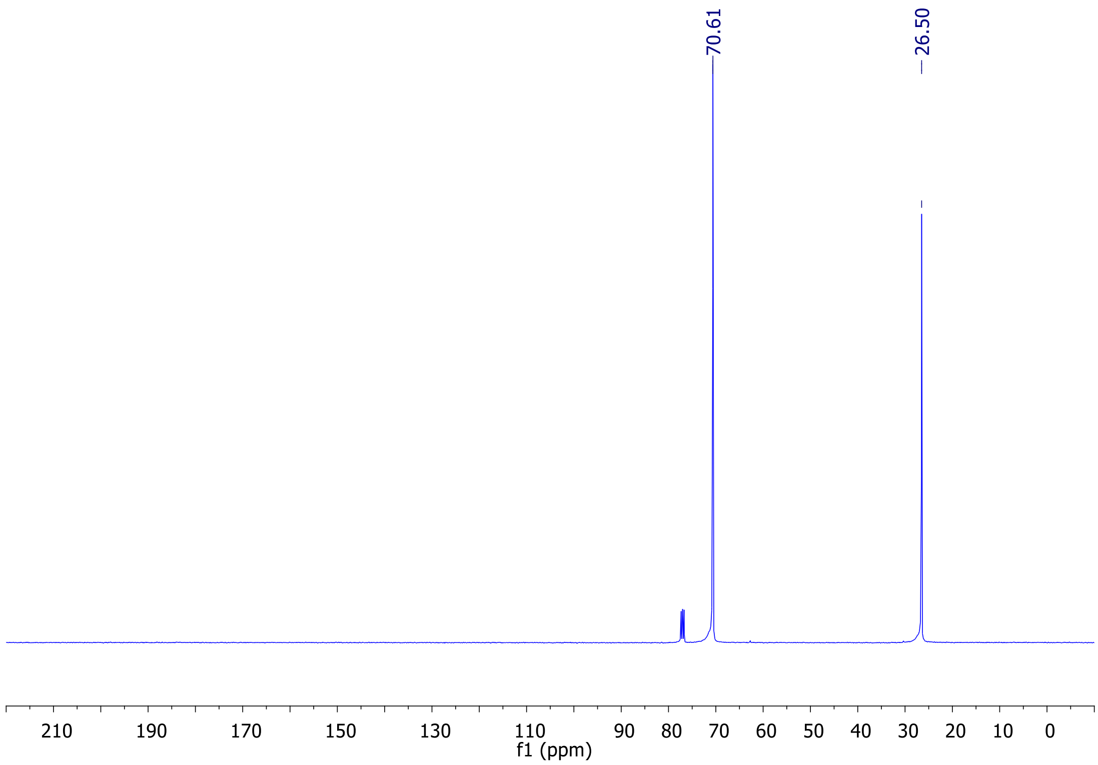

Figure S64. $^{13}C\{^{1}H\}$-JMOD NMR spectrum (100 MHz, $CDCl_3$) of Poly(tetrahydrofuran).

1.10 Reaction of $[L_2P^+][B(C_6F_5)_4]$ with 1,1-Diphenylethylene:

In a J-Young NMR tube, solution of [$\mathbf{L_2P^+}$][$B(C_6F_5)_4$] (15 mg, 0.013 mmol, 5 mol %) was prepared in 0.5 mL $CDCl_3$. To this solution, 46 µL (0.26 mmol) of 1,1-diphenylethylene was added. The reaction progress was monitored by ^{1}H NMR spectroscopy, and complete consumption of 1,1-diphenylethylene was observed after 12 hours. Subsequently, all volatiles were removed under reduced pressure, and the residue was extracted with n-hexane. The resulting solution was dried under vacuum to yield a colorless oil, identified as 1-Methyl-1,3,3-triphenyl-2,3-dihydro-1H-indene. [6] The NMR conversion was determined to be 99%, based on the residual amount of starting material in the reaction mixture.

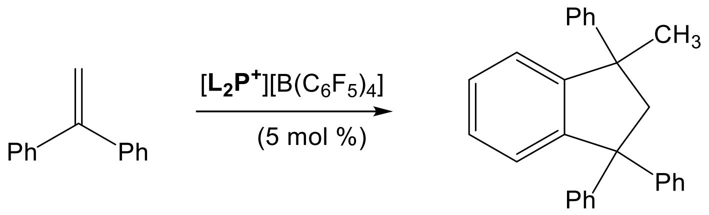

Scheme S10. Reactivity of [$\mathbf{L_2P^+}$][$B(C_6F_5)_4$] with 1,1-diphenylethylene.

NMR:

^{1}**H NMR** (400 MHz, $CDCl_3$); δ = 7.34-7.05 (m, 19H), 3.44 (d, J = 13.5 Hz, 1H), 3.15 (d, J = 13.5 Hz, 1H), 1.59 (s, 3H).

^{13}**C{^{1}H} NMR** (100 MHz, $CDCl_3$); δ = 150.52, 149.33, 148.84, 148.50, 147.47, 128.76, 128.66, 127.93, 127.84, 127.65, 127.57, 127.42, 127.34, 126.87, 126.79, (m) 125.97, 125.64, 125.57, 125.01, 61.34, 60.92, 51.17, 28.82.

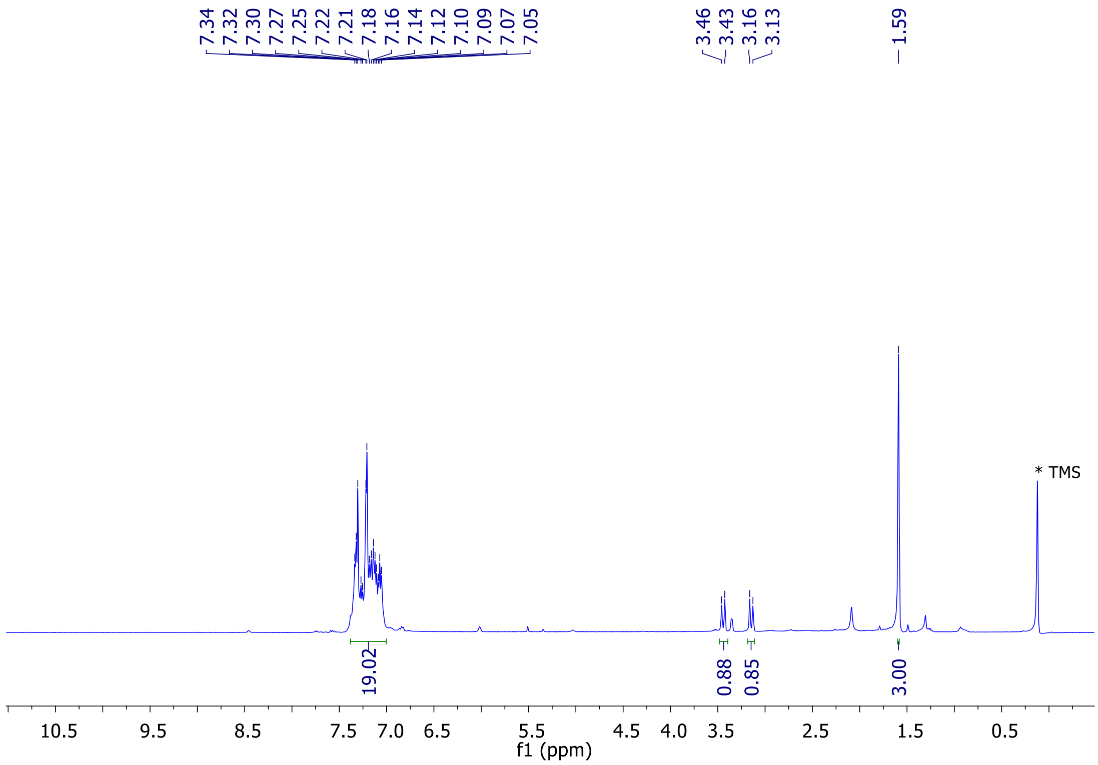

Figure S65. ^{1}H NMR spectrum (400 MHz, $CDCl_3$) of Methyl-1,3,3-triphenyl-2,3-dihydro-1H-indene.

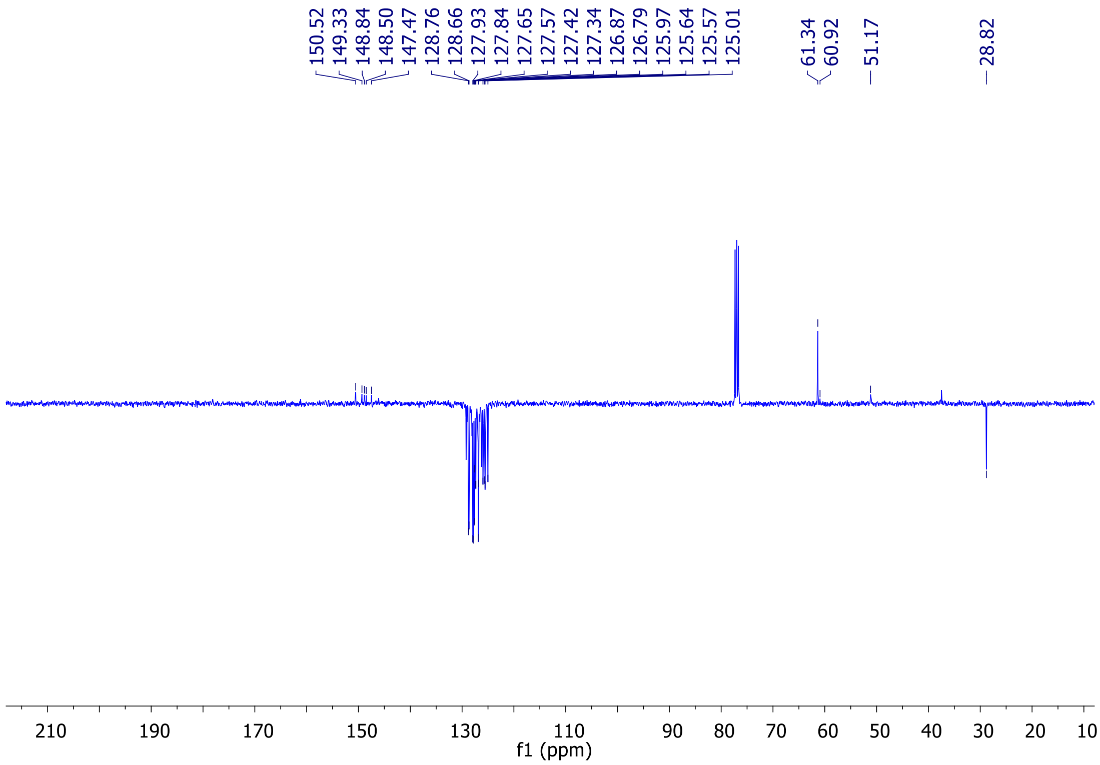

Figure S66. $^{13}C\{^{1}H\}$-JMOD NMR spectrum (100 MHz, $CDCl_3$) of -Methyl-1,3,3-triphenyl-2,3-dihydro-1H-indene.

1.11 $[L_2P^+][B(C_6F_5)_4]$ Catalyzed Cyanosilylation of Benzaldehyde:

In a J-Young NMR tube, a solution of [$\mathbf{L_2P^+}$][$B(C_6F_5)_4$] (15 mg, 0.013 mmol, 5 mol %) was prepared in 0.5 mL of $CDCl_3$. Benzaldehyde (26.4 µL, 0.26 mmol) and trimethylsilyl cyanide (TMSCN, 32.5 µL, 0.26 mmol) were then added to this solution. Immediate ^{1}H NMR monitoring showed the emergence of a new resonance at 5.6 ppm, consistent with the formation of the cyanosilylated product 2-Phenyl-2-((trimethylsilyl)oxy)acetonitrile. The mixture was subsequently stirred for 1 h to ensure completion of reaction. After 1 h, ^{1}H and ^{13}C NMR spectra were recorded, which matched the reported values[7], confirming successful cyanosilylation of the aldehyde. The NMR conversion was determined to be 78%, based on the residual amount of starting material in the reaction mixture.

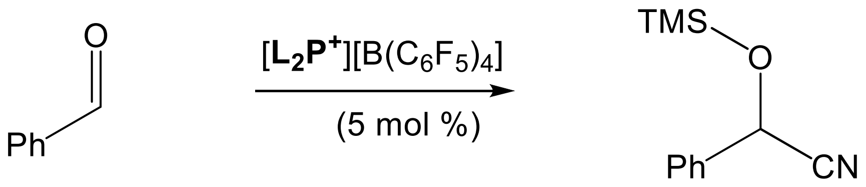

Scheme S11. [$\mathbf{L_2P^+}$][$B(C_6F_5)_4$] catalyzed cyanosilylation of benzaldehyde.

NMR: ^{1}H NMR (400 MHz, $CDCl_3$); δ = 7.51−7.55 (m, 2 H, Ar), 7.45−7.47 (m, 3 H, Ar), 5.56 (s, 1 H, CH), 0.28 (s, 9 H, $SiMe_3$), ppm.

^{13}C{^{1}H} NMR (100 MHz, $CDCl_3$); δ = 136.15, 129.32, 128.90, 126.26, 119.22, 63.61, –0.40 ppm.

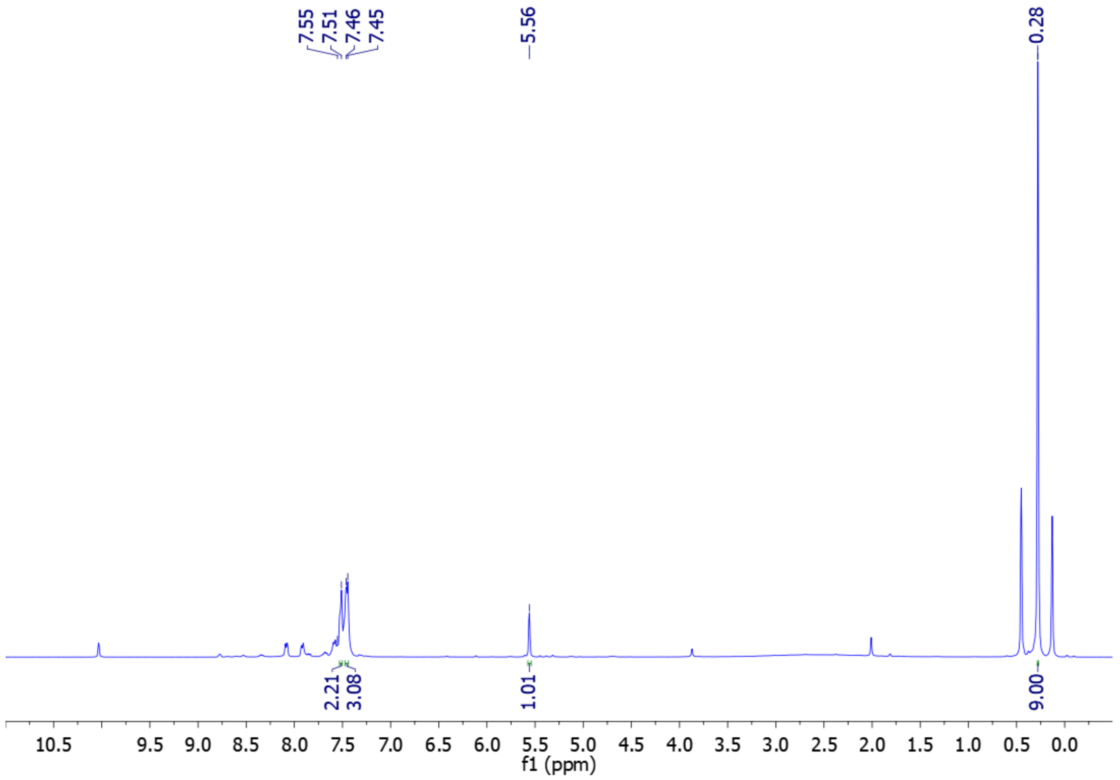

Figure S67. ^{1}H NMR spectrum (400 MHz, $CDCl_3$) of 2-Phenyl-2-((trimethylsilyl)oxy)acetonitrile.

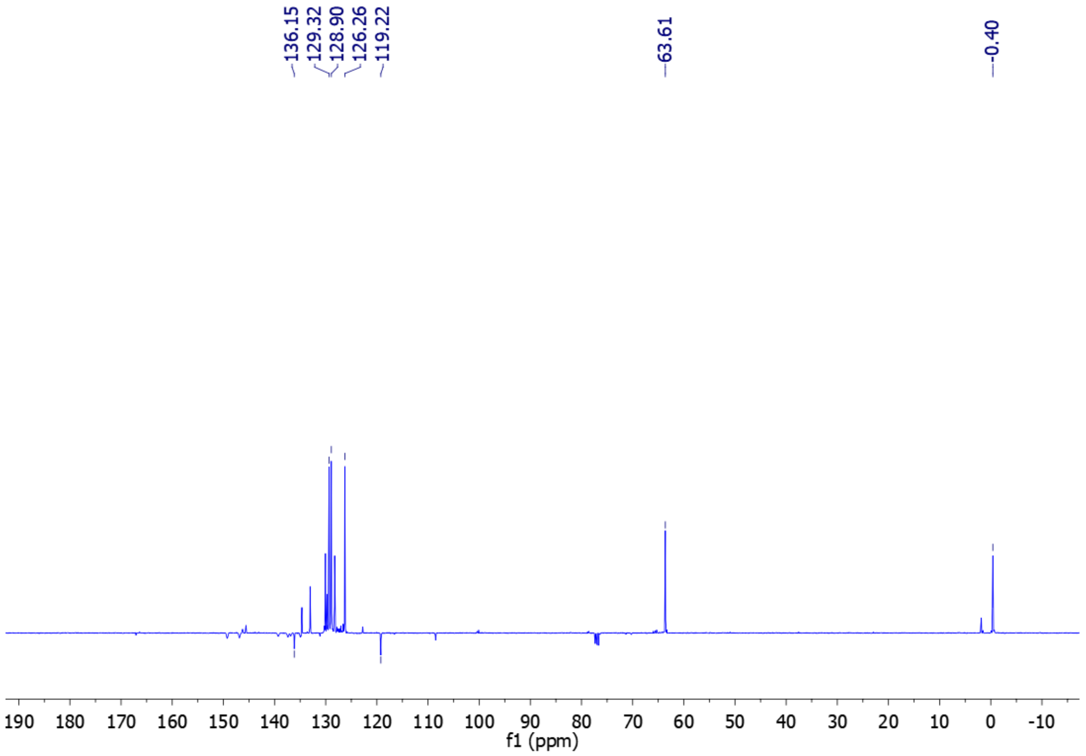

Figure S68. $^{13}C\{^{1}H\}$-JMOD NMR spectrum (100 MHz, $CDCl_3$) of 2-Phenyl-2-((trimethylsilyl)oxy)acetonitrile.

1.12 Reaction of Ph_2P(*o*Cb) with H_2O:

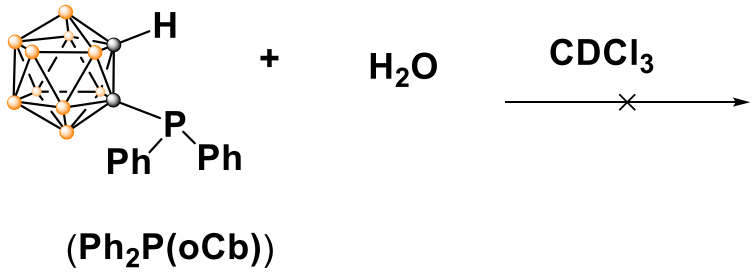

Scheme S12. Reaction of **Ph_2P(*o*Cb)** with H_2O.

The observed reactivity of [**L_2P^+**][$B(C_6F_5)_4$] with O-H containing molecules prompted us to check whether such reactivity is associated with the *o*Cb-based system in general. Therefore, we treated **Ph_2P(*o*Cb)** with 5equiv of H_2O in $CDCl_3$. Initially, the reaction was monitored by ^{31}P NMR spectroscopy at r.t. for 24 h, and no change was observed. Further, it was monitored at 85 °C for 24 h; no reaction was observed in this case as well.

NMR:

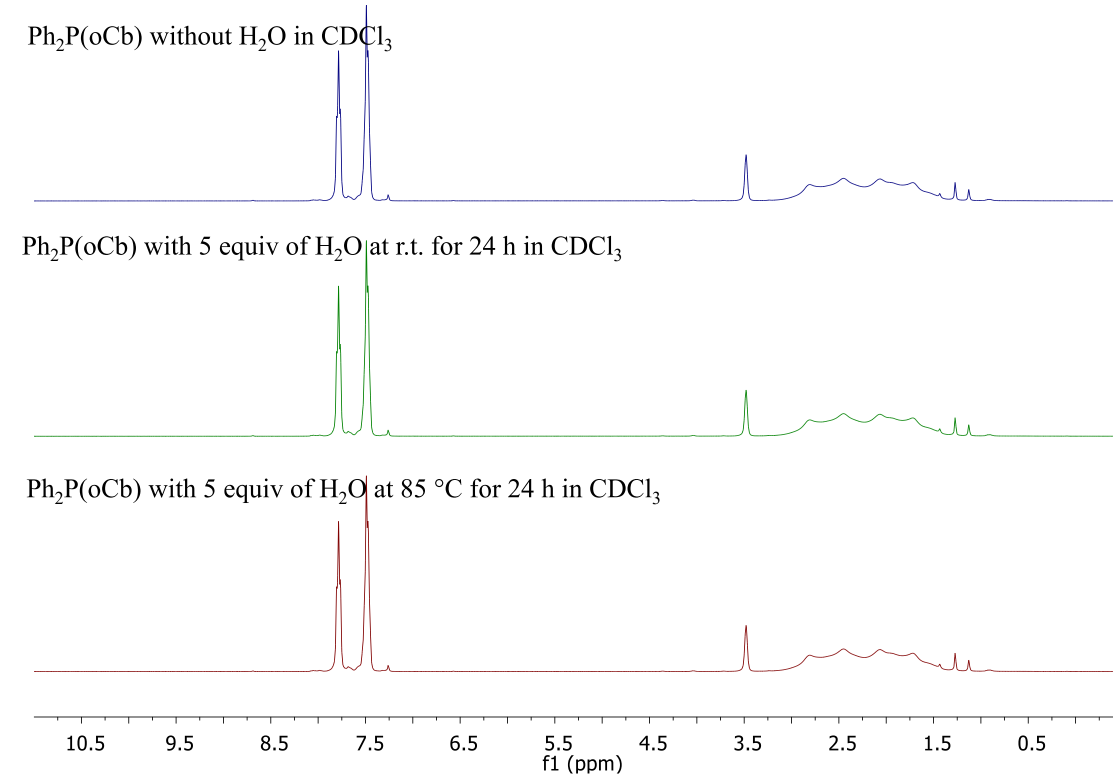

Figure S69. ${}^{1}H$ NMR stack plot for reaction of $Ph_2P(o\text{Cb})$ with H_2O.

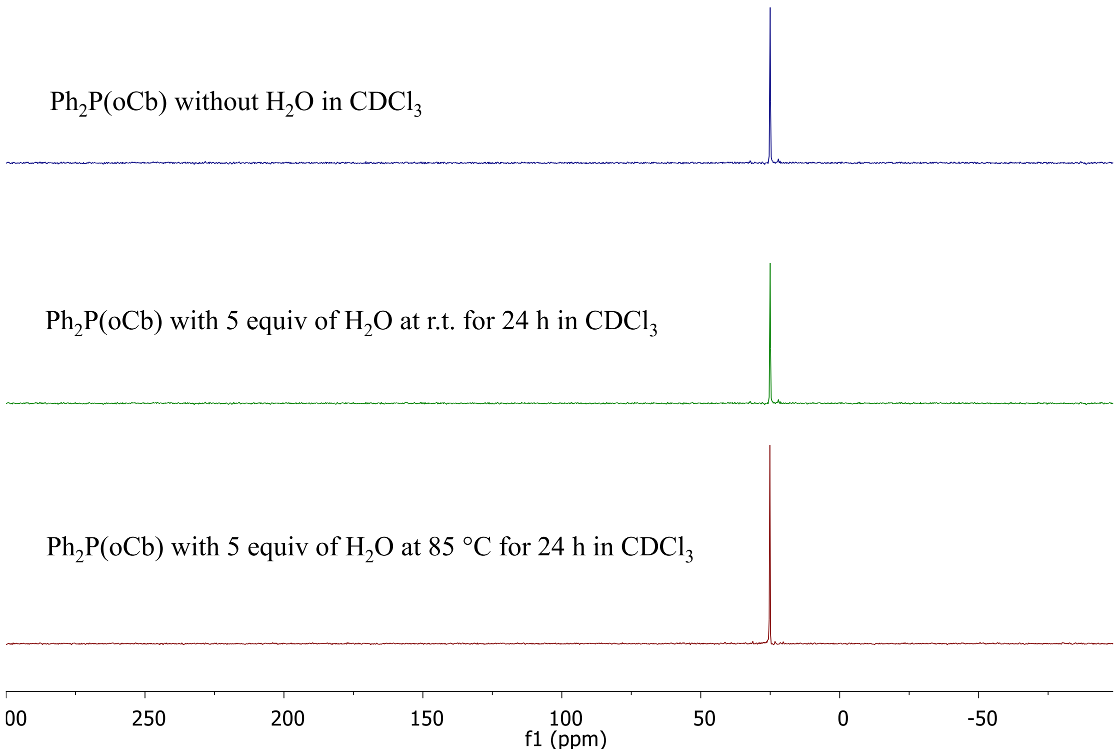

Figure S70. ${}^{31}P$ NMR stack plot for reaction of $Ph_2P(o\text{Cb})$ with H_2O.

3.13 Decapitation of $[L_2P^+][B(C_6F_5)_4]$ with amines.

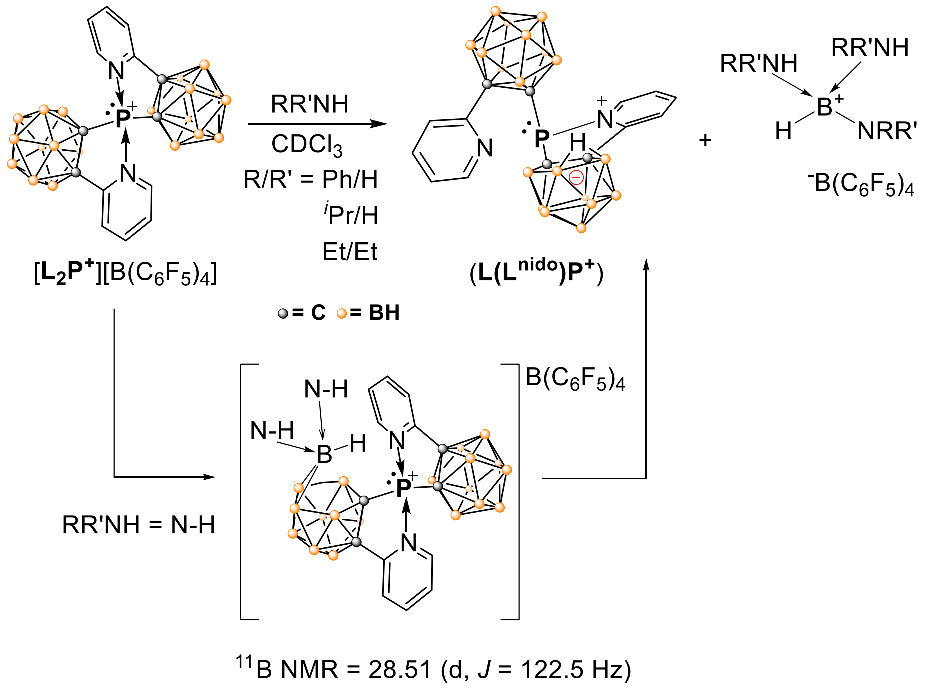

Scheme S13. Mechanism for decapitation of [$\mathbf{L_2P^+}$][$B(C_6F_5)_4$].

The reaction of [$\mathbf{L_2P^+}$][$B(C_6F_5)_4$] with amines produces $\mathbf{L(L^{nido})P^+}$ and the decapitated boron fragments. The possible mechanism for the deboronation is shown below (Scheme S13). The B3 center in these $\mathbf{L_2P^+}$ is highly Lewis acidic, and thus initially forms an adduct with amines, which resonates at 78 ppm in the ^{31}P NMR and a corresponding doublet at 28.15 ppm (d, *J(BH)* = 122 Hz) in the ^{11}B NMR spectrum (Figure S71).[8] The decapitated boron species itself proved to be unstable; attempts to isolate this intermediate were unsuccessful, as it possibly gradually converted into the corresponding boronium species.[9] The boronium species itself proved to be unstable as well and underwent further decomposition, thereby precluding its comprehensive characterization.

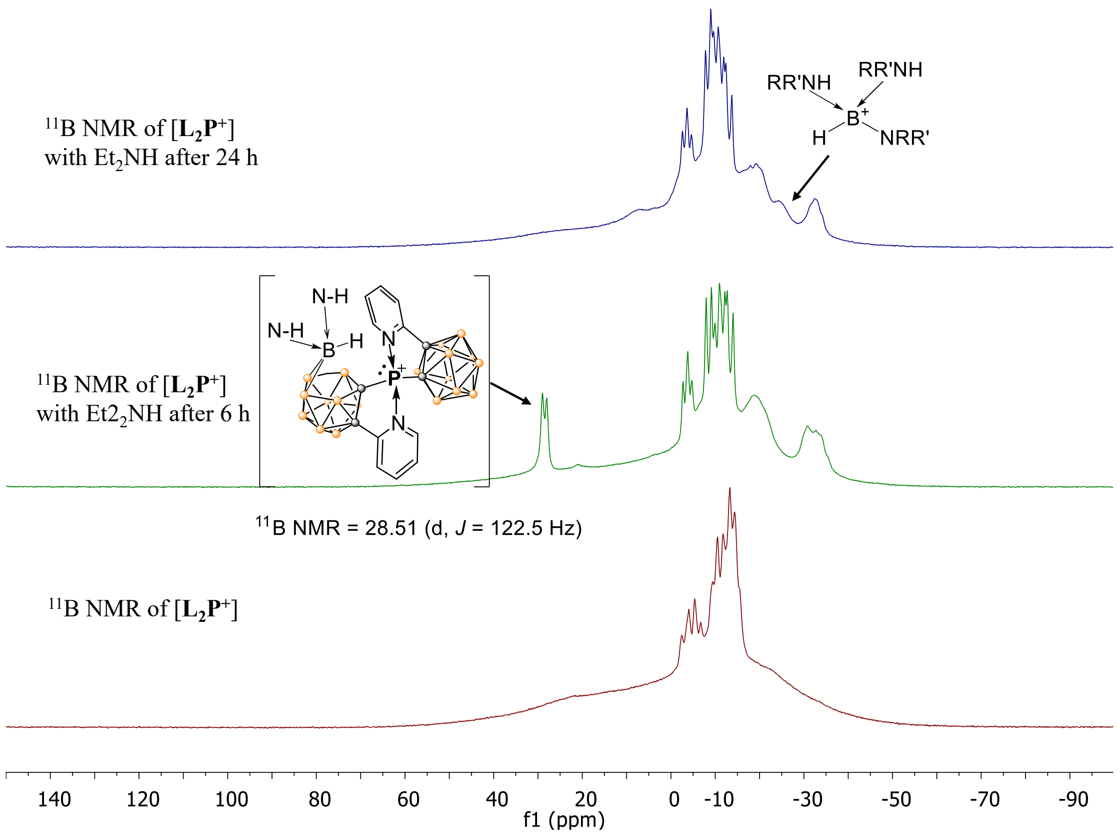

Figure S71. ^{11}B NMR stack plot for deborylation of $\mathbf{L_2P^+}$.

3.14 X-Ray crystallographic tables

Table 1. Crystallographic data for compound **$LPCl_2$**, **LL'P**, **L_2P^+**, **$L(L^{nido})P$+** and **$L(L^{nido})P^+$=S**.

Compound	$LPCl_2$	LL'P	L_2P^+	$L(L^{nido})P^+$	$L(L^{nido})P^+$=S	LH_2^+
**Chemical formula**	$C_7H_{14}B_{10}Cl_2NP$	$C_{28}H_{50}B_{30}N_3P$	$C_{63}H_{29}B_{22}Cl_3F_{40}KN_2P$	$C_{20}H_{34}B_{19}N_2P$	$C_{14}H_{28}B_{19}N_2PS$	$C_{34}H_{19}B_{11}F_{20}N$
**Molar mass**	322.16	783.98	1988.12	538.85	492.80	940.41
**Crystal system**	monoclinic	orthorhombic	triclinic	monoclinic	triclinic	triclinic
**Space group**	$P2_1/c$	$P2_12_12_1$	$P\bar{1}$	$P2_1/n$	$P\bar{1}$	$P\bar{1}$
T **[K]**	100(2)	110(2)	100(2)	100(2)	100(2)	100(2)
a **[Å]**	7.1150(3)	11.4873(2)	14.6028(2)	13.4442(5)	7.6649(2)	10.2357(2)
b **[Å]**	15.7332(5)	13.9008(2)	15.6679(2)	14.3449(5)	12.5082(3)	14.1709(3)
c **[Å]**	13.5319(6)	27.3069(4)	19.2963(2)	15.3724(5)	13.7842(4)	14.4073(3)
α **[°]**	90	90	97.3707(10)	90	100.149(2)	105.591(2)
β **[°]**	91.911(4)	90	110.9652(12)	91.843(3)	103.060(2)	108.133(2)
γ **[°]**	90	90	106.5838(13)	90	94.905(2)	95.587(2)
V **[Å^3]**	1513.94(10)	4360.44(12)	3817.94(10)	2963.12(18)	1256.35(6)	1875.13(7)
Z	4	4	2	4	2	2
ρ **(calcd.) [g·cm^{-3}]**	1.413	1.194	1.729	1.208	1.303	1.666
μ**[mm^{-1}]**	0.513	0.094	3.083	0.112	0.205	1.457
**Reflections collected**	12640	65145	62297	21284	14923	28924
**Independent reflections**	3469	10822	16292	6875	5576	7987
**Data/restraints/ parameters**	3469/0/190	10822/0/549	16292/0/1189	6875/0/395	5576/0/367	7987/0/600
$R1, wR_2[I>2\sigma(I)]$[a]	0.0540, 0.1332	0.0409, 0.1006	0.0683, 0.1892	0.0441, 0.1125	0.0433, 0.1060	0.0402, 0.1062
**R1, wR$_2$ (all data)**[a]	0.0746, 0.1517	0.0452, 0.1042	0.0798, 0.1995	0.0570, 0.1191	0.0526, 0.1103	0.0427, 0.1082
**GOF**	1.047	1.048	1.030	1.025	1.044	1.062

[a] $R1 = \Sigma||Fo| - |Fc||/\Sigma|Fo|, wR_2 = [\Sigma w(|Fo^2| - |Fc^2|)^2/\Sigma w|Fo^2|^2]^{1/2}$

2. DFT Computations

2.1 DFT optimized structure of [L_2P^+][$B(C_6F_5)_4$]

DFT calculation of [$\mathbf{L_2P^+}$][$B(C_6F_5)_4$] was performed at B3LYP-D3(BJ)/6-311++G(d,p) level of theory in gas phase. The optimized geometry was in good agreement with the obtained SC-XRD molecular structure (Table T1). Frontier molecular orbital analysis reveals that the HOMO is primarily localized on the phosphorus center, whereas the LUMO is distributed over the π*-orbitals of the pyridine rings (Figure S72). Further LUMO+4 and LUMO+5, which are located over the σ* of the P–C bond.

(a)

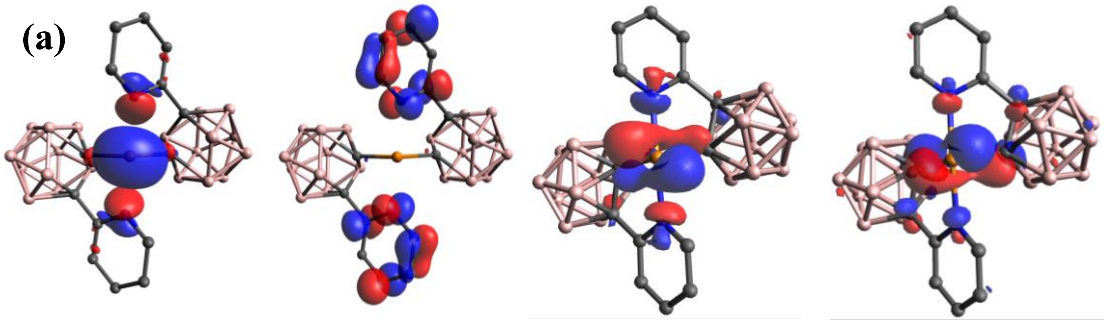

HOMO LUMO LUMO+4 LUMO+5

Figure S72. At B3LYP-D3(BJ)/6-311++G(d,p) level of theory in gas phase: **a**) HOMO, LUMO, LUMO+4 and LUMO+5 orbitals (isovalue 0.05) of [$\mathbf{L_2P^+}$][$B(C_6F_5)_4$].

Bond length (Å) & Bond angles (°)	**Experimental X-ray structure**	**DFT optimized structure**
P1−N1	2.054(2)	2.1097
P1−N2	2.031(2)	2.1090
P1−C1	1.911(3)	1.9119
P1−C2	1.913(3)	1.9119
∠C1−P1−C2	108.81°	109.27
∠N1−P1−N2	173.84°	172.02

Table T1. Comparison of bond length and bond angle for experimental vs. DFT optimized structure of [$\mathbf{L_2P^+}$][$B(C_6F_5)_4$].

2.2 DFT optimized structure of ($L(L^{nido})P^+$)

DFT calculations of (**$L(L^{nido})P^+$**) were carried out at the B3LYP-D3(BJ)/6-311++G(d,p) level of theory in the gas phase. The optimized geometry showed good agreement with the experimentally determined SC-XRD structure (Table T2), and the molecular orbitals were visualized using Avogadro. Frontier molecular orbital analysis reveals that the lone pair at phosphorus corresponds to the HOMO−1, while the HOMO is primarily localized on the nido-carborane fragment. The LUMO is mainly distributed over the π* system of the pyridine ring attached to the nido-carborane moiety (Figure S73).

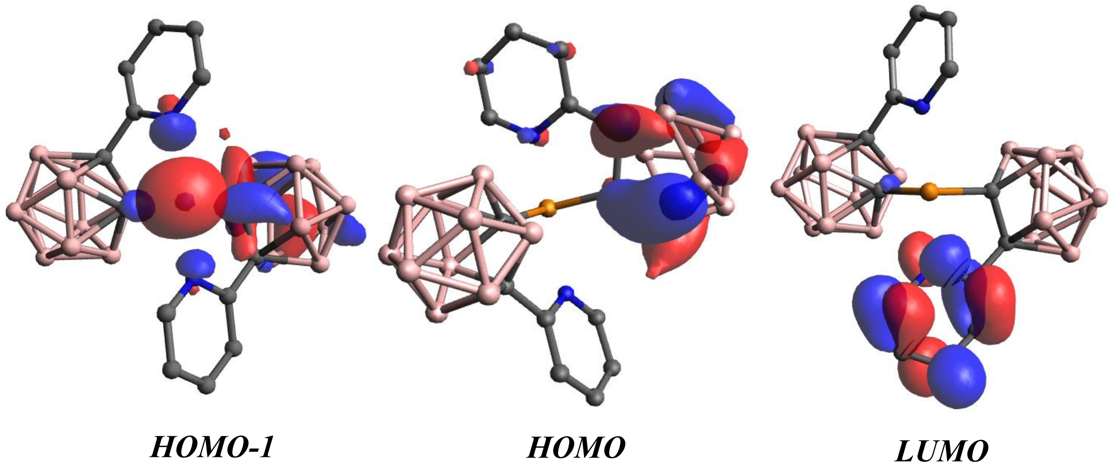

Figure S73. Optimized structure and calculated MO profiles of compound **$L(L^{nido})P^+$** at B3LYP-D3(BJ)/6-311++ G(d,p) level of theory (iso value = 0.05).

Bond length (Å) & Bond angles (°)	**Experimental X-ray structure**	**DFT optimized structure**
P1−N1	2.180(7)	2.2923
P1−N2	1.946(7)	1.9495
P1−C1	1.921(9)	1.9301
P1−C3	1.867(9)	1.8637
∠C1−P1−C3	105.03°	104.89
∠N1−P1−N2	168.93°	168.62

Table 2. Comparison of bond length and bond angle for experimental vs. DFT optimized structure of **L(L^{nido})P$^+$**.

2.3 DFT optimized structure of L$_2$PCl.

The formation of **L'LP** and the observed selectivity of third **LLi** at ortho position of pyridine ring prompted us to investigate the underlying reaction mechanism. Since **L'LP** is likely formed via the slow in situ generation of **L$_2$PCl**, we carried out DFT calculations on **L$_2$PCl** to analyze its localized molecular orbitals.

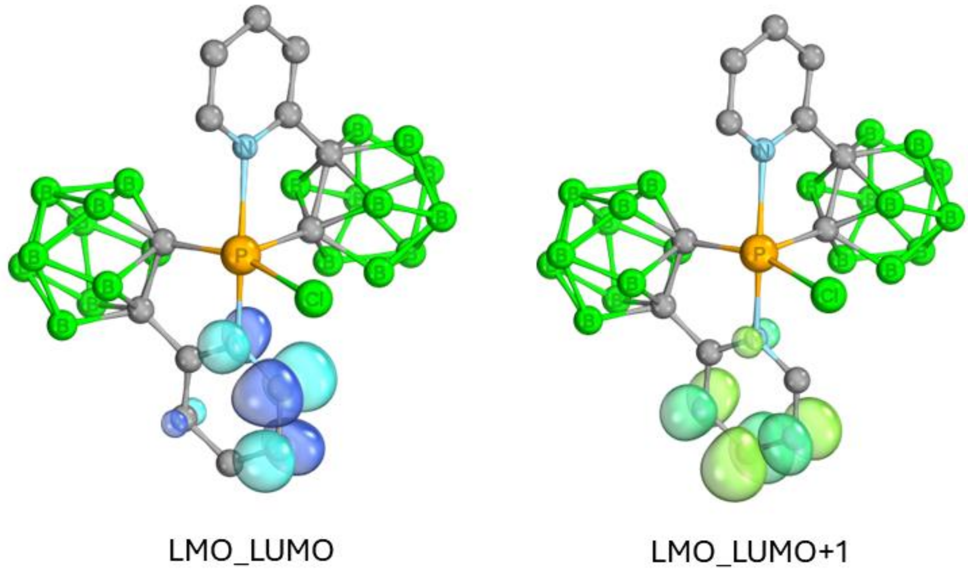

Figure S74. Optimized structure and calculated localized MO profiles of compound **L$_2$PCl** at B3LYP-D3(BJ)/6-311++G(d,p).

The nucleophilic attack at the ortho-position of the pyridine ring in **L$_2$PCl** is favored because the LUMO (E = −1.09 eV), is predominantly localized at the ortho-position. The LUMO+1 (E = −1.06 eV) is mainly distributed at the para-position.

2.4 Determination of hydride and fluoride ion affinities of [L$_2$P$^+$][B(C$_6$F$_5$)$_4$] and L(L^{nido})P$^+$ via isodesmic reactions

The assessment of the relative Lewis acidity of **L$_2$P$^+$** vs. **L(L^{nido})P$^+$**, their hydride and fluoride ion affinities were theoretically evaluated using isodesmic reactions.[10] The calculations were performed using DFT at the BP86-D3(BJ)/def2-TZVP level of theory, with the Conductor-like Polarizable Continuum Model (CPCM) employed to account for the solvent effect of $CHCl_3$. Thus, hydride abstraction from **L(L^{nido})P$^+$-H** by **L$_2$P$^+$**, giving **L(L^{nido})P$^+$** and **L$_2$P$^+$-H** is strongly exothermic with $\Delta H = -27.62$ kcal·mol^{-1}.

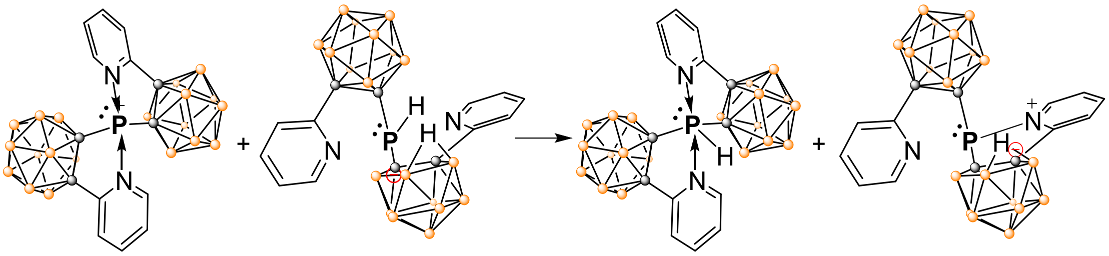

(L_2P^+) ($L(L^{nido})P^+$-H) (L_2P^+-H) ($L(L^{nido})P^+$)

Sum of electronic and thermal Enthalpies	L_2P^+	$L(L^{nido})P^+$-H	L_2P^+-H	$L(L^{nido})P^+$
	-1498.322596	-1474.284039	-1499.032380	-1473.618270
**Δ H = - 27.62 kcal/mol**				

Similarly, the fluoride abstraction from **$L(L^{nido})P^+$-F** by **L_2P^+**, giving **$L(L^{nido})P^+$** and **L_2P^+-F**, is also strongly exothermic with $\Delta H = -22.58$ kcal mol^{-1}.

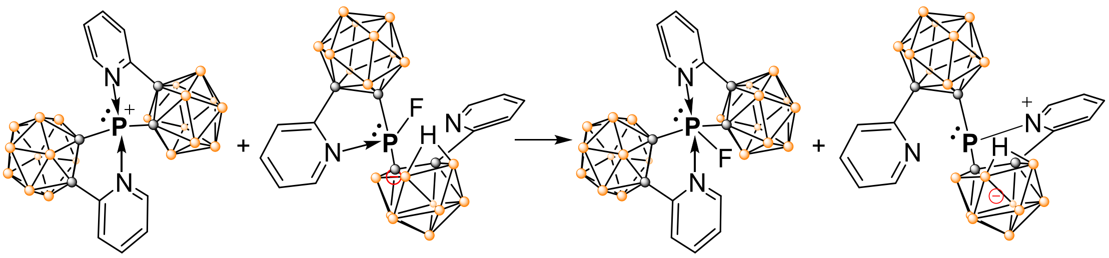

(L_2P^+) ($L(L^{nido})P^+$-F) (L_2P^+-F) ($L(L^{nido})P^+$)

Sum of electronic and thermal Enthalpies	L_2P^+	$L(L^{nido})P^+$-F	L_2P^+-F	$L(L^{nido})P^+$
	-1498.322596	-1573.625396	-1598.365708	-1473.618270
**Δ H = - 22.58kcal/mol**				

These values indicate that **L_2P^+** is a stronger Lewis acid compared to **$L(L^{nido})P^+$**.

2.5 Determination of fluoride ion affinities of $[L_2P^+][B(C_6F_5)_4]$ and similar phospheniums.

The assessment of the relative fluoride ion affinities (FIA) of **L_2P^+** with other systems were calculated at wb97xd/def2tzvp in $CHCl_3$. Interestingly, FIA for tri-coordinate system were found to be higher than the tetra-coordinate. FIA, values of following phosphenium are given below in table in KJ/mol.

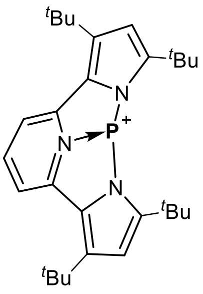

(I)

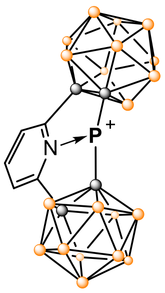

(III)

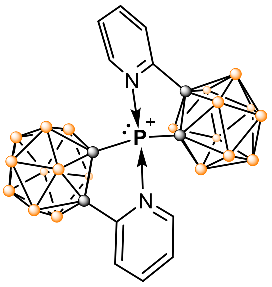

= C = BH (L_2P^+)

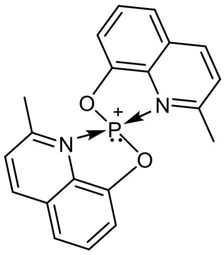

(IV)

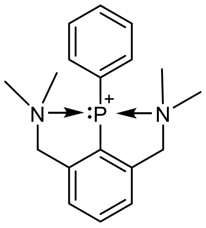

(V)

FIA values	(I)	(II)	(III)	(IV)	(V)
	**397**	**376**	**178**	**158**	**171**

Computationally calculated cartesian coordinates and energies:

At B3LYP-D3(BJ)/6-311++G(d,p)_gas phase

[L_2P^+]

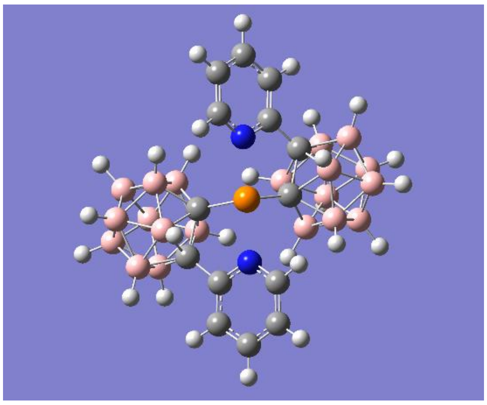

P	0.00015000	-0.00002700	-1.15938700
N	-1.30617500	1.65015000	-1.01266500
N	1.30596000	-1.64977000	-1.01283700
C	-1.31554300	-0.83674600	-0.05281700
C	1.31574700	0.83678900	-0.05282800
C	2.61023200	-0.13151600	0.25064500
C	2.42489500	-1.49889500	-0.28482400
C	3.35873200	-2.51538700	-0.15962600
H	4.24158000	-2.36393800	0.44545800
C	3.13806600	-3.70305700	-0.85070200

H	3.84779000	-4.51719500	-0.77143200
C	2.02116000	-3.81838100	-1.67526400
H	1.84441000	-4.70756700	-2.26517400
C	1.12604100	-2.76366500	-1.73931300
H	0.25552800	-2.78992900	-2.37728300
C	-2.61021400	0.13140700	0.25053800
C	-2.42517000	1.49886400	-0.28488100
C	-3.35936600	2.51504100	-0.15968900
H	-4.24227900	2.36323700	0.44521100
C	-3.13894000	3.70289400	-0.85052200
H	-3.84893000	4.51680100	-0.77125000
C	-2.02189200	3.81869600	-1.67481700
H	-1.84527500	4.70804000	-2.26453000
C	-1.12644800	2.76424200	-1.73885800
H	-0.25580100	2.79092700	-2.37662500
B	-1.15812400	-1.99371800	1.19194000
H	-0.07910000	-2.36884300	1.46435200
B	-1.63058900	-0.32659800	1.58275700
H	-0.96666600	0.50988400	2.04716800
B	-3.39882900	-0.25679500	1.70381800
H	-3.84587300	0.63901300	2.32314300
B	-2.49188100	-1.65534600	2.32685300
H	-2.36780500	-1.83786600	3.48384700
B	-2.64854500	-2.96299200	1.12694100
H	-2.62418400	-4.10072500	1.43267700
B	-1.87761500	-2.43247500	-0.36895600
H	-1.29394200	-3.10183300	-1.13069000
B	-3.64336100	-2.37007700	-0.23459500
H	-4.32333300	-3.06413100	-0.90074200
B	-4.03078600	-1.88844700	1.44156100
H	-5.01340800	-2.24587000	1.98381100
B	-4.10022300	-0.69651500	0.13187600

H	-5.01290200	-0.08878900	-0.29579300
B	-2.77607700	-1.05521500	-0.99755600
H	-2.73761600	-0.66394800	-2.10558400
B	1.63073900	0.32680400	1.58285100
H	0.96670300	-0.50952100	2.04737300
B	1.15847800	1.99391500	1.19177600
H	0.07950400	2.36918700	1.46418400
B	1.87800300	2.43240300	-0.36919000
H	1.29441200	3.10169200	-1.13104500
B	2.64904400	2.96296700	1.12663500
H	2.62487400	4.10072800	1.43227400
B	2.49223800	1.65550000	2.32670800
H	2.36821900	1.83817800	3.48368300
B	3.39897700	0.25672600	1.70382600
H	3.84591900	-0.63906100	2.32325600
B	4.10040000	0.69616700	0.13181500
H	5.01296300	0.08823200	-0.29580700
B	4.03115500	1.88826900	1.44135400
H	5.01384000	2.24561300	1.98354100
B	3.64376500	2.36977000	-0.23484300
H	4.32382700	3.06363600	-0.90109400
B	2.77628300	1.05495900	-0.99762400
H	2.73774600	0.66356000	-2.10560200

Zero-point correction= 0.473676 (Hartree/Particle)

Thermal correction to Energy= 0.500045

Thermal correction to Enthalpy= 0.500989

Thermal correction to Gibbs Free Energy= 0.422712

Sum of electronic and zero-point Energies= -1498.298836

Sum of electronic and thermal Energies= -1498.272467

Sum of electronic and thermal Enthalpies= -1498.271523

Sum of electronic and thermal Free Energies= -1498.349800

At B3LYP-D3(BJ)/6-311++G(d,p)_gas phase

L(L^{nido})P^{+}

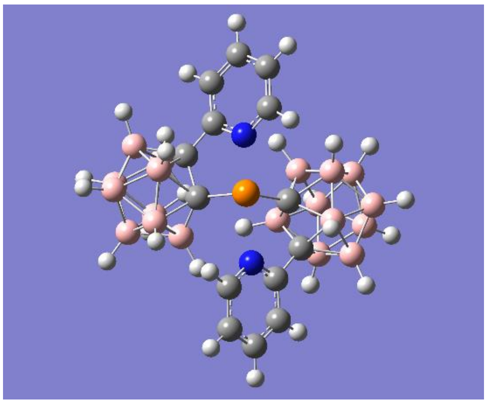

P	-0.16648800	-0.04189700	-1.31835300
N	1.45880000	1.56696500	-1.16065300
N	-1.45346400	-1.48768800	-1.08597600
C	1.09577800	-0.94149100	-0.16818200
C	2.42997600	-0.07592400	0.24165500
C	2.43943400	1.31240300	-0.29347600
C	3.40253400	2.25646400	0.03365700
H	4.17837300	2.02016600	0.74800700
C	3.32969700	3.50157100	-0.58400100
H	4.05750400	4.26756400	-0.34547100
C	2.32260100	3.75095800	-1.51186100
H	2.24304000	4.70570000	-2.01440300
C	1.39795200	2.74887700	-1.77398800
H	0.57731400	2.88897300	-2.46457000
C	-2.41774000	0.07243200	0.37815300
C	-1.24312600	0.95022900	-0.16500500
C	-2.42049400	-1.27056000	-0.16381800
C	-3.34482100	-2.28007500	0.12094900
H	-4.09365800	-2.10227900	0.87948000
C	-3.29081600	-3.46016900	-0.59644000
H	-4.00348700	-4.25017200	-0.39193900
C	-2.33722000	-3.62308900	-1.61210000

H	-2.29938500	-4.52023700	-2.21418600
C	-1.43244200	-2.60958400	-1.83248400
H	-0.66960600	-2.65996800	-2.59586400
B	1.54624400	-2.57656000	-0.43993900
H	0.97486800	-3.19742300	-1.25487800
B	2.14930600	-3.16842800	1.11330700
H	2.01571600	-4.30194500	1.41463900
B	3.57947700	-2.20223700	1.54493400
H	4.48567500	-2.63396600	2.16576600
B	3.84750900	-1.02517900	0.25178200
H	4.84563800	-0.49929700	-0.08839000
B	2.59725700	-1.27701000	-0.98362100
H	2.69200500	-0.89874700	-2.09176500
B	3.29386700	-2.65782000	-0.15650400
H	3.97608100	-3.40583700	-0.76315200
B	3.05073100	-0.52398800	1.75804600
H	3.52523100	0.32347900	2.42422300
B	2.00022800	-1.84782200	2.29758200
H	1.77030600	-2.01939400	3.44169200
B	0.74521700	-2.07971700	1.06110000
H	-0.37746400	-2.35975700	1.26413300
B	1.29621700	-0.45975900	1.48831100
H	0.62648700	0.39923700	1.90196500
B	-3.89442200	0.93860600	0.50129200
H	-4.89780400	0.33456100	0.34168200
B	-2.83121500	1.18715200	-0.89402800
H	-3.05950500	0.74115500	-1.96312000
B	-1.79461000	2.56053500	-0.56365900
H	-1.38308100	3.14672600	-1.50350900
B	-2.19026200	3.15865400	1.03221600
H	-2.08598700	4.29903900	1.33261600
B	-3.51897500	2.13523800	1.72920600

H	-4.37165700	2.57368600	2.42183500
B	-3.49299300	2.55148200	-0.03040500
H	-4.30736700	3.24052100	-0.53975000
B	-2.84445000	0.48990300	1.90546900
H	-3.26771800	-0.32109700	2.65881800
H	-1.53464500	0.75743600	2.24195700
B	-1.84486000	1.98359200	2.30733400
H	-1.58794700	2.34713200	3.40521600
B	-0.78882000	2.14349400	0.83152600
H	0.31592500	2.55533400	0.88259000

Zero-point correction= 0.466192 (Hartree/Particle)

Thermal correction to Energy= 0.492203

Thermal correction to Enthalpy= 0.493147

Thermal correction to Gibbs Free Energy= 0.415366

Sum of electronic and zero-point Energies= -1473.629910

Sum of electronic and thermal Energies= -1473.603900

Sum of electronic and thermal Enthalpies= -1473.602956

Sum of electronic and thermal Free Energies= -1473.680737

At **BP86-D3(BJ)/def2-TZVP** level of theory with CPCM in $CHCl_3$

[L_2P^+]

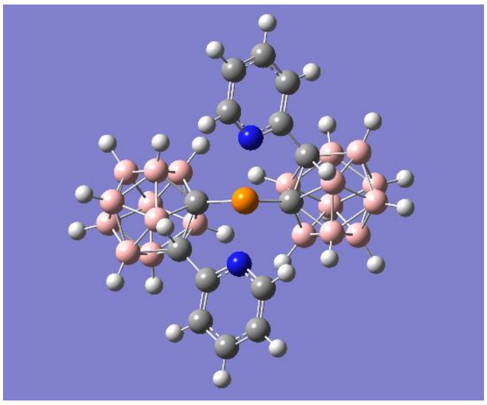

P	0.00005200	-0.00016400	-1.16319200
N	-1.29245400	1.64229400	-1.01553000
N	1.29255700	-1.64251700	-1.01524200

C	-1.31689200	-0.83471300	-0.04672000
C	1.31683100	0.83459200	-0.04667900
C	2.60932900	-0.13955000	0.25093600
C	2.41986100	-1.50293000	-0.29738500
C	3.35325800	-2.51963100	-0.19551600
H	4.24471200	-2.38027100	0.39897700
C	3.12148900	-3.69903500	-0.89845300
H	3.83110500	-4.51404700	-0.83677500
C	1.99607000	-3.80287500	-1.71121400
H	1.81037600	-4.68397300	-2.30950900
C	1.10196600	-2.74598400	-1.75458600
H	0.22698000	-2.76193000	-2.38648400
C	-2.60931000	0.13962300	0.25093000
C	-2.41966300	1.50294900	-0.29746100
C	-3.35286400	2.51981900	-0.19551000
H	-4.24424600	2.38065800	0.39913600
C	-3.12097600	3.69916400	-0.89850300
H	-3.83044000	4.51430300	-0.83675300
C	-1.99565900	3.80278100	-1.71142600
H	-1.80991700	4.68382000	-2.30979200
C	-1.10177300	2.74571100	-1.75493300
H	-0.22688900	2.76147300	-2.38697200
B	-1.15683400	-1.98000200	1.20953900
H	-0.08045900	-2.35740100	1.48740000
B	-1.62761500	-0.30887800	1.58348000
H	-0.96280200	0.52786600	2.04397500
B	-3.39441000	-0.23315500	1.70912400
H	-3.84209200	0.66483700	2.32383500
B	-2.48841100	-1.62746800	2.34265900
H	-2.36456000	-1.80070200	3.50247200
B	-2.64832000	-2.94599700	1.15830900

H	-2.62856500	-4.08166100	1.47802700
B	-1.87922000	-2.43497100	-0.34496300
H	-1.29951500	-3.11781700	-1.09636500
B	-3.64381500	-2.36533600	-0.20495000
H	-4.32912800	-3.06751100	-0.85931200
B	-4.02807000	-1.86585600	1.46420200
H	-5.01105300	-2.21866900	2.01293200
B	-4.09821700	-0.68781900	0.14257100
H	-5.01562300	-0.08875900	-0.28606100
B	-2.77660300	-1.06049800	-0.98527900
H	-2.74357100	-0.68389100	-2.09730500
B	1.62758300	0.30876300	1.58352300
H	0.96289300	-0.52811500	2.04395400
B	1.15656800	1.97982100	1.20963000
H	0.08013500	2.35706300	1.48747500
B	1.87887600	2.43496600	-0.34484800
H	1.29900900	3.11778400	-1.09614300
B	2.64790900	2.94603200	1.15843700
H	2.62798400	4.08168300	1.47819600
B	2.48819600	1.62744200	2.34274400
H	2.36432000	1.80061700	3.50256300
B	3.39438700	0.23329000	1.70914700
H	3.84221000	-0.66466300	2.32381300
B	4.09811600	0.68811400	0.14259900
H	5.01561300	0.08920100	-0.28604500
B	4.02780800	1.86608500	1.46428500
H	5.01074200	2.21902500	2.01302200
B	3.64346700	2.36557300	-0.20485700
H	4.32868800	3.06786300	-0.85919400
B	2.77643400	1.06063300	-0.98523400
H	2.74341800	0.68403100	-2.09726100

Zero-point correction=	0.473702 (Hartree/Particle)
Thermal correction to Energy=	0.500000
Thermal correction to Enthalpy=	0.500944
Thermal correction to Gibbs Free Energy=	0.423103
Sum of electronic and zero-point Energies=	-1498.349839
Sum of electronic and thermal Energies=	-1498.323541
Sum of electronic and thermal Enthalpies=	-1498.322596
Sum of electronic and thermal Free Energies=	-1498.400438

At **BP86-D3(BJ)/def2-TZVP** level of theory with CPCM in $CHCl_3$

$L(L^{nido})P^+$

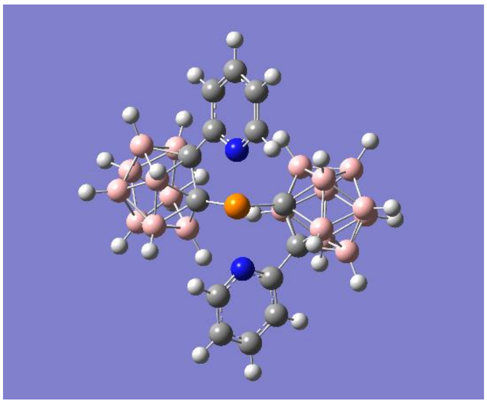

P	-0.14815700	-0.01157600	-1.30506000
N	1.44463800	1.56719800	-1.12598500
N	-1.44633100	-1.46801700	-1.10415100
C	1.09304600	-0.93209700	-0.14821500
C	2.43862000	-0.07648800	0.25606200
C	2.45369400	1.30659500	-0.29155000
C	3.44899900	2.23331800	-0.02058800
H	4.24902400	1.99519100	0.66572600
C	3.38444600	3.46597300	-0.66520500
H	4.14064900	4.21686200	-0.47405100
C	2.35264500	3.71719500	-1.56526000
H	2.28394400	4.65754600	-2.09507900

C	1.39413300	2.73425300	-1.77125700
H	0.56191900	2.87631300	-2.44695700
C	-2.42579300	0.06919500	0.37682300
C	-1.24808300	0.95895100	-0.14462200
C	-2.42144700	-1.27433600	-0.18905100
C	-3.34813100	-2.28395600	0.06595900
H	-4.10921200	-2.12705000	0.81675800
C	-3.28585100	-3.45244200	-0.67583500
H	-4.00067400	-4.24564200	-0.49530600
C	-2.32284600	-3.59294100	-1.68202200
H	-2.27814100	-4.47678300	-2.30254800
C	-1.41491500	-2.57376700	-1.87314400
H	-0.64641900	-2.61033400	-2.63167400
B	1.52377500	-2.57493500	-0.40912000
H	0.94512100	-3.19694100	-1.21638900
B	2.12192900	-3.15970900	1.14810200
H	1.97747100	-4.29014600	1.45937000
B	3.56348300	-2.20654700	1.57126100
H	4.46513700	-2.64595200	2.19520800
B	3.84447000	-1.04230000	0.26970000
H	4.84944300	-0.53414300	-0.07440500
B	2.58890800	-1.28860000	-0.96245200
H	2.68795400	-0.92127600	-2.07328800
B	3.27014300	-2.67111500	-0.12537400
H	3.94379300	-3.43303200	-0.72529200
B	3.05623600	-0.52164200	1.77424000
H	3.54104300	0.32391500	2.43492100
B	1.98958700	-1.83012700	2.32277900
H	1.75918700	-1.99210700	3.46917100
B	0.72966400	-2.05729100	1.08906700
H	-0.39596800	-2.32228000	1.29546500

B	1.30306500	-0.44198300	1.50363900
H	0.64477500	0.42566700	1.91706200
B	-3.90230200	0.92876700	0.49725000
H	-4.90862900	0.33218400	0.32897300
B	-2.82818000	1.19631800	-0.88060600
H	-3.05159300	0.77065100	-1.95803900
B	-1.80322000	2.57379400	-0.52760800
H	-1.38817200	3.18022200	-1.45190200
B	-2.21167800	3.14836300	1.07146200
H	-2.11895300	4.28753200	1.38983400
B	-3.53777200	2.10809300	1.74578400
H	-4.39678600	2.53652100	2.44182700
B	-3.50346300	2.55119600	-0.00539900
H	-4.31904100	3.24858300	-0.50583500
B	-2.85883900	0.46329200	1.90250200
H	-3.28643500	-0.35191900	2.64854100
H	-1.54880400	0.73539200	2.25072800
B	-1.86719900	1.95637500	2.33221200
H	-1.62580700	2.31116200	3.43902600
B	-0.80439900	2.14205800	0.86785200
H	0.29723200	2.56248700	0.92598700

Zero-point correction= 0.466467 (Hartree/Particle)

Thermal correction to Energy= 0.492366

Thermal correction to Enthalpy= 0.493310

Thermal correction to Gibbs Free Energy= 0.416012

Sum of electronic and zero-point Energies= -1473.645114

Sum of electronic and thermal Energies= -1473.619214

Sum of electronic and thermal Enthalpies= -1473.618270

Sum of electronic and thermal Free Energies= -1473.695568

At **BP86-D3(BJ)/def2-TZVP** level of theory with CPCM in $CHCl_3$

L_2P^+-H

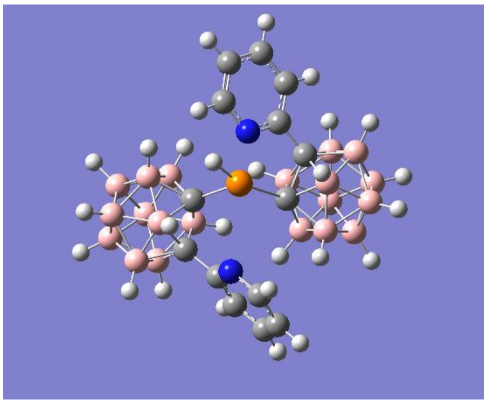

P	-0.11023600	-0.56582400	-1.41220500
N	-2.05194200	1.77419200	-1.70790300
N	1.19849800	-1.88662600	-0.91004700
C	-1.51842600	-0.94075900	-0.19420400
C	1.30310000	0.72166900	0.12394200
C	2.63213300	-0.19286000	0.13072000
C	2.38627800	-1.58842100	-0.31680400
C	3.34916100	-2.57111900	-0.13602100
H	4.28559900	-2.29604700	0.32505900
C	3.10121700	-3.87738100	-0.53214300
H	3.84912900	-4.64520900	-0.38157500
C	1.87563800	-4.17793400	-1.11533400
H	1.62336600	-5.17942300	-1.43471500
C	0.95574700	-3.16609000	-1.29092500
H	-0.00517600	-3.35099400	-1.74156900
C	-2.57287200	0.32967100	0.15526100
C	-2.22314200	1.69065800	-0.38627300
C	-2.09547100	2.78688300	0.46449300
H	-2.21467400	2.67648500	1.53181700
C	-1.80810600	4.02690300	-0.09488900
H	-1.70384000	4.89817900	0.53970600
C	-1.64395300	4.12478400	-1.47210800

H	-1.41534900	5.07025700	-1.94699400
C	-1.76319200	2.96707300	-2.23452800
H	-1.62010700	2.99297200	-3.30968800
B	-1.47342700	-2.03556100	1.11004800
H	-0.45565900	-2.58201900	1.33748400
B	-1.57620500	-0.29002100	1.40300400
H	-0.70734000	0.37738800	1.78764900
B	-3.27495100	0.12466200	1.68902100
H	-3.51416800	1.08561300	2.32347100
B	-2.60258600	-1.38727200	2.31761300
H	-2.41158600	-1.53474200	3.47387000
B	-3.12357700	-2.69696300	1.23308200
H	-3.29949600	-3.80218000	1.61382400
B	-2.40255500	-2.40030300	-0.34907800
H	-2.02471600	-3.20061400	-1.12385900
B	-4.09446500	-1.97649100	-0.07829600
H	-4.96012700	-2.55008200	-0.64146900
B	-4.23098600	-1.35653300	1.58237900
H	-5.21285400	-1.47997600	2.22870000
B	-4.18313400	-0.22832200	0.20791200
H	-5.01932800	0.50883200	-0.17245100
B	-3.04802400	-0.88386400	-0.98555200
H	-3.06698200	-0.61685400	-2.12526400
B	1.94606100	0.21199900	1.63159800
H	1.39226900	-0.63388000	2.23449200
B	1.40698300	1.86176800	1.39643400
H	0.42044900	2.21845100	1.93299500
B	1.73788100	2.32366400	-0.26493000
H	0.97726500	2.98809100	-0.87279500
B	2.81777500	2.87275500	1.04282900
H	2.85922800	4.00915700	1.37372500
B	2.94904400	1.54134800	2.22380300

H	3.07914500	1.70703400	3.38807700
B	3.71900200	0.17704200	1.40811600
H	4.32819000	-0.69757800	1.91274700
B	4.07285200	0.65259000	-0.26906800
H	4.90343500	0.07796800	-0.87909800
B	4.25577800	1.82063700	1.04326800
H	5.33111800	2.19139700	1.37077600
B	3.51246300	2.31888700	-0.50194000
H	4.04371200	3.03830400	-1.27678000
B	2.51834600	0.99170800	-1.07299000
H	2.30491600	0.63220400	-2.17547500
H	-0.63881900	-1.63545100	-2.22731100

Zero-point correction= 0.480143 (Hartree/Particle)

Thermal correction to Energy= 0.507208

Thermal correction to Enthalpy= 0.508153

Thermal correction to Gibbs Free Energy= 0.427151

Sum of electronic and zero-point Energies= -1499.060389

Sum of electronic and thermal Energies= -1499.033324

Sum of electronic and thermal Enthalpies= -1499.032380

Sum of electronic and thermal Free Energies= -1499.113381

At **BP86-D3(BJ)/def2-TZVP** level of theory with CPCM in $CHCl_3$

L_2P^+-F

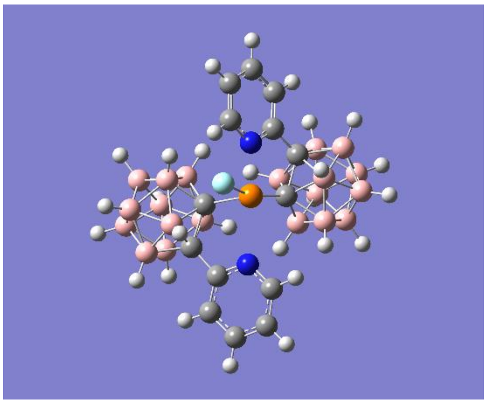

P	0.08426000	-0.14715200	-1.31947000
N	-1.65142900	1.72277100	-1.03228400
N	1.46898600	-1.57014500	-0.94129900
C	-1.22949300	-0.89935200	-0.06038700
C	1.30684600	0.94482900	0.02653700
C	2.68307200	0.12247200	0.26448100
C	2.56118400	-1.28081400	-0.19235700
C	3.53421500	-2.23067500	0.06614400
H	4.38823300	-1.95879200	0.66915600
C	3.39425700	-3.50846400	-0.46303800
H	4.14118900	-4.26613600	-0.26355700
C	2.29650900	-3.78457600	-1.26841100
H	2.15813500	-4.75396000	-1.72680000
C	1.35908300	-2.79294800	-1.49838900
H	0.50293400	-2.93097100	-2.13665100
C	-2.59522200	0.00799800	0.33443200
C	-2.66382900	1.38666100	-0.23591900
C	-3.73304000	2.24008600	0.02261900
H	-4.53452500	1.93825600	0.68131000
C	-3.73885800	3.48512900	-0.59820100
H	-4.55367500	4.17582400	-0.41984800
C	-2.69646500	3.82529700	-1.45622800
H	-2.67583400	4.77823700	-1.96829400
C	-1.66905500	2.90821200	-1.64528000
H	-0.83481700	3.12144100	-2.30175000
B	-0.86134300	-1.93294200	1.24890600
H	0.26553100	-2.20230800	1.44232500
B	-1.42506900	-0.28249100	1.54157300
H	-0.79542400	0.62717700	1.89304700
B	-3.16036100	-0.34506900	1.90003900
H	-3.60619300	0.54909800	2.52333200

B	-2.07497700	-1.60641000	2.50155800
H	-1.80451800	-1.68160300	3.64889900
B	-2.25421600	-3.01518100	1.43040700
H	-2.10090900	-4.12339800	1.81223600
B	-1.70049700	-2.54685500	-0.18182100
H	-1.16243900	-3.24949100	-0.94606800
B	-3.43377000	-2.60459100	0.15853300
H	-4.12910400	-3.40228100	-0.36627600
B	-3.67425500	-2.03292900	1.83224500
H	-4.55908300	-2.42229100	2.51216700
B	-3.98959300	-0.95766800	0.46213200
H	-5.00573200	-0.48016100	0.10659500
B	-2.76877100	-1.27597400	-0.79204500
H	-2.94433800	-0.98874600	-1.91207400
B	1.78446400	0.56426500	1.64468000
H	1.23070700	-0.28645400	2.22396100
B	1.15983500	2.15951800	1.22260400
H	0.08913600	2.48454800	1.58321800
B	1.70024400	2.56057800	-0.40601700
H	0.99844300	3.13641400	-1.15139800
B	2.54854500	3.24081600	0.99445700
H	2.46705100	4.39136300	1.25577400
B	2.60054400	1.98997600	2.26161400
H	2.56111300	2.22622300	3.41876800
B	3.56090900	0.63540700	1.63605000
H	4.14269300	-0.17324700	2.26565900
B	4.10317000	1.04842200	-0.00487700
H	5.03590400	0.49788300	-0.46933800
B	4.03722000	2.29409200	1.24796400
H	5.03027700	2.75664800	1.69256600
B	3.48091600	2.65932100	-0.40648300
H	4.05859700	3.37147500	-1.15191200

B	2.66414300	1.24392600	-1.02498600
H	2.59709000	0.80583100	-2.11493700
F	-0.58757200	-1.36954100	-2.58907300

Zero-point correction= 0.474119 (Hartree/Particle)

Thermal correction to Energy= 0.502155

Thermal correction to Enthalpy= 0.503099

Thermal correction to Gibbs Free Energy= 0.421382

Sum of electronic and zero-point Energies= -1598.394688

Sum of electronic and thermal Energies= -1598.366652

Sum of electronic and thermal Enthalpies= -1598.365708

Sum of electronic and thermal Free Energies= -1598.447425

At **BP86-D3(BJ)/def2-TZVP** level of theory with CPCM in $CHCl_3$

L(L^{nido})P$^+$-H

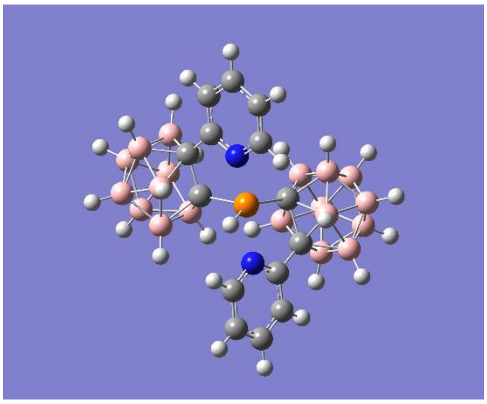

P	-0.56288600	-0.07281400	-1.28908200
N	1.17086400	1.42577700	-1.25169600
N	-2.17481700	-1.24940800	-1.41133300
C	1.43857700	-0.95920900	0.55208900
C	2.71542400	-0.05965700	-0.00014000
C	2.40553500	1.20940300	-0.75359600
C	3.42527200	2.14059200	-0.96178900
H	4.40868500	1.94616400	-0.56284500
C	3.17190300	3.30406800	-1.67489100

H	3.96118400	4.02944900	-1.83066700
C	1.89666500	3.51522100	-2.18482200
H	1.64483500	4.40295600	-2.74966700
C	0.93463400	2.54591500	-1.94989800
H	-0.07213000	2.66474900	-2.32568700
C	-2.74721600	-0.01572900	0.50898100
C	-1.50470600	0.83383300	0.05948700
C	-2.98518100	-1.18598500	-0.33643000
C	-3.94734700	-2.17649300	-0.12416000
H	-4.58387200	-2.11873400	0.74749400
C	-4.06741200	-3.20391300	-1.04553500
H	-4.81071600	-3.97819400	-0.90012600
C	-3.22190700	-3.24229200	-2.16167700
H	-3.29074400	-4.03364600	-2.89541900
C	-2.27146200	-2.25180000	-2.30217100
H	-1.55677000	-2.23841400	-3.11599700
B	1.89077900	-2.60466000	0.46817700
H	1.05299700	-3.41172400	0.23557100
B	3.27620000	-2.83361500	1.56487400
H	3.46078000	-3.85599400	2.14016900
B	4.61141200	-1.82841500	0.96148500
H	5.75575500	-2.11361300	1.09593800
B	4.05807800	-1.01334700	-0.50515700
H	4.70155900	-0.62728600	-1.41908200
B	2.36175200	-1.50111400	-0.79317000
H	1.90843400	-1.45569600	-1.88178300
B	3.54563400	-2.67402300	-0.18987900
H	3.91190300	-3.56742900	-0.87992200
B	4.09081800	-0.14304300	1.03753700
H	4.77537300	0.80875800	1.17740100
B	3.60665700	-1.25174000	2.31547300
H	4.02187800	-1.12439700	3.41945900

B	1.93156100	-1.74510400	1.98694700
H	1.11964900	-1.94284600	2.82753500
B	2.41515100	-0.09829100	1.66542200
H	2.01180300	0.86339700	2.21008000
B	-4.05121400	0.94553200	1.04454200
H	-5.15207300	0.53856300	0.88788100
B	-3.09993800	1.43115200	-0.36536000
H	-3.51308500	1.33730000	-1.46775800
B	-1.85974500	2.54147700	0.15817700
H	-1.45892600	3.32946600	-0.62784500
B	-1.98764900	2.72177700	1.89102100
H	-1.69765100	3.72361900	2.46164100
B	-3.37511400	1.70390200	2.48019900
H	-4.07549500	2.03237100	3.38184000
B	-3.47225500	2.58179400	0.90633800
H	-4.22675400	3.47846000	0.72146700
B	-2.93860700	0.01277300	2.13067100
H	-3.39978900	-0.91091000	2.71597600
H	-1.55540500	0.03059600	2.32766700
B	-1.68981500	1.20826600	2.76213000
H	-1.28575800	1.23433100	3.87990800
B	-0.78203100	1.63889200	1.24300400
H	0.36271700	1.91440300	1.21732700
H	-1.06246800	0.71713800	-2.37795400

Zero-point correction= 0.480143 (Hartree/Particle)

Thermal correction to Energy= 0.507208

Thermal correction to Enthalpy= 0.508153

Thermal correction to Gibbs Free Energy= 0.427151

Sum of electronic and zero-point Energies= -1499.060389

Sum of electronic and thermal Energies= -1499.033324

Sum of electronic and thermal Enthalpies= -1499.032380

Sum of electronic and thermal Free Energies= -1499.113381

At **BP86-D3(BJ)/def2-TZVP** level of theory with CPCM in $CHCl_3$

L(L^{nido})P^{+}-F

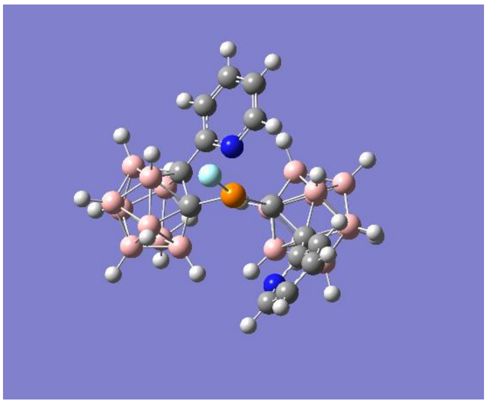

P	-0.16889400	0.60431600	-1.10331000
N	2.56407300	2.19993100	0.43212400
N	-1.11700100	-0.94660500	-1.51064900
C	0.85048400	-0.73128600	0.38699900
C	2.46750800	-0.22138700	0.48095800
C	2.86312600	1.06443300	-0.20245400
C	3.53226300	1.04376200	-1.42780600
H	3.77395800	0.10663100	-1.90722800
C	3.86696900	2.25211100	-2.02633000
H	4.37782300	2.26439000	-2.98169800
C	3.52863200	3.43914700	-1.38400000
H	3.76027400	4.40344900	-1.81845100
C	2.88467100	3.35671700	-0.15284700
H	2.61325300	4.25679600	0.39000600
C	-2.54531200	-0.10677000	0.18013900
C	-1.54336600	1.07617500	0.03803200
C	-2.21210800	-1.18938800	-0.74010800
C	-2.92633900	-2.37161600	-0.92694100
H	-3.78474400	-2.56697900	-0.30000800
C	-2.54130700	-3.24789900	-1.92806100

H	-3.09676300	-4.16379500	-2.08726300
C	-1.44679000	-2.94139500	-2.74375500
H	-1.13355500	-3.59863600	-3.54244200
C	-0.74888800	-1.77787500	-2.50596500
H	0.11385100	-1.48096800	-3.08352200
B	0.84379500	-2.44671200	0.37105300
H	0.00434700	-3.00779800	-0.23372100
B	1.53727400	-2.98209900	1.90969700
H	1.17514100	-3.98698100	2.42287700
B	3.21089000	-2.40645800	1.98420300
H	4.07165000	-2.98995600	2.55212100
B	3.55332700	-1.53659100	0.48516900
H	4.59341100	-1.38828800	-0.05156800
B	2.07170100	-1.54767900	-0.50096300
H	2.09171600	-1.41311000	-1.67091100
B	2.53374100	-2.97586200	0.43280600
H	2.89285700	-3.96120300	-0.11737000
B	3.17683900	-0.63402500	1.96693300
H	3.96589800	0.11650400	2.42029900
B	1.92547100	-1.51938800	2.84672500
H	1.85497600	-1.46685700	4.02724600
B	0.47365300	-1.56055700	1.83224500
H	-0.62706100	-1.51051400	2.24134100
B	1.47117000	-0.11418000	1.86355200
H	1.11727000	0.92715700	2.25135500
B	-4.15024300	0.41877500	0.44024500
H	-5.04116800	-0.25236800	0.04401600
B	-3.12038500	1.25500200	-0.72763800
H	-3.27672100	1.13391600	-1.88568900
B	-2.34946900	2.61293900	0.07163900
H	-2.01046500	3.52416200	-0.60274100
B	-2.87770200	2.62205900	1.74355100

H	-3.00646600	3.61608800	2.38153400
B	-4.01906400	1.22761700	1.99614300
H	-4.95257900	1.28765700	2.72764900
B	-4.03097700	2.16260900	0.45503700
H	-4.95032200	2.83514300	0.12599600
B	-3.07278700	-0.25685500	1.72391200
H	-3.35931300	-1.31333200	2.17956800
H	-1.82600700	0.11904300	2.21876800
B	-2.36063200	1.18056200	2.63398300
H	-2.21370800	1.22276300	3.81262900
B	-1.31355100	1.95897200	1.35629200
H	-0.28808300	2.51334200	1.55630400
F	-0.90312000	1.30852200	-2.63109500

Zero-point correction= 0.465643 (Hartree/Particle)

Thermal correction to Energy= 0.493695

Thermal correction to Enthalpy= 0.494639

Thermal correction to Gibbs Free Energy= 0.411643

Sum of electronic and zero-point Energies= -1573.654392

Sum of electronic and thermal Energies= -1573.626340

Sum of electronic and thermal Enthalpies= -1573.625396

Sum of electronic and thermal Free Energies= -1573.708391

At **B3LYP-D3(BJ)/6-311++G(d,p)** gas phase

Int1a (ligand assisted)

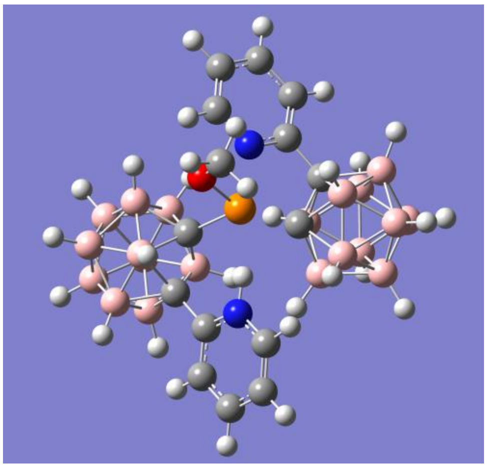

N	1.17710600	1.61521000	0.93640500
N	-1.17856000	-1.60177200	0.95860100
C	1.33697400	-0.76433800	-0.18895900
C	-1.38450600	0.80266600	-0.16670400
C	-2.63196000	-0.22104100	-0.34627200
C	-2.30807800	-1.55058900	0.21591800
C	-3.11648700	-2.66223600	0.05047500
H	-4.00993500	-2.58002700	-0.55199300
C	-2.75911600	-3.85326400	0.67316200
H	-3.37467800	-4.73620100	0.55288400
C	-1.61220800	-3.89083400	1.46018900
H	-1.30665200	-4.79272100	1.97290700
C	-0.84362300	-2.74798400	1.58541900
H	0.05667500	-2.70725000	2.17487900
C	2.49705800	0.31073900	-0.56376300
C	2.24089700	1.60811900	0.10467600
C	3.05041100	2.72357700	-0.03393600
H	3.88738300	2.68943000	-0.71711200
C	2.77059200	3.85321400	0.72821900
H	3.38941100	4.73718300	0.63570500
C	1.70478300	3.82936300	1.62529700
H	1.47392400	4.67933300	2.25276800
C	0.92547300	2.68977300	1.70412300
H	0.08402500	2.61287200	2.37668100

B	1.10373300	-1.85128000	-1.47542600
H	0.03036400	-2.30425900	-1.62601900
B	1.39951800	-0.13675500	-1.79703200
H	0.64423600	0.68175400	-2.11125100
B	3.11352300	0.10330500	-2.15111600
H	3.39405100	1.07506500	-2.75391700
B	2.23594400	-1.32666800	-2.75553400
H	1.95923500	-1.43705000	-3.89541500
B	2.65047500	-2.69286700	-1.68523800
H	2.66702800	-3.80579100	-2.07316600
B	2.05581900	-2.31968800	-0.05290100
H	1.62285300	-3.08901700	0.71876100
B	3.78358100	-2.10868200	-0.44184100
H	4.60174000	-2.78523100	0.06986500
B	3.89085200	-1.48678900	-2.11230600
H	4.81162700	-1.72169200	-2.80885500
B	4.06026600	-0.38132100	-0.73824800
H	4.97391800	0.27530600	-0.39017400
B	2.96617900	-0.92608700	0.53869300
H	3.14097700	-0.60570400	1.64659100
B	-1.78202900	0.24176500	-1.75800200
H	-1.12008300	-0.57610200	-2.25537300
B	-1.38760500	1.94044200	-1.44236500
H	-0.36273100	2.39420900	-1.79220600
B	-2.01984400	2.37530100	0.14918000
H	-1.41698300	3.10381500	0.84479400
B	-2.92118400	2.82553100	-1.30808600
H	-2.98010800	3.95265500	-1.64789900
B	-2.76805000	1.49163400	-2.48159200
H	-2.72988000	1.64098000	-3.64956200
B	-3.54633100	0.06153500	-1.76041200
H	-3.97973600	-0.87420000	-2.32847800

B	-4.17708400	0.51311400	-0.16573800
H	-5.02034600	-0.13665500	0.33746500
B	-4.25626400	1.66385300	-1.50930200
H	-5.29144000	1.94531000	-1.99658500
B	-3.79489000	2.22244900	0.12303700
H	-4.47047300	2.89510100	0.81599100
B	-2.81375200	0.98448000	0.85533300
H	-2.72554800	0.63419100	1.97304100
H	-0.72091800	0.66371300	2.04283700
O	0.89913400	-0.75095400	2.27822800
C	0.79164000	-0.32292900	3.63072800
H	0.92716600	-1.19698600	4.26793800
H	1.57277400	0.40861800	3.85662700
H	-0.18968700	0.11703100	3.84554700
P	0.02290200	-0.00239200	1.04632600

Zero-point correction= 0.526373 (Hartree/Particle)

Thermal correction to Energy= 0.556665

Thermal correction to Enthalpy= 0.557609

Thermal correction to Gibbs Free Energy= 0.469782

Sum of electronic and zero-point Energies= -1613.972425

Sum of electronic and thermal Energies= -1613.942133

Sum of electronic and thermal Enthalpies= -1613.941189

Sum of electronic and thermal Free Energies= -1614.029015

At **B3LYP-D3(BJ)/6-311++G(d,p)** gas phase

Int1b (oxidative addition)

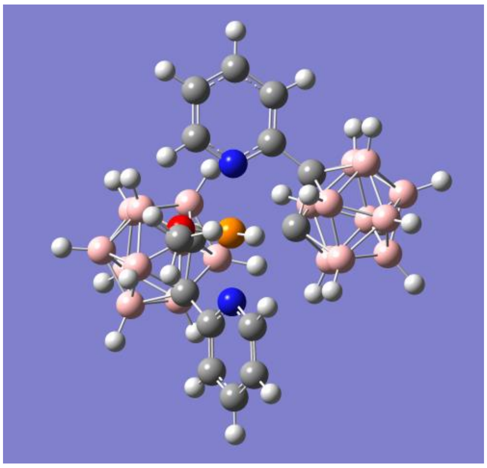

N	1.99683700	1.73318000	1.16013900
N	-1.77366300	-1.53614000	0.59719100
C	1.13640500	-1.05936300	-0.02488400
C	-1.25417900	0.96616800	-0.06584200
C	-2.71723100	0.41853600	-0.41635800
C	-2.78996000	-1.03201000	-0.13617300
C	-3.83932300	-1.82790700	-0.56773700
H	-4.63459300	-1.37795800	-1.14513500
C	-3.83886600	-3.17988200	-0.24849500
H	-4.64533600	-3.82050200	-0.58324900
C	-2.79307900	-3.69159000	0.51338600
H	-2.75317400	-4.73532600	0.79420400
C	-1.78341700	-2.84333200	0.92966300
H	-0.95707100	-3.17143800	1.53666200
C	2.55818500	-0.14473700	-0.34864600
C	2.75017700	1.25435500	0.14994300
C	3.73841900	2.08535400	-0.36803400
H	4.36531400	1.72522300	-1.16791100
C	3.90887200	3.36443000	0.14934300
H	4.67808400	4.00680400	-0.26160500
C	3.09787600	3.81901400	1.18885000
H	3.20880500	4.80965500	1.60737900
C	2.13246200	2.96730400	1.67858400
H	1.44455500	3.22525200	2.47124500

B	0.82775800	-2.08211600	-1.34871300
H	-0.29910400	-2.30273900	-1.60021600
B	1.42857200	-0.43694900	-1.61373700
H	0.82558100	0.47549400	-2.00184400
B	3.18180100	-0.54062500	-1.88788500
H	3.66026900	0.32790000	-2.51944100
B	2.11064700	-1.78161700	-2.53250000
H	1.89966200	-1.84732600	-3.68964300
B	2.21535200	-3.18944100	-1.44692800
H	2.06343000	-4.29556400	-1.82446300
B	1.58538700	-2.70337600	0.13064200
H	1.00498800	-3.37644800	0.89037400
B	3.33657000	-2.78258500	-0.11293200
H	3.98851500	-3.57827400	0.46121300
B	3.66802700	-2.23359000	-1.77985700
H	4.58097300	-2.62949000	-2.41069300
B	3.93879700	-1.14881300	-0.40286500
H	4.93497400	-0.67222100	0.00653400
B	2.63811800	-1.43353700	0.78870400
H	2.73769500	-1.13678600	1.91906600
B	-1.71348200	0.85228100	-1.72843100
H	-1.27539300	-0.02447900	-2.37058000
B	-0.84760700	2.26194300	-1.09823700
H	0.28150800	2.43020900	-1.38829900
B	-1.37780200	2.55708300	0.55640500
H	-0.60719600	2.88552600	1.38676300
B	-2.04369000	3.53074800	-0.76280800
H	-1.75749300	4.66936900	-0.88029200
B	-2.25463300	2.45968500	-2.17820000
H	-2.13208700	2.81823500	-3.29443300
B	-3.45182000	1.22152400	-1.73196800
H	-4.13674700	0.59663800	-2.45821800

B	-3.97913700	1.52644800	-0.05725100
H	-5.00162600	1.08197000	0.32201300
B	-3.65913300	2.88233800	-1.15185300
H	-4.54114400	3.55222800	-1.55503300
B	-3.11301000	2.95826300	0.54610100
H	-3.58654500	3.66382500	1.36348700
B	-2.56606400	1.36251200	1.00147000
H	-2.59136600	0.78392300	2.02904100
O	0.11851700	-1.58414400	2.31826900
C	-0.30974800	-1.31977100	3.65559900
H	0.38421200	-1.82838300	4.32441800
H	-0.30100400	-0.24494300	3.87083000
H	-1.32055500	-1.70479800	3.82360600
P	-0.18935700	-0.34699100	1.16588300
H	1.20907400	1.13286900	1.52754500

Zero-point correction= 0.528460 (Hartree/Particle)

Thermal correction to Energy= 0.557601

Thermal correction to Enthalpy= 0.558545

Thermal correction to Gibbs Free Energy= 0.474905

Sum of electronic and zero-point Energies= -1613.995171

Sum of electronic and thermal Energies= -1613.966031

Sum of electronic and thermal Enthalpies= -1613.965087

Sum of electronic and thermal Free Energies= -1614.048726

At **B3LYP-D3(BJ)/6-311++G(d,p)** gas phase

Int1b (oxidative addition)

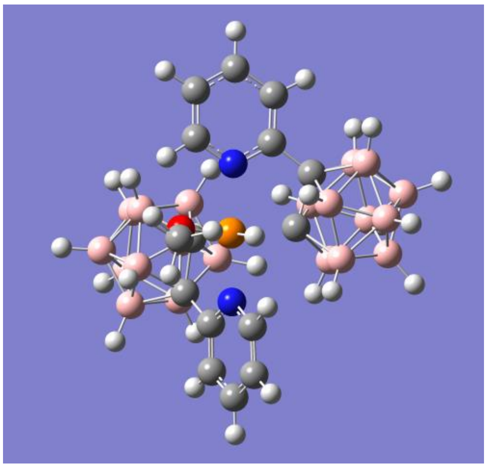

N	1.17710600	1.61521000	0.93640500
N	-1.17856000	-1.60177200	0.95860100
C	1.33697400	-0.76433800	-0.18895900
C	-1.38450600	0.80266600	-0.16670400
C	-2.63196000	-0.22104100	-0.34627200
C	-2.30807800	-1.55058900	0.21591800
C	-3.11648700	-2.66223600	0.05047500
H	-4.00993500	-2.58002700	-0.55199300
C	-2.75911600	-3.85326400	0.67316200
H	-3.37467800	-4.73620100	0.55288400
C	-1.61220800	-3.89083400	1.46018900
H	-1.30665200	-4.79272100	1.97290700
C	-0.84362300	-2.74798400	1.58541900
H	0.05667500	-2.70725000	2.17487900
C	2.49705800	0.31073900	-0.56376300
C	2.24089700	1.60811900	0.10467600
C	3.05041100	2.72357700	-0.03393600
H	3.88738300	2.68943000	-0.71711200
C	2.77059200	3.85321400	0.72821900
H	3.38941100	4.73718300	0.63570500
C	1.70478300	3.82936300	1.62529700
H	1.47392400	4.67933300	2.25276800
C	0.92547300	2.68977300	1.70412300
H	0.08402500	2.61287200	2.37668100

B	1.10373300	-1.85128000	-1.47542600
H	0.03036400	-2.30425900	-1.62601900
B	1.39951800	-0.13675500	-1.79703200
H	0.64423600	0.68175400	-2.11125100
B	3.11352300	0.10330500	-2.15111600
H	3.39405100	1.07506500	-2.75391700
B	2.23594400	-1.32666800	-2.75553400
H	1.95923500	-1.43705000	-3.89541500
B	2.65047500	-2.69286700	-1.68523800
H	2.66702800	-3.80579100	-2.07316600
B	2.05581900	-2.31968800	-0.05290100
H	1.62285300	-3.08901700	0.71876100
B	3.78358100	-2.10868200	-0.44184100
H	4.60174000	-2.78523100	0.06986500
B	3.89085200	-1.48678900	-2.11230600
H	4.81162700	-1.72169200	-2.80885500
B	4.06026600	-0.38132100	-0.73824800
H	4.97391800	0.27530600	-0.39017400
B	2.96617900	-0.92608700	0.53869300
H	3.14097700	-0.60570400	1.64659100
B	-1.78202900	0.24176500	-1.75800200
H	-1.12008300	-0.57610200	-2.25537300
B	-1.38760500	1.94044200	-1.44236500
H	-0.36273100	2.39420900	-1.79220600
B	-2.01984400	2.37530100	0.14918000
H	-1.41698300	3.10381500	0.84479400
B	-2.92118400	2.82553100	-1.30808600
H	-2.98010800	3.95265500	-1.64789900
B	-2.76805000	1.49163400	-2.48159200
H	-2.72988000	1.64098000	-3.64956200
B	-3.54633100	0.06153500	-1.76041200
H	-3.97973600	-0.87420000	-2.32847800

B	-4.17708400	0.51311400	-0.16573800
H	-5.02034600	-0.13665500	0.33746500
B	-4.25626400	1.66385300	-1.50930200
H	-5.29144000	1.94531000	-1.99658500
B	-3.79489000	2.22244900	0.12303700
H	-4.47047300	2.89510100	0.81599100
B	-2.81375200	0.98448000	0.85533300
H	-2.72554800	0.63419100	1.97304100
H	-0.72091800	0.66371300	2.04283700
O	0.89913400	-0.75095400	2.27822800
C	0.79164000	-0.32292900	3.63072800
H	0.92716600	-1.19698600	4.26793800
H	1.57277400	0.40861800	3.85662700
H	-0.18968700	0.11703100	3.84554700
P	0.02290200	-0.00239200	1.04632600

Zero-point correction= 0.528460 (Hartree/Particle)

Thermal correction to Energy= 0.557601

Thermal correction to Enthalpy= 0.558545

Thermal correction to Gibbs Free Energy= 0.474905

Sum of electronic and zero-point Energies= -1613.995171

Sum of electronic and thermal Energies= -1613.966031

Sum of electronic and thermal Enthalpies= -1613.965087

Sum of electronic and thermal Free Energies= -1614.048726

At **B3LYP-D3(BJ)/6-311++G(d,p)** gas phase

Int2

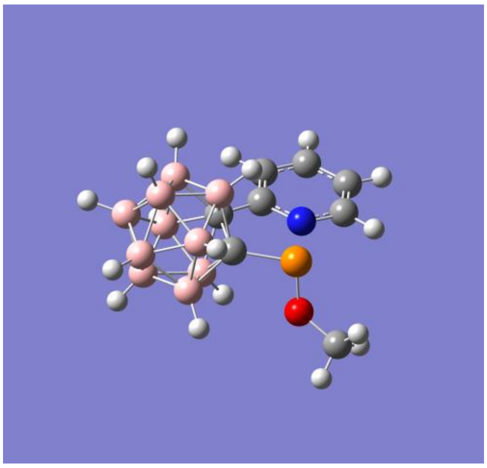

N	1.72450100	-0.20513400	-0.33297500
C	-0.69168600	0.71187500	-0.31306600
C	-0.52158200	-0.84505900	0.10002000
C	0.90948300	-1.21032300	0.07797800
C	1.43339400	-2.44577800	0.41627600
H	0.76673700	-3.23442500	0.73720400
C	2.80960200	-2.63559800	0.33116700
H	3.23935100	-3.59494400	0.59215000
C	3.63327500	-1.59257600	-0.09751200
H	4.70439900	-1.71711600	-0.17982300
C	3.05973700	-0.38219700	-0.43079700
H	3.63646000	0.46260600	-0.78511600
B	-2.12008600	0.98121000	-1.20740400
H	-2.08224600	1.81199400	-2.04078700
B	-1.15290400	-0.47669500	-1.48303600
H	-0.40588500	-0.64066600	-2.37867100
B	-1.80267200	-1.79868800	-0.48089100
H	-1.54971200	-2.90020700	-0.81058200
B	-2.88056800	-0.63054700	-1.29943700
H	-3.52777300	-0.95304400	-2.22850900
B	-3.41325600	0.55781400	-0.07089200
H	-4.46241000	1.08804700	-0.13525600
B	-1.98397900	1.44743500	0.51028500
H	-1.87599100	2.58151800	0.80165800

B	-2.67099600	0.12581400	1.49903000
H	-3.17654200	0.33813400	2.54096400
B	-3.21941400	-1.15811300	0.37588600
H	-4.13016100	-1.85849300	0.63322900
B	-1.67790800	-1.33465700	1.23956700
H	-1.34687900	-2.13515100	2.03724300
B	-0.93972800	0.28389300	1.35884700
H	-0.08502600	0.55944000	2.11264100
O	1.25374500	2.27484800	0.59153400
C	2.12327900	3.44336800	0.60320000
H	3.05380800	3.17115300	1.09920500
H	2.31795900	3.79858700	-0.41086300
H	1.60373800	4.20835200	1.17492400
P	0.93288300	1.46678400	-0.76629400

Zero-point correction= 0.279120 (Hartree/Particle)

Thermal correction to Energy= 0.295783

Thermal correction to Enthalpy= 0.296727

Thermal correction to Gibbs Free Energy= 0.236775

Sum of electronic and zero-point Energies= -1034.854283

Sum of electronic and thermal Energies= -1034.837620

Sum of electronic and thermal Enthalpies= -1034.836676

Sum of electronic and thermal Free Energies= -1034.896628

At **B3LYP-D3(BJ)/6-311++G(d,p)** gas phase

Int3

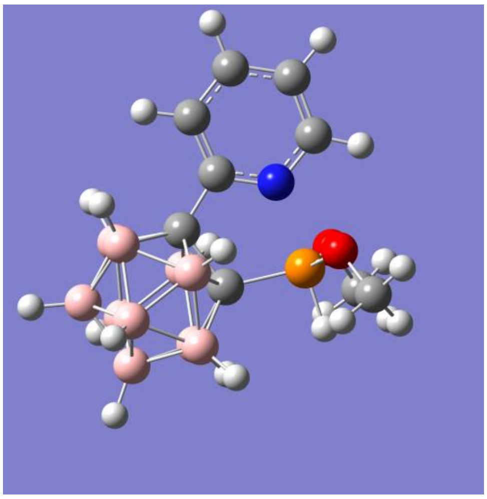

N	1.51180200	0.67714800	0.00001400
C	-0.73217400	-0.62819600	0.00018300
C	-0.83827600	0.96947700	0.00004700
C	0.51611400	1.57806500	0.00003100
C	0.76917700	2.94018000	0.00002400
H	-0.05491100	3.64004700	0.00008200
C	2.09442700	3.36394400	-0.00006000
H	2.32515100	4.42205700	-0.00009300
C	3.12015200	2.41955300	-0.00010100
H	4.15974200	2.71791100	-0.00016500
C	2.79381800	1.07448600	-0.00004400
H	3.53999800	0.29285800	0.00000400
B	-1.98535100	-1.36276000	0.89041400
H	-1.72969300	-2.36016500	1.46304700
B	-1.22913200	0.13344500	1.47893700
H	-0.42706100	0.19460400	2.33417300
B	-2.18065600	1.51386000	0.89178500
H	-2.06892900	2.53124800	1.47261100
B	-2.96946100	0.01122000	1.45354400
H	-3.55043600	-0.02846700	2.47703700
B	-3.45468100	-0.91353600	0.00003400
H	-4.39488900	-1.62229600	-0.00002000
B	-1.98518600	-1.36271100	-0.89005700

H	-1.72952600	-2.36010300	-1.46272700
B	-2.96937900	0.01109200	-1.45353300
H	-3.55022200	-0.02893300	-2.47708700
B	-3.57129300	0.86595300	-0.00007400
H	-4.60076600	1.43740800	-0.00002400
B	-2.18070100	1.51385800	-0.89189800
H	-2.06858100	2.53130000	-1.47255400
B	-1.22907400	0.13344200	-1.47876000
H	-0.42686200	0.19423400	-2.33388700
O	1.77395000	-1.30797900	-1.38950700
C	1.81741400	-2.49074100	-2.23809100
H	1.97519700	-3.39278900	-1.64613400
H	0.89068100	-2.56294600	-2.80604100
H	2.65849200	-2.33304100	-2.90776300
P	0.97330400	-1.28561600	0.00001800
H	0.58040600	-2.64716700	0.00008000
O	1.77431400	-1.30800400	1.38931300
C	1.81802600	-2.49079600	2.23790700
H	2.65977100	-2.33340500	2.90681600
H	0.89175600	-2.56251400	2.80666500
H	1.97490600	-3.39292300	1.64583700

Zero-point correction= 0.333355 (Hartree/Particle)

Thermal correction to Energy= 0.353289

Thermal correction to Enthalpy= 0.354233

Thermal correction to Gibbs Free Energy= 0.286659

Sum of electronic and zero-point Energies= -1150.594054

Sum of electronic and thermal Energies= -1150.574119

Sum of electronic and thermal Enthalpies= -1150.573175

Sum of electronic and thermal Free Energies= -1150.640749

At **B3LYP-D3(BJ)/6-311++G(d,p)** gas phase

Int4

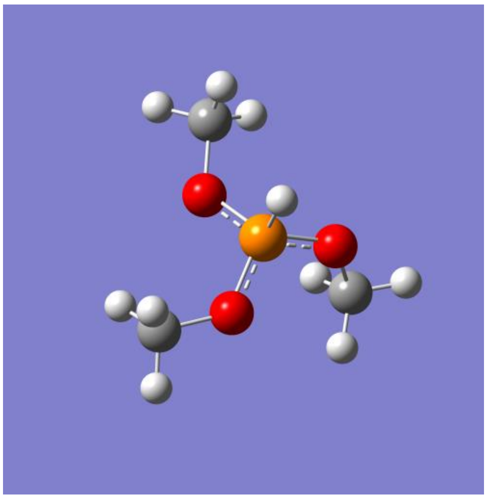

O	-1.36576100	-0.62475600	0.03637900
C	-2.61176200	0.07425900	-0.32458800
H	-2.39036600	0.83276100	-1.07187900
H	-3.25480700	-0.70074300	-0.72845000
H	-3.04740200	0.50726600	0.57428300
P	-0.03635400	0.00887900	0.52948600
H	-0.07557400	0.39086900	1.87264300
C	1.37804700	-2.00512400	-0.59868700
H	2.23220300	-2.57513600	-0.24855000
H	0.51410000	-2.64718000	-0.75398200
H	1.62570300	-1.44082200	-1.49596700
C	1.34464000	2.20567700	-0.21735300
H	2.27587000	1.69440900	-0.45381500
H	1.14476400	2.99643400	-0.93308500
H	1.35575600	2.60178900	0.79782900
O	1.07845400	-1.06896600	0.50065700
O	0.22474600	1.26350900	-0.36922900

Zero-point correction= 0.139485 (Hartree/Particle)

Thermal correction to Energy= 0.150150

Thermal correction to Enthalpy= 0.151094

Thermal correction to Gibbs Free Energy= 0.102002

Sum of electronic and zero-point Energies= -687.152409

Sum of electronic and thermal Energies= -687.141745

Sum of electronic and thermal Enthalpies= -687.140800

Sum of electronic and thermal Free Energies= -687.189893

At **B3LYP-D3(BJ)/6-311++G(d,p)** gas phase

Int5

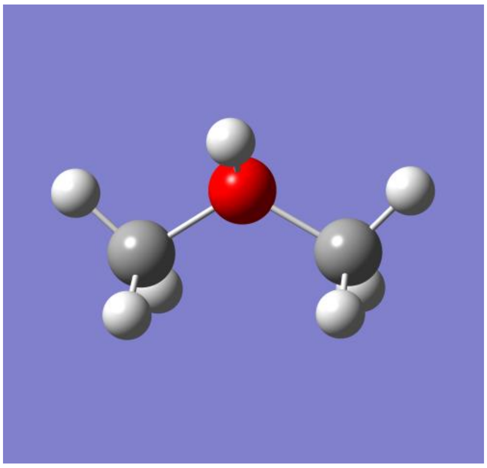

O	-0.00001400	0.51120400	-0.11286000
C	-1.28045200	-0.24957200	0.02312900
H	-2.07100200	0.47554500	-0.14988800
H	-1.25232700	-0.99768700	-0.76353900
H	-1.32047700	-0.68881200	1.01800600
H	0.00000600	1.32708000	0.41616000
C	1.28047600	-0.24958100	0.02313900
H	1.25227900	-0.99774700	-0.76349500
H	2.07096700	0.47556400	-0.15006300
H	1.32052400	-0.68866400	1.01809600

Zero-point correction= 0.092671 (Hartree/Particle)

Thermal correction to Energy= 0.097485

Thermal correction to Enthalpy= 0.098429

Thermal correction to Gibbs Free Energy= 0.066576

Sum of electronic and zero-point Energies= -155.305156

Sum of electronic and thermal Energies= -155.300342

Sum of electronic and thermal Enthalpies= -155.299398

Sum of electronic and thermal Free Energies= -155.331251

At **B3LYP-D3(BJ)/6-311++G(d,p)** gas phase

MeOH

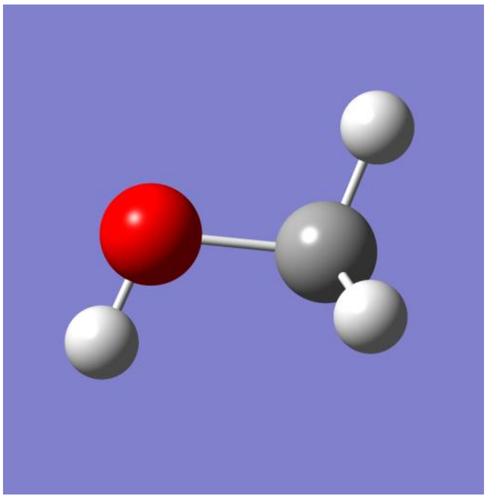

C	0.66772400	-0.02026000	0.00000000
H	1.08368700	0.98752300	-0.00000100
H	1.02826900	-0.54504000	-0.89320200
H	1.02826900	-0.54503800	0.89320300
O	-0.74979600	0.12211900	0.00000000
H	-1.14820400	-0.75283600	0.00000000

Zero-point correction= 0.051042 (Hartree/Particle)

Thermal correction to Energy= 0.054392

Thermal correction to Enthalpy= 0.055336

Thermal correction to Gibbs Free Energy= 0.028268

Sum of electronic and zero-point Energies= -115.717059

Sum of electronic and thermal Energies= -115.713710

Sum of electronic and thermal Enthalpies= -115.712766

Sum of electronic and thermal Free Energies= -115.739833

At **B3LYP-D3(BJ)/6-311++G(d,p)** gas phase

$(OMe)_2P(O)H$

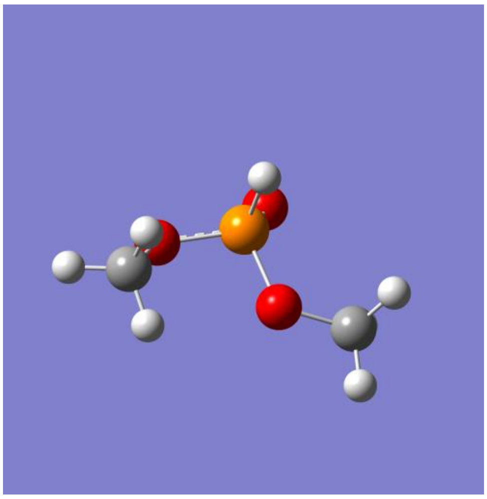

O	0.70708600	1.74223400	-0.23536700
P	0.02113600	0.53460600	0.25700900
H	-0.15287600	0.48572900	1.65660100
C	-2.21534500	-0.87467100	-0.00145000
H	-2.31194400	-0.99227500	1.08216500
H	-3.19994200	-0.72596000	-0.43897600
H	-1.73917000	-1.75769000	-0.42894600
C	2.16350300	-0.99680600	-0.07122400
H	2.39127300	-1.97861200	-0.48077200
H	2.60938000	-0.21638800	-0.68853000
H	2.54832000	-0.92717700	0.94973700
O	-1.45289300	0.30375700	-0.32128500
O	0.72692900	-0.88072200	-0.07714300

Zero-point correction= 0.099704 (Hartree/Particle)

Thermal correction to Energy= 0.107879

Thermal correction to Enthalpy= 0.108823

Thermal correction to Gibbs Free Energy= 0.066292

Sum of electronic and zero-point Energies= -647.529521

Sum of electronic and thermal Energies= -647.521346

Sum of electronic and thermal Enthalpies= -647.520402

Sum of electronic and thermal Free Energies= -647.562933

At **B3LYP-D3(BJ)/6-311++G(d,p)** gas phase

LH_2^+

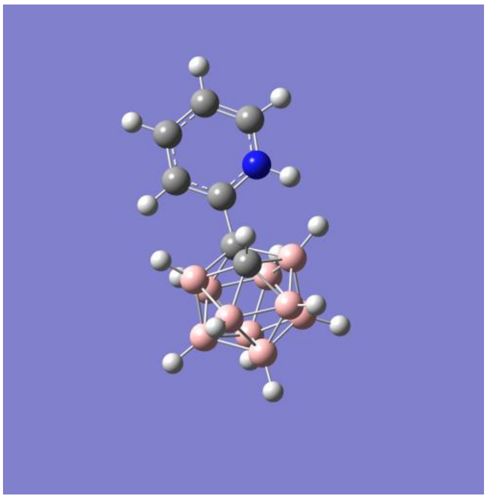

C	-0.95549800	-0.17854400	1.36108000
C	-0.07276200	0.04216400	-0.02141400
C	1.41707000	0.08183100	0.03075400
C	2.16512600	1.25009500	0.01472600
H	1.65437600	2.20125400	0.00698000
C	3.55603400	1.18037500	-0.00443800
H	4.13738400	2.09433000	-0.02014800
C	4.20051700	-0.05721700	-0.00980100
H	5.27880600	-0.13599100	-0.02708600
C	3.43052900	-1.20127600	0.00023100
H	3.83601300	-2.20377000	-0.00939400

B	-2.40696400	0.69255600	1.31997200
H	-2.79663200	1.13919000	2.33529600
B	-0.91778300	1.38996300	0.65501800
H	-0.27430400	2.20837200	1.19922600
B	-0.89119200	1.08015900	-1.10000800
H	-0.21839300	1.77565300	-1.77090600
B	-2.40726300	1.48830300	-0.27424700
H	-2.91802700	2.53659200	-0.43573300
B	-3.31344100	-0.03390200	-0.02679900
H	-4.49000300	-0.06539500	-0.00913700
B	-2.36854300	-1.07034100	1.06540400
H	-2.72857800	-1.80562400	1.90936300
B	-2.34337700	-1.38316300	-0.68978800
H	-2.80194000	-2.36906900	-1.14141700
B	-2.37514300	0.20320000	-1.52150100
H	-2.86348400	0.34555700	-2.58346200
B	-0.85157300	-0.68936000	-1.35827200
H	-0.14402700	-1.14229600	-2.18541900
B	-0.85846400	-1.47170100	0.23896100
H	-0.19306800	-2.40228200	0.53399000
H	-0.38387800	-0.29502300	2.27020300
N	2.08655700	-1.09569600	0.01961600
H	1.52246300	-1.94476100	0.01980500

Zero-point correction= 0.258406 (Hartree/Particle)

Thermal correction to Energy= 0.271039

Thermal correction to Enthalpy= 0.271983

Thermal correction to Gibbs Free Energy= 0.220358

Sum of electronic and zero-point Energies= -579.503754

Sum of electronic and thermal Energies= -579.491121

Sum of electronic and thermal Enthalpies= -579.490177

Sum of electronic and thermal Free Energies= -579.541802

At **B3LYP-D3(BJ)/6-311++G(d,p)** gas phase

LH

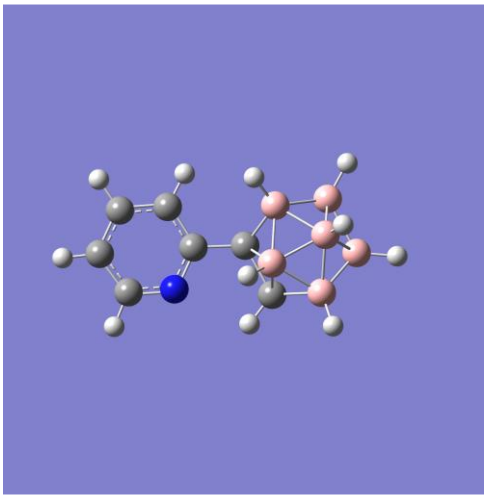

N	-2.00559900	-1.15273000	-0.00005700
C	0.83284300	-1.31211500	0.00090800
C	0.04865400	0.11446200	-0.00009300
C	-1.45284600	0.06133800	-0.00018400
C	-2.20007900	1.23856400	-0.00021200
H	-1.70741100	2.20049100	-0.00035500
C	-3.58638600	1.13712300	-0.00001500
H	-4.19813400	2.03140200	0.00001700
C	-4.17251600	-0.12504900	0.00012600
H	-5.24823400	-0.24760300	0.00030600
C	-3.33795000	-1.23861100	0.00010300
H	-3.75049800	-2.24248100	0.00017500
B	2.27250300	-1.25666100	-0.88812100
H	2.60039700	-2.22503200	-1.47365000
B	0.82718200	-0.40361800	-1.45361700
H	0.12977100	-0.76785700	-2.32607900
B	0.92343300	1.27632700	-0.88923900
H	0.30695000	2.08910500	-1.47901000
B	2.37818700	0.42567300	-1.44962100

H	2.89281200	0.70736900	-2.47384300
B	3.28334900	-0.09872500	0.00022200
H	4.45992400	-0.19559700	0.00051900
B	2.27244700	-1.25520100	0.89013500
H	2.60068100	-2.22239900	1.47740800
B	2.37793700	0.42787600	1.44904900
H	2.89226400	0.71098100	2.47304200
B	2.44376200	1.47021000	-0.00101100
H	3.01311800	2.50481900	-0.00179000
B	0.92343000	1.27783600	0.88729400
H	0.30693500	2.09149500	1.47581200
B	0.82677700	-0.40141000	1.45399900
H	0.12938700	-0.76442800	2.32698200
H	0.17588100	-2.16695600	0.00160600

Zero-point correction= 0.244939 (Hartree/Particle)

Thermal correction to Energy= 0.257341

Thermal correction to Enthalpy= 0.258285

Thermal correction to Gibbs Free Energy= 0.207449

Sum of electronic and zero-point Energies= -579.163913

Sum of electronic and thermal Energies= -579.151512

Sum of electronic and thermal Enthalpies= -579.150568

Sum of electronic and thermal Free Energies= -579.201404

At **B3LYP-D3(BJ)/6-311++G(d,p)** gas phase

MeOMe

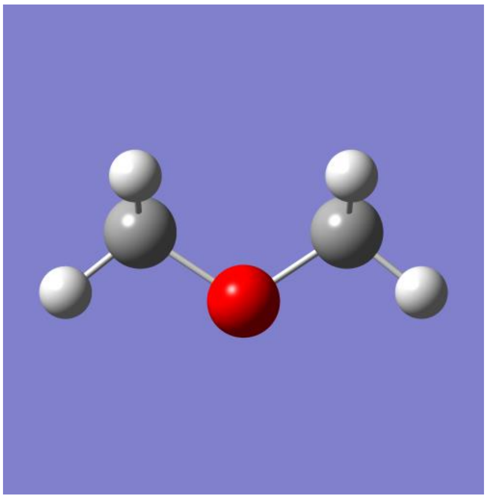

C	1.17458100	0.19549200	0.00003000
H	2.02259800	-0.48943200	-0.00000600
H	1.22961500	0.83562700	0.89250200
H	1.22963900	0.83575300	-0.89235100
O	0.00005700	-0.58874700	-0.00004200
C	-1.17468500	0.19554300	-0.00000200
H	-1.22945900	0.83589800	-0.89270000
H	-2.02271600	-0.48980900	-0.00009000
H	-1.22951000	0.83573200	0.89281200

Zero-point correction= 0.079268 (Hartree/Particle)

Thermal correction to Energy= 0.083614

Thermal correction to Enthalpy= 0.084558

Thermal correction to Gibbs Free Energy= 0.053843

Sum of electronic and zero-point Energies= -155.004613

Sum of electronic and thermal Energies= -155.000268

Sum of electronic and thermal Enthalpies= -154.999324

Sum of electronic and thermal Free Energies= -155.030039

Cartesian Coordinates for FIA calculations.

L_2P^+ at wb97xd/def2tzvp in $CHCl_3$

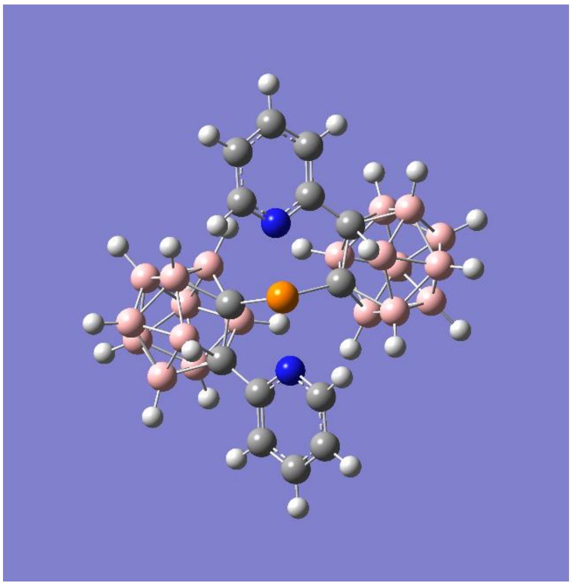

P	-0.00003200	0.00018600	-1.16219800
N	-1.24035800	1.64607300	-1.01628000
N	1.24012400	-1.64596400	-1.01633900
C	-1.33864000	-0.81064100	-0.04903900
C	1.33872200	0.81077200	-0.04904400
C	2.59486400	-0.18405900	0.25371200
C	2.36585600	-1.54630900	-0.29588700
C	3.26695200	-2.59220000	-0.19165000
H	4.16852100	-2.48124000	0.40964400
C	2.99689300	-3.76235400	-0.89958200
H	3.68402400	-4.60734000	-0.83775000
C	1.87344700	-3.82378500	-1.72051900
H	1.65964800	-4.70148300	-2.32957000
C	1.01796400	-2.73349600	-1.76430800
H	0.14527300	-2.71341500	-2.41643900
C	-2.59490500	0.18400400	0.25377100
C	-2.36607500	1.54628800	-0.29581900
C	-3.26726800	2.59209000	-0.19155300
H	-4.16880400	2.48104400	0.40977400
C	-2.99728900	3.76230900	-0.89941500
H	-3.68445100	4.60726300	-0.83748400

C	-1.87382400	3.82389000	-1.72032200
H	-1.66006400	4.70165600	-2.32928800
C	-1.01827100	2.73366200	-1.76419500
H	-0.14557700	2.71368400	-2.41633200
B	-1.19967600	-1.96722200	1.20603000
H	-0.11556000	-2.38439700	1.47961400
B	-1.62776800	-0.28121100	1.58572100
H	-0.94049800	0.55641500	2.05631000
B	-3.39310600	-0.16198500	1.71588300
H	-3.82086100	0.76466100	2.33771900
B	-2.52355500	-1.58652000	2.34795400
H	-2.39534900	-1.76634900	3.52481200
B	-2.71938000	-2.89733200	1.15849000
H	-2.72538200	-4.05218000	1.47809700
B	-1.93866300	-2.40034300	-0.34949100
H	-1.37317300	-3.11388000	-1.11206400
B	-3.70584400	-2.29008900	-0.20080600
H	-4.42030700	-2.98313100	-0.86662100
B	-4.07178600	-1.78254000	1.47132000
H	-5.07927100	-2.11265700	2.02933800
B	-4.11199400	-0.59473100	0.14913500
H	-5.02605000	0.04251700	-0.28291100
B	-2.80443300	-0.99915100	-0.98809600
H	-2.76703400	-0.61399100	-2.11703700
B	1.62786400	0.28127800	1.58570200
H	0.94049000	-0.55625100	2.05633600
B	1.19997100	1.96735200	1.20604100
H	0.11592600	2.38464800	1.47969600
B	1.93893900	2.40040400	-0.34949800
H	1.37351800	3.11401000	-1.11206500
B	2.71980100	2.89726300	1.15844500

H	2.72598400	4.05210200	1.47807900
B	2.52385900	1.58646400	2.34789700
H	2.39573100	1.76630000	3.52476200
B	3.39319200	0.16181800	1.71577200
H	3.82085700	-0.76488300	2.33758800
B	4.11205400	0.59448300	0.14898800
H	5.02599500	-0.04289000	-0.28311600
B	4.07207300	1.78228700	1.47118900
H	5.07962800	2.11226200	2.02916400
B	3.70611400	2.28990300	-0.20090800
H	4.42063500	2.98285400	-0.86675500
B	2.80448700	0.99909600	-0.98816500
H	2.76697800	0.61395800	-2.11711000

Zero-point correction= 0.478137 (Hartree/Particle)

Thermal correction to Energy= 0.502695

Thermal correction to Enthalpy= 0.503639

Thermal correction to Gibbs Free Energy= 0.429934

Sum of electronic and zero-point Energies= -1497.945487

Sum of electronic and thermal Energies= -1497.920928

Sum of electronic and thermal Enthalpies= -1497.919984

Sum of electronic and thermal Free Energies= -1497.993689

L_2P_F at wb97xd/def2tzvp in $CHCl_3$

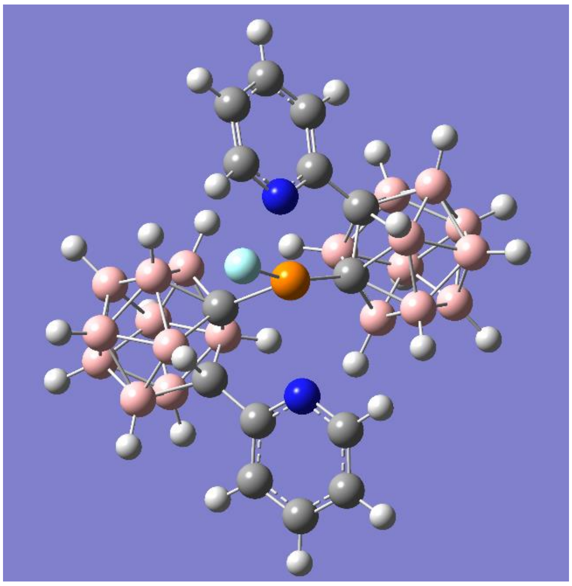

N	-1.71353700	1.71488900	-1.09489000
N	1.48798000	-1.56525300	-0.94160500
C	-1.22546700	-0.90973500	-0.07541600
C	1.30505200	0.94429500	0.03471800
C	2.68092300	0.15303000	0.26385700
C	2.56891100	-1.25874300	-0.18652100
C	3.54729800	-2.19789500	0.09568500
H	4.39904300	-1.90653500	0.70829700
C	3.42366200	-3.48555700	-0.41544300
H	4.17892300	-4.24053300	-0.19354600
C	2.33765400	-3.77954800	-1.23060500
H	2.20846900	-4.76164900	-1.68388200
C	1.39909900	-2.79381500	-1.48396100
H	0.54888100	-2.95386300	-2.13985400
C	-2.58302600	-0.01378200	0.31321400
C	-2.67631900	1.37572800	-0.24938700
C	-3.72686200	2.23163500	0.07894000
H	-4.50093900	1.92495500	0.78107000
C	-3.75610900	3.48662200	-0.52317200
H	-4.56049500	4.18596200	-0.28912000
C	-2.75566300	3.83290600	-1.42838400

H	-2.75208100	4.80175100	-1.92843500
C	-1.74923100	2.90639400	-1.68439000
H	-0.94304600	3.12578100	-2.38906800
B	-0.84827000	-1.94562900	1.23592500
H	0.29818600	-2.21751200	1.43246400
B	-1.41812800	-0.29365000	1.52795900
H	-0.78776000	0.63482400	1.88718200
B	-3.15449700	-0.36401500	1.88081200
H	-3.61082400	0.54202200	2.51390100
B	-2.06822900	-1.62612600	2.49097300
H	-1.79513000	-1.69830500	3.65683900
B	-2.24080000	-3.03706800	1.42030000
H	-2.07885100	-4.16112000	1.80975000
B	-1.68611200	-2.56641700	-0.19448900
H	-1.13636900	-3.28700800	-0.96297300
B	-3.42336000	-2.63423500	0.14745900
H	-4.12550500	-3.44632900	-0.38766400
B	-3.66692300	-2.05762800	1.81917300
H	-4.56571400	-2.45005300	2.51120000
B	-3.98215700	-0.98053200	0.44258000
H	-5.01384400	-0.49480600	0.08072300
B	-2.75883400	-1.29751100	-0.81072700
H	-2.94025400	-1.01005200	-1.95043100
B	1.79501700	0.58040900	1.65222100
H	1.25481000	-0.29016800	2.25425400
B	1.14405500	2.16797500	1.22625400
H	0.05693200	2.48976800	1.60397200
B	1.66014700	2.56823600	-0.41210000
H	0.93029700	3.13801300	-1.16686500
B	2.51652900	3.27282000	0.97870000
H	2.41775200	4.44201600	1.23718500

B	2.60054300	2.03032400	2.25434900
H	2.56775600	2.27433000	3.42907300
B	3.57225100	0.67955800	1.62554600
H	4.18334800	-0.12872700	2.26313900
B	4.09176000	1.08941400	-0.02563100
H	5.04220500	0.53835800	-0.50191200
B	4.02315400	2.34796300	1.22297900
H	5.03133400	2.83156100	1.66139400
B	3.44428500	2.69638700	-0.42807300
H	4.01555600	3.42177200	-1.19510300
B	2.63814500	1.25838900	-1.03416900
H	2.57238500	0.80673400	-2.13962200
P	0.10060200	-0.20442800	-1.35240900
F	-0.52862300	-1.38719400	-2.50171000

Zero-point correction= 0.479273 (Hartree/Particle)

Thermal correction to Energy= 0.505365

Thermal correction to Enthalpy= 0.506309

Thermal correction to Gibbs Free Energy= 0.429179

Sum of electronic and zero-point Energies= -1597.970654

Sum of electronic and thermal Energies= -1597.944562

Sum of electronic and thermal Enthalpies= -1597.943618

Sum of electronic and thermal Free Energies= -1598.020748

(V) at wb97xd/def2tzvp in $CHCl_3$

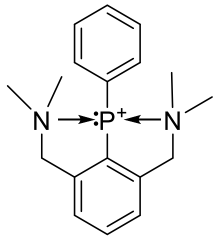

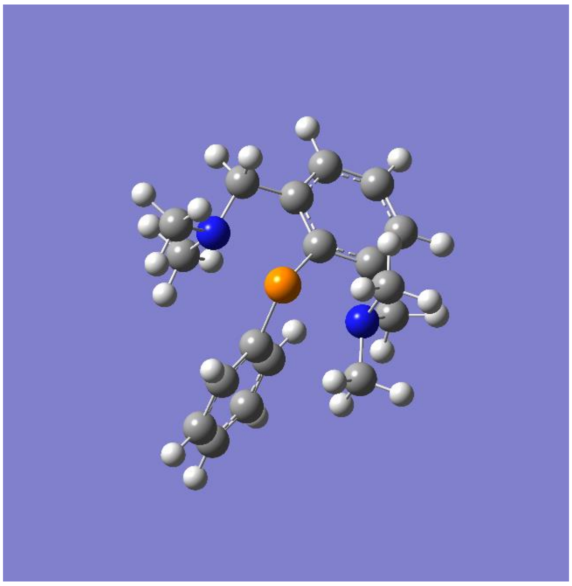

C	-3.61277200	-0.13870400	1.64769000
C	-3.25370800	1.06665200	1.03803800
C	-2.12064000	1.11612800	0.22887300
C	-1.35586800	-0.03641600	0.06124000
C	-1.72555200	-1.24843200	0.64081500
C	-2.86388300	-1.29975400	1.44458500
H	-4.49974600	-0.17577000	2.28321900
H	-3.86184800	1.96108300	1.18943600
H	-3.16947300	-2.23932200	1.90983100
C	-0.81915600	-2.40758800	0.32238500
H	-0.05620500	-2.52503600	1.10702100
H	-1.36534900	-3.36135500	0.25041100
C	-1.66236500	2.30849700	-0.56939100
H	-2.12551100	2.27027600	-1.56810900
H	-1.95470400	3.26628500	-0.10992800
N	-0.19652600	2.22296600	-0.73344100
N	-0.13117700	-2.10684900	-0.95274600
C	0.47394200	2.79138900	0.44481300
H	1.55726400	2.64070700	0.36824100
H	0.26312100	3.87115300	0.51587400
H	0.11086100	2.29802500	1.35548400
C	0.27195200	2.88218700	-1.95781100

H	0.03587800	3.95855100	-1.94148600
H	1.36029000	2.75911500	-2.03980600
H	-0.20090300	2.41835600	-2.83302400
C	-1.00793600	-2.40579100	-2.10217400
H	-0.51378300	-2.08593000	-3.02800400
H	-1.21419600	-3.48583500	-2.15152700
H	-1.95753800	-1.86430500	-1.99910900
C	1.13993900	-2.83392200	-1.08791700
H	0.95440400	-3.91748700	-1.14686000
H	1.64786000	-2.50798000	-2.00538700
H	1.78609100	-2.62284500	-0.22788300
P	0.09431400	0.02290700	-1.03392500
C	1.54421300	-0.00204200	0.08457400
C	1.46771400	-0.16522400	1.47399000
C	2.79914000	0.15081400	-0.51931300
C	2.62872000	-0.18185100	2.24325600
H	0.49637000	-0.26749200	1.96391900
C	3.96041900	0.14162700	0.25290400
H	2.87313100	0.27656200	-1.60303500
C	3.87530600	-0.02739100	1.63421800
H	2.55937200	-0.31145000	3.32504500
H	4.93327400	0.26477200	-0.22684200
H	4.78376800	-0.03758200	2.24005200

Zero-point correction= 0.391374 (Hartree/Particle)

Thermal correction to Energy= 0.409858

Thermal correction to Enthalpy= 0.410803

Thermal correction to Gibbs Free Energy= 0.347306

Sum of electronic and zero-point Energies= -1150.712799

Sum of electronic and thermal Energies= -1150.694315

Sum of electronic and thermal Enthalpies= -1150.693371

Sum of electronic and thermal Free Energies= -1150.756867

(V-F) at wb97xd/def2tzvp in $CHCl_3$

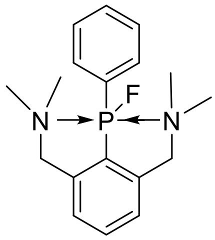

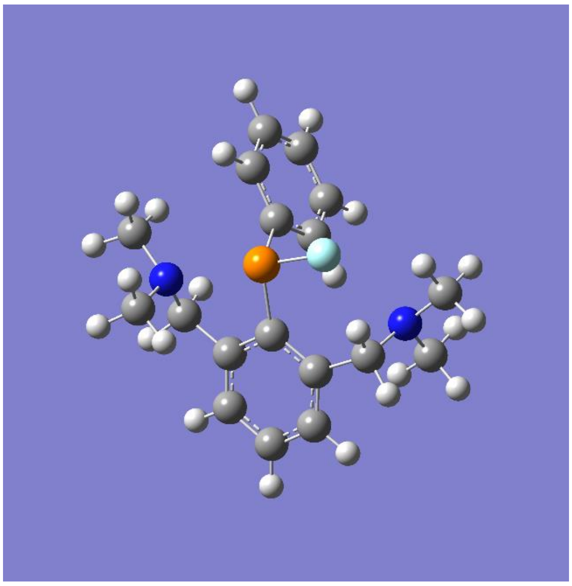

C	-1.93588900	-2.89557400	1.44778500
C	-2.61491100	-2.01518700	0.61682200
C	-1.94801200	-1.07245200	-0.17794900
C	-0.53050600	-1.03254200	-0.16893200
C	0.14750900	-1.90846200	0.71476000
C	-0.55093000	-2.82431100	1.50326200
H	-2.48250600	-3.61927500	2.05576500
H	-3.70737600	-2.03793400	0.58529200
H	0.00822500	-3.49110900	2.16430600
C	1.64648500	-1.84388200	0.87274000
H	1.89309600	-0.99501900	1.53343500
H	2.01839400	-2.75943100	1.37662500
C	-2.83725600	-0.08359500	-0.90921900
H	-2.47896900	0.08191800	-1.92857800
H	-3.85493300	-0.52599700	-0.99237900
N	-2.88108900	1.20172300	-0.22776700

N	2.29944700	-1.61800100	-0.39705300
C	-3.61305300	1.16542400	1.01648300
H	-3.54535200	2.14280200	1.51785500
H	-4.69304900	0.92724600	0.87849800
H	-3.18919300	0.41186900	1.69662800
C	-3.32768600	2.26861600	-1.09368900
H	-4.37527700	2.13491800	-1.44698500
H	-3.27735200	3.23023100	-0.56018900
H	-2.66988500	2.33245400	-1.97109700
C	2.17811100	-2.74247300	-1.30829000
H	2.59234300	-2.46809300	-2.28885800
H	2.71541500	-3.63652500	-0.93405800
H	1.12099900	-3.00865900	-1.44944900
C	3.67848200	-1.19262700	-0.25764500
H	4.32148800	-1.99874900	0.14937700
H	4.07950000	-0.89686800	-1.23836700
H	3.74063400	-0.32735500	0.41600200
C	1.21283700	1.23065500	-0.16317000
C	0.48352700	1.66114200	0.95327000
C	2.44576500	1.83192400	-0.44028800
C	0.99497400	2.64947000	1.79220600
H	-0.50384800	1.23386800	1.14483500
C	2.95973700	2.82153600	0.39868400
H	3.01384100	1.52228900	-1.32166300
C	2.23719700	3.22593000	1.52085100
H	0.41937500	2.97505400	2.66178500
H	3.92594300	3.27878100	0.17415100
H	2.63843200	3.99780500	2.18159600
P	0.53964700	-0.01627100	-1.34298700
F	-0.61016000	1.02135900	-1.97689700

Zero-point correction= 0.389317 (Hartree/Particle)

Thermal correction to Energy= 0.410115

Thermal correction to Enthalpy= 0.411059

Thermal correction to Gibbs Free Energy= 0.341650

Sum of electronic and zero-point Energies= -1250.736086

Sum of electronic and thermal Energies= -1250.715288

Sum of electronic and thermal Enthalpies= -1250.714343

Sum of electronic and thermal Free Energies= -1250.783753

(IV) at wb97xd/def2tzvp in $CHCl_3$

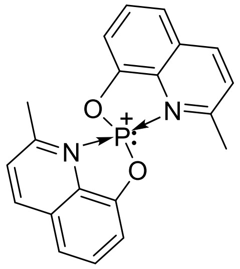

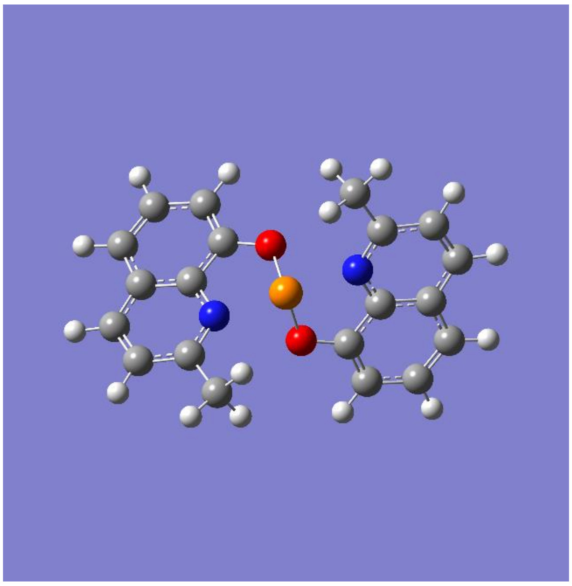

C	-2.51860300	0.30080700	-0.16584000
C	-1.67296100	1.35677700	-0.55552300
O	-0.40635100	1.26772900	-0.08014600
P	0.00000800	0.00001200	0.96206200
C	1.67295300	-1.35682500	-0.55548700
O	0.40635600	-1.26776500	-0.08007700
C	-3.86317800	0.22523600	-0.56074800
C	-4.35785600	1.26505500	-1.39270700
C	-3.51760400	2.29066900	-1.77096300

C	-2.15948900	2.35759900	-1.35759600
N	-1.91234800	-0.61145700	0.62639000
C	-2.57190000	-1.65888800	1.09739400
C	-3.94263400	-1.80756700	0.75573700
C	-4.57699900	-0.89469200	-0.05643800
C	2.51859900	-0.30081800	-0.16590200
C	3.86315300	-0.22524800	-0.56088100
C	4.35780800	-1.26510800	-1.39280200
C	3.51755600	-2.29075700	-1.77095800
C	2.15946200	-2.35768500	-1.35752400
N	1.91237400	0.61148100	0.62631200
C	2.57193400	1.65895400	1.09721800
C	3.94264600	1.80763600	0.75547900
C	4.57698200	0.89472200	-0.05667400
C	1.83964500	2.65648200	1.93577400
C	-1.83958100	-2.65638400	1.93596100
H	-5.39842700	1.24612400	-1.72020700
H	-3.90098000	3.08775100	-2.41047900
H	-1.51733800	3.18208900	-1.66857400
H	-4.48442900	-2.66825400	1.14890900
H	-5.63061400	-1.02696300	-0.31100200
H	5.39836400	-1.24617900	-1.72035400
H	3.90091400	-3.08786800	-2.41044700
H	1.51730900	-3.18220000	-1.66843100
H	4.48445000	2.66835200	1.14857400
H	5.63058300	1.02699300	-0.31129800
H	2.53867400	3.29938800	2.48280200
H	1.21103800	3.29084800	1.29239400
H	1.17767100	2.15210000	2.65459100
H	-1.21107600	-3.29084500	1.29257400
H	-2.53859100	-3.29920600	2.48311400
H	-1.17750800	-2.15197700	2.65466800

Zero-point correction= 0.319479 (Hartree/Particle)

Thermal correction to Energy= 0.338341

Thermal correction to Enthalpy= 0.339286

Thermal correction to Gibbs Free Energy= 0.273851

Sum of electronic and zero-point Energies= -1372.696657

Sum of electronic and thermal Energies= -1372.677794

Sum of electronic and thermal Enthalpies= -1372.676850

Sum of electronic and thermal Free Energies= -1372.742285

(IV-F) at wb97xd/def2tzvp in $CHCl_3$

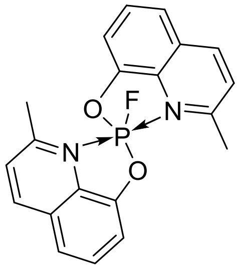

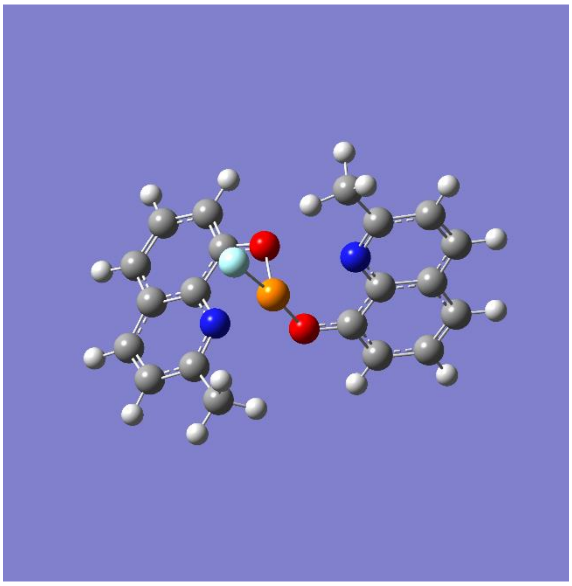

C	-2.47869800	-0.22852300	0.34455000
C	-1.56980900	-1.10387600	0.98086300
O	-0.37414300	-1.18863400	0.40397700
C	1.51315900	1.42360600	0.40937100
O	0.26507200	1.20645000	0.06431500
C	-3.77931300	0.01231000	0.81083300
C	-4.18840000	-0.67932600	1.98173000

C	-3.30166100	-1.53782100	2.59899800
C	-1.98648900	-1.76492700	2.11628800
N	-1.96049400	0.36268200	-0.75710400
C	-2.65067900	1.23358300	-1.47539100
C	-3.97799900	1.52995700	-1.07038000
C	-4.53467800	0.93587500	0.04030200
C	2.52303900	0.41895500	0.18864500
C	3.87230400	0.64812400	0.56395100
C	4.23438900	1.87881500	1.16748700
C	3.26071500	2.83347300	1.37401800
C	1.91824000	2.61903000	1.00017700
N	2.12630200	-0.74024100	-0.39361100
C	2.97758000	-1.70681300	-0.64443400
C	4.35548900	-1.55810900	-0.31358600
C	4.78988900	-0.40314100	0.28286400
C	2.45573600	-2.97484400	-1.25881800
C	-2.00298300	1.86614700	-2.66508200
H	-5.19192600	-0.52507000	2.38091900
H	-3.61631500	-2.06841000	3.50006200
H	-1.31099500	-2.44675700	2.63386700
H	-4.55224500	2.24308500	-1.66224900
H	-5.55926700	1.17538000	0.33308100
H	5.27366400	2.05646300	1.45115200
H	3.52860600	3.78635500	1.83758300
H	1.16461600	3.39033600	1.17200300
H	5.05103500	-2.36989200	-0.53579800
H	5.84341600	-0.27456000	0.54391500
H	3.09298700	-3.30159500	-2.09350800
H	2.45147800	-3.78472400	-0.51236200
H	1.42854900	-2.83096000	-1.61698900
H	-1.09487300	2.40240600	-2.35326600
H	-2.68664200	2.56857700	-3.15494000

H	-1.70186700	1.09506000	-3.38942600
P	-0.18263200	-0.28800400	-1.07385900
F	-0.91642300	-1.66113500	-1.85686900

Zero-point correction= 0.319811 (Hartree/Particle)

Thermal correction to Energy= 0.340434

Thermal correction to Enthalpy= 0.341379

Thermal correction to Gibbs Free Energy= 0.272125

Sum of electronic and zero-point Energies= -1472.714150

Sum of electronic and thermal Energies= -1472.693526

Sum of electronic and thermal Enthalpies= -1472.692582

Sum of electronic and thermal Free Energies= -1472.761836

(I) at wb97xd/def2tzvp in $CHCl_3$

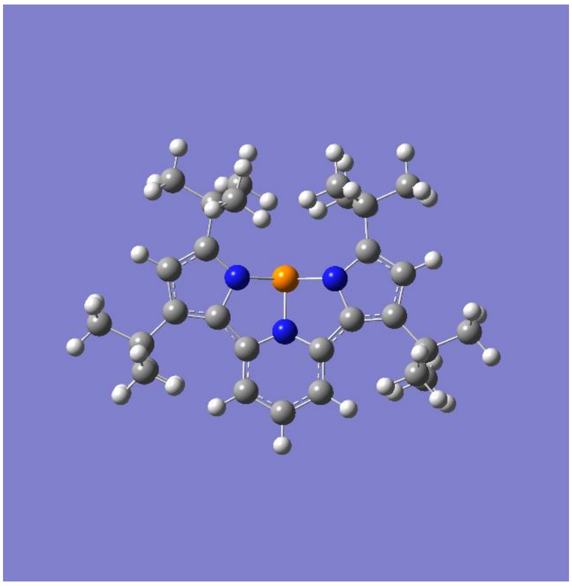

C	-1.31176500	-3.21771500	-0.10756100
C	-0.09966000	-3.90991800	-0.01874900
C	1.13332800	-3.24936500	0.02926600
C	1.16150100	-1.86045700	-0.14653700
C	-1.27670000	-1.82616700	-0.25837700
H	-2.25818500	-3.73535300	0.01426600
H	-0.12057000	-4.99337800	0.10835600
H	2.04518100	-3.79470800	0.24925900
C	-2.22873000	-0.78697600	-0.08356800

C	-3.61850500	-0.61697700	0.00727100
N	-1.59282000	0.50478400	0.02406000
C	-3.81817700	0.77257800	0.17943600
C	-2.60196300	1.45004800	0.17755100
H	-4.77866700	1.25397200	0.32508400
C	2.15228500	-0.85725400	0.02878900
C	3.54191400	-0.75582100	0.18232900
N	1.60410800	0.46424000	-0.12587200
C	3.83115100	0.62867100	0.10830700
C	2.67683100	1.36903600	-0.10982300
H	4.81954800	1.06319100	0.20226000
P	-0.01751800	0.45793600	-0.87120700
N	-0.03568600	-1.26527400	-0.49603300
C	2.58636300	2.86614500	-0.30872800
C	1.70785700	3.49127800	0.79241500
H	1.59438400	4.57201600	0.62322200
H	0.70735300	3.05148700	0.81439000
H	2.16058500	3.34013500	1.78292100
C	2.03205100	3.16336600	-1.72304900
H	1.05426400	2.69851400	-1.91351900
H	1.91085400	4.24805400	-1.85742600
H	2.72592900	2.79339400	-2.49135400
C	3.99075400	3.49652200	-0.21412100
H	4.66951300	3.10395400	-0.98480700
H	3.90555400	4.58156400	-0.36716300
H	4.44419500	3.33455000	0.77448300
C	-2.36660600	2.91603900	0.45409100
C	-1.68651500	3.03918700	1.84105700
H	-0.78764100	2.41316400	1.91979000
H	-1.39945100	4.08294900	2.03569300
H	-2.38048200	2.71792700	2.63086100
C	-1.50728300	3.52586400	-0.66801800

H	-2.04608300	3.51087500	-1.62616000
H	-1.23808600	4.56488600	-0.42943400
H	-0.56262500	2.98297900	-0.81463400
C	-3.70602200	3.67754500	0.49913800
H	-4.25334100	3.59135000	-0.45074800
H	-4.35265600	3.31819400	1.31238100
H	-3.50937100	4.74380800	0.68005800
C	4.56835600	-1.86530500	0.35018400
C	4.27347000	-2.64626200	1.65315000
H	3.25612500	-3.05755900	1.68258300
H	4.98205800	-3.48103100	1.75875000
H	4.38534200	-1.99015200	2.52865000
C	4.54072300	-2.79806900	-0.88363200
H	5.26067100	-3.61845200	-0.74784900
H	3.55348900	-3.24217700	-1.06347200
H	4.82288000	-2.24317300	-1.79022000
C	5.98949100	-1.27742400	0.46467000
H	6.27544400	-0.72093100	-0.44012800
H	6.08686600	-0.61091100	1.33437400
H	6.71247200	-2.09560900	0.59234600
C	-4.71397100	-1.66986500	-0.06195600
C	-6.10343300	-1.00151400	-0.07450100
H	-6.88085400	-1.77564400	-0.14429800
H	-6.29021300	-0.42990100	0.84656300
H	-6.22253400	-0.32854600	-0.93656400
C	-4.57345200	-2.49886500	-1.35925400
H	-4.69404700	-1.85496400	-2.24267900
H	-3.59941200	-2.99755100	-1.44480500
H	-5.35230500	-3.27469100	-1.39435600
C	-4.63196100	-2.57585600	1.19023800
H	-3.65340700	-3.06209200	1.29745000
H	-4.80728500	-1.98877900	2.10347700

H	-5.39995600	-3.36147600	1.13532500

Zero-point correction= 0.647545 (Hartree/Particle)

Thermal correction to Energy= 0.678572

Thermal correction to Enthalpy= 0.679516

Thermal correction to Gibbs Free Energy= 0.592489

Sum of electronic and zero-point Energies= -1634.621532

Sum of electronic and thermal Energies= -1634.590505

Sum of electronic and thermal Enthalpies= -1634.589561

Sum of electronic and thermal Free Energies= -1634.676588

(I-F) at wb97xd/def2tzvp in $CHCl_3$

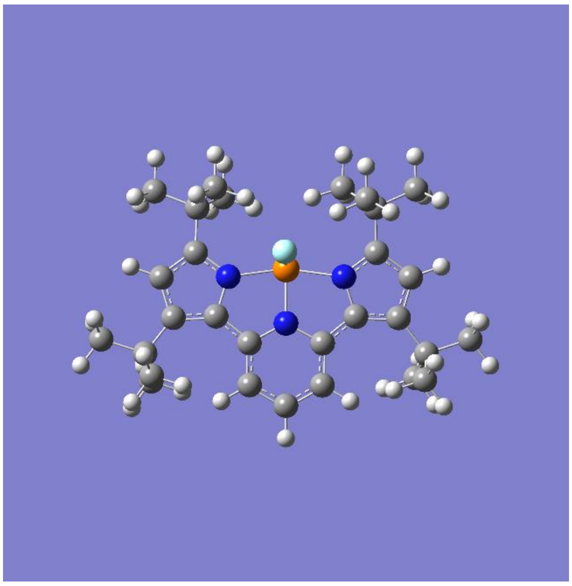

C	-1.22006600	-3.08563200	0.16382200
C	-0.00137300	-3.77528400	0.14534000
C	1.21807000	-3.09268000	0.06623700
C	1.22940500	-1.68192400	0.00942900
C	-1.23003600	-1.67623400	0.08648500
H	-2.16714000	-3.62782500	0.23585500
H	-0.00235900	-4.87497600	0.20225100
H	2.16612700	-3.63832300	0.07033500
C	-2.34577300	-0.77696900	0.05496100
C	-3.77774800	-0.82702200	0.02470400

N	-1.93530200	0.54766400	-0.06098700
C	-4.17897500	0.52166300	-0.12891900
C	-3.03316500	1.35212500	-0.18794500
H	-5.21111600	0.87953000	-0.18705600
C	2.34616700	-0.78534000	-0.01922500
C	3.77557200	-0.83342100	-0.11899500
N	1.93997800	0.54435900	0.06399600
C	4.18065600	0.52185800	-0.08793500
C	3.03818600	1.35321000	0.02730400
H	5.21278600	0.88314600	-0.12763000
N	-0.00037900	-1.01827800	0.00399500
C	2.99844000	2.86221700	0.23056100
C	2.71928200	3.14796900	1.73088800
H	2.68794500	4.24307300	1.91784400
H	1.75142800	2.70647300	2.03606100
H	3.51522000	2.70890000	2.36722500
C	1.89749800	3.52150300	-0.63047100
H	0.88258400	3.20047500	-0.31892000
H	1.93290500	4.62515100	-0.51589400
H	2.01646700	3.27592200	-1.70561300
C	4.36226600	3.47550800	-0.15821200
H	4.62266100	3.25093500	-1.21344900
H	4.33067300	4.57818900	-0.03820300
H	5.18064200	3.09602300	0.48743300
C	-2.97918600	2.86485500	-0.36483600
C	-2.38205800	3.53624500	0.89809600
H	-1.35119100	3.19025900	1.09623000
H	-2.36582900	4.64005100	0.77388100
H	-2.99073500	3.29672500	1.79449900
C	-2.11945900	3.21484600	-1.60546400
H	-2.54873700	2.76395700	-2.52402100
H	-2.07365100	4.31571000	-1.74408400

H	-1.07927700	2.84231600	-1.50493300
C	-4.40586700	3.41400200	-0.58632400
H	-4.88234100	2.96466900	-1.48215500
H	-5.05872000	3.21826700	0.28938100
H	-4.36716100	4.51265400	-0.73734200
C	4.71384900	-2.03116000	-0.26408800
C	4.65280100	-2.91232600	1.01117200
H	3.62673900	-3.25579500	1.24597200
H	5.29814000	-3.80989600	0.89900500
H	5.00832200	-2.33848300	1.89160800
C	4.34540500	-2.84549000	-1.53112400
H	5.00163400	-3.73683200	-1.62796500
H	3.29488800	-3.19461500	-1.52592800
H	4.47526000	-2.22109100	-2.43900600
C	6.17676100	-1.55741100	-0.42806500
H	6.30043500	-0.91814800	-1.32633200
H	6.52426600	-0.98424700	0.45600200
H	6.84709100	-2.43478600	-0.54276900
C	-4.72518800	-2.02278000	0.11521100
C	-6.19783000	-1.55078100	0.09003000
H	-6.87629500	-2.42563300	0.17098500
H	-6.42284000	-0.87034100	0.93693800
H	-6.44342900	-1.02221500	-0.85399500
C	-4.51818200	-2.96071200	-1.10274800
H	-4.77429600	-2.43013300	-2.04283700
H	-3.47172200	-3.30730300	-1.20399100
H	-5.16981800	-3.85722900	-1.02304900
C	-4.50385600	-2.77346000	1.45389300
H	-3.45743800	-3.10757000	1.59079500
H	-4.74394500	-2.10921000	2.30946000
H	-5.16016100	-3.66792100	1.51547600
P	0.00542800	0.80389100	-0.20887500

F	-0.04256600	1.38681100	1.34659300

Zero-point correction= 0.643385 (Hartree/Particle)

Thermal correction to Energy= 0.674770

Thermal correction to Enthalpy= 0.675714

Thermal correction to Gibbs Free Energy= 0.588065

Sum of electronic and zero-point Energies= -1734.728649

Sum of electronic and thermal Energies= -1734.697264

Sum of electronic and thermal Enthalpies= -1734.696320

Sum of electronic and thermal Free Energies= -1734.783969

(III) at wb97xd/def2tzvp in $CHCl_3$

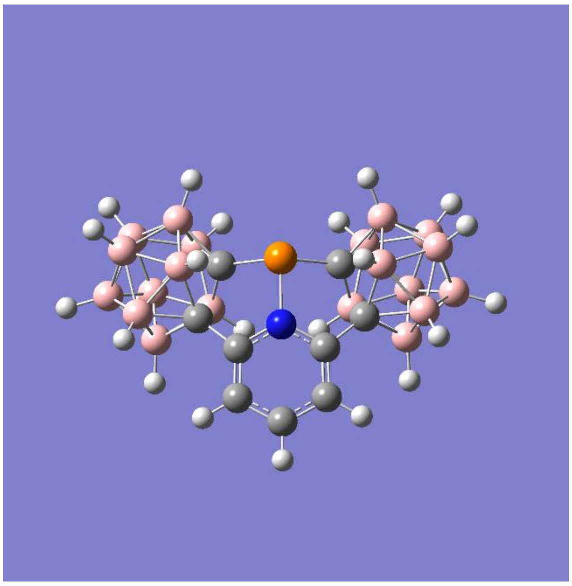

N	0.52224600	1.10209700	0.00000000
C	0.27055000	-0.83142600	1.63080800
C	0.07862500	0.67049200	2.27048800
C	0.23928500	1.68443200	1.18569700
C	-0.07442800	3.02892300	1.21696300
H	-0.29500600	3.51716400	2.16560900
C	-0.16592700	3.71234000	0.00000000
H	-0.40510800	4.77664600	0.00000000
B	-1.33733700	-0.29566000	1.93901500
H	-2.01066600	0.03946800	1.02024400
B	-0.80927900	-1.95808600	2.30098200

H	-1.21200900	-2.83054500	1.59231200
B	0.91030800	-1.90248500	2.78716300
H	1.67201400	-2.73843100	2.40016400
B	1.47799900	-0.21755100	2.74506200
H	2.51401300	0.13000300	2.26296600
B	-1.18178400	0.69627400	3.41424400
H	-1.85222800	1.68348300	3.47467800
B	-1.74228700	-1.00100300	3.49348500
H	-2.88781300	-1.25439500	3.72886300
B	-0.36091400	-2.00259300	4.02802200
H	-0.51114800	-3.00380500	4.66683900
B	1.04499700	-0.92999500	4.28723300
H	1.90566100	-1.14725700	5.08931600
B	0.53502400	0.74622700	3.90410000
H	1.02295100	1.76528400	4.29139900
B	-0.59641300	-0.36905800	4.71216600
H	-0.92423000	-0.17979300	5.84792200
C	0.27055000	-0.83142600	-1.63080800
C	0.07862500	0.67049200	-2.27048800
C	0.23928500	1.68443200	-1.18569700
C	-0.07442800	3.02892300	-1.21696300
H	-0.29500600	3.51716400	-2.16560900
B	1.47799900	-0.21755100	-2.74506200
H	2.51401300	0.13000300	-2.26296600
B	0.91030800	-1.90248500	-2.78716300
H	1.67201400	-2.73843100	-2.40016400
B	-0.80927900	-1.95808600	-2.30098200
H	-1.21200900	-2.83054500	-1.59231200
B	-1.33733700	-0.29566000	-1.93901500
H	-2.01066600	0.03946800	-1.02024400
B	0.53502400	0.74622700	-3.90410000
H	1.02295100	1.76528400	-4.29139900

B	1.04499700	-0.92999500	-4.28723300
H	1.90566100	-1.14725700	-5.08931600
B	-0.36091400	-2.00259300	-4.02802200
H	-0.51114800	-3.00380500	-4.66683900
B	-1.74228700	-1.00100300	-3.49348500
H	-2.88781300	-1.25439500	-3.72886300
B	-1.18178400	0.69627400	-3.41424400
H	-1.85222800	1.68348300	-3.47467800
B	-0.59641300	-0.36905800	-4.71216600
H	-0.92423000	-0.17979300	-5.84792200
P	1.15535600	-0.60116500	0.00000000

Zero-point correction= 0.384472 (Hartree/Particle)

Thermal correction to Energy= 0.404024

Thermal correction to Enthalpy= 0.404968

Thermal correction to Gibbs Free Energy= 0.341567

Sum of electronic and zero-point Energies= -1249.689410

Sum of electronic and thermal Energies= -1249.669859

Sum of electronic and thermal Enthalpies= -1249.668915

Sum of electronic and thermal Free Energies= -1249.732316

(III-F) at wb97xd/def2tzvp in $CHCl_3$

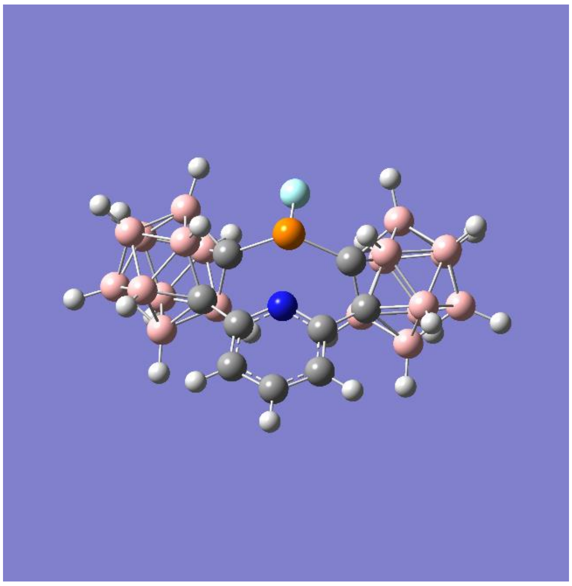

N	1.12965900	-0.42250800	0.00000000

C	-0.76661300	-0.18761900	1.70900300
C	0.76372800	-0.06623800	2.26589900
C	1.75058700	-0.27359300	1.16184800
C	3.13190500	-0.15255800	1.21547700
H	3.64722200	-0.03425500	2.16817000
C	3.82185300	-0.13752700	0.00000000
H	4.90881100	-0.04499400	0.00000000
B	-0.14958300	1.38821000	1.99769700
H	0.15232900	2.05932800	1.06298500
B	-1.79515000	0.94051100	2.47235900
H	-2.70479500	1.39315200	1.84522100
B	-1.78961300	-0.77362000	2.94972700
H	-2.69604900	-1.49002200	2.64735900
B	-0.14537700	-1.41976900	2.78921400
H	0.12578800	-2.47742900	2.30911600
B	0.92426300	1.18868400	3.40774100
H	1.94811000	1.80774600	3.40930200
B	-0.73048100	1.82661700	3.60009800
H	-0.91875100	2.98277800	3.85567500
B	-1.75331800	0.49321200	4.19763100
H	-2.70415300	0.68695100	4.90303300
B	-0.73451900	-0.95984300	4.38095800
H	-0.94048600	-1.81197500	5.19875000
B	0.92527800	-0.52531700	3.89037500
H	1.94817800	-1.05243000	4.21774200
B	-0.07381800	0.65246700	4.77519100
H	0.20462300	0.96595200	5.89924100
C	-0.76661300	-0.18761900	-1.70900300
C	0.76372800	-0.06623800	-2.26589900
C	1.75058700	-0.27359300	-1.16184800
C	3.13190500	-0.15255800	-1.21547700
H	3.64722200	-0.03425500	-2.16817000

B	-0.14537700	-1.41976900	-2.78921400
H	0.12578800	-2.47742900	-2.30911600
B	-1.78961300	-0.77362000	-2.94972700
H	-2.69604900	-1.49002200	-2.64735900
B	-1.79515000	0.94051100	-2.47235900
H	-2.70479500	1.39315200	-1.84522100
B	-0.14958300	1.38821000	-1.99769700
H	0.15232900	2.05932800	-1.06298500
B	0.92527800	-0.52531700	-3.89037500
H	1.94817800	-1.05243000	-4.21774200
B	-0.73451900	-0.95984300	-4.38095800
H	-0.94048600	-1.81197500	-5.19875000
B	-1.75331800	0.49321200	-4.19763100
H	-2.70415300	0.68695100	-4.90303300
B	-0.73048100	1.82661700	-3.60009800
H	-0.91875100	2.98277800	-3.85567500
B	0.92426300	1.18868400	-3.40774100
H	1.94811000	1.80774600	-3.40930200
B	-0.07381800	0.65246700	-4.77519100
H	0.20462300	0.96595200	-5.89924100
P	-0.93239900	-1.03619900	0.00000000
F	-2.57974400	-0.73781300	0.00000000

Zero-point correction= 0.386835 (Hartree/Particle)

Thermal correction to Energy= 0.407679

Thermal correction to Enthalpy= 0.408623

Thermal correction to Gibbs Free Energy= 0.342545

Sum of electronic and zero-point Energies= -1349.789794

Sum of electronic and thermal Energies= -1349.768950

Sum of electronic and thermal Enthalpies= -1349.768006

Sum of electronic and thermal Free Energies= -1349.834084

Flouride at wb97xd/def2tzvp in $CHCl_3$

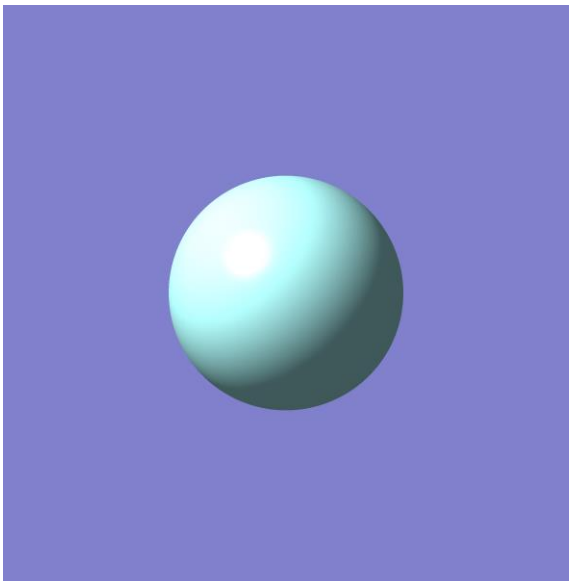

F 0.00000000 0.00000000 0.00000000

Zero-point correction= 0.000000 (Hartree/Particle)

Thermal correction to Energy= 0.001416

Thermal correction to Enthalpy= 0.002360

Thermal correction to Gibbs Free Energy= -0.014159

Sum of electronic and zero-point Energies= -99.956939

Sum of electronic and thermal Energies= -99.955523

Sum of electronic and thermal Enthalpies= -99.954578

Sum of electronic and thermal Free Energies= -99.971098

3. Refrences

(1) J. C. Axtell, K. O. Kirlikovali, P. I. Djurovich, D. Jung, V. T. Nguyen, B. Munekiyo, A. T. Royappa, A. L. Rheingold and A. M. Spokoyny, *J. Am. Chem. Soc.*, 2016, **138**, 15758–15765.

(2) C. Li, Q. Wang, J. Q. Zhang, J. Ye, J. Xie, Q. Xu, L. B. Han, *Green Chem.*, 2019, **21**, 2916–2922; (b) J. Plutnar, Z. Sofer, M. Pumera, *ACS Nano* 2018, **12**, 8390-8396.

(3) (a) A. Inoue, H. Shinokubo, K.Oshima, *J. Am. Chem. Soc.* 2003, **125**, 1484-1885; (b) P. E. Garrou, *Chem. Rev.* 1985, **85**, 171–185. (c) P. H. M. Budzelaar, J. A. van Doorn, N. Meijboom, *Recl. Trav. Chim. Pays-Bas*, 1991, **110**, 420–432.

(4) (a) C. E. Willans, C. A. Kilner, M. A. Fox, *Chem. Eur. J.*, 2010, **16**,10644– 10648; (b) Y. Taoda, T. Sawabe, Y. Endo, K. Yamaguchi, S. Fujiid, H. Kagechika, *Chem. Commun.*, 2008, 2049-2051; (c) M. A. Fox, K. Wade, *J. Organomet. Chem.*, 1999, **573**, 279–291; (d) M. G. Davidson, M. A. Fox, T. G. Hibbert, J. K. Howard, A. Mackinnon, I. S. Neretin, K. Wade, *Chem. Commun.*, 1999, 1649–1650.

(5) Z. Z. Han, C. P. Zhang, *Tetrahedron Lett.*, 2021, **73**, 153146.

(6) M. Pérez, L. J. Hounjet, C. B.Caputo, R. Dobrovetsky, D. W. Stephan, *J. Am. Chem. Soc.* 2013, **135**, 18308–18310.

(7) S. Rawat, M. Bhandari, B. Prashanth, S. Singh, *ChemCatChem* 2020, **12**, 2407 – 2411.

(8) F. Zheng, Z. Xie, *Dalton Trans.*, 2012, **41**, 12907-12914.

(9) For details on the fate of the decapitated B vertex, see the ESI and the following references: (a) Y. Taoda, T. Sawabe, Y. Endo, K. Yamaguchi, S. Fujiid, H. Kagechika, *Chem. Commun.* 2008, 2049–2051; (b) M. G. Davidson, M. A. Fox, T. G. Hibbert, J. K. Howard, A. Mackinnon, I. S. Neretin, K. Wade, *Chem. Commun.* 1999, 1649–1650.

(10) (a) T. E. Mallouk, G. L. Rosenthal, G. Muller, R. Brusasco, N. Bartlett. *Inorg. Chem.* 1984, **23**, 3167-3173; (b) K. O. Christe, D. A. Dixon, D. McLemore, W. W. Wilson, J. A. Sheehy,J. A. Boatz, *J. Fluorine Chem.* 2000, **101**, 151–153; (c) P. Erdmann, J. Leitner, J. Schwarz, L. Greb, ChemPhysChem 2020, **21**, 987; (d) R. E. Rosenberg, *J. Am. Chem. Soc.* 1995, 117, 10358–10364; (e) R.Vianello, Z. B. Maksić, *Inorg. Chem.* 2005, **44**, 1095–1102; (f) H. Böhrer, N. Trapp,D. Himmel, M. Schleep, I. Krossing, *Dalton Trans.* 2015, **44**, 7489–7499; (g) Z. M. Heiden, A. P. Lathem, *Organometallics* 2015, **34**, 1818–1827; (h) S.Ilic, A. Alherz, C. B. Musgrave, K. D. Glusac, Chem. Soc. Rev. 2018, **47**,2809–2836.
